# Forecasting vaping health risks through neural network model prediction of flavour pyrolysis reactions

**DOI:** 10.1038/s41598-024-59619-x

**Published:** 2024-05-08

**Authors:** Akihiro Kishimoto, Dan Wu, Donal F. O’Shea

**Affiliations:** 1 IBM Research - Tokyo, Shin-Kawasaki, Japan; 2 Department of Chemistry, Royal College of Surgeons in Ireland (RCSI), Dublin 2, Ireland

**Keywords:** Cheminformatics, Public health

## Abstract

Vaping involves the heating of chemical solutions (e-liquids) to high temperatures prior to lung inhalation. A risk exists that these chemicals undergo thermal decomposition to new chemical entities, the composition and health implications of which are largely unknown. To address this concern, a graph-convolutional neural network (NN) model was used to predict pyrolysis reactivity of 180 e-liquid chemical flavours. The output of this supervised machine learning approach was a dataset of probability ranked pyrolysis transformations and their associated 7307 products. To refine this dataset, the molecular weight of each NN predicted product was automatically correlated with experimental mass spectrometry (MS) fragmentation data for each flavour chemical. This blending of deep learning methods with experimental MS data identified 1169 molecular weight matches that prioritized these compounds for further analysis. The average number of discrete matches per flavour between NN predictions and MS fragmentation was 6.4 with 92.8% of flavours having at least one match. Globally harmonized system classifications for NN/MS matches were extracted from PubChem, revealing that 127 acute toxic, 153 health hazard and 225 irritant classifications were predicted. This approach may reveal the longer-term health risks of vaping in advance of clinical diseases emerging in the general population.

## Introduction

The delivery of nicotine to the lungs through the inhalation of tobacco smoke has been practiced by mankind for centuries with devastating impacts on public health^1^. Relatively recently, vaping of e-liquids has emerged as a modern variant of this ancient practice. In their original construction, the constituents of e-liquids contained only four chemical entities, nicotine, propane-1,2-diol, propane-1,2,3-triol and water, with the goal of providing a less hazardous means of nicotine delivery than tobacco leaf^2^. Their use as an aid for tobacco smoking cessation has evolved as a cornerstone of some national public health policies, though others have restricted or prohibited their use^3^.

Soon after their first commercialization in the mid 2000’s, the number of chemical entities used in vaping e-liquids dramatically increased as an array of flavours were added. Currently, at least 180 discrete chemicals are known to be in use in e-liquids, blended in various amounts to produce a specific flavour branded product^4^. A European based study identified a mean and range of 6 (±) 4 chemical flavours used per specific e-liquid product, whereas a comparable US study found a range of 22 to 47 chemical flavours per e-liquid^5,6^. In both studies it was found that the total flavour chemical concentration in the majority of e-liquids exceeded that of nicotine. Furthermore, several studies have shown that flavoured e-liquids are linked to a lowering of the vaping age demographic^7^. Their appeal to non-smoking teenagers and young adults has led to a divergence of opinion on their use as an aid for tobacco smoking cessation in the established smoking population^8^. Concerns are growing that vaping in younger generations jeopardizes the decline in nicotine use and also risks the emergence of future vaping induced diseases^9^. Yet, while the strongly polarized debates about the pros and cons of vaping are ongoing, the implications for long-term effects on public health, morbidity and mortality are simply unknown^10–13^. While the health risks from exposure to the carcinogenic chemicals in tobacco smoke are known, it can take decades of accumulative damage before clinical manifestation of disease occurs.

Intuitively for vaping, it would be reasonable to anticipate that lung exposure to a large number of chemical entities can only increase health risks. In 2019, the potential for vaping health risks became apparent when cases of acute lung injury emerged attributable to tetrahydrocannabinol vaping products. E-cigarette or vaping use-associated lung injury (EVALI) statistics from the CDC document 2807 hospitalizations and 68 deaths over one year in the United States. A single chemical additive, vitamin E acetate (VEA), has been strongly linked to the outbreak that ended once its use stopped^14^. Our previous research showed that the action of pyrolysis heating within a vaping device could transform VEA into more than ten different substances including the highly toxic gas ketene which could account for the severe lung injuries^15^. While the chemicals used for nicotine vaping are different from tetrahydrocannabinol products^16,17^, the number of chemicals is considerably higher. Prolonged exposure to these chemicals and their pyrolysis products makes it plausible that we are standing at the starting line of a new wave of chronic diseases that will only emerge in 15 to 20 years from now.

The chemicals used as e-liquid flavours are not specifically developed for vaping and are adopted from the food industry^18^. Much like VEA, which is also widely used in foodstuffs and cosmetics, these compounds have a good safety record for these specific uses. However, it was not envisaged that they would be used in a significantly different manner that involves heating to high temperatures with inhalation into the lungs. Remarkably, there are a myriad of different vaping devices whose operating temperature ranges are often unknowingly determined by user preferences. Studies have measured typical temperatures ranging from 100 to 400 °C depending upon factors such as power, heating coil materials, puff size and e-liquid quantity, with dry coil temperature measured above 1000 °C^19,20^. Pyrolysis decomposition of flavours at these temperatures could produce large numbers of unknown secondary chemical entities, thereby hugely amplifying the health risks from each flavour. In contrast to tobacco smoking, combustion products are minor in vaping so were not included in this initial study.

As hundreds of chemicals are used in tens of thousands of commercial e-liquid products, the experimental analysis of all their vaping induced chemistries and associated products could take decades of research. In this study, a holistic research strategy employing artificial intelligence (AI) was adopted to simultaneously investigate all flavours in e-liquids. AI is increasingly being used to perform chemistry tasks such as retrosynthetic route planning, the prediction of reaction outcomes and the acceleration of drug discovery^21–23^. Currently, a unique opportunity exists to exploit AI to anticipate vaping risks in advance of their public health impact, which may take years to emerge.

### Overview of 180 e-liquid flavour chemicals

**Figure 1:**
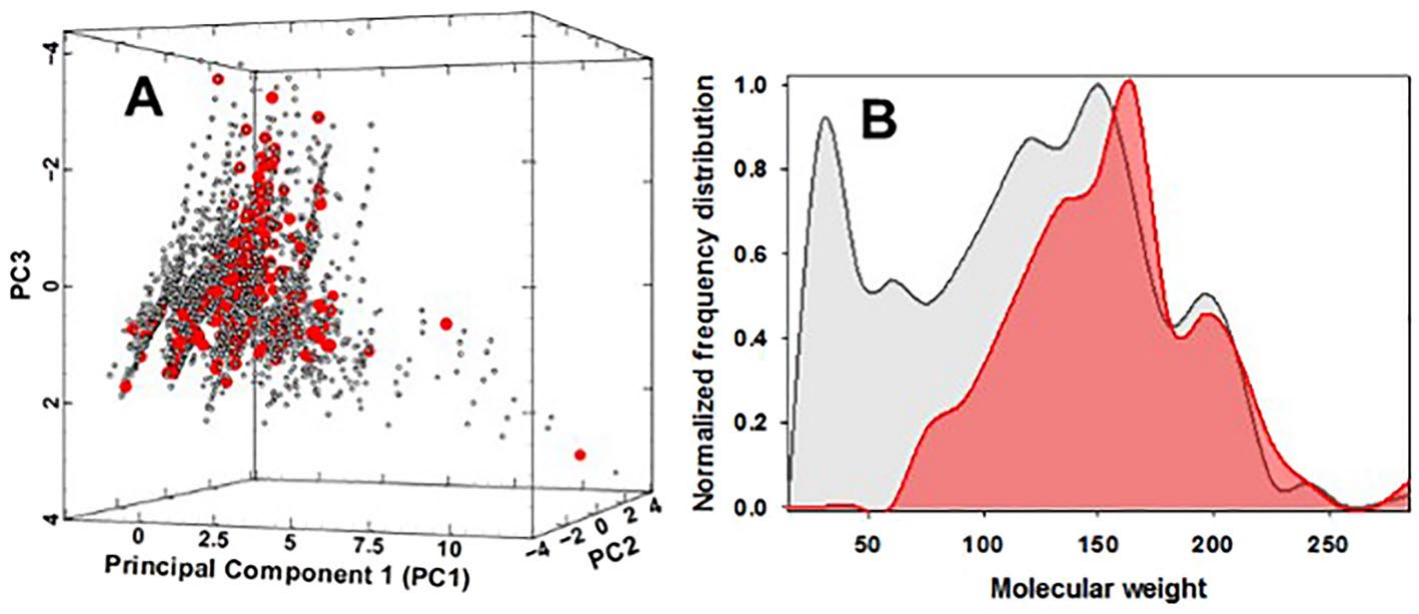
Chemistry diversity analysis of 180 flavour chemicals and their predicted pyrolysis products. (**A**) 3D representation of the chemical space occupied by 180 e-liquid compounds (red circles) and their discrete 4524 NN predicted pyrolysis products (grey circles). Principal component (PC) scale refers to normalized projections of the six molecular properties. (**B**) Molecular weight distribution of 180 e-liquid compounds (red distribution profile) and their discrete 4524 NN predicted pyrolysis products (grey distribution profile).

While the exact number of flavour chemicals in current worldwide e-liquid use is unclear, 180 representative chemicals known to be used as flavourings in e-liquids were chosen for this study based on literature reports^4–6,18^. Structural inspection of the chemical functional groups within the 180 flavours revealed 66 esters, 46 ketones/aldehydes, 27 alcohols/acetals, 26 aromatics/heterocycles/carbocycles and 15 carboxylic acids/amides, clearly indicating the potential for a wide range of pyrolysis reactions (Supplementary Table S1). Flavour structural diversity was analyzed by comparing their molecular weight, hydrogen bond donors/acceptors, topological polar surface area, number of rotatable bonds, and octanol–water partition coefficient properties^24^. A 3D visualization of the chemical space reflecting these six properties shows that compounds are clustered in a similar area, indicating moderate diversity with 85.5% of the variance accounted for by molecular weight, surface area and number of rotatable bonds (Fig. 1A, red circles, Supplementary Dataset S1). Their mean molecular weight was 146.2 signifying a relative volatile set of molecules (Fig. 1B, red distribution profile).

### Workflow for e-liquid flavour risk identification and classification

**Figure 2:**
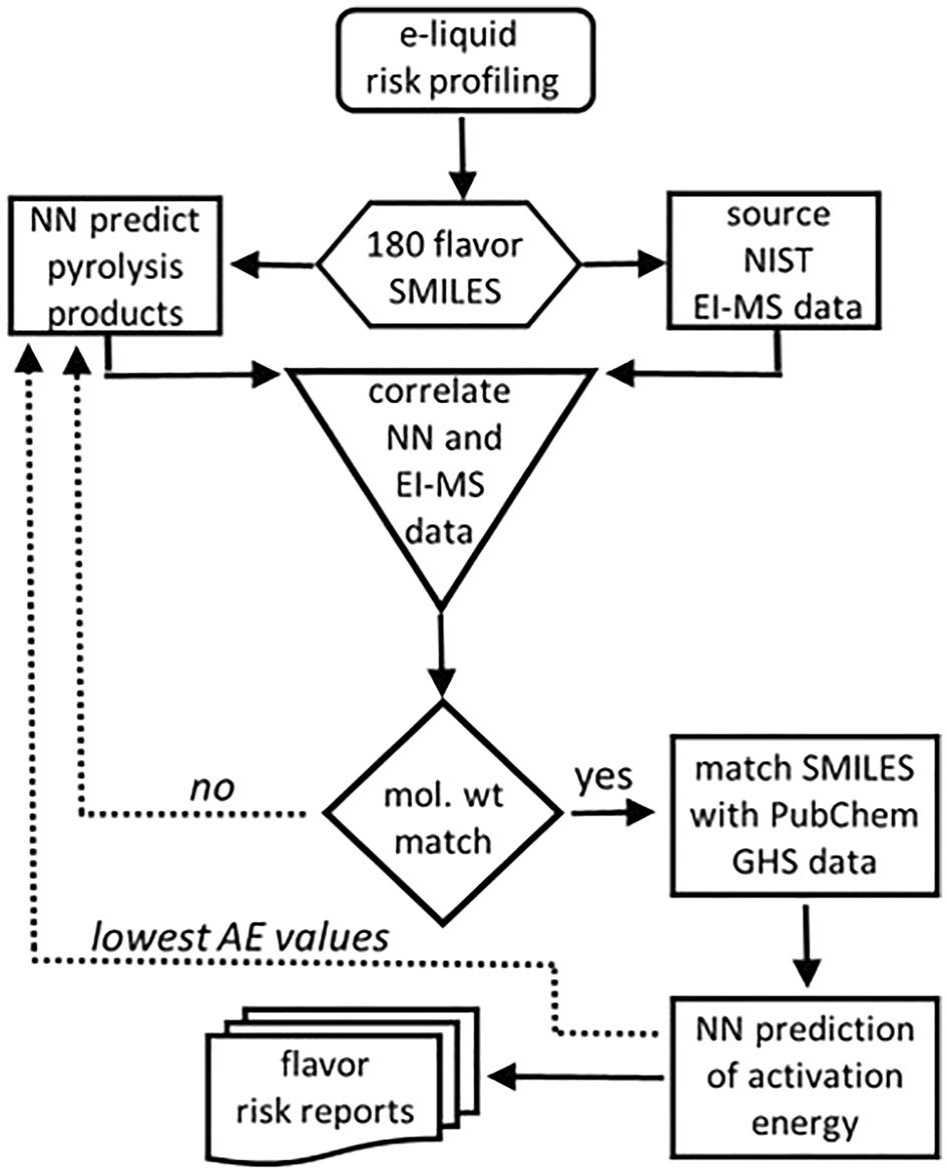
Workflow chart for the pyrolysis risk identification of vaping e-liquid components (solid arrows). Dashed arrows indicate future scope for an informative feedback into NN pyrolysis predictor.

All 180 flavours were subjected to a common workflow that blended NN pyrolysis prediction with experimental electron-impact mass spectrometry (EI-MS) data. An overview of each stage is shown in Fig. 2, which started with transcribing the 180 chemical structures into their simplified molecular-input line-entry system (SMILES) format. A graph-convolutional neural network model was used to predict pyrolysis chemical transformations and their associated products for each flavour. Experimental MS data containing the molecular ion, associated fragmentation masses and their relative abundances were sourced for each flavour. As both pyrolysis reactions and MS fragmentations involved energy induced bond breaking; a correlation between both was anticipated. Using specifically written script, the molecular weight of each NN predicted product from each flavour was correlated against the MS fragmentation masses for that flavour. A data subset was formed containing NN-reactions with a predicted product which matched a MS fragmentation mass. Next, the GHS classification was identified for each NN/MS matched product. A second NN was used to predict activation energies (AE) for reactions producing products with the most significant health implications. Data collation generated an enumerated list of NN predicted products, MS matched products and their associated GHS hazard classification for each of the 180 flavours (Fig. 2). Each step was automated and could accept new compound inputs as required.

### Graph-convolutional neural network model for pyrolysis products prediction

**Figure 3:**
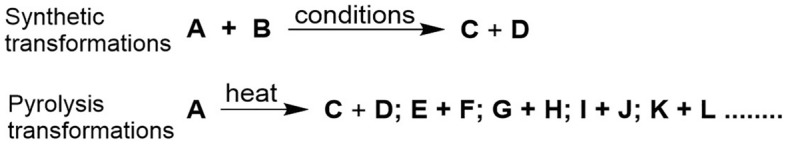
Synthetic and pyrolysis transformations.

To date, reaction prediction methods have primarily focused on synthetic transformations in which at least two reactants generate a product and a byproduct under varying experimental conditions^25–27^. Pyrolysis reactions differ in that a single reactant produces an array of lower molecular weight products by different transformation pathways with heat being the driving force of the reactions (Fig. 3).

**Figure 4:**
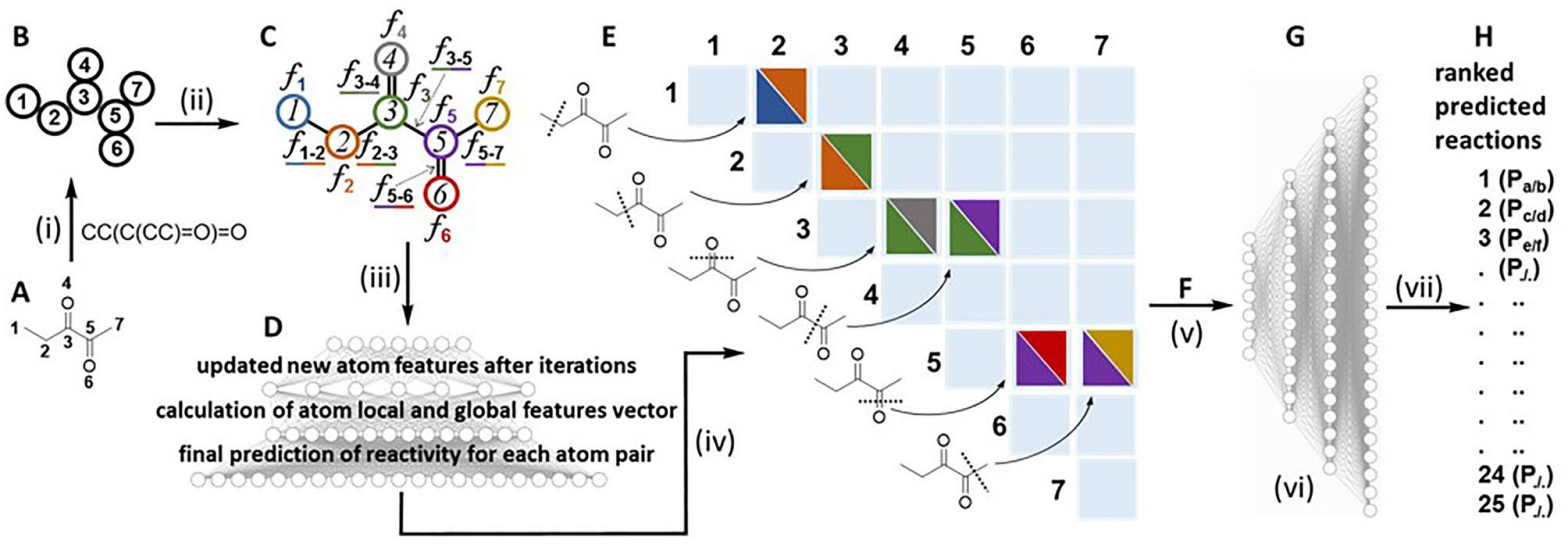
W–L neural network for predicting bond changes between every pair of atoms in 2,3-pentandione. (i) Molecular SMILES converted to attributed graph. (ii) Atom descriptors generated by incorporating information from neighboring atoms. (iii) Updated new atom features after iterations, calculation of atom local and global features vector and final prediction of reactivity for each pair of atoms. (iv) Calculated scores for each likelihood bond change by W–L neural network. (v) Potential products enumerated after removal of those failing chemical valence rules. (vi): W–L difference network model for ranking pyrolysis reactions enumerated based on the most probable bond changes. (vii) Predicted pyrolysis reactions ranked from 1 to 25 and their associated products (P).

It was found that the previously described Weisfeiler–Lehman neural network (W–L NN) model suited our requirements as it operates by prediction of reaction centers based on bond changes for every pair of atoms in a molecule^26^. As a graph convolutional network, it can predict unimolecular pyrolysis transformations without any training data specific to pyrolysis reactions. Supervised learning of the W–L NN was achieved using US patent literature as a source of data, with pyrolysis predictions based on a training set of 354,937 reactions^26,28,29^. For this study, only first phase pyrolysis products were considered with further pyrolysis of initial pyrolysis products not included. All reactions that included flavour molecules were removed from the training data to ensure that no characteristics of these flavour molecules were leaked before their pyrolysis predictions were performed. Prevention of such data leakage allows the performance assessment of the trained W–L NN model without bias, even if a new flavour molecule is passed to the trained model. As designed, the W–L NN architecture embedded the inherent computations in the W–L graph kernel to learn atomic representations. This starts by converting chemical SMILES (notation to describe a chemical structure that can be understood by computer software) to attributed graph representations of molecules. For example, the SMILES for flavour 2,3-pentanedione being CCC(=O)C(=O)C converts to a labelled form of atoms 1 to 7 as shown in Fig. 4A,B. Each atom representation was computed by including contributions from adjacent atoms such as atom 3 with atom 2, 4, and 5. Specifically, each atom was initialized with a feature vector *f*_*atom*_ indicating its key properties such as atomic number, connectivity, valence, formal charge, and aromaticity. Representation of the bond order (number of chemical bonds between a pair of atoms) and connectivity of each bond was through the feature vectors *f*_*bond*_ (Fig. 4C). Local feature vectors were calculated for each atom based on its representation and those of other atoms directly bonded to it. Next, global atom features were produced for each atom to account for the influence of atoms not directly bonded to it. Finally, a combination of local and global feature vectors was used to predict the likelihood of bond changes for each pair of atoms (Fig. 4D).

In the representative input example of 2,3-pentandione, all atom pairs were “tested” to identify high probability bond breaking positions (Fig. 4E). Up to 16 likely bond-breaking positions were identified to enumerate their possible pyrolysis transformations and associate output products. Up to five simultaneous chemically feasible bond changes per pyrolysis reaction were allowed. Any predicted products that did not comply with chemical valence rules (correct number of bonds from each atom) were removed (Fig. 4F). Next, a W–L difference network (W–L DN) generated a probability score for each predicted pyrolysis transformation based on the differences in atom representations between the products and the original molecule (Fig. 4G). The W–L DN then selected and ranked the twenty-five most likely transformations based on their probability scores (Fig. 4H). Analysis of the NN output of 4500 pyrolysis predictions for the 180 flavours showed 7307 products (Supplementary Dataset S2). When duplicate products from the same flavour are not included, the total number was 4524. The average number of discrete products per flavour was 25.1 with a greater number predicted for compounds of larger molecular size and complexity. The top 20 predicted pyrolysis products (excluding duplicates arising from the same flavour) included alkanes, alkene, alcohols, aldehydes, acids, and aromatics, as shown in Table 1.

**Table 1:** Top 20 most commonly W-LNN predicted pyrolysis products.

Product	Times predicted	Product	Times predicted
CH_4_	171	CH_3_CO_2_H	29
H_2_O	129	CH_2_ = CH_2_	22
C_2_H_6_	82	C_4_H_9_OH	20
C_3_H_8_	71	CH_2_ = C = O	20
CH_2_ = O	63	CH_2_ = CHCH_3_	20
C_2_H_5_OH	53	C_5_H_12_	19
CH_3_OH	50	C_3_H_7_CH = O	18
CH_3_CH = O	41	C_6_H_5_CH_3_	18
HCO_2_H	32	C_6_H_6_	18
C_4_H_10_	31	CH_3_C(O)OC_2_H_5_	18

Structural diversity of the 4524 predicted products was determined using the same molecular parameters used for the 180 flavours (23). The 3D chemical space visualization showed the NN-predicted pyrolysis products clustered in a similar space as their originating flavours (Fig. 1A, grey circles, Supplementary Dataset S1). The expected difference was a significant shift to lower molecular weight compounds, with a mean molecular weight of 111.7 indicating the production of highly volatile organic compounds (Fig. 1B, grey distribution profile).

### Sourcing experimental EI-MS data for each e-liquid flavour

**Figure 5:**
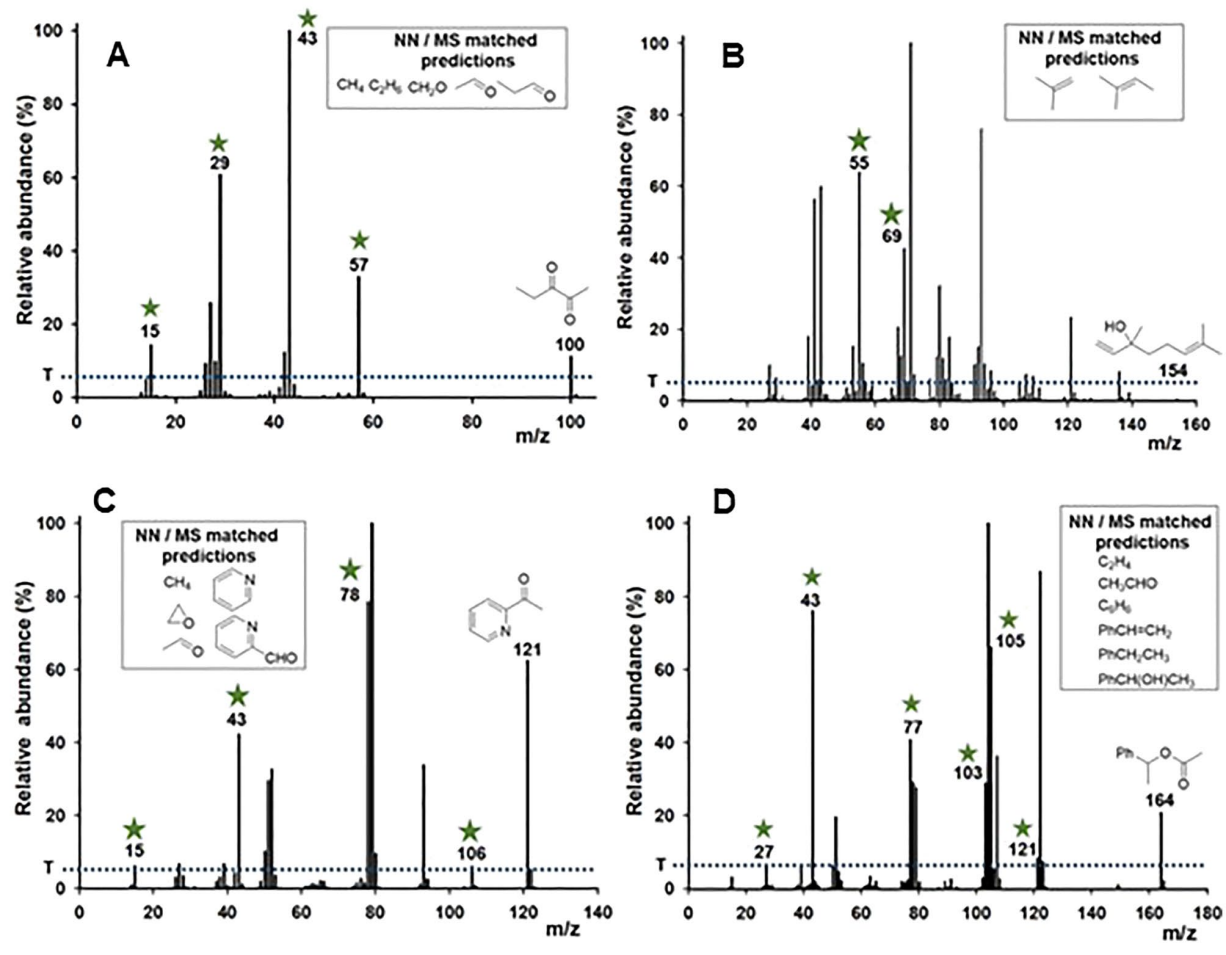
Representative flavour EI-MS data of (**A**) 2,3-pentandione; (**B**) linalool; (**C**) 2-acetyl pyridine; (**D**) α-methylbenzyl acetate from the NIST database. Threshold (T) set at 5% relative abundance indicated by blue dotted line. Green asterisk indicates the molecular weight matches with W–L NN predicted products. Insets show structures of NN-predicted products that are molecular weight matched with an MS fragmentation.

Mass spectrometry fragmentation identifies intramolecular bond breaking positions that occur as a result of molecular interaction with the applied energy from the instrument source. As pyrolysis is a heat induced bond breaking process, a correlation between both can exist^30^. Experimental EI-MS mass data was retrieved, using Python script, from the online National Institute of Standards and Technology (NIST) database for each of the 180 e-liquid flavours^31,32^. Data obtained included the molecular weight of all fragmentations from the parent ion and their relative abundance. Representative flavour examples for 2,3-pentandione, linalool, 2-acetyl pyridine and α-methylbenzyl acetate in Fig. 5, show their MS fragmentation patterns and corresponding molecular weights. Specifically for 2,3-pentandione, the series of fragmentation masses (% relative abundance) of 100 (11), 57 (32), 43 (100), 42 (12), 29 (60), 27 (25) and 15 (14) can be seen which correspond to the molecular ion and the most likely bond breaking positions of the molecule (Fig. 5). In this way, the MS fragmentation data for each e-liquid component can act as a minable dataset to identify molecular weight alignments with the products from the NN predicted pyrolysis reactions. A 5% relative abundance threshold for each mass peak was applied to the MS data to eliminate the possibility of instrument noise or isotope contributions. The average number of mass fragmentation peaks per e-liquid component was 16.5 with the maximum at 54 and the minimum at 2. As expected, larger molecular weight compounds typically have more fragmentation mass peaks than those of lower weight.

### Amalgamation of W–L NN and EI-MS data

Next, automated amalgamation of in silico NN with experimental MS data was carried out^32^. The goal of merging these two information sources was to identify the most likely pyrolysis products for each flavour, from which their health risks could be assigned. Correlation of molecular weights of NN predicted products with their experimental MS fragmentation masses identified 1169 discrete matches between the two datasets (not counting repeat matches for a flavour) (Supplementary Dataset S2). The average number of NN/MS matches per flavour was 6.4 with 92.8% having at least one match and 86% having more than one match. Examples of specific NN/MS matches are shown in Fig. 5 for four structurally different flavours. Green asterisks indicate the molecular weight matches in the mass spectral data with W–L NN predicted products and the insets show structures of the matched compounds. It is noteworthy that this data amalgamation was successful for a wide variety of different molecular structures and functional groups.

**Figure 6:**
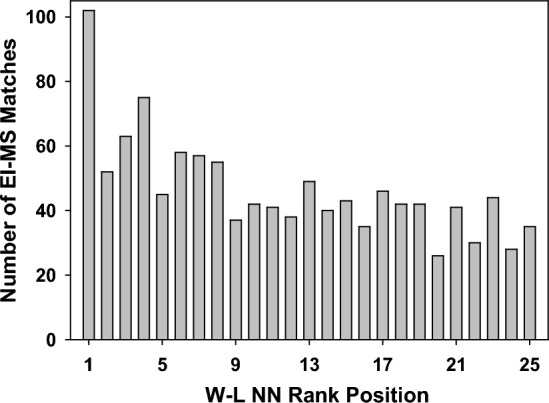
Comparative plot of W–L NN rank position from 1 to 25 for predicted products matched with experimental EI-MS data.

Encouragingly, plotting the number of MS matches against the NN rank position for each predicted product shows a clear bias towards higher rank positions, with the highest NN-rank 1 accounting for 8.7% of all matches (Fig. 6, rank 1). Comparison of the cumulative number of matches for the top (1–5) and bottom (21–25) rank positions show that the higher positions accounted for 29% of all matches whereas the lower ranks accounted for only 15% (Fig. 6). These correlations indicate that NN predicted products could be substantiated through experimental MS fragmentations and that in future work MS data could be used in a hybrid supervised and reinforcement learning model. The non-matched W–L NN predicted products were not used further in this work but may serve as an informative feedback allowing future refinement of the NN pyrolysis predictor (Fig. 2, dashed arrow).

Examining the most commonly matched compounds it was encouraging to find that a broad distribution of molecular classes was matched (saturated, unsaturated and aromatic hydrocarbons, aliphatic alcohols and carboxylic acids) (Table 2). Seventeen of the top twenty W–L NN predicted products (Table 1) were also in the top 20 matched compounds, further increasing confidence in the NN/MS matched predictions. Next, the health risk of each NN/MS matched product was identified.

**Table 2:** Top 20 most commonly W-L NN predicted products matched with experimental EI-MS data.

Product	Times matched	Product	Times matched
C_2_H_6_	64	C_6_H_6_	16
C_3_H_8_	60	CH_3_OH	15
CH_3_CH=O	37	C_3_H_7_CH=O	15
CH_2_=O	36	CH_2_=CHOH	14
C_4_H_10_	26	C_6_H_5_CH_3_	13
HCO_2_H	23	CH_2_=C=O	10
C_2_H_5_OH	22	C_5_H_12_	10
CH_4_	21	C_2_H_5_CO_2_H	10
CH_2_=CH_2_	19	H_2_C=CHC_2_H_5_	9
CH_2_=CHCH_3_	17	C_4_H_9_OH	8

### Acquisition of risk assessment data for W–L NN and EI-MS matched products

**Figure 7:**
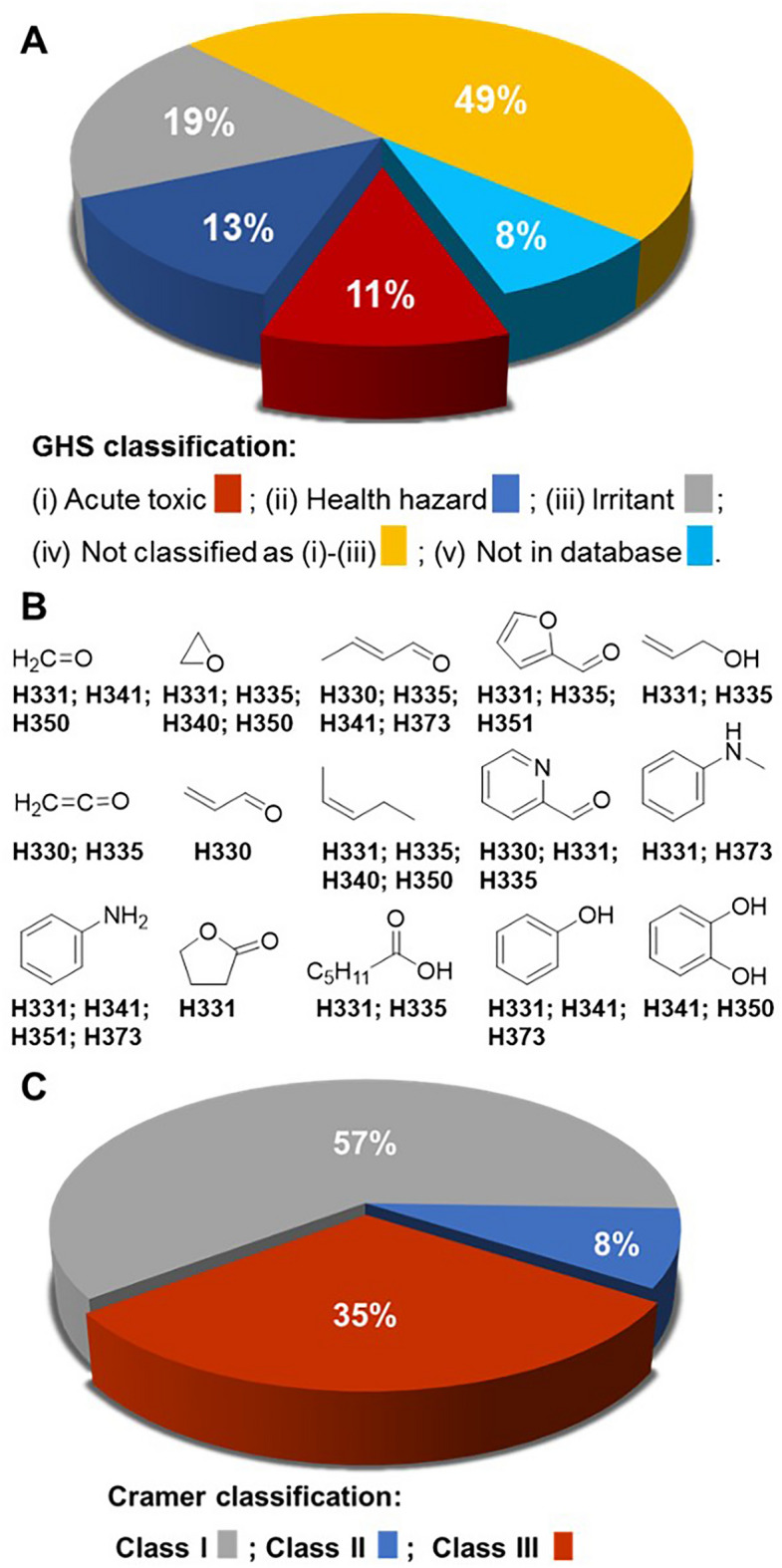
(**A**) Distribution of globally harmonized system classifications of W–L NN/MS matched products (for compounds with more than one classification only the most serious classification is included). (**B**) Representative examples of the structure for W–L NN/MS predicted compounds with acute toxic GHS classification and their specific inhalation hazard warning. GHS hazard statements: H330 fatal if inhaled; H331 toxic if inhaled; H335 may cause respiratory irritation; H340 may cause genetic defects; H341 suspected of causing genetic defects; H350 may cause cancer; H373 causes damage to organs through prolonged or repeated exposure. (**C**) Distribution of Cramer classifications of W–L NN/MS matched products.

Using specifically written Python script, the GHS classifications for each NN/MS matched product was obtained from the open-source PubChem database^32,33^. The script used the SMILES string of each compound as a query keyword to identify matching URLs within the site. Within each URL, the hazard statements in the GHS classification section were downloaded in JSON format. Three different classification categories were used to build each flavour risk profile (i) acute toxic; (ii) health hazard; or (iii) irritant. In addition, a category (iv) was used to group compounds not classified as either (i), (ii) or (iii) but that may have other hazard warnings, and category (v) was compounds for which a search query did not produce a result, indicating they were not in the database (Supplementary Dataset S3). The GHS classifications of acute toxic (127 compounds), health hazard (153 compounds) and irritant (225 compounds) accounted for 11%, 13% and 19% of classifications attributed to the dataset respectively (Fig. 7A). Only 49% of products were not included in the categories (i) to (iii) and 8% had no classification information available. Mining classification data specifically for inhalation health hazards revealed further insights into their health risks with a representative selection of these results for a structurally diverse set of the functional compounds shown in Fig. 7B (Supplementary Dataset S3). It is noteworthy that while some similarities to compounds produced by tobacco smoke exist (e.g. formaldehyde, ethylene oxide, aromatic amines), many others differ such as α,β-unsaturated carbonyl compounds (aldehydes, ketones, esters), heterocycles and phenols. This is due to the diverse chemical makeup of the individual vaping flavours, which differ from the natural products found within tobacco leaf. This indicates that, while related, vaping biomarkers and their clinical disease manifestations could differ significantly from those of tobacco smoking^34^. Furthermore, vaping biomarkers are likely to differ based on commercial e-liquid product, as the spectrum of pyrolysis products differ for each chemical flavour^35^.

Additionally, NN/MS matched products were grouped using the three Cramer classes (Supplementary Dataset S4)^36^. Cramer classification is a commonly used predictive approach for classifying chemicals on the basis of their expected level of oral toxicity. Cramer Class III, which represents the most severe potential toxic hazard, accounted for 35% of these compounds with Class II (moderate risk) and Class I (low risk) accounting for 8 and 57% respectively (Fig. 7C). It was noteworthy that many of the more commonly used flavour chemicals (e.g. *cis*-3-hexenol, isoamyl acetate, benzaldehyde, ethyl hexanoate, cinnamaldehyde, benzyl acetate, hexyl acetate) had one or more predicted products identified as Class III. Across all 180 flavours, the most common Cramer III classifications were for benzene, ketene and ethylene oxide predicted by 16, 10 and 8 different flavours respectively (Supplementary Dataset S4).

### W–L NN prediction of pyrolysis activation energies

The activation energy (AE) of a chemical reaction is the minimum energy required for a reaction to proceed. With respect to vaping, AEs are an excellent means of obtaining a first approximation of the thermal conditions required for pyrolysis to occur. Yet, determination of AEs is experimentally very laborious and computationally expensive, as it requires quantum chemical calculations. As such, the use of NN methods to obtain quantitative values for flavour pyrolysis reactions would be of significant value. To address this goal, a recently reported directed message passing neural network (D-MPNN) for AE predictions has been employed^37,38^. D-MPNN is a graph convolutional neural network, similar to that used for pyrolysis predictions described earlier, though it should be noted that other NN methods have been employed for AE predictions^39,40^. The training data used consisted of published gas phase energy activation data of 16,264 transformations determined by quantum chemistry calculations using B97-D3/def2-mSVP theory following the exclusion of flavour compounds to prevent data leakage of the test set^38^. AEs for 482 NN predicted reactions were determined. The reactions were chosen to reflect different transformation types and reactions that generated products classified as high health risk were prioritized (Supplementary Dataset S5). The outcome gave a wide range of AE values from 45 to 121 kcal/mol indicating that comparisons could be made between different degradation pathways for each flavour.

**Figure 8:**
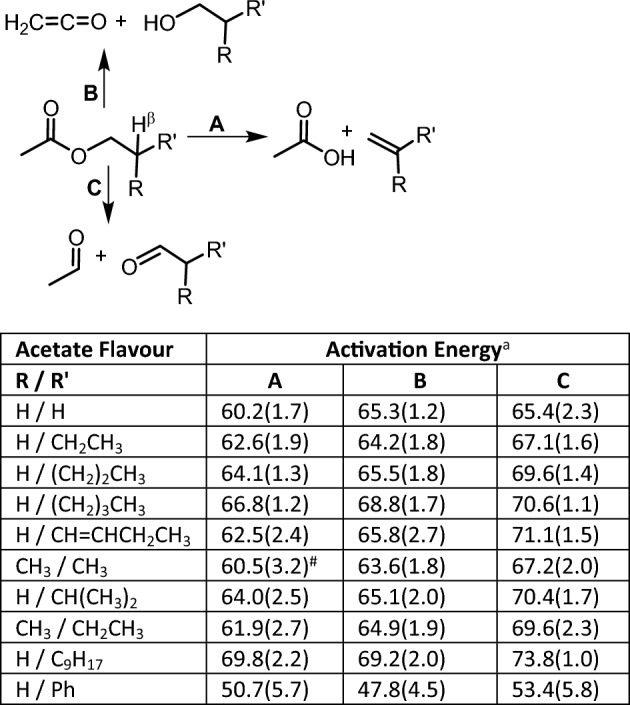
D-MPNN derived activation energies applied to three different NN predicted pyrolysis pathways of acetate fruit flavours (ethyl acetate, butyl acetate, amyl acetate, hexyl acetate, *cis*-3-hexenyl acetate, isobutyl acetate, isoamyl acetate, 2-methylbutyl acetate, citronellyl acetate, 2-phenylethyl acetate). ^a^kcal/mol, ^#^not predicted by NN.

Fruit flavoured products are the most popular commercial brands for the younger vaping demographic so warrant particular attention. These compounds commonly have an ester functional group which are known to undergo thermal decomposition by different elimination and free radical β-scission reactions, both of which are plausible under vaping conditions^41,42^. To illustrate use of AE values, ten acetate esters with substituent containing β-hydrogens were selected for comparative data analysis (Fig. 8). Previously reported experimental and computational studies have mostly focused on the simplest derivatives such as ethyl acetate with others as of yet unstudied^42–45^. These results show that three different elimination pathways are possible to generate either acetic acid and substituted alkenes (pathway A); ketene with substituted alcohols (pathway B); or C–O cleavage resulting in the formation of two carbonyls (pathway C)^42^. Analysis of the NN predicted reactions for each of these acetates showed that these transformation pathways were common to all. Comparison of the D-MPNN derived AE values for these reactions showed that pathway A consistently predicted the lowest energy requirement for most of the acetates (Fig. 8, table). The identification of pathway A as most favorable is consistent with literature reports and thus identifies inhalation of acetic acid and substituted alkenes as the likely health hazards^46^. It is noteworthy that of the ten different alkenes producible via AE favored pathway A, eight are GHS classified as either irritant or health hazard (ethene, hexene, 1,3-hexadiene, 2-methylpropene, 3-methyl-1-butene, 2-methyl-1-butene, 3,7-dimethylocta-1,6-diene, styrene). Additionally, it is important to recognize that due to the complex reacting conditions within a vaping device, pyrolysis would not be expected to follow a single pathway^47^. In the case of acetates, products could also occur via free radical β-scission reactions as vaping conditions have been shown to promote radical type reactions^48,49^.

While these AE values are, as yet, a first approximation, taking no account of the conditions under which reactions are taking place, their importance will grow as the accuracy in predicting these values improves. It could be envisaged that they play a future role in reinforcement learning models in conjunction with MS fragmentation data (Fig. 2, dashed arrows).

### E-Liquid flavour reports

Collation of all data generated an output for each of the 180 flavours with an enumerated list of NN predicted reactions and their associated products, EI-MS matched products identified and their associated GHS hazard classifications (Supplementary Datasets S2, S3). Taken together, these constitute a minable reference source that encompasses the complex and interconnected facets of vaping with the potential to be refined and adapted in the future.

## Discussion

The e-liquid marketplace is vast and growing, driven by increased investment by tobacco companies into vaping products^50^. The original source of flavours in e-liquids stems from food flavouring compounds so it could be anticipated that the number of compounds being used will increase over time^4–6^. Since their inception, an incorrect assumption has grown that the flavour ingredients used in e-liquids are designated “generally recognized as safe” (GRAS) under health regulations. However, this GRAS status only relates to human consumption via ingestion (compatible with their use in foodstuffs) and not inhalation following thermal activation^18^. While the health concerns for lung exposure to the flavours themselves are serious, what is even more concerning is the array of thermal degradation products which they generate as a consequence of their heating immediately prior to inhalation^51^. The vast majority of these degradation products remain unknown as do their health consequences from long-term exposure. From a public health perspective, the use of flavours in e-liquids can be viewed as a double-edged sword. The role for vaping flavours is cited as a support for smoking cessation for those already addicted to nicotine tobacco products, but the same flavours are the main attractant for a non-smoking younger demographic^8,52^.

Experimental research into the heat-induced breakdown of organic compounds has its origins in the early twentieth century with the seminal work of Hurd and others^53^. Such research was conducted to gain fundamental scientific insights into the nature of chemical bond dissociations and formations. Vaping devices can be considered as crude versions of a laboratory pyrolysis apparatus^54^. Both are designed to rapidly heat organic molecules to high temperatures, although when using an experimental apparatus the products are safely trapped, quantified and characterized whereas in vaping they are drawn into the lungs. A laboratory pyrolysis apparatus has rigorous control over temperature, is made from materials to limit radical formation and is used to study test molecules individually. In contrast, a vaping device has poor temperature control, is constructed using metal materials that induce radical reactions and simultaneously heats an array of chemical entities in an e-liquid. By its nature, experimental pyrolysis chemistry is highly complex, but within vaping this complexity is magnified due to e-liquid, device and user variabilities making it a daunting task to map all possible chemical outcomes from a vaping “experiment”.

**Figure 9:**
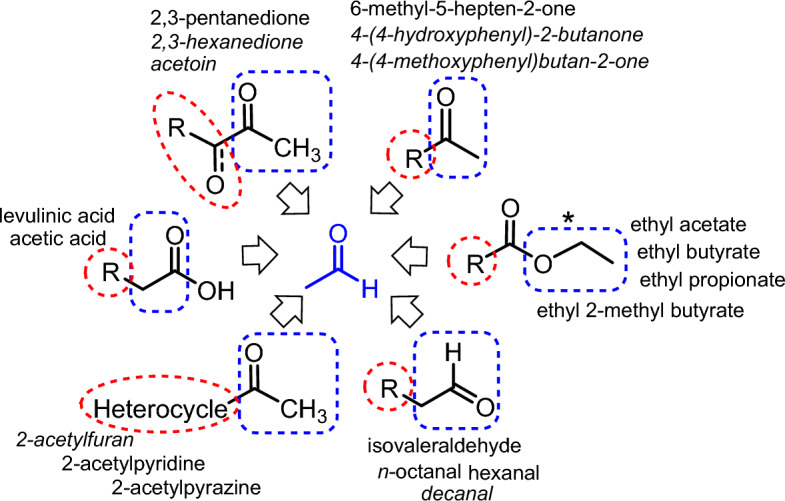
Map showing structural classes of e-liquid flavour chemicals identified as having the potential to produce acetaldehyde (blue box) with a representative selection of co-products (red circle) and the names of the chemical flavours from which they could be produced (see Supplementary Dataset S6). *For ethyl esters the prediction of CH_3_CH_2_O˙ (blue box) indicates the first step in formation of acetaldehyde^47^.

To date, experimental studies on the thermal decomposition products from vaping flavours have focused on detecting and quantifying volatile carbonyls (VC) as they have known negative health implications^55–60^. Several research teams have conclusively shown that VCs such as formaldehyde, acetalaldehyde and propanaldehyde are produced in concerning quantities as a result of the vaping decomposition of flavours. The quantity of aldehydes produced is proportional to the specific commercial brands, flavour and nicotine quantity in the e-liquid, the vaping device power, and users’ puff topography^61–64^. Establishing which flavours produced which VCs is challenging, as the e-liquids tested were comprised of mixtures of several flavour chemicals, propane-1,2-diol, propane-1,2,3-triol (which also produce aldehydes) and nicotine. Mining our dataset allows mapping of VC producers back to specific flavour chemicals while also identifying the other chemicals co-produced with these VCs. For example, the results for acetaldehyde revealed over forty flavours as having the potential to produce it with co-products including heterocycles, aromatics, aldehydes, alkenes and alkanes (Fig. 9, Supplementary Dataset S6). Sources were mostly fruit, candy and dessert flavoured products containing ester, ketone, di-ketone, aldehyde and carboxylic acid functional groups. Cross-referencing this list with the most commonly used fruit and candy flavours^4–6^ implicates ethyl acetate, ethyl butyrate, ethyl 2-methylbutryate and 2,3-pentanedione as the more common sources of pyrolysis produced acetaldehyde. The rapid identification of chemicals of concern is an advantageous feature of this dataset which could be of assistance in focusing experimental work to confirm their generation which could in turn inform regulatory agencies.

Reflecting on the limitations of this study, it is important to identify areas that warrant further development. In this initial iteration of our AI-vape forecast, only the first phase of pyrolysis products has been explored. It would be expected that some pyrolysis products would themselves undergo further pyrolysis reactions and that intermolecular reactions between pyrolysis products could occur^65^. The framework we have put in place lends itself to building a second layer of prediction using the predicted products described in this work as new starting points. Generation of combustion products has not been included in this study as they are considered minor to pyrolysis products, though ambient oxygen reactions could be investigated by inclusion of O_2_ as a reagent in the NN predictions^66^. The cross correlation of predicted pyrolysis products with experimental MS fragmentation data does not distinguish between different structural isomers with the same molecular weights, though in the future more elaborate MS experiments may do so if needed. A limitation of the NN-predictions is the current size of the training sets, though it is anticipated that these will continue to grow in the near future. Additionally, improvements in NN ranking of reaction predictions could be guided by predicted AEs acting as an informative feedback loop (Fig. 2, dashed arrows). A merit of the scientific approach adopted in this work is its openness to continual refinement as chemistry related NNs evolve and that it can serve as a benchmark for other AI methods to achieve similar aims. We hope that this work motivates further research in these areas.

## Conclusion

The aerosols produced by e-cigarette vaping contain immensely complex uncharacterized mixtures of pyrolysis products, the health implications of which are, as yet, mostly unidentified. In advance of health effects of vaping becoming apparent in the general population, AI can be exploited to give guidance to the public, policy makers and health care professionals. It was envisaged that this could be achieved through a strategy that utilizes a combination of innovative NN prediction of pyrolysis transformations and freely accessible experimental EI-MS data to construct an in silico dataset of pyrolysis products from e-liquid chemical flavours. Screening of predicted pyrolysis products against databases of chemical hazard classifications identified those of highest health risk, allowing individual flavour risk profiles to be constructed. Results show that relatively low molecular weight volatile compounds can be produced of which 24% are categorized as either acute toxic or health hazard using the GHS classification system. Collated flavour risk reports may act as an informative public health resource and assist experimental vaping research. Results show that while similarities do exist with conventional tobacco smoking, a significantly different profile of hazardous compounds emerges from vaping. As such, using tobacco smoking as the sole comparison for gauging vaping health risks is likely to give a false sense of security, especially for younger non-tobacco smokers. Regulations could be employed such that attempts to remedy nicotine addictions of older tobacco smokers does not risk the transferal of new health issues to younger generations. A protective balance needs to be struck for both cohorts rather than pitching one against the other. AI methods appear ideally suited to address the complex and multifaceted health concerns that vaping raises. As vaping is a new and unprecedented stress to the human body, with the ability to generate pyrolysis products more toxic that their parent compounds, it seems prudent to strictly limit the number of chemical entities in e-liquids.

## Methods

### Diversity analysis of flavours and WL-NN predicted pyrolysis products

Chemical diversity analysis was carried out using PUMA 1.0 (http://132.248.103.152:3838/PUMA/)^24^. The SMILES structure of 180 flavours and 4524 NN predicted products (duplicate predicted products from the same flavour removed) were used as inputs. This platform computed six molecular properties of pharmaceutical relevance including molecular weight (MW), hydrogen bond donors (nHBDon), hydrogen bond acceptors (nHBAcc), topological polar surface area (TopoPSA), number of rotatable bonds (nRotB), and the octanol–water partition coefficient (ALogP). Then six principal components (PCs) were computed based on these molecular properties. The 3D representation of the chemical space was plotted by using Veusz 3.3.1 software using the three PCs that contributed the most proportion of the variance.

### WL–NN pyrolysis reaction predictions

Supervised learning of Weisfeiler–Lehman network was achieved utilizing the US patent literature as a source of data. The starting dataset which consists of 409,035 reactions is available at: https://github.com/connorcoley/rexgen_direct/tree/master/rexgen_direct/data/^26^. All reactions within the training data that included a flavour molecule were removed to prevent the trained W–L network from data leakage of the test set. In total, a training set of 354,937 reactions was used, on which the pyrolysis predictions were based. The Python script to remove flavour molecules from the original dataset is available at https://github.com/IBM/pyrolysis-prediction. SMILES of the 180 flavour chemicals were used as inputs for the W–L NN using the published protocols. The computational training and prediction were run using a machine with eight CPU cores (Intel Xeon E5-2690 at 2.60 GHz), one GPU (Tesla V100) and 60 GB memory. The number of iterations to train the W–L network and W-LDN was set to 140,000 mini-batches of size 20 and 1,000,000 mini-batches of a single reaction and its candidate outcomes, respectively. Accuracy of prediction tasks was determined using 40,000 test examples^26^ (not in the training set) which gave 0.924 for the model to identify reaction mode when the top 25 predictions are considered and 0.9341 for ranking reactions when the top 5 predictions are considered. The total training time was 2.5 days to train both models (reaction centre and ranking) for reaction prediction. Results from 4500 pyrolysis predictions for 180 flavours gave 7307 products of which 4524 were discrete products (when duplicate products from the same flavour are not included). Average reaction prediction times were 40 ms per reaction for reaction core identification and 127 ms per reaction for ranking.

### Experimental EI-MS data retrieval

Using specifically written script available at https://github.com/IBM/pyrolysis-prediction, the SMILES representations for each flavour were converted to their corresponding InChIKey and the EI-mass spectra data associated with each InChIKey was extracted from the online NIST database at https://webbook.nist.gov/chemistry/^31^. EI-mass data including fragmentation molecular weights and relative abundance were retrieved in JCAMP format. The data for each flavour was checked manually to ensure that the correct data had been acquired for each flavour and errors corrected. Data for some flavours (2-ethyl-3-methyl pyrazine, 2-methoxy-3-methylpyrazine, acetoin, α-damascone, benzaldehyde propylene glycol acetal, benzyl alcohol, β-damascenone, cedrol, citral, ethyl lactate, ethyl vanillin propylene glycol acetal, γ-dodecalactone, γ-octalactone, menthone, neral, propenyl guaethol, tabanone, thio-menthone, trans-2-hexenylacetate, vanillin propylene glycol acetal) were either not available in the NIST database or not accessible and data was manually extracted from the NIST or from Spectrabase (https://spectrabase.com/).

### Correlation of EI-MS fragmentation molecular weights with W–L NN predicted products

Using specifically written script available at https://github.com/IBM/pyrolysis-prediction, the molecular weight of each W–L NN predicted product was calculated using the Descriptors.ExactMolWt method in RDKit. The value of 1 was subtracted from that weight, followed by a rounding to the nearest whole number. NN predictions with the same molecular weight as the flavour molecular ion were not included. The values obtained for each product were correlated with the EI-mass spectrum fragmentation mass data for the relevant flavour molecule available in JCAMP format. Correlation results identified 1169 discrete matches between NN predicted products with EI-MS fragmentations.

### GHS classification data retrieval and cramer classifications

Using specifically written script available at https://github.com/IBM/pyrolysis-prediction, a NN/MS matched product represented as SMILES was converted to its InChIKey and all compounds matching the InChIKey in the PubChem database (https://pubchem.ncbi.nlm.nih.gov/) were retrieved. For each compound identifier, the script retrieved the hazard keywords in the pictographs that appeared in the GHS Classification subsection. Specific inhalation hazards were searched and compiled manually. Cramer classifications were obtained by using compound SMILES inputs into the available prediction software^36^. Results of GHS classifications identified 127 NN/MS matched product predictions as acute toxic, 153 as health hazard, 225 as irritant, 566 as neither acute, health hazard nor irritant and 95 were not identified in the database.

### Mapping reactions for activation energy predictions

The SMILES of reactant and NN-predicted products were used as inputs for the automated mapping algorithm available at http://mapper.grzybowskigroup.pl/marvinjs/^67^. The full atoms reaction maps were completed by using the “map the drawing” command after adding explicit hydrogen atoms.

### D-MPNN pyrolysis activation energy predictions

The original training dataset for activation energy prediction consisting of 16,365 reactions is available at https://zenodo.org/record/3715478#.Yich5BPP2Wj^37,38^. To prevent from data leakage of the test set, reactions involving the 180 flavour chemicals were removed using specific Python script available at https://github.com/IBM/pyrolysis-prediction. The resulting dataset consisted of default hyper-parameters and the b97d3 theory data consisting of 16,264 reactions. Accuracy for AE predictions was determined by performing a tenfold cross validation to train the model with the data split into 85% training, 5% validation and 10% test data^38^. The performance/accuracy metric for the AE is the rooted mean square error defined as $\sqrt {\frac{1}{N}\backslash_{i = 1}^{N} \left( {y_{i} - z_{i} } \right)^{2} }$ where N is the number of examples, *y*_*i*_ is an AE value in the i-th example, and *z*_*i*_ is a predicted AE value in the i-the example. The average accuracy for AE prediction was determined as the root mean square error (RMSE) which was 7.53 mol^−1^ with standard deviation of 0.74 mol^−1^. The average AE value calculated by the 10 models was used to predict AE for pyrolysis with standard deviations included.

### Supplementary Information

Supplementary Information.

Supplementary Information.

Supplementary Information.

Supplementary Information.

Supplementary Information.

Supplementary Information.

Supplementary Information.

## Data Availability

All data are available in the main text, Supplementary Information or GitHub (https://github.com/IBM/pyrolysis-prediction). Raw data files are available from the corresponding author upon request.
