# Supplementary material for: Forecasting vaping health risks through neural network model prediction of flavour pyrolysis reactions
Source: Sci Rep. 2024 May 8;14:9591. doi: 10.1038/s41598-024-59619-x (PMC11079048; doi:10.1038/s41598-024-59619-x)
Supplement: Supplementary file 1 — Supplementary Information. [file 41598_2024_59619_MOESM1_ESM.pdf]

Supplementary Information for

Forecasting Vaping Health Risks through Neural Network Model Prediction of Flavour Pyrolysis Reactions

Akihiro Kishimoto,[a] Dan Wu,[b,1] Donal F. O'Shea[b,1]

[a] IBM Research - Tokyo, Shin-Kawasaki, Japan.

[b] Department of Chemistry, Royal College of Surgeons in Ireland (RCSI), Dublin 2, Ireland.

Contents

Supplementary Table S1. S2

Legends for Supplementary Datasets S1 to S6. S13

Description of AI models used S14

Other supplementary materials for this manuscript include the following:

Supplementary Datasets S1 to S6.

Table S1. Names and Structures of e-Liquid Flavours

Flavour number	Flavour name	Flavour SMILES	Flavour structure
1	2,3-Pentanedione	CCC(=O)C(=O)C	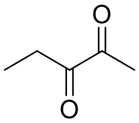
2	1-Pentanol	CCCCCO	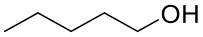
3	1,4-Dimethoxybenzene	COC1=CC=C(C=C1)OC	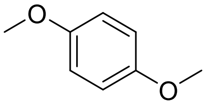
4	2,3-Hexanedione	CC(C(CCC)=O)=O	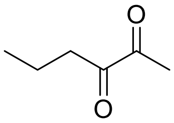
5	2-Acetyl furan	CC(=O)C1=CC=CO1	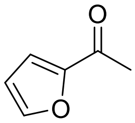
6	2-Acetyl pyridine	CC(=O)C1=CC=CC=N1	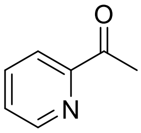
7	2-Acetylpyrazine	CC(=O)C1=NC=CN=C1	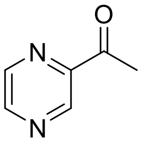
8	2-Ethyl-3-methyl pyrazine	CCC1=NC=CN=C1C	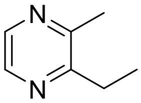
9	2-Isopropyl-4-methyl thiazole	CC1=CSC(=N1)C(C)C	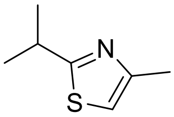
10	2,3-Butanedione	CC(=O)C(=O)C	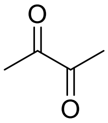
11	2-Methoxy-3-methyl pyrazine	CC1=NC=CN=C1OC	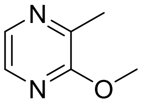
12	2-Methyl butyric acid	CCC(C)C(=O)O	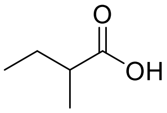
13	2-Methylbutyl acetate	CCC(C)COC(=O)C	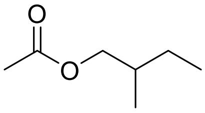
14	2-Phenylethanol	C1=CC=C(C=C1)CCO	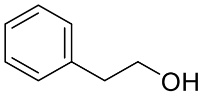

15	2-Phenylethyl acetate	CC(=O)OCCC1=CC=CC=C1	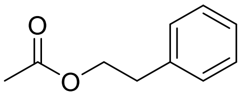
16	2-Propanol	CC(C)O	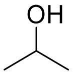
17	2,3-Dimethylpyrazine	CC1=NC=CN=C1C	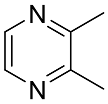
18	2,3,5-Trimethylpyrazine	CC1=CN=C(C(=N1)C)C	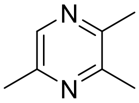
19	2,3,5,6-Tetramethylpyrazine	CC1=C(N=C(C(=N1)C)C)C	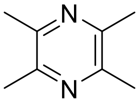
20	2,5-Dimethylpyrazine	CC1=CN=C(C=N1)C	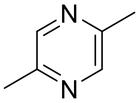
21	2,6-Dimethyl-5-heptenal	CC(CCC=C(C)C)C=O	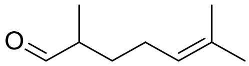
22	4-(4-Hydroxyphenyl)-2-butanone	CC(=O)CCC1=CC=C(C=C1)O	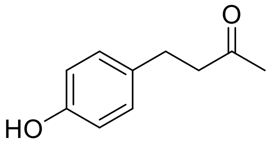
23	4-(4-methoxyphenyl) butan-2-one	CC(=O)CCC1=CC=C(C=C1)OC	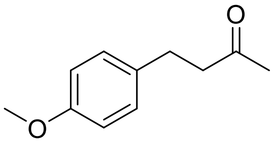
24	4-Methyl-5-thiazole ethanol	CC1=C(SC=N1)CCO	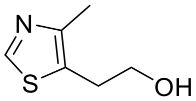
25	4-Terpinenol	CC1=CCC(CC1)(C(C)C)O	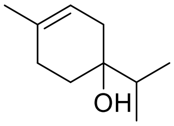
26	4,5-Dimethyl-3-hydroxy-2,5-dihydrofuran-2-one	CC1C(=C(C(=O)O1)O)C	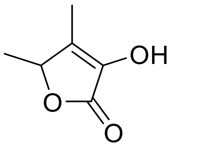
27	5-Methyl-2-phenyl-2-hexenal	CC(C)C/C=C(/C=O)\C1=CC=CC=C1	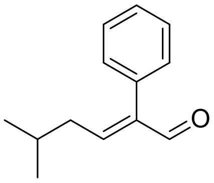
28	5-Methyl furfural	CC1=CC=C(O1)C=O	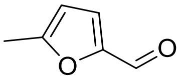
29	6-Methyl-5-hepten-2-one	CC(=CCCC(=O)C)C	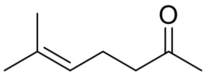
30	6-Methyl coumarin	CC1=CC2=C(C=C1)OC(=O)C=C2	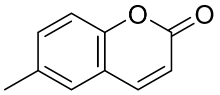

31	Veratraldehyde	COC1=C(C=C(C=C1)C=O)OC	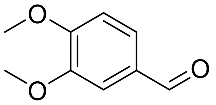
32	Vanillin propylene glycol acetal	CC1COC(O1)C2=CC(=C(C=C2)O)OC	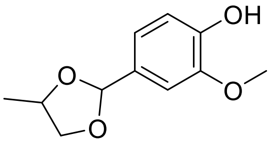
33	Acetic acid	CC(=O)O	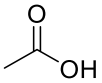
34	Acetoin	CC(C(=O)C)O	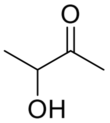
35	Allyl hexanoate	CCCCCC(=O)OCC=C	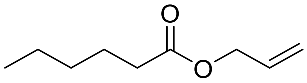
36	*alpha*-Damascenone	C/C=C/C(=O)C1C(=CC=CC1(C)C)C	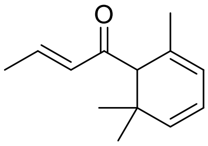
37	*alpha*-Damascone	C/C=C/C(=O)C1C(=CCCC1(C)C)C	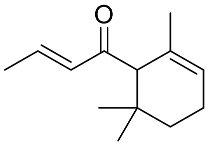
38	*alpha*-Ionone	CC1=CCCC(C1/C=C/C(=O)C)(C)C	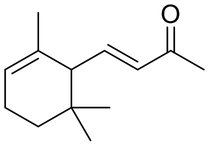
39	*alpha*-Methylbenzyl acetate	CC(C1=CC=CC=C1)OC(=O)C	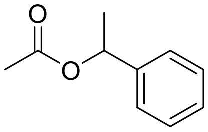
40	*alpha*-Pinene	CC1=CCC2CC1C2(C)C	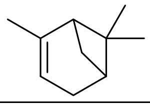
41	*alpha*-Terpineol	CC1=CCC(CC1)C(C)(C)O	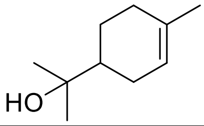
42	Amyl acetate	CCCCCOC(=O)C	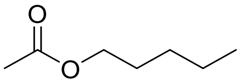
43	Amyl Butyrate	CCCCCOC(=O)CCC	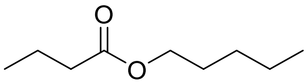
44	Anethole	C/C=C/C1=CC=C(C=C1)OC	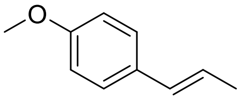
45	Anisaldehyde	COC1=CC=C(C=C1)C=O	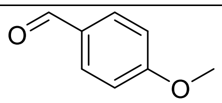

46	Anisyl acetate	CC(=O)OCC1=CC=C(C=C1)OC	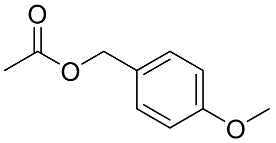
47	Anisyl alcohol	COC1=CC=C(C=C1)CO	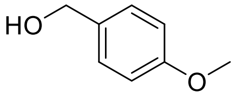
48	Benzaldehyde	C1=CC=C(C=C1)C=O	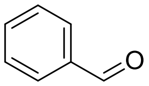
49	Benzaldehyde propylene glycol acetal	CC1COC(O1)C2=CC=CC=C2	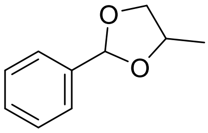
50	Benzyl acetate	CC(=O)OCC1=CC=CC=C1	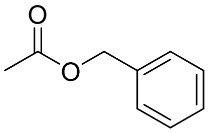
51	Benzyl alcohol	C1=CC=C(C=C1)CO	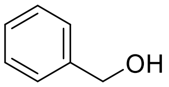
52	Benzyl benzoate	C1=CC=C(C=C1)COC(=O)C2=CC=CC=C2	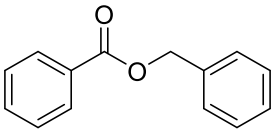
53	Benzyl butyrate	CCCC(=O)OCC1=CC=CC=C1	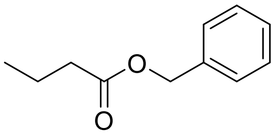
54	*beta*-Caryophyllene	C/C/1=C\CCC(=C)[C@H]2CC([C@@H]2CC1)(C)C	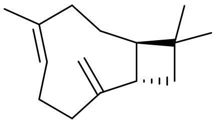
55	*beta*-Damascenone	C/C=C/C(=O)C1=C(C=CCC1(C)C)C	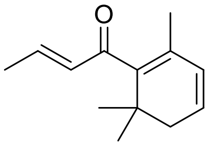
56	*beta*-Damascone	CC=CC(=O)C1=C(CCCC1(C)C)C	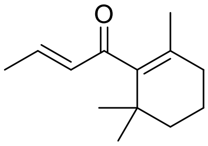
57	*beta*-Ionone	CC1=C(C(CCC1)(C)C)/C=C/C(=O)C	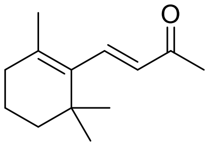
58	*beta*-Pinene	CC1(C2CCC(=C)C1C2)C	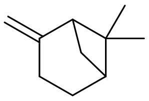
59	Butyl acetate	CCCCOC(=O)C	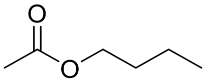
60	Butyl butyrate	CCCCOC(=O)CCC	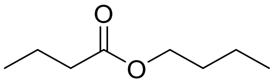

61	Butyl butyryl lactate	CCCCOC(=O)C(C)OC(=O)CCC	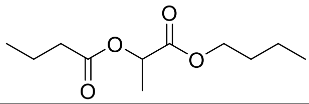
62	Butyric acid	CCCC(=O)O	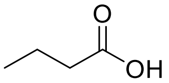
63	Vanillin	COC1=C(C=CC(=C1)C=O)O	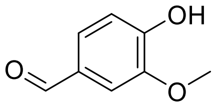
64	Carvone	CC1=CCC(CC1=O)C(=C)C	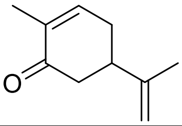
65	Cassia oil-B	COC1=C(C=CC(=C1)CC=C)O	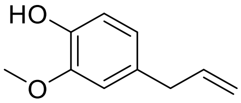
66	Cedrol	C[C@@H]1CC[C@@H]2[C@]13C C[C@@]([C@H](C3)C2(C)C)(C)O	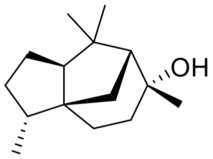
67	Cinnamaldehyde	C1=CC=C(C=C1)/C=C/C=O	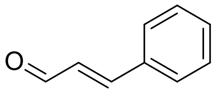
68	Cinnamyl alcohol	C1=CC=C(C=C1)/C=C/CO	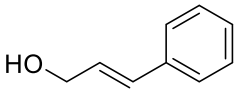
69	*cis*-3-Hexenol	CC/C=C\CCO	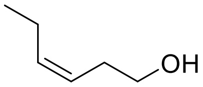
70	*cis*-3-Hexenyl acetate	CC/C=C\CCOC(=O)C	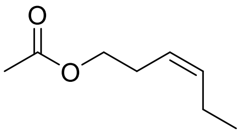
71	*cis*-3-Hexenyl butyrate	CCCC(=O)OCC/C=C\CC	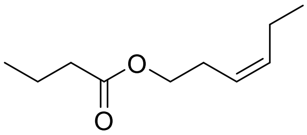
72	*cis*-3-Hexenyl isovalerate	CC/C=C\CCOC(=O)CC(C)C	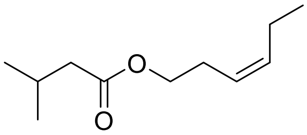
73	*cis*-6-Nonen-1-ol	CC/C=C\CCCCCO	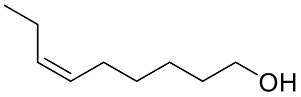
74	Citral	CC(=CCC/C(=C/C=O)/C)C	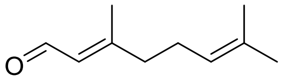
75	Citric acid	C(C(=O)O)C(CC(=O)O)(C(=O)O)O	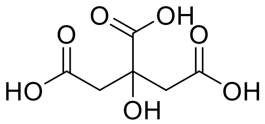
76	Citronellol	CC(CCC=C(C)C)CCO	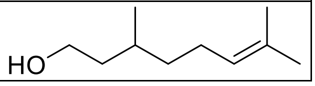

77	Citronellyl acetate	CC(CCC=C(C)C)CCOC(=O)C	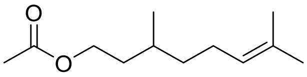
78	D-Carvone	CC1=CC[C@@H](CC1=O)C(=C)C	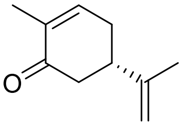
79	Decanal	CCCCCCCCCC=O	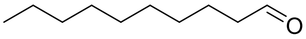
80	Decanoic acid	CCCCCCCCCC(=O)O	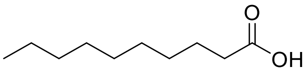
81	*delta*-Decalactone	CCCCCC1CCCC(=O)O1	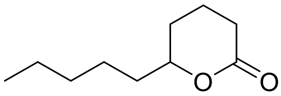
82	*delta*-Dodecalactone	CCCCCCCC1CCCC(=O)O1	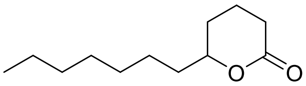
83	Dihydrocoumarin	C1CC(=O)OC2=CC=CC=C21	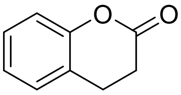
84	Dimethyl anthranilate	CNC1=CC=CC=C1C(=O)OC	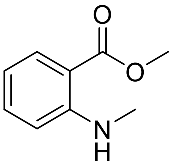
85	Cassia oil-A	C/C=C/C1=CC=CC=C1	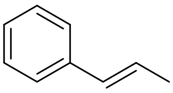
86	Dodecane	CCCCCCCCCCCC	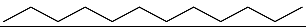
87	Triethyl citrate	CCOC(=O)CC(CC(=O)OCC)(C(=O<br>)OCC)O	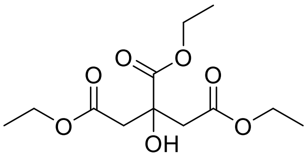
88	Ethyl-3-hydroxy butyrate	CC(O)CC(OCC)=O	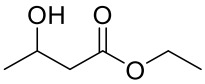
89	Ethyl 2-methylbutyrate	CCC(C)C(=O)OCC	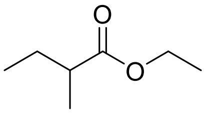
90	Ethyl 2-phenyl acetate	O=C(OCC)CC1=CC=CC=C1	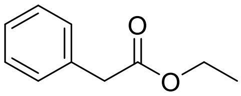
91	Ethyl acetate	CCOC(=O)C	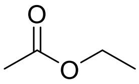
92	Ethyl acetoacetate	CCOC(=O)CC(=O)C	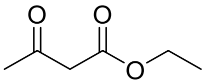
93	Ethyl butyrate	CCCC(=O)OCC	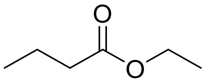
94	Ethyl cinnamate	CCOC(=O)/C=C/C1=CC=CC=C1	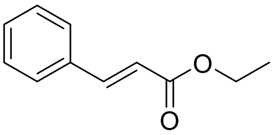
95	Ethyl decanoate	CCCCCCCCCC(=O)OCC	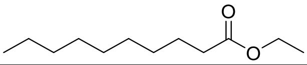

96	Ethyl dodecanoate	CCCCCCCCCCCC(=O)OCC	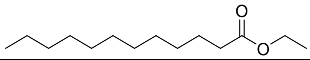
97	Ethyl heptanoate	CCCCCCC(=O)OCC	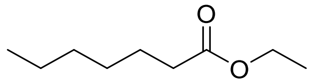
98	Ethyl hexanoate	CCCCCC(=O)OCC	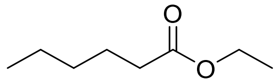
99	Ethyl isovalerate	CCOC(=O)CC(C)C	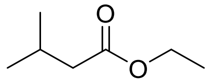
100	Ethyl lactate	CCOC(=O)C(C)O	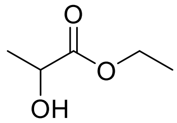
101	Ethyl maltol	CCC1=C(C(=O)C=CO1)O	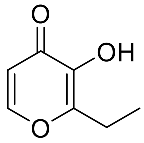
102	*trans*-2-Hexenylacetate	CCC/C=C/COC(C)=O	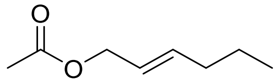
103	Ethyl methyl phenylglycidate	O=C(C1OC1(C)C2=CC=CC=C2)OCC	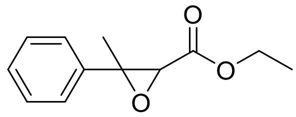
104	Ethyl nonanoate	CCCCCCCCC(=O)OCC	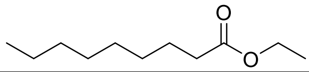
105	Ethyl octanoate	CCCCCCCC(=O)OCC	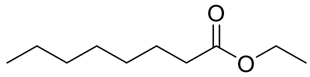
106	Ethyl propionate	CCC(=O)OCC	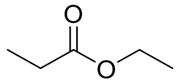
107	Ethyl vanillin	CCOC1=C(C=CC(=C1)C=O)O	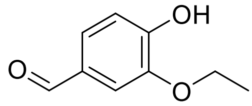
108	Ethyl vanillin propylene glycol acetal	CCOC1=C(C=CC(=C1)C2OCC(O2)C)O	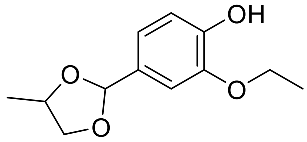
109	Eucalyptol	CC1(C2CCC(O1)(CC2)C)C	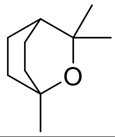
110	*trans*-2-Hexenol	CCC/C=C/CO	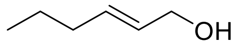
111	Furaneol	CC1C(=O)C(=C(O1)C)O	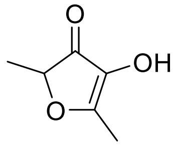
112	Furfural	C1=COC(=C1)C=O	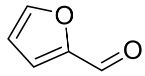
113	Furfuryl alcohol	C1=COC(=C1)CO	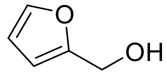

114	*gamma*-Decalactone	CCCCCCC1CCC(=O)O1	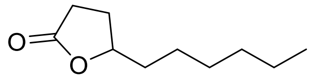
115	*gamma*-Dodecalactone	CCCCCCCCC1CCC(=O)O1	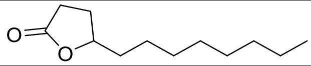
116	*gamma*-Hexalactone	CCC1CCC(=O)O1	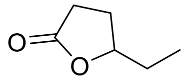
117	*gamma*-Nonalactone	CCCCCC1CCC(=O)O1	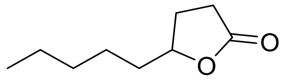
118	*gamma*-Octalactone	CCCCC1CCC(=O)O1	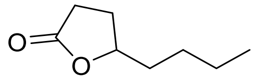
119	*gamma*-Terpinene	CC1=CCC(=CC1)C(C)C	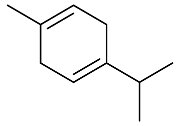
120	*gamma*-Undecalactone	CCCCCCCC1CCC(=O)O1	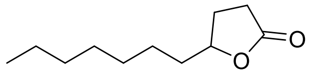
121	*gamma*-Valerolactone	CC1CCC(=O)O1	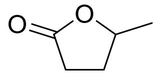
122	*trans*-2-Hexenoic acid	CCC/C=C/C(=O)O	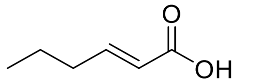
123	Geraniol	CC(=CCC/C(=C/CO)/C)C	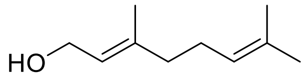
124	Geranyl acetate	CC(=CCC/C(=C/COC(=O)C)/C)C	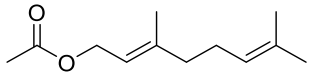
125	*trans*-2-Hexenal	CCC/C=C/C=O	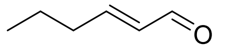
126	Thio menthone	CC(C1)CCC(C(C)(S)C)C1=O	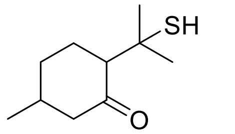
127	Guaiacol	COC1=CC=CC=C1O	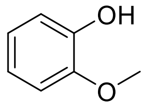
128	Hexanal	CCCCCC=O	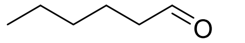
129	Hexanoic acid	CCCCCC(=O)O	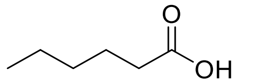
130	Hexyl acetate	CCCCCCOC(=O)C	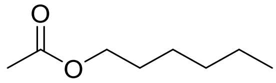
131	Hexyl butyrate	CCCCCCOC(=O)CCC	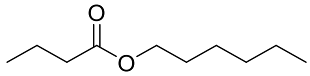
132	Ionone	O=C(/C=C/C1C(C)=CCCC1(C)C)C	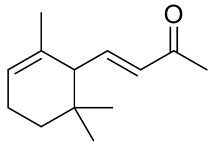
133	Isoamyl acetate	CC(C)CCOC(=O)C	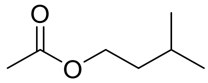
134	Isoamyl alcohol	CC(C)CCO	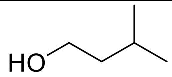

135	Isoamyl butyrate	CCCC(=O)OCCC(C)C	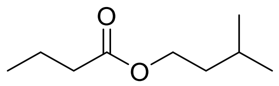
136	Isoamyl isovalerate	CC(C)CCOC(=O)CC(C)C	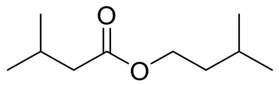
137	Isoamyl phenyl acetate	CC(C)CCOC(=O)CC1=CC=CC=C 1	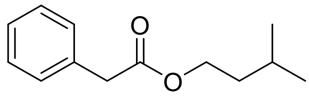
138	Isobutyl acetate	CC(C)COC(=O)C	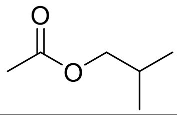
139	Isobutyl alcohol	CC(C)CO	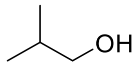
140	Isobutyl butyrate	CCCC(=O)OCC(C)C	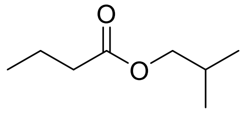
141	Isobutyric acid	CC(C)C(=O)O	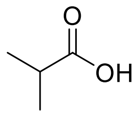
142	Isovaleraldehyde	CC(C)CC=O	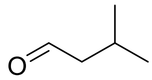
143	*L*-Carvone	CC1=CC[C@H](CC1=O)C(=C)C	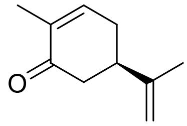
144	Lactic acid	CC(C(=O)O)O	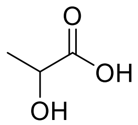
145	Levulinic acid	CC(=O)CCC(=O)O	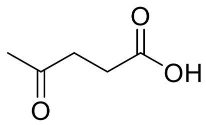
146	Limonene	CC1=CCC(CC1)C(=C)C	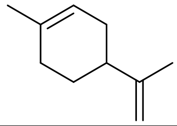
147	Linalool	CC(=CCCC(C)(C=C)O)C	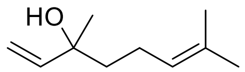
148	Linalyl acetate	CC(=CCCC(C)(C=C)OC(=O)C)C	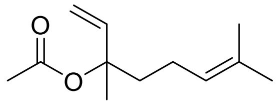
149	Maltol	CC1=C(C(=O)C=CO1)O	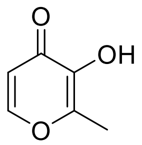
150	Menthol	CC1CCC(C(C1)O)C(C)C	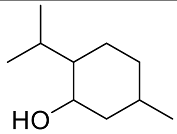

151	Menthone	CC1CCC(C(=O)C1)C(C)C	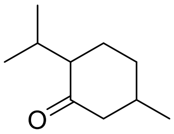
152	Menthyl acetate	CC1CCC(C(C1)OC(=O)C)C(C)C	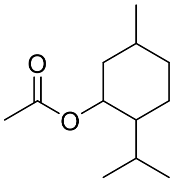
153	Methyl-alpha-ionone	CCC(=O)/C=C/C1C(=CCCC1(C)C)C	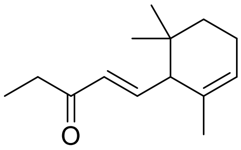
154	Methyl anthranilate	COC(=O)C1=CC=CC=C1N	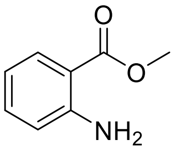
155	Methyl cinnamate	COC(=O)/C=C/C1=CC=CC=C1	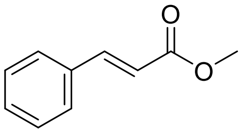
156	Methyl cyclopentenolone	CC1=C(C(=O)CC1)O	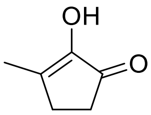
157	Methyl dihydrojasmonate	CCCCCC1C(CCC1=O)CC(=O)OC	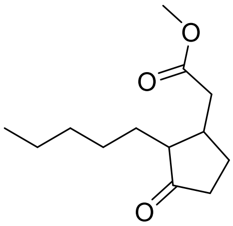
158	Methyl salicylate	COC(=O)C1=CC=CC=C1O	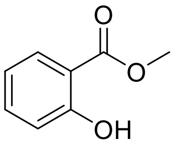
159	Methyl thiobutyrate	CCCC(OC)=S	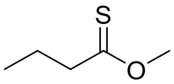
160	Terpinolene	CC1=CCC(=C(C)C)CC1	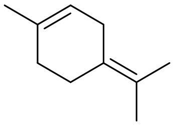
161	Methyl-thio-methylpyrazine	CSC1=NC=CN=C1C	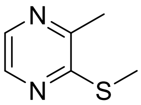
162	Myrcene	CC(=CCCC(=C)C=C)C	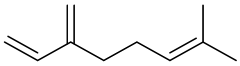
163	n-Butanol	CCCCO	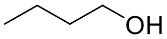
164	n-Hexanol	CCCCCCO	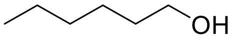
165	n-Octanal	CCCCCCCC=O	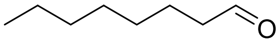
166	n-Propanol	CCCO	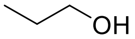

167	Neral	CC(=CCC/C(=C\C=O)/C)C	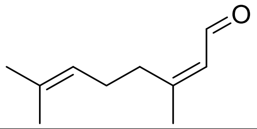
168	Nerol	CC(=CCC/C(=C\CO)/C)C	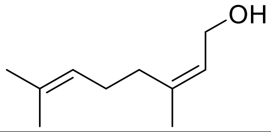
169	Neryl acetate	CC(=CCC/C(=C\COC(=O)C)/C)C	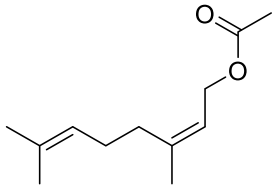
170	Tabanone	CC=CC=C1C(=CC(=O)CC1(C)C)C	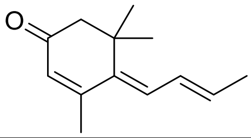
171	Octanoic acid	CCCCCCCC(=O)O	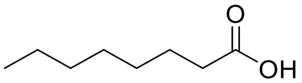
172	Octanol	CCCCCCCCO	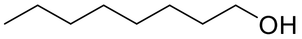
173	Oleic acid	CCCCCCCC/C=C\CCCCCCCC(=O)O	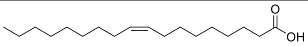
174	p-Cymene	CC1=CC=C(C=C1)C(C)C	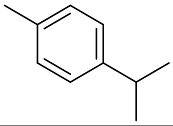
175	Piperonal	C1OC2=C(O1)C=C(C=C2)C=O	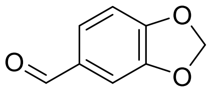
176	Sorbic acid	CC=CC=CC(=O)O	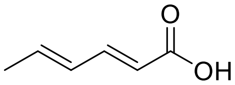
177	Propenyl guaethol	CCOC1=CC=C(/C=C/C)C=C1O	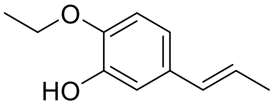
178	Propionic acid	CCC(=O)O	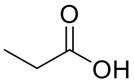
179	Propyl acetate	CCCOC(=O)C	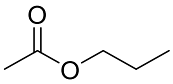
180	Benzoic acid	C1=CC=C(C=C1)C(=O)O	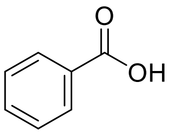

Legends for Supplementary Datasets S1 to S6

Supplementary Dataset S1 (separate file).

Diversity analysis of 180 flavours and WL-NN predicted pyrolysis products.

Supplementary Dataset S2 (separate file).

e-Liquid Reports: WL-NN pyrolysis reaction predictions, EI-MS matched products, GHS classifications for each e-liquid flavour.

Supplementary Dataset S3 (separate file).

GHS Classifications of NN/MS matched predicted products for each e-liquid flavour.

Supplementary Dataset S4 (separate file).

Cramer classifications for NN/MS matched pyrolysis predictions.

Supplementary Dataset S5 (separate file).

D-MPNN pyrolysis activation energy prediction data.

Supplementary Dataset S6 (separate file).

Mapping predicted acetaldehyde-producing flavours.

1. Architecture of pyrolysis prediction model

The architecture of the pyrolysis prediction model used was identical to that of Coley *et al.* (see the supplementary material of reference 1 for details).[1,2]

1.1. Reaction Core Identification

Let u and v be atoms, (u, v) be a bond connecting from atom u to atom v, and $N(v)$ be a set of atoms adjacent to v. Let M, P, U, V, and W be matrices representing model parameters updated during training, w^T be a transposed vector of model parameters, $\tau(\cdot)$ be the ReLU activation function, $\sigma(\cdot)$ be the Sigmoid function, and $\mathrm{cat}(\cdot,\cdot)$ be a function that returns a concatenated vector/matrix. The initial six atom features f_v^0 and two bond features b_{vu} for v and (v, u) are represented as vectors and extracted by RDKit.

Then, for each v, a so-called message passing step is performed to iteratively update a hidden state f_v^t, accounting for v as well as atoms connections through their bonds:

$$f_v^{t+1} = \tau(U_1 f_v^t + U_2 \sum_{u \in N(v)} \tau(V_1 f_u^t + V_2 b_{uv}))$$

where t is the number of iterations. The message passing step is performed L times. L is set to 3. The dimension size of the hidden state is set to 300.

The next NN layers calculate a local atom representation c_v that accounts for atoms and bonds accessible to v in L steps:

$$c_v = \mathrm{cat}(\sum_{u \in N(v)} \mathrm{cat}(W_1 f_u^L, W_2 b_{uv}), W_3 f_v^L)$$

The attention layers are designed to calculate a more global atom representation $\widehat{c_v}$, based on an attention score of atom v upon atom z, which allows to account for distant or disconnected atoms:

$$\alpha_{vz} = \sigma(w_a^T \tau(P_a(c_v + c_z) + P_b e_{vz})$$

$$\widehat{c_v} = \sum_z \alpha_{vz} c_z$$

where e_{vz} is a set of features for atom-atom relationships including the bond type between the atoms (if any) and whether they belong to the same molecule.

Let a reaction center $\{(u, v, b)\}$ be a set of changes, where (u, v) is a pair of atoms whose connecting bond has changed to type b. Given the local and global atom representations, the following formula is finally used to predict a likelihood of (u, v, b) being in reaction center:

$$s_{u,v,b} = \sigma(w_b^T \left(M_a(\widehat{c_u} + \widehat{c_v}\right) + P_a(c_u + c_v) + M_b f_{uv}))$$

1.2 Pyrolysis Reaction Ranking

Given a set of reactant molecules r, the goal of evaluating pyrolysis reactions is to learn a scoring function for a list of candidate products $\{p_0, p_1, \cdots, p_m\}$, starting with a difference vector $d_v^{(p_i)}$ between learned atom representations $c_v^{(p_i)}$ and $c_v^{(r)}$ for atom v that has the same mapping id in p_i and r:

$d_v^{(p_i)} = c_v^{(p_i)} - c_v^{(r)}$

Based on this difference vector, a difference graph $D(r, p_i)$ is defined as a molecular graph that has the same structure as p_i but has an atom feature of $d_v^{(p_i)}$ for v.

A message passing step is performed L times for the t-th update of hidden state $h_v^{(p_i,t)}$ for atom v, thus calculating a feature vector $g_v^{(p_i)}$:

$$h_v^{(p_i,t+1)} = \tau(U_1 h_v^{(p_i,t)} + U_2 \sum_{u \in N(v)} \tau(V_1 h_u^{(p_i,t)} + V_2 b_{uv}))$$

$$g_v^{(p_i)} = \text{cat}(\sum_{u \in N(v)} \text{cat}(W_1 h_u^{(p_i,L)}, W_2 b_{uv}), W_3 h_v^{(pi,L)})$$

where $h_v^{(p_i,0)} = d_v^{(p_i)}$, $L = 3$, and W_* are distinct matrices from those for reaction core identification. The dimension size of the hidden state is set to 500.

Let $RC(p_i) = \{(u_i, v_i, b_i)\}$, defined as the set of bonds that changed from the reactant r to product p_i. The final score of candidate p_i is calculated as:

$$s(p_i) = w^T \tau\left(M \sum_{v \in p_i} g_v^{(p^i)}\right) + \sum_{(u,v,b) \in RC(p_i)} s_{u,v,b}$$

2. Architecture of AE model

The architecture of AE model is identical to that of Grambow et al. (see the supplementary material of reference 35 for details). As described in their supplementary material, some NN layers may include bias parameters that are not explicitly shown in the equations here.

Let f_v and b_{vu} be initial eight atom features for v and initial four bond features for bond (v, u), respectively. These initial features are extracted by RDKit. Hydrogens are represented as explicit atoms.

Unlike the pyrolysis prediction model, hidden states are defined for edges. For each bond (v, u), the starting layers yield initial hidden state h_{vu}^0 with dimension size 1800, defined as follows:

$$h_{vu}^0 = \tau(W_1 \text{cat}(f_v, b_{vu}))$$

Then, the following message passing step is performed to iteratively update a hidden state for (v, u) to be able to account for itself as well as its indirect bond connections via connected atoms.

$$h_{vu}^{t+1} = \tau(h_{vu}^0 + W_2 \sum_{k \in N(v) \backslash \{u\}} h_{kv}^t)$$

The message passing step is performed L times and h_{vu}^L is calculated, representing a bond fingerprint for (v, u). L is set to 5.

The next layers calculate an atom fingerprint h_v for each v, accounting for v and its directly connected bonds.

$$h_v = \tau(W_3 \text{cat}\left(f_v, \sum_{u \in N(v)} h_{uv}^L\right))$$

The atom fingerprints are calculated for each v in the reactant and the product, denoted as $h_v^{(p)}$ and $h_v^{(r)}$

The next layers account for a difference between atom fingerprints of the product and the reactant. This difference indicates an influence of each v on reaction.

$$d_v = \tau(W_4(h_v^{(p)} - h_v^{(r)}))$$

Next, 200 molecular features calculated by RDKit are prepared for the product and reactant, denoted by $f^{(p)}$ and $f^{(r)}$. Let G be a set of atoms in the reactant and the product. A vector $\hat{r}$ representing a reaction is obtained by the following equation.

$$\hat{r} = \text{cat}(\sum_{v \in G} d_v, f^{(p)} - f^{(r)})$$

Finally, an estimated AE value is calculated as follows.

$$\hat{E} = w_1^T \hat{r}$$

As an improvement to the model performance, a multi-task learning is performed that secondarily outputs a prediction of the enthalpy $\Delta\hat{H}$ of reaction.

$$\Delta\hat{H} = w_2^T \hat{r}$$

$\Delta\widehat{H}$ is supplied only for training a model and is not used when AE is predicted.

References:

1. C. W. Coley *et al.*, A graph-convolutional neural network model for the prediction of chemical reactivity. *Chem. Sci.*, 2019, **10**, 370.
2. W. Jin, C. Coley, R. Barzilay, T. Jaakkola, Predicting organic reaction outcomes with Weisfeiler-Lehman network. *NeurIPS*, 2017, 2604.
