# Supplementary material for: Forecasting vaping health risks through neural network model prediction of flavour pyrolysis reactions
Source: Sci Rep. 2024 May 8;14:9591. doi: 10.1038/s41598-024-59619-x (PMC11079048; doi:10.1038/s41598-024-59619-x)
Supplement: Supplementary file 3 — Supplementary Information. [file 41598_2024_59619_MOESM3_ESM.pdf]

Appendix Dataset S2

NN-ranked 1 to 25 pyrolysis prediction products for 180 e-liquids flavours.

NN/MS matched products highlighted in bold font, relative abundance of MS peak included in brackets.

GHS classification (acute, health hazard, irritant) included for all NN/MS matched products.

Summary report of GHS Classification (acute, health hazard, irritant) for NN/MS matches were generated in structures.

Table of Contents:

Number	Chemical name	Page
1.	2,3-Pentanedione	1
2.	1-Pentanol	4
3.	1,4-Dimethoxybenzene	7
4.	2,3-Hexanedione	9
5.	2-Acetyl Furan	11
6.	2-Acetyl Pyridine	13
7.	2-Acetylpyrazine	16
8.	2-Ethyl-3-methyl pyrazine	18
9.	2-Isopropyl-4-methyl thiazole	20
10.	2,3-Butanedione	22
11.	2-Methoxy-3-methyl pyrazine	23
12.	2-Methyl Butyric Acid	26
13.	2-Methylbutyl Acetate	28
14.	2-Phenylethanol	31
15.	2-Phenylethyl Acetate	33
16.	2-Propanol	35
17.	2,3-Dimethylpyrazine	38
18.	2,3,5-Trimethylpyrazine	40
19.	2,3,5,6-Tetramethylpyrazine	42
20.	2,5-Dimethylpyrazine	43
21.	2,6-Dimethyl-5-heptenal	44
22.	4-(4-Hydroxyphenyl)-2-butanone	46
23.	4-(4-Methoxyphenyl)butan-2-one	48
24.	4-Methyl-5-Thiazole ethanol	50
25.	4-Terpinenol	52
26.	4,5-Dimethyl-3-Hydroxy-2,5-Dihydrofuran-2-One	54
27.	5-Methyl-2-Phenyl-2-Hexenal	56
28.	5-Methyl Furfural	59
29.	6-Methyl-5-Hepten-2-one	62
30.	6-Methyl Coumarin	64
31.	Veratraldehyde	65

32.	Vanillin Propylene Glycol Acetal	68
33.	Acetic Acid	71
34.	Acetoin	74
35.	Allyl Hexanoate	76
36.	alpha-Damascenone	79
37.	alpha-Damascone	81
38.	Alpha-Ionone	82
39.	alpha-Methylbenzyl acetate	83
40.	alpha-Pinene	85
41.	alpha-Terpineol	87
42.	Amyl acetate	89
43.	Amyl Butyrate	91
44.	Anethole	93
45.	Anisaldehyde	96
46.	Anisyl Acetate	98
47.	Anisyl Alcohol	100
48.	Benzaldehyde	103
49.	Benzaldehyde propylene glycol acetal	106
50.	Benzyl Acetate	109
51.	Benzyl Alcohol	112
52.	Benzyl Benzoate	114
53.	Benzyl Butyrate	117
54.	beta-Caryophyllene	120
55.	beta-Damascenone	122
56.	beta-Damascone	123
57.	Beta-Ionone	125
58.	beta-Pinene	127
59.	Butyl Acetate	128
60.	Butyl Butyrate	131
61.	Butyl Butyryl Lactate	134
62.	Butyric Acid	136
63.	Vanillin	138
64.	Carvone	141
65.	Cassia oil-B	143
66.	Cedrol	145
67.	Cinnamaldehyde	146
68.	Cinnamyl alcohol	149
69.	Cis-3-hexenol	153
70.	Cis-3-hexenyl Acetate	155
71.	Cis-3-Hexenyl Butyrate	157
72.	Cis-3-Hexenyl Isovalerate	159
73.	cis-6-Nonen-1-ol	161
74.	Citral	163
75.	Citric Acid	165
76.	Citronellol	166
77.	Citronellyl Acetate	168
78.	D-Carvone	170
79.	Decanal	172

80.	Decanoic Acid	174
81.	delta-Decalactone	176
82.	delta-Dodecalactone	178
83.	Dihydrocoumarin	179
84.	Dimethyl Anthranilate	181
85.	Cassia oil-A	183
86.	Dodecane	186
87.	Triethyl Citrate	188
88.	Ethyl-3-Hydroxy Butyrate	190
89.	Ethyl 2-Methyl Butyrate	193
90.	Ethyl 2-Phenyl Acetate	196
91.	Ethyl Acetate	198
92.	Ethyl Acetoacetate	201
93.	Ethyl Butyrate	203
94.	Ethyl Cinnamate	207
95.	Ethyl Decanoate	210
96.	Ethyl Dodecanoate	213
97.	Ethyl Heptanoate	216
98.	Ethyl Hexanoate	219
99.	Ethyl Isovalerate	223
100.	Ethyl Lactate	226
101.	Ethyl Maltol	228
102.	trans-2-Hexenylacetate	230
103.	Ethyl Methyl Phenylglycidate	232
104.	Ethyl Nonanoate	235
105.	Ethyl Octanoate	238
106.	Ethyl Propionate	241
107.	Ethyl Vanillin	244
108.	Ethyl Vanillin Propylene Glycol Acetal	246
109.	Eucalyptol	249
110.	trans-2-Hexenol	251
111.	Furaneol	253
112.	Furfural	255
113.	Furfuryl Alcohol	258
114.	gamma-Decalactone	261
115.	gamma-Dodecalactone	262
116.	gamma-Hexalactone	263
117.	gamma-Nonalactone	264
118.	gamma-Octalactone	266
119.	gamma-Terpinene	268
120.	gamma-Undecalactone	270
121.	gamma-Valerolactone	271
122.	trans-2-Hexenoic acid	273
123.	Geraniol	276
124.	Geranyl Acetate	278
125.	trans-2-Hexenal	280
126.	Thio Menthone	283
127.	Guaiacol	285

128.	Hexanal	287
129.	Hexanoic Acid	289
130.	Hexyl Acetate	291
131.	Hexyl Butyrate	293
132.	Ionone (mixed isomers)	295
133.	Isoamyl Acetate	296
134.	Isoamyl Alcohol	299
135.	Isoamyl Butyrate	302
136.	Isoamyl Isovalerate	304
137.	Isoamyl Phenyl Acetate	306
138.	Isobutyl Acetate	308
139.	Isobutyl Alcohol	311
140.	Isobutyl Butyrate	313
141.	Isobutyric Acid	316
142.	Isovaleraldehyde	318
143.	L-Carvone	320
144.	Lactic Acid	322
145.	Levulinic Acid	324
146.	Limonene	326
147.	Linalool	328
148.	Linalyl Acetate	330
149.	Maltol	332
150.	Menthol	334
151.	Menthone	336
152.	Menthyl acetate	338
153.	Methyl-alpha-ionone	340
154.	Methyl Anthranilate	341
155.	Methyl Cinnamate	343
156.	Methyl Cyclopentenolone	345
157.	Methyl Dihydrojasmonate	347
158.	Methyl Salicylate	349
159.	Methyl Thiobutyrate	351
160.	Terpinolene	353
161.	Methyl-thio-methylpyrazine	356
162.	Myrcene	358
163.	n-Butanol	360
164.	n-Hexanol	363
165.	n-Octanal	365
166.	n-Propanol	367
167.	Neral	369
168.	Nerol	371
169.	Neryl Acetate	373
170.	Tabanone	375
171.	Octanoic Acid	377
172.	Octanol	379
173.	Oleic Acid	381
174.	p-Cymene	382
175.	Piperonal	384

176.	Sorbic Acid	387
177.	Propenyl Guaethol	390
178.	Propionic Acid	382
179.	Propyl Acetate	394
180.	Benzoic Acid	397

1. 2,3-Pentanedione: CCC(=O)C(=O)C

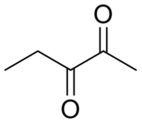

Summary Report of GHS Classification for NN/MS Matches:

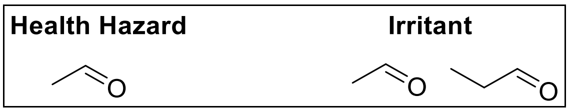

NN-rank 1: ['CCC(O)C(C)=O']

NN-rank 2: ['CC=C(O)C(C)=O']

NN-rank 3: ['CCC(=O)CC', 'O']

NN-rank 4: ['C', 'CC(=O)C(C)=O']

> **EI-MS matched molecular weight (relative abundance: 14.33%) for C**
>
> **GHS hazard classification: none of the hazards selected for correlation**

NN-rank 5: ['CC=CC(C)=O', 'O']

NN-rank 6: ['CC', 'CC(=O)CO']

> **EI-MS matched molecular weight (relative abundance: 60.81%) for CC**
>
> **GHS hazard classification: none of the hazards selected for correlation**

NN-rank 7: ['CCCC(C)=O', 'O']

NN-rank 8: ['CC', 'CC(=O)C=O']

> **EI-MS matched molecular weight (relative abundance: 60.81%) for CC**
>
> **GHS hazard classification: none of the hazards selected for correlation**

NN-rank 9: ['O=C1CCCC1=O']

NN-rank 10: ['C', 'C=C(O)C(C)=O']

> **EI-MS matched molecular weight (relative abundance: 14.33%) for C**
>
> **GHS hazard classification: none of the hazards selected for correlation**

NN-rank 11: ['CC=O', 'CCC=O']

> **EI-MS matched molecular weight (relative abundance: 99.99%) for CC=O**
>
> **GHS hazard classification: Health Hazard, Irritant**
>
> **EI-MS matched molecular weight (relative abundance: 32.85%) for CCC=O**
>
> **GHS hazard classification: Irritant**

NN-rank 12: ['CC=O', 'CCCO']

NN/MS matches corresponding with tox GHS classification highlight in Bold font.

EI-MS matched molecular weight (relative abundance: 99.99%) for CC=O

GHS hazard classification: Health Hazard, Irritant

NN-rank 13: ['CC=C=O', 'CC=O']

EI-MS matched molecular weight (relative abundance: 99.99%) for CC=O

GHS hazard classification: Health Hazard, Irritant

NN-rank 14: ['CCc1cc1=O', 'O']

NN-rank 15: ['C', 'CCC(=O)C=O']

EI-MS matched molecular weight (relative abundance: 14.33%) for C

GHS hazard classification: none of the hazards selected for correlation

NN-rank 16: ['C=CC(=O)C(C)=O']

NN-rank 17: ['CC', 'O=c1cc1O']

EI-MS matched molecular weight (relative abundance: 60.81%) for CC

GHS hazard classification: none of the hazards selected for correlation

NN-rank 18: ['CCC(=O)C(C)=O']

NN-rank 19: ['CCC(=O)C(C)O']

NN-rank 20: ['CC=CO', 'CC=O']

EI-MS matched molecular weight (relative abundance: 32.85%) for CC=CO

GHS hazard classification: none of the hazards selected for correlation

EI-MS matched molecular weight (relative abundance: 99.99%) for CC=O

GHS hazard classification: Health Hazard, Irritant

NN-rank 21: ['C', 'C=CC(C)=O', 'O']

EI-MS matched molecular weight (relative abundance: 14.33%) for C

GHS hazard classification: none of the hazards selected for correlation

NN-rank 22: ['C', 'CCC(C)=O', 'O']

EI-MS matched molecular weight (relative abundance: 14.33%) for C

GHS hazard classification: none of the hazards selected for correlation

NN-rank 23: ['C=O', 'CC', 'CC=O']

EI-MS matched molecular weight (relative abundance: 60.81%) for C=O

GHS hazard classification: Acute Toxic, Health Hazard, Irritant

EI-MS matched molecular weight (relative abundance: 60.81%) for CC

GHS hazard classification: none of the hazards selected for correlation

EI-MS matched molecular weight (relative abundance: 99.99%) for CC=O

GHS hazard classification: Health Hazard, Irritant

NN-rank 24: ['C=CC(=O)CC', 'O']

NN-rank 25: ['CC', 'CC(C)=O', 'O']

NN/MS matches corresponding with tox GHS classification highlight in Bold font.

EI-MS matched molecular weight (relative abundance: 60.81%) for CC

GHS hazard classification: none of the hazards selected for correlation

EI-MS matched molecular weight (relative abundance: 32.85%) for CC(C)=O

GHS hazard classification: Irritant

NN/MS matches corresponding with tox GHS classification highlight in Bold font.

2. 1-Pentanol: CCCCCO

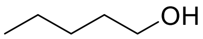

Summary Report of GHS Classification for NN/MS Matches:

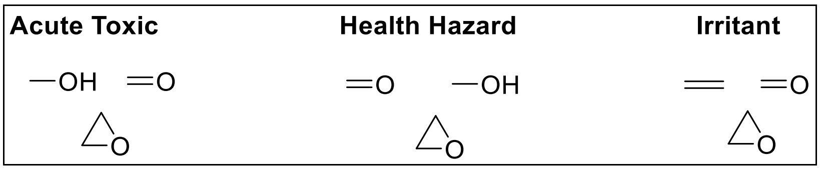

NN-rank 1: ['C#CCCC', 'O']

NN-rank 2: ['C=CCCC', 'O']

EI-MS matched molecular weight (relative abundance: 6.5%) for C=CCCC

GHS hazard classification: none of the hazards selected for correlation

NN-rank 3: ['CCCCC', 'O']

NN-rank 4: ['CCCC', 'CO']

EI-MS matched molecular weight (relative abundance: 25.8%) for CCCC

GHS hazard classification: none of the hazards selected for correlation

EI-MS matched molecular weight (relative abundance: 36.9%) for CO

GHS hazard classification: Acute Toxic, Health Hazard

NN-rank 5: ['CCCCC=O']

NN-rank 6: ['C#C', 'CCC', 'O']

EI-MS matched molecular weight (relative abundance: 27.2%) for CCC

GHS hazard classification: none of the hazards selected for correlation

NN-rank 7: ['C', 'CCCCO']

NN-rank 8: ['CCC', 'CCO']

EI-MS matched molecular weight (relative abundance: 27.2%) for CCC

GHS hazard classification: none of the hazards selected for correlation

EI-MS matched molecular weight (relative abundance: 5.6%) for CCO

GHS hazard classification: none of the hazards selected for correlation

NN-rank 9: ['CCCC#CO']

NN-rank 10: ['CC', 'CCCO']

EI-MS matched molecular weight (relative abundance: 33.0%) for CC

GHS hazard classification: none of the hazards selected for correlation

NN-rank 11: ['C', 'CCCC', 'O']

NN/MS matches corresponding with tox GHS classification highlight in Bold font.

EI-MS matched molecular weight (relative abundance: 25.8%) for CCCC

GHS hazard classification: none of the hazards selected for correlation

NN-rank 12: ['C=C', 'CCC', 'O']

EI-MS matched molecular weight (relative abundance: 19.2%) for C=C

GHS hazard classification: Irritant

EI-MS matched molecular weight (relative abundance: 27.2%) for CCC

GHS hazard classification: none of the hazards selected for correlation

NN-rank 13: ['CCCC(C)O']

NN-rank 14: ['CCCC1CO1']

NN-rank 15: ['C#CO', 'CCC']

EI-MS matched molecular weight (relative abundance: 68.99%) for C#CO

GHS hazard classification: none of the hazards selected for correlation

EI-MS matched molecular weight (relative abundance: 27.2%) for CCC

GHS hazard classification: none of the hazards selected for correlation

NN-rank 16: ['CCC', 'CCO']

EI-MS matched molecular weight (relative abundance: 27.2%) for CCC

GHS hazard classification: none of the hazards selected for correlation

EI-MS matched molecular weight (relative abundance: 5.6%) for CCO

GHS hazard classification: none of the hazards selected for correlation

NN-rank 17: ['CCCC=CO']

NN-rank 18: ['C1CCC1', 'CO']

EI-MS matched molecular weight (relative abundance: 94.99%) for C1CCC1

GHS hazard classification: none of the hazards selected for correlation

EI-MS matched molecular weight (relative abundance: 36.9%) for CO

GHS hazard classification: Acute Toxic, Health Hazard

NN-rank 19: ['C=O', 'CCCC']

EI-MS matched molecular weight (relative abundance: 33.0%) for C=O

GHS hazard classification: Acute Toxic, Health Hazard, Irritant

EI-MS matched molecular weight (relative abundance: 25.8%) for CCCC

GHS hazard classification: none of the hazards selected for correlation

NN-rank 20: ['C1CCOCC1']

NN-rank 21: ['CCCCCO']

NN-rank 22: ['OCC1CCC1']

NN-rank 23: ['C1CO1', 'CCC']

EI-MS matched molecular weight (relative abundance: 27.2%) for C1CO1

NN/MS matches corresponding with tox GHS classification highlight in Bold font.

GHS hazard classification: Acute Toxic, Health Hazard, Irritant

EI-MS matched molecular weight (relative abundance: 27.2%) for CCC

GHS hazard classification: none of the hazards selected for correlation

NN-rank 24: ['CC1CC1', 'CO']

EI-MS matched molecular weight (relative abundance: 94.99%) for CC1CC1

GHS hazard classification: none of the hazards selected for correlation

EI-MS matched molecular weight (relative abundance: 36.9%) for CO

GHS hazard classification: Acute Toxic, Health Hazard

NN-rank 25: ['C=C(O)CCC']

NN/MS matches corresponding with tox GHS classification highlight in Bold font.

3. 1,4-Dimethoxybenzene: COC1=CC=C(C=C1)OC

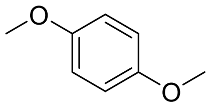

Summary Report of GHS Classification for NN/MS Matches:

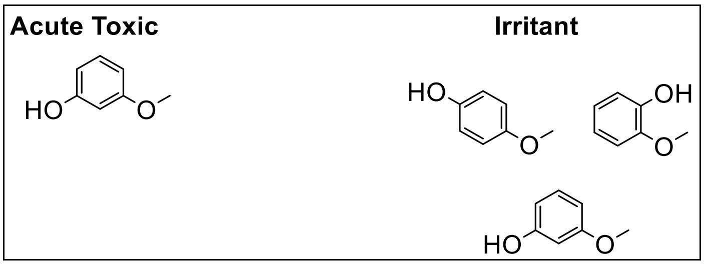

NN-rank 1: ['C', 'COc1ccc(O)cc1']

EI-MS matched molecular weight (relative abundance: 99.99%) for COc1ccc(O)cc1

GHS hazard classification: Irritant

NN-rank 2: ['C', 'COc1ccc(O)cc1']

EI-MS matched molecular weight (relative abundance: 99.99%) for COc1ccc(O)cc1

GHS hazard classification: Irritant

NN-rank 3: ['CO', 'COc1ccccc1']

NN-rank 4: ['CO', 'COc1ccccc1']

NN-rank 5: ['C', 'COc1ccc2cc1O2']

NN-rank 6: ['C', 'COc1ccc2cc1O2']

NN-rank 7: ['C', 'COc1ccc2cc1O2']

NN-rank 8: ['C', 'COc1ccc2cc1O2']

NN-rank 9: ['C', 'COc1ccc2c(c1)O2']

NN-rank 10: ['C', 'COc1ccc2c(c1)O2']

NN-rank 11: ['C', 'COc1ccc2c(c1)O2']

NN-rank 12: ['C', 'COc1ccc2c(c1)O2']

NN-rank 13: ['COc1ccc(O)cc1C']

NN-rank 14: ['COc1ccc(O)c(C)c1']

NN-rank 15: ['COc1ccc2cc1CO2']

NN-rank 16: ['COc1ccc2c(c1)CO2']

NN-rank 17: ['C', 'COc1ccccc1O']

NN/MS matches corresponding with tox GHS classification highlight in Bold font.

EI-MS matched molecular weight (relative abundance: 99.99%) for COc1ccccc1O

GHS hazard classification: Irritant

NN-rank 18: ['C', 'COc1ccccc1O']

EI-MS matched molecular weight (relative abundance: 99.99%) for COc1ccccc1O

GHS hazard classification: Irritant

NN-rank 19: ['C', 'COc1ccccc1O']

EI-MS matched molecular weight (relative abundance: 99.99%) for COc1ccccc1O

GHS hazard classification: Irritant

NN-rank 20: ['C', 'COc1ccccc1O']

EI-MS matched molecular weight (relative abundance: 99.99%) for COc1ccccc1O

GHS hazard classification: Irritant

NN-rank 21: ['C', 'COc1cccc(O)c1']

EI-MS matched molecular weight (relative abundance: 99.99%) for COc1cccc(O)c1

GHS hazard classification: Acute Toxic, Irritant

NN-rank 22: ['C', 'COc1cccc(O)c1']

EI-MS matched molecular weight (relative abundance: 99.99%) for COc1cccc(O)c1

GHS hazard classification: Acute Toxic, Irritant

NN-rank 23: ['C', 'COc1cccc(O)c1']

EI-MS matched molecular weight (relative abundance: 99.99%) for COc1cccc(O)c1

GHS hazard classification: Acute Toxic, Irritant

NN-rank 24: ['C', 'COc1cccc(O)c1']

EI-MS matched molecular weight (relative abundance: 99.99%) for COc1cccc(O)c1

GHS hazard classification: Acute Toxic, Irritant

NN-rank 25: ['COc1ccccc1OC']

NN/MS matches corresponding with tox GHS classification highlight in Bold font.

4. 2,3-Hexanedione: CC(C(CCC)=O)=O

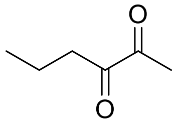

Summary Report of GHS Classification for NN/MS Matches:

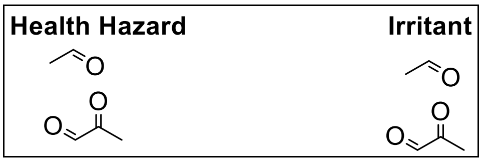

NN-rank 1: ['CCCC(O)C(C)=O']

NN-rank 2: ['CCCC(=O)CC', 'O']

NN-rank 3: ['CCCCC(C)=O', 'O']

NN-rank 4: ['C', 'CCC(=O)C(C)=O']

NN-rank 5: ['CC', 'CC(=O)C(C)=O']

NN-rank 6: ['CC(=O)C=O', 'CCC']

EI-MS matched molecular weight (relative abundance: 19.9%) for CC(=O)C=O

GHS hazard classification: Health Hazard, Irritant

EI-MS matched molecular weight (relative abundance: 99.99%) for CCC

GHS hazard classification: none of the hazards selected for correlation

NN-rank 7: ['CCC=C(O)C(C)=O']

NN-rank 8: ['CCC=CC(C)=O', 'O']

NN-rank 9: ['CC(=O)CO', 'CCC']

EI-MS matched molecular weight (relative abundance: 99.99%) for CCC

GHS hazard classification: none of the hazards selected for correlation

NN-rank 10: ['CCCC(=O)C(C)O']

NN-rank 11: ['CC=O', 'CCCC=O']

EI-MS matched molecular weight (relative abundance: 99.99%) for CC=O

GHS hazard classification: Health Hazard, Irritant

EI-MS matched molecular weight (relative abundance: 19.9%) for CCCC=O

GHS hazard classification: none of the hazards selected for correlation

NN-rank 12: ['CCCc1cc1=O', 'O']

NN-rank 13: ['CC=O', 'CCCCO']

NN/MS matches corresponding with tox GHS classification highlight in Bold font.

EI-MS matched molecular weight (relative abundance: 99.99%) for CC=O

GHS hazard classification: Health Hazard, Irritant

NN-rank 14: ['C', 'CCCC(=O)C=O']

NN-rank 15: ['CC(=O)C1=CCC1', 'O']

NN-rank 16: ['CC', 'C=C(O)C(C)=O']

NN-rank 17: ['CCC', 'O=c1cc1O']

EI-MS matched molecular weight (relative abundance: 99.99%) for CCC

GHS hazard classification: none of the hazards selected for correlation

NN-rank 18: ['CC', 'C=CC(C)=O', 'O']

NN-rank 19: ['C=CC(=O)CCC', 'O']

NN-rank 20: ['CC=O', 'CCC=C=O']

EI-MS matched molecular weight (relative abundance: 99.99%) for CC=O

GHS hazard classification: Health Hazard, Irritant

NN-rank 21: ['C', 'CCCC(C)=O', 'O']

NN-rank 22: ['C', 'CCCC(=O)CO']

NN-rank 23: ['C=C(CC)C(C)=O', 'O']

NN-rank 24: ['CC(C)=O', 'CCC', 'O']

EI-MS matched molecular weight (relative abundance: 99.99%) for CCC

GHS hazard classification: none of the hazards selected for correlation

NN-rank 25: ['CC=O', 'CCC=CO']

EI-MS matched molecular weight (relative abundance: 99.99%) for CC=O

GHS hazard classification: Health Hazard, Irritant

EI-MS matched molecular weight (relative abundance: 19.9%) for CCC=CO

GHS hazard classification: none of the hazards selected for correlation

NN/MS matches corresponding with tox GHS classification highlight in Bold font.

5. 2-Acetyl Furan: CC(=O)C1=CC=CO1

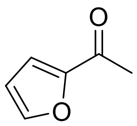

Summary Report of GHS Classification for NN/MS Matches:

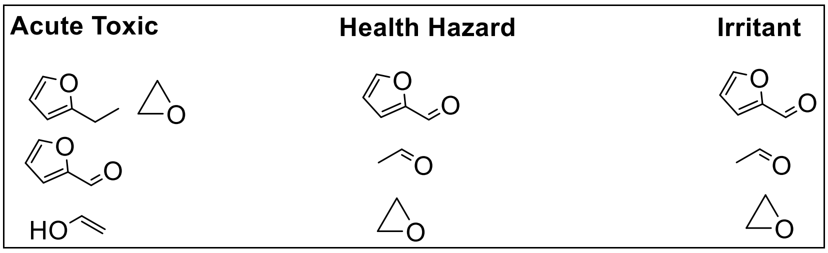

NN-rank 1: ['CC(O)c1ccco1']

NN-rank 2: ['CC=O', 'c1ccoc1']

EI-MS matched molecular weight (relative abundance: 16.89%) for CC=O

GHS hazard classification: Health Hazard, Irritant

NN-rank 3: ['C=C(O)c1ccco1']

NN-rank 4: ['c1coc(C2CO2)c1']

NN-rank 5: ['CCc1ccco1', 'O']

EI-MS matched molecular weight (relative abundance: 99.99%) for CCc1ccco1

GHS hazard classification: Acute Toxic

NN-rank 6: ['C', 'OCc1ccco1']

EI-MS matched molecular weight (relative abundance: 5.29%) for C

GHS hazard classification: none of the hazards selected for correlation

NN-rank 7: ['O=C1Cc2ccc1o2']

NN-rank 8: ['CCO', 'c1ccoc1']

NN-rank 9: ['C', 'O=Cc1ccco1']

EI-MS matched molecular weight (relative abundance: 5.29%) for C

GHS hazard classification: none of the hazards selected for correlation

EI-MS matched molecular weight (relative abundance: 99.99%) for O=Cc1ccco1

GHS hazard classification: Acute Toxic, Health Hazard, Irritant

NN-rank 10: ['O=C1Cc2ccoc21']

NN-rank 11: ['C=C=O', 'c1ccoc1']

NN/MS matches corresponding with tox GHS classification highlight in Bold font.

NN-rank 12: ['C1=C(c2ccco2)O1']

NN-rank 13: ['C=Cc1ccco1', 'O']

NN-rank 14: ['C=CO', 'c1ccoc1']

EI-MS matched molecular weight (relative abundance: 16.89%) for C=CO

GHS hazard classification: Acute Toxic

NN-rank 15: ['CC1(O)c2ccc1o2']

NN-rank 16: ['COCc1ccco1']

NN-rank 17: ['C1CO1', 'c1ccoc1']

EI-MS matched molecular weight (relative abundance: 16.89%) for C1CO1

GHS hazard classification: Acute Toxic, Health Hazard, Irritant

NN-rank 18: ['CC1Oc2ccoc21']

NN-rank 19: ['C', 'Cc1ccco1', 'O']

EI-MS matched molecular weight (relative abundance: 5.29%) for C

GHS hazard classification: none of the hazards selected for correlation

NN-rank 20: ['C', 'C=O', 'c1ccoc1']

EI-MS matched molecular weight (relative abundance: 5.29%) for C

GHS hazard classification: none of the hazards selected for correlation

NN-rank 21: ['Oc1cc2ccc1o2']

NN-rank 22: ['OCCc1ccco1']

NN-rank 23: ['CC1(O)c2ccoc21']

NN-rank 24: ['OC1=Cc2ccoc21']

NN-rank 25: ['O=CCc1ccco1']

NN/MS matches corresponding with tox GHS classification highlight in Bold font.

6. 2-Acetyl Pyridine: CC(=O)C1=CC=CC=N1

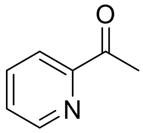

Summary Report of GHS Classification for NN/MS Matches:

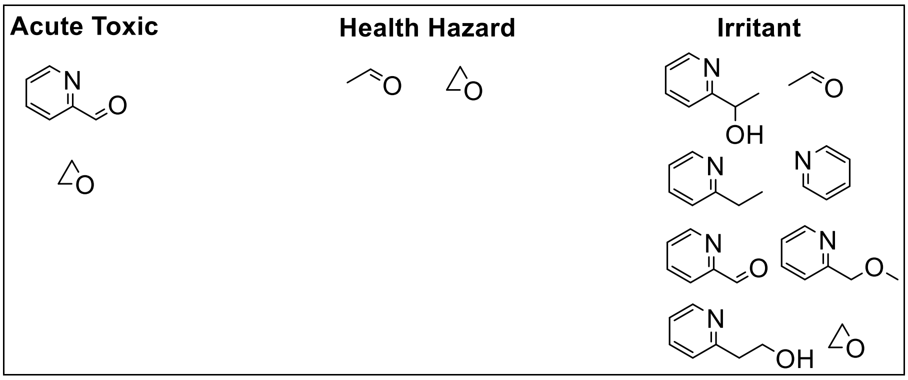

NN-rank 1: ['CC(O)c1ccccn1']

EI-MS matched molecular weight (relative abundance: 5.29%) for CC(O)c1ccccn1

GHS hazard classification: Irritant

NN-rank 2: ['CC=O', 'c1ccncc1']

EI-MS matched molecular weight (relative abundance: 42.29%) for CC=O

GHS hazard classification: Health Hazard, Irritant

EI-MS matched molecular weight (relative abundance: 78.49%) for c1ccncc1

GHS hazard classification: Irritant

NN-rank 3: ['C', 'OCc1ccccn1']

EI-MS matched molecular weight (relative abundance: 6.29%) for C

GHS hazard classification: none of the hazards selected for correlation

NN-rank 4: ['C=C(O)c1ccccn1']

NN-rank 5: ['O=C1Cc2cccc1n2']

NN-rank 6: ['CCc1ccccn1', 'O']

EI-MS matched molecular weight (relative abundance: 5.99%) for CCc1ccccn1

GHS hazard classification: Irritant

NN-rank 7: ['c1ccc(C2CO2)nc1']

NN-rank 8: ['C', 'O=Cc1ccccn1']

EI-MS matched molecular weight (relative abundance: 6.29%) for C

NN/MS matches corresponding with tox GHS classification highlight in Bold font.

GHS hazard classification: none of the hazards selected for correlation

EI-MS matched molecular weight (relative abundance: 5.99%) for O=Cc1ccccn1

GHS hazard classification: Acute Toxic, Irritant

NN-rank 9: ['O=C1Cc2cccnc21']

NN-rank 10: ['O=C1Cc2ccc1nc2']

NN-rank 11: ['O=C1C[n+]2ccccc21']

NN-rank 12: ['CCO', 'c1ccncc1']

EI-MS matched molecular weight (relative abundance: 78.49%) for c1ccncc1

GHS hazard classification: Irritant

NN-rank 13: ['CC(=O)c1cc2cc-2n1']

NN-rank 14: ['C1=C(c2ccccn2)O1']

NN-rank 15: ['C=C=O', 'c1ccncc1']

EI-MS matched molecular weight (relative abundance: 78.49%) for c1ccncc1

GHS hazard classification: Irritant

NN-rank 16: ['COCc1ccccn1']

EI-MS matched molecular weight (relative abundance: 5.29%) for COCc1ccccn1

GHS hazard classification: Irritant

NN-rank 17: ['CC1(O)C2=CC=C1N=C2']

NN-rank 18: ['CC1(O)c2cccc1n2']

NN-rank 19: ['C=Cc1ccccn1', 'O']

NN-rank 20: ['CC(=O)c1nc2ccc1-2']

NN-rank 21: ['C', 'Cc1ccccn1', 'O']

EI-MS matched molecular weight (relative abundance: 6.29%) for C

GHS hazard classification: none of the hazards selected for correlation

NN-rank 22: ['Cc1cccc(C=O)n1']

NN-rank 23: ['C', 'C=O', 'c1ccncc1']

EI-MS matched molecular weight (relative abundance: 6.29%) for C

GHS hazard classification: none of the hazards selected for correlation

EI-MS matched molecular weight (relative abundance: 78.49%) for c1ccncc1

GHS hazard classification: Irritant

NN-rank 24: ['C1CO1', 'c1ccncc1']

EI-MS matched molecular weight (relative abundance: 42.29%) for C1CO1

GHS hazard classification: Acute Toxic, Health Hazard, Irritant

EI-MS matched molecular weight (relative abundance: 78.49%) for c1ccncc1

GHS hazard classification: Irritant

NN/MS matches corresponding with tox GHS classification highlight in Bold font.

NN-rank 25: ['OCCc1ccccn1']

EI-MS matched molecular weight (relative abundance: 5.29%) for OCCc1ccccn1

GHS hazard classification: Irritant

NN/MS matches corresponding with tox GHS classification highlight in Bold font.

7. 2-Acetylpyrazine: CC(=O)C1=NC=CN=C1

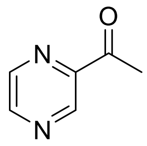

Summary Report of GHS Classification for NN/MS Matches:

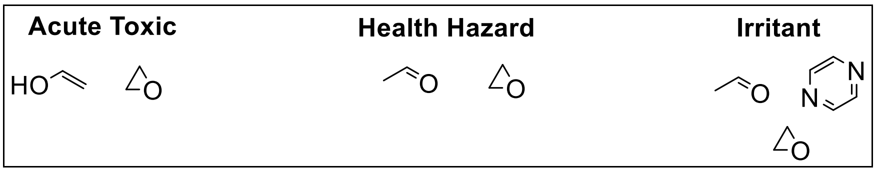

NN-rank 1: ['CC(O)c1cnccn1']

NN-rank 2: ['CC=O', 'c1cnccn1']

EI-MS matched molecular weight (relative abundance: 99.99%) for CC=O

GHS hazard classification: Health Hazard, Irritant

EI-MS matched molecular weight (relative abundance: 28.05%) for c1cnccn1

GHS hazard classification: Irritant

NN-rank 3: ['CCc1cnccn1', 'O']

NN-rank 4: ['C', 'O=Cc1cnccn1']

EI-MS matched molecular weight (relative abundance: 21.0%) for C

GHS hazard classification: none of the hazards selected for correlation

NN-rank 5: ['C', 'OCc1cnccn1']

EI-MS matched molecular weight (relative abundance: 21.0%) for C

GHS hazard classification: none of the hazards selected for correlation

NN-rank 6: ['C=C(O)c1cnccn1']

NN-rank 7: ['O=C1Cc2cnc1cn2']

NN-rank 8: ['c1cnc(C2CO2)cn1']

NN-rank 9: ['O=C1Cc2cncc1n2']

NN-rank 10: ['CCO', 'c1cnccn1']

EI-MS matched molecular weight (relative abundance: 28.05%) for c1cnccn1

GHS hazard classification: Irritant

NN-rank 11: ['C=Cc1cnccn1', 'O']

NN-rank 12: ['O=C1Cc2nccnc21']

NN/MS matches corresponding with tox GHS classification highlight in Bold font.

NN-rank 13: ['C', 'Cc1cnccn1', 'O']

EI-MS matched molecular weight (relative abundance: 21.0%) for C

GHS hazard classification: none of the hazards selected for correlation

NN-rank 14: ['C=C=O', 'c1cnccn1']

EI-MS matched molecular weight (relative abundance: 28.05%) for c1cnccn1

GHS hazard classification: Irritant

NN-rank 15: ['C', 'C=O', 'c1cnccn1']

EI-MS matched molecular weight (relative abundance: 21.0%) for C

GHS hazard classification: none of the hazards selected for correlation

EI-MS matched molecular weight (relative abundance: 28.05%) for c1cnccn1

GHS hazard classification: Irritant

NN-rank 16: ['OCCc1cnccn1']

NN-rank 17: ['CC1(O)C2=CN=C1C=N2']

NN-rank 18: ['COCc1cnccn1']

NN-rank 19: ['C1=C(c2cnccn2)O1']

NN-rank 20: ['CC=O', 'c1nc2cnc1-2']

EI-MS matched molecular weight (relative abundance: 99.99%) for CC=O

GHS hazard classification: Health Hazard, Irritant

NN-rank 21: ['C=CO', 'c1cnccn1']

EI-MS matched molecular weight (relative abundance: 99.99%) for C=CO

GHS hazard classification: Acute Toxic

EI-MS matched molecular weight (relative abundance: 28.05%) for c1cnccn1

GHS hazard classification: Irritant

NN-rank 22: ['C', 'O=C1C2=CN=C1C=N2']

EI-MS matched molecular weight (relative abundance: 21.0%) for C

GHS hazard classification: none of the hazards selected for correlation

NN-rank 23: ['C1CO1', 'c1cnccn1']

EI-MS matched molecular weight (relative abundance: 99.99%) for C1CO1

GHS hazard classification: Acute Toxic, Health Hazard, Irritant

EI-MS matched molecular weight (relative abundance: 28.05%) for c1cnccn1

GHS hazard classification: Irritant

NN-rank 24: ['Cc1cnc(C=O)cn1']

NN-rank 25: ['CC(=O)c1cnccn1']

NN/MS matches corresponding with tox GHS classification highlight in Bold font.

8. 2-Ethyl-3-methyl pyrazine: CCC1=NC=CN=C1C

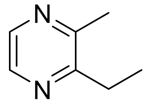

Summary Report of GHS Classification for NN/MS Matches:

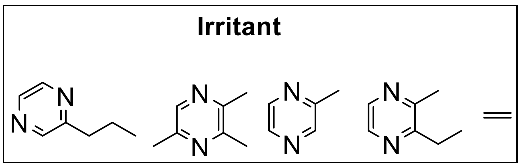

NN-rank 1: ['C', 'CCc1cnccn1']

NN-rank 2: ['C', 'Cc1nccnc1C']

NN-rank 3: ['CC', 'Cc1cnccn1']

EI-MS matched molecular weight (relative abundance: 10.1%) for Cc1cnccn1

GHS hazard classification: Irritant

NN-rank 4: ['C=Cc1nccnc1C']

NN-rank 5: ['c1cnc2c(n1)CCC2']

NN-rank 6: ['CCc1nccnc1C']

EI-MS matched molecular weight (relative abundance: 99.9%) for CCc1nccnc1C

GHS hazard classification: Irritant

NN-rank 7: ['Cc1nc2cnc1CC2']

NN-rank 8: ['Cc1ncc2nc1CC2']

NN-rank 9: ['CCCc1cnccn1']

EI-MS matched molecular weight (relative abundance: 99.9%) for CCCc1cnccn1

GHS hazard classification: Irritant

NN-rank 10: ['C', 'c1cnc2c(n1)CC2']

NN-rank 11: ['Cc1cnc(C)c(C)n1']

EI-MS matched molecular weight (relative abundance: 99.9%) for Cc1cnc(C)c(C)n1

GHS hazard classification: Irritant

NN-rank 12: ['Cc1ncc[n+]2c1CC2']

NN-rank 13: ['CC1Cc2nccnc21']

NN-rank 14: ['Cc1cnc(C)c(C)n1']

EI-MS matched molecular weight (relative abundance: 99.9%) for Cc1cnc(C)c(C)n1

NN/MS matches corresponding with tox GHS classification highlight in Bold font.

GHS hazard classification: Irritant

NN-rank 15: ['CC1=NC2=CN=C1C2C']

NN-rank 16: ['Cc1ncc[n+](C)c1C']

EI-MS matched molecular weight (relative abundance: 96.0%) for Cc1ncc[n+](C)c1C

GHS hazard classification: none of the hazards selected for correlation

NN-rank 17: ['Cc1ncc2nc1C2C']

NN-rank 18: ['CC(C)c1cnccn1']

EI-MS matched molecular weight (relative abundance: 99.9%) for CC(C)c1cnccn1

GHS hazard classification: none of the hazards selected for correlation

NN-rank 19: ['C', 'c1cnc2c(n1)CC2']

NN-rank 20: ['C', 'CC1=NC2=CN=C1C2']

NN-rank 21: ['C=C', 'Cc1cnccn1']

EI-MS matched molecular weight (relative abundance: 26.0%) for C=C

GHS hazard classification: Irritant

EI-MS matched molecular weight (relative abundance: 10.1%) for Cc1cnccn1

GHS hazard classification: Irritant

NN-rank 22: ['Cc1ncc[n+]2c1C2C']

NN-rank 23: ['C', 'C', 'Cc1cnccn1']

EI-MS matched molecular weight (relative abundance: 10.1%) for Cc1cnccn1

GHS hazard classification: Irritant

NN-rank 24: ['C', 'Cc1ncc2nc1C2']

NN-rank 25: ['C1=Cc2nccnc2C1']

NN/MS matches corresponding with tox GHS classification highlight in Bold font.

9. 2-Isopropyl-4-methyl thiazole: CC1=CSC(=N1)C(C)C

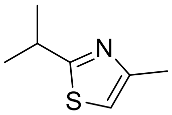

Summary Report of GHS Classification for NN/MS Matches:

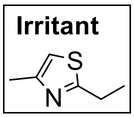

NN-rank 1: ['C', 'CCc1nc(C)cs1']

EI-MS matched molecular weight (relative abundance: 99.99%) for CCc1nc(C)cs1

GHS hazard classification: Irritant

NN-rank 2: ['C', 'CCc1nc(C)cs1']

EI-MS matched molecular weight (relative abundance: 99.99%) for CCc1nc(C)cs1

GHS hazard classification: Irritant

NN-rank 3: ['CCC', 'Cc1cscn1']

NN-rank 4: ['C', 'C', 'Cc1csc(C)n1']

NN-rank 5: ['CC1(C)Cc2csc1n2']

NN-rank 6: ['CC1CCc2csc1n2']

NN-rank 7: ['CC1CCc2csc1n2']

NN-rank 8: ['C=C(C)c1nc(C)cs1']

NN-rank 9: ['C=C(C)c1nc(C)cs1']

NN-rank 10: ['Cc1csc(C2CC2)n1']

NN-rank 11: ['CCc1csc(CC)n1']

EI-MS matched molecular weight (relative abundance: 18.79%) for CCc1csc(CC)n1

GHS hazard classification: none of the hazards selected for correlation

NN-rank 12: ['CCc1csc(CC)n1']

EI-MS matched molecular weight (relative abundance: 18.79%) for CCc1csc(CC)n1

GHS hazard classification: none of the hazards selected for correlation

NN-rank 13: ['C', 'CC(C)c1nccs1']

EI-MS matched molecular weight (relative abundance: 99.99%) for CC(C)c1nccs1

GHS hazard classification: none of the hazards selected for correlation

NN-rank 14: ['C', 'C=Cc1nc(C)cs1']

NN/MS matches corresponding with tox GHS classification highlight in Bold font.

NN-rank 15: ['C', 'C=Cc1nc(C)cs1']

NN-rank 16: ['C', 'CC1Cc2csc1n2']

NN-rank 17: ['C', 'CC1Cc2csc1n2']

NN-rank 18: ['CC(C)Cc1cscn1']

EI-MS matched molecular weight (relative abundance: 18.79%) for CC(C)Cc1cscn1

GHS hazard classification: none of the hazards selected for correlation

NN-rank 19: ['CCCc1nc(C)cs1']

EI-MS matched molecular weight (relative abundance: 18.79%) for CCCc1nc(C)cs1

GHS hazard classification: none of the hazards selected for correlation

NN-rank 20: ['CCCc1nc(C)cs1']

EI-MS matched molecular weight (relative abundance: 18.79%) for CCCc1nc(C)cs1

GHS hazard classification: none of the hazards selected for correlation

NN-rank 21: ['C=CC', 'Cc1cscn1']

EI-MS matched molecular weight (relative abundance: 5.19%) for C=CC

GHS hazard classification: none of the hazards selected for correlation

NN-rank 22: ['C=CC', 'Cc1cscn1']

EI-MS matched molecular weight (relative abundance: 5.19%) for C=CC

GHS hazard classification: none of the hazards selected for correlation

NN-rank 23: ['C', 'CC', 'Cc1cscn1']

NN-rank 24: ['C', 'CC', 'Cc1cscn1']

NN-rank 25: ['C', 'C', 'c1sc2nc1CC2']

NN/MS matches corresponding with tox GHS classification highlight in Bold font.

10. 2,3-Butanedione: CC(=O)C(=O)C

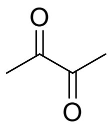

NN-rank 1: ['CC(=O)C(C)O']

NN-rank 2: ['CC(=O)C(C)O']

NN-rank 3: ['C=C(O)C(C)=O']

NN-rank 4: ['C=C(O)C(C)=O']

NN-rank 5: ['C', 'CC(=O)CO']

NN-rank 6: ['C', 'CC(=O)CO']

NN-rank 7: ['CC1OCC1=O']

NN-rank 8: ['CC1OCC1=O']

NN-rank 9: ['CC(=O)C1CO1']

NN-rank 10: ['CC(=O)C1CO1']

NN-rank 11: ['C', 'CC(=O)C=O']

NN-rank 12: ['C', 'CC(=O)C=O']

NN-rank 13: ['C=C1OCC1=O']

NN-rank 14: ['C=C1OCC1=O']

NN-rank 15: ['CCC(C)=O', 'O']

NN-rank 16: ['CCC(C)=O', 'O']

NN-rank 17: ['CC(=O)C1=CO1']

NN-rank 18: ['CC(=O)C1=CO1']

NN-rank 19: ['CC1(O)CC1=O']

NN-rank 20: ['CC1(O)CC1=O']

NN-rank 21: ['C=CC(C)=O', 'O']

NN-rank 22: ['C=CC(C)=O', 'O']

NN-rank 23: ['C', 'O=C1COC1']

NN-rank 24: ['C', 'O=C1COC1']

NN-rank 25: ['COCC(C)=O']

NN/MS matches corresponding with tox GHS classification highlight in Bold font.

11. 2-Methoxy-3-methyl pyrazine: CC1=NC=CN=C1OC

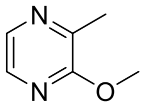

Summary Report of GHS Classification for NN/MS Matches:

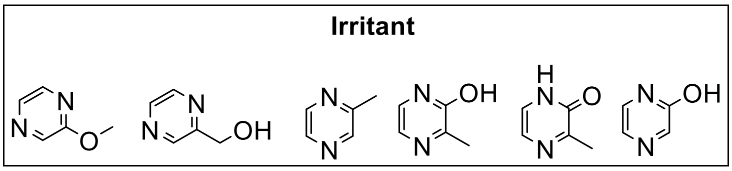

NN-rank 1: ['C', 'Cc1ncc[nH]c1=O']

EI-MS matched molecular weight (relative abundance: 48.0%) for Cc1ncc[nH]c1=O

GHS hazard classification: Irritant

NN-rank 2: ['C', 'Cc1nccnc1O']

EI-MS matched molecular weight (relative abundance: 48.0%) for Cc1nccnc1O

GHS hazard classification: Irritant

NN-rank 3: ['CO', 'Cc1cnccn1']

EI-MS matched molecular weight (relative abundance: 21.0%) for Cc1cnccn1

GHS hazard classification: Irritant

NN-rank 4: ['C', 'Cc1ncc2nc1O2']

NN-rank 5: ['C', 'O=Cc1cnccn1']

NN-rank 6: ['C', 'Cc1nc2cnc1o2']

NN-rank 7: ['Cc1c[nH]c(=O)c(C)n1']

EI-MS matched molecular weight (relative abundance: 26.0%) for Cc1c[nH]c(=O)c(C)n1

GHS hazard classification: none of the hazards selected for correlation

NN-rank 8: ['Cc1cnc(O)c(C)n1']

EI-MS matched molecular weight (relative abundance: 26.0%) for Cc1cnc(O)c(C)n1

GHS hazard classification: none of the hazards selected for correlation

NN-rank 9: ['C', 'COc1cnccn1']

EI-MS matched molecular weight (relative abundance: 48.0%) for COc1cnccn1

GHS hazard classification: Irritant

NN-rank 10: ['C', 'Cc1cncc(O)n1']

NN/MS matches corresponding with tox GHS classification highlight in Bold font.

EI-MS matched molecular weight (relative abundance: 48.0%) for Cc1cncc(O)n1

GHS hazard classification: none of the hazards selected for correlation

NN-rank 11: ['C', 'Cc1cnc(O)cn1']

EI-MS matched molecular weight (relative abundance: 48.0%) for Cc1cnc(O)cn1

GHS hazard classification: none of the hazards selected for correlation

NN-rank 12: ['C', 'c1cnc2c(n1)CO2']

NN-rank 13: ['C']

NN-rank 14: ['Cc1cnc(C)c(=O)[nH]1']

EI-MS matched molecular weight (relative abundance: 26.0%) for Cc1cnc(C)c(=O)[nH]1

GHS hazard classification: Irritant

NN-rank 15: ['Cc1cnc(C)c(O)n1']

EI-MS matched molecular weight (relative abundance: 26.0%) for Cc1cnc(C)c(O)n1

GHS hazard classification: Irritant

NN-rank 16: ['C', 'Cc1cnccn1', 'O']

EI-MS matched molecular weight (relative abundance: 21.0%) for Cc1cnccn1

GHS hazard classification: Irritant

NN-rank 17: ['C', 'OCc1cnccn1']

EI-MS matched molecular weight (relative abundance: 48.0%) for OCc1cnccn1

GHS hazard classification: Irritant

NN-rank 18: ['C', 'C', 'c1cnc2c(n1)O2']

EI-MS matched molecular weight (relative abundance: 21.0%) for c1cnc2c(n1)O2

GHS hazard classification: none of the hazards selected for correlation

NN-rank 19: ['C=O', 'Cc1cnccn1']

EI-MS matched molecular weight (relative abundance: 21.0%) for Cc1cnccn1

GHS hazard classification: Irritant

NN-rank 20: ['Cc1nc2cnc1OC2']

NN-rank 21: ['C', 'C', 'Oc1cnccn1']

EI-MS matched molecular weight (relative abundance: 31.0%) for Oc1cnccn1

GHS hazard classification: Irritant

NN-rank 22: ['COc1cncc(C)n1']

EI-MS matched molecular weight (relative abundance: 26.0%) for COc1cncc(C)n1

GHS hazard classification: none of the hazards selected for correlation

NN-rank 23: ['COc1cnc(C)cn1']

EI-MS matched molecular weight (relative abundance: 26.0%) for COc1cnc(C)cn1

NN/MS matches corresponding with tox GHS classification highlight in Bold font.

GHS hazard classification: none of the hazards selected for correlation

NN-rank 24: ['C', 'COc1cnccn1']

EI-MS matched molecular weight (relative abundance: 48.0%) for COc1cnccn1

GHS hazard classification: Irritant

NN-rank 25: ['Cc1ncc2nc1OC2']

NN/MS matches corresponding with tox GHS classification highlight in Bold font.

12. 2-Methyl Butyric Acid: CCC(C)C(=O)O

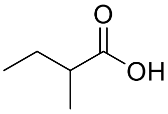

Summary Report of GHS Classification for NN/MS Matches:

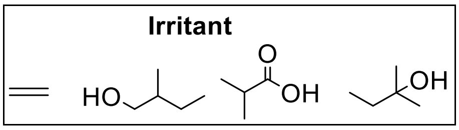

NN-rank 1: ['C', 'CCCC(=O)O']

EI-MS matched molecular weight (relative abundance: 23.8%) for CCCC(=O)O

GHS hazard classification: none of the hazards selected for correlation

NN-rank 2: ['CC', 'CCC(=O)O']

EI-MS matched molecular weight (relative abundance: 62.12%) for CC

GHS hazard classification: none of the hazards selected for correlation

EI-MS matched molecular weight (relative abundance: 14.8%) for CCC(=O)O

GHS hazard classification: none of the hazards selected for correlation

NN-rank 3: ['CC=C(C)C(=O)O']

NN-rank 4: ['CCC(C)CO', 'O']

EI-MS matched molecular weight (relative abundance: 23.8%) for CCC(C)CO

GHS hazard classification: Irritant

NN-rank 5: ['CCC(C)CO', 'O']

EI-MS matched molecular weight (relative abundance: 23.8%) for CCC(C)CO

GHS hazard classification: Irritant

NN-rank 6: ['CCC(C)C=O', 'O']

NN-rank 7: ['C=C(CC)C(=O)O']

NN-rank 8: ['C', 'CC(C)C(=O)O']

EI-MS matched molecular weight (relative abundance: 23.8%) for CC(C)C(=O)O

GHS hazard classification: Irritant

NN-rank 9: ['C', 'CC', 'CC(=O)O']

EI-MS matched molecular weight (relative abundance: 62.12%) for CC

GHS hazard classification: none of the hazards selected for correlation

NN-rank 10: ['C=CC(C)C(=O)O']

NN/MS matches corresponding with tox GHS classification highlight in Bold font.

NN-rank 11: ['C', 'CC=CC(=O)O']

NN-rank 12: ['CCC(C)C(=O)O']

NN-rank 13: ['C=CC(=O)O', 'CC']

EI-MS matched molecular weight (relative abundance: 62.12%) for CC

GHS hazard classification: none of the hazards selected for correlation

NN-rank 14: ['C', 'C=C(C)C(=O)O']

NN-rank 15: ['CCCC', 'O=CO']

EI-MS matched molecular weight (relative abundance: 63.87%) for CCCC

GHS hazard classification: none of the hazards selected for correlation

EI-MS matched molecular weight (relative abundance: 16.78%) for O=CO

GHS hazard classification: none of the hazards selected for correlation

NN-rank 16: ['CCC(C)C', 'O', 'O']

NN-rank 17: ['CCC(C)C=O', 'O']

NN-rank 18: ['CCC(C)(C)O', 'O']

EI-MS matched molecular weight (relative abundance: 23.8%) for CCC(C)(C)O

GHS hazard classification: Irritant

NN-rank 19: ['C=C', 'CCC(=O)O']

EI-MS matched molecular weight (relative abundance: 32.39%) for C=C

GHS hazard classification: Irritant

EI-MS matched molecular weight (relative abundance: 14.8%) for CCC(=O)O

GHS hazard classification: none of the hazards selected for correlation

NN-rank 20: ['CCC1(C)OC1=O']

NN-rank 21: ['CCC1(C)OC1=O']

NN-rank 22: ['CC', 'CC1OC1=O']

EI-MS matched molecular weight (relative abundance: 62.12%) for CC

GHS hazard classification: none of the hazards selected for correlation

NN-rank 23: ['O=C(O)C1CCC1']

NN-rank 24: ['C', 'CCC1OC1=O']

NN-rank 25: ['CCC(C)(O)CO']

NN/MS matches corresponding with tox GHS classification highlight in Bold font.

13. 2-Methylbutyl acetate: CCC(C)COC(=O)C

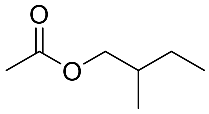

Summary Report of GHS Classification for NN/MS Matches:

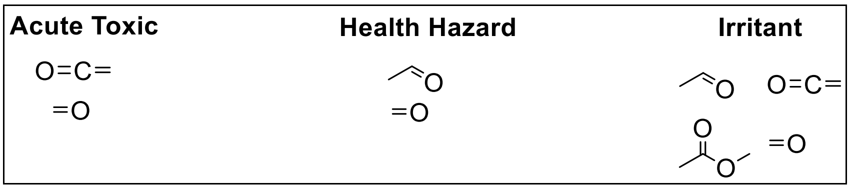

NN-rank 1: ['CC=O', 'CCC(C)CO']

> **EI-MS matched molecular weight (relative abundance: 99.99%) for CC=O**
>
> **GHS hazard classification: Health Hazard, Irritant**

NN-rank 2: ['CC(=O)O', 'C=C(C)CC']

NN-rank 3: ['CC(=O)O', 'CCC(C)C']

NN-rank 4: ['CCCC', 'COC(C)=O']

> **EI-MS matched molecular weight (relative abundance: 6.19%) for CCCC**
>
> **GHS hazard classification: none of the hazards selected for correlation**
>
> **EI-MS matched molecular weight (relative abundance: 15.69%) for COC(C)=O**
>
> **GHS hazard classification: Irritant**

NN-rank 5: ['CCC(C)=COC(C)=O']

NN-rank 6: ['C', 'CCCCOC(C)=O']

NN-rank 7: ['CC=O', 'CCC(C)C=O']

> **EI-MS matched molecular weight (relative abundance: 99.99%) for CC=O**
>
> **GHS hazard classification: Health Hazard, Irritant**

NN-rank 8: ['CCC(C)CO', 'CCO']

NN-rank 9: ['C=C=O', 'CCC(C)CO']

> **EI-MS matched molecular weight (relative abundance: 10.19%) for C=C=O**
>
> **GHS hazard classification: Acute Toxic, Irritant**

NN-rank 10: ['C=C(CC)COC(C)=O']

NN-rank 11: ['CC(=O)CCC(C)CO']

NN-rank 12: ['CCC(CO)CC(C)=O']

NN-rank 13: ['C', 'CC(=O)O', 'C=CCC']

NN/MS matches corresponding with tox GHS classification highlight in Bold font.

EI-MS matched molecular weight (relative abundance: 16.39%) for C=CCC

GHS hazard classification: none of the hazards selected for correlation

NN-rank 14: ['C', 'CC(=O)OCC(C)C']

NN-rank 15: ['C', 'C=O', 'CCC(C)CO']

EI-MS matched molecular weight (relative abundance: 8.19%) for C=O

GHS hazard classification: Acute Toxic, Health Hazard, Irritant

NN-rank 16: ['C=CC(C)COC(C)=O']

NN-rank 17: ['C', 'CCC=COC(C)=O']

NN-rank 18: ['CC=O', 'CCC(C)=C=O']

EI-MS matched molecular weight (relative abundance: 99.99%) for CC=O

GHS hazard classification: Health Hazard, Irritant

NN-rank 19: ['CC', 'CCCOC(C)=O']

EI-MS matched molecular weight (relative abundance: 8.19%) for CC

GHS hazard classification: none of the hazards selected for correlation

NN-rank 20: ['C', 'CCC(C)COC=O']

NN-rank 21: ['CCC(C)COC(C)O']

NN-rank 22: ['CC', 'CC(=O)O', 'C=CC']

EI-MS matched molecular weight (relative abundance: 8.19%) for CC

GHS hazard classification: none of the hazards selected for correlation

EI-MS matched molecular weight (relative abundance: 10.19%) for C=CC

GHS hazard classification: none of the hazards selected for correlation

NN-rank 23: ['CC', 'CC=COC(C)=O']

EI-MS matched molecular weight (relative abundance: 8.19%) for CC

GHS hazard classification: none of the hazards selected for correlation

NN-rank 24: ['C', 'CCC', 'COC(C)=O']

EI-MS matched molecular weight (relative abundance: 99.99%) for CCC

GHS hazard classification: none of the hazards selected for correlation

EI-MS matched molecular weight (relative abundance: 15.69%) for COC(C)=O

GHS hazard classification: Irritant

NN-rank 25: ['C=CCC', 'COC(C)=O']

EI-MS matched molecular weight (relative abundance: 16.39%) for C=CCC

GHS hazard classification: none of the hazards selected for correlation

EI-MS matched molecular weight (relative abundance: 15.69%) for COC(C)=O

GHS hazard classification: Irritant

NN/MS matches corresponding with tox GHS classification highlight in Bold font.

NN/MS matches corresponding with tox GHS classification highlight in Bold font.

14. 2-Phenylethanol: C1=CC=C(C=C1)CCO

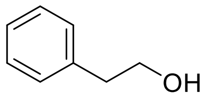

Summary Report of GHS Classification for NN/MS Matches:

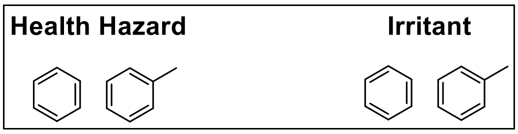

NN-rank 1: ['C=Cc1ccccc1', 'O']

NN-rank 2: ['C#Cc1ccccc1', 'O']

NN-rank 3: ['CCc1ccccc1', 'O']

NN-rank 4: ['c1ccc2c(c1)CC2', 'O']

NN-rank 5: ['c1ccc2c(c1)CC2', 'O']

NN-rank 6: ['OC=Cc1ccccc1']

NN-rank 7: ['CO', 'Cc1ccccc1']

EI-MS matched molecular weight (relative abundance: 99.99%) for Cc1ccccc1

GHS hazard classification: Health Hazard, Irritant

NN-rank 8: ['O=CCc1ccccc1']

NN-rank 9: ['C=C(O)c1ccccc1']

NN-rank 10: ['C1=Cc2ccccc21', 'O']

NN-rank 11: ['C1=Cc2ccccc21', 'O']

NN-rank 12: ['C=C', 'O', 'c1ccccc1']

EI-MS matched molecular weight (relative abundance: 5.67%) for c1ccccc1

GHS hazard classification: Health Hazard, Irritant

NN-rank 13: ['CC(O)c1ccccc1']

NN-rank 14: ['C', 'Cc1ccccc1', 'O']

EI-MS matched molecular weight (relative abundance: 99.99%) for Cc1ccccc1

GHS hazard classification: Health Hazard, Irritant

NN-rank 15: ['OC#Cc1ccccc1']

NN-rank 16: ['CCO', 'c1ccccc1']

EI-MS matched molecular weight (relative abundance: 5.67%) for c1ccccc1

GHS hazard classification: Health Hazard, Irritant

NN/MS matches corresponding with tox GHS classification highlight in Bold font.

NN-rank 17: ['C=C1c2ccccc21', 'O']

NN-rank 18: ['C=C1c2ccccc21', 'O']

NN-rank 19: ['c1ccc(C2CO2)cc1']

NN-rank 20: ['C#C', 'O', 'c1ccccc1']

EI-MS matched molecular weight (relative abundance: 5.67%) for c1ccccc1

GHS hazard classification: Health Hazard, Irritant

NN-rank 21: ['Cc1ccccc1C', 'O']

NN-rank 22: ['Cc1ccccc1C', 'O']

NN-rank 23: ['c1ccc2c(c1)C2', 'CO']

NN-rank 24: ['c1ccc2c(c1)C2', 'CO']

NN-rank 25: ['C1#Cc2ccccc21', 'O']

NN/MS matches corresponding with tox GHS classification highlight in Bold font.

15. 2-Phenylethyl Acetate: CC(=O)OCCC1=CC=CC=C1

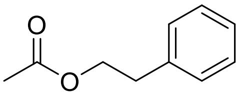

Summary Report of GHS Classification for NN/MS Matches:

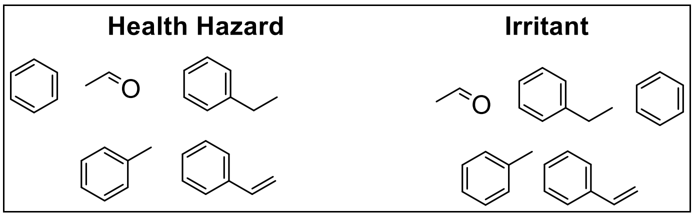

NN-rank 1: ['CC=O', 'OCCc1ccccc1']

EI-MS matched molecular weight (relative abundance: 58.99%) for CC=O

GHS hazard classification: Health Hazard, Irritant

NN-rank 2: ['CC=O', 'O=CCc1ccccc1']

EI-MS matched molecular weight (relative abundance: 58.99%) for CC=O

GHS hazard classification: Health Hazard, Irritant

NN-rank 3: ['C=Cc1ccccc1', 'CC(=O)O']

EI-MS matched molecular weight (relative abundance: 5.49%) for C=Cc1ccccc1

GHS hazard classification: Health Hazard, Irritant

NN-rank 4: ['CC(=O)c1ccccc1CCO']

NN-rank 5: ['CC(=O)c1ccccc1CCO']

NN-rank 6: ['CCO', 'OCCc1ccccc1']

NN-rank 7: ['C#Cc1ccccc1', 'CC(=O)O']

NN-rank 8: ['COC(C)=O', 'Cc1ccccc1']

EI-MS matched molecular weight (relative abundance: 18.29%) for Cc1ccccc1

GHS hazard classification: Health Hazard, Irritant

NN-rank 9: ['CC=O', 'O=C=Cc1ccccc1']

EI-MS matched molecular weight (relative abundance: 58.99%) for CC=O

GHS hazard classification: Health Hazard, Irritant

NN-rank 10: ['CC(=O)O', 'CCc1ccccc1']

EI-MS matched molecular weight (relative abundance: 11.69%) for CCc1ccccc1

GHS hazard classification: Health Hazard, Irritant

NN/MS matches corresponding with tox GHS classification highlight in Bold font.

NN-rank 11: ['CC(=O)OC=Cc1ccccc1']

NN-rank 12: ['C=C=O', 'OCCc1ccccc1']

NN-rank 13: ['C=O', 'CC=O', 'Cc1ccccc1']

EI-MS matched molecular weight (relative abundance: 58.99%) for CC=O

GHS hazard classification: Health Hazard, Irritant

EI-MS matched molecular weight (relative abundance: 18.29%) for Cc1ccccc1

GHS hazard classification: Health Hazard, Irritant

NN-rank 14: ['CC(=O)c1ccccc1CC=O']

NN-rank 15: ['CC(=O)c1ccccc1CC=O']

NN-rank 16: ['CCO', 'O=CCc1ccccc1']

NN-rank 17: ['CC(=O)OC#Cc1ccccc1']

NN-rank 18: ['C=C=O', 'O=CCc1ccccc1']

NN-rank 19: ['O=C1Cc2ccccc2CCO1']

NN-rank 20: ['O=C1Cc2ccccc2CCO1']

NN-rank 21: ['C', 'C=O', 'OCCc1ccccc1']

NN-rank 22: ['CCOC(C)=O', 'c1ccccc1']

EI-MS matched molecular weight (relative abundance: 5.49%) for c1ccccc1

GHS hazard classification: Health Hazard, Irritant

NN-rank 23: ['CC(O)OCCc1ccccc1']

NN-rank 24: ['C', 'O=COCCc1ccccc1']

NN-rank 25: ['C', 'C=O', 'O=CCc1ccccc1']

NN/MS matches corresponding with tox GHS classification highlight in Bold font.

16. 2-Propanol: CC(C)O

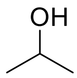

Summary Report of GHS Classification for NN/MS Matches:

Acute Toxic	Health Hazard	Irritant
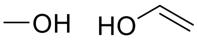	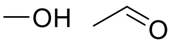	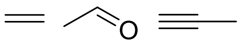

NN-rank 1: ['CCC', 'O']

EI-MS matched molecular weight (relative abundance: 19.12%) for CCC

GHS hazard classification: none of the hazards selected for correlation

NN-rank 2: ['C', 'CCO']

EI-MS matched molecular weight (relative abundance: 9.51%) for C

GHS hazard classification: none of the hazards selected for correlation

EI-MS matched molecular weight (relative abundance: 99.99%) for CCO

GHS hazard classification: none of the hazards selected for correlation

NN-rank 3: ['C', 'CCO']

EI-MS matched molecular weight (relative abundance: 9.51%) for C

GHS hazard classification: none of the hazards selected for correlation

EI-MS matched molecular weight (relative abundance: 99.99%) for CCO

GHS hazard classification: none of the hazards selected for correlation

NN-rank 4: ['C', 'CC', 'O']

EI-MS matched molecular weight (relative abundance: 9.51%) for C

GHS hazard classification: none of the hazards selected for correlation

EI-MS matched molecular weight (relative abundance: 12.51%) for CC

GHS hazard classification: none of the hazards selected for correlation

NN-rank 5: ['C', 'CC', 'O']

EI-MS matched molecular weight (relative abundance: 9.51%) for C

GHS hazard classification: none of the hazards selected for correlation

EI-MS matched molecular weight (relative abundance: 12.51%) for CC

GHS hazard classification: none of the hazards selected for correlation

NN-rank 6: ['C=CC', 'O']

EI-MS matched molecular weight (relative abundance: 8.21%) for C=CC

NN/MS matches corresponding with tox GHS classification highlight in Bold font.

GHS hazard classification: none of the hazards selected for correlation

NN-rank 7: ['C=CC', 'O']

EI-MS matched molecular weight (relative abundance: 8.21%) for C=CC

GHS hazard classification: none of the hazards selected for correlation

NN-rank 8: ['C=C(C)O']

NN-rank 9: ['C=C(C)O']

NN-rank 10: ['C', 'C=CO']

EI-MS matched molecular weight (relative abundance: 9.51%) for C

GHS hazard classification: none of the hazards selected for correlation

EI-MS matched molecular weight (relative abundance: 19.12%) for C=CO

GHS hazard classification: Acute Toxic

NN-rank 11: ['C', 'C=CO']

EI-MS matched molecular weight (relative abundance: 9.51%) for C

GHS hazard classification: none of the hazards selected for correlation

EI-MS matched molecular weight (relative abundance: 19.12%) for C=CO

GHS hazard classification: Acute Toxic

NN-rank 12: ['C', 'C', 'CO']

EI-MS matched molecular weight (relative abundance: 9.51%) for C

GHS hazard classification: none of the hazards selected for correlation

EI-MS matched molecular weight (relative abundance: 9.51%) for C

GHS hazard classification: none of the hazards selected for correlation

EI-MS matched molecular weight (relative abundance: 6.81%) for CO

GHS hazard classification: Acute Toxic, Health Hazard

NN-rank 13: ['C', 'C=C', 'O']

EI-MS matched molecular weight (relative abundance: 9.51%) for C

GHS hazard classification: none of the hazards selected for correlation

EI-MS matched molecular weight (relative abundance: 16.82%) for C=C

GHS hazard classification: Irritant

NN-rank 14: ['C', 'C=C', 'O']

EI-MS matched molecular weight (relative abundance: 9.51%) for C

GHS hazard classification: none of the hazards selected for correlation

EI-MS matched molecular weight (relative abundance: 16.82%) for C=C

GHS hazard classification: Irritant

NN-rank 15: ['CC1CO1']

NN-rank 16: ['CC1CO1']

NN/MS matches corresponding with tox GHS classification highlight in Bold font.

NN-rank 17: ['C', 'C#CO']

EI-MS matched molecular weight (relative abundance: 9.51%) for C

GHS hazard classification: none of the hazards selected for correlation

EI-MS matched molecular weight (relative abundance: 8.21%) for C#CO

GHS hazard classification: none of the hazards selected for correlation

NN-rank 18: ['C', 'C#CO']

EI-MS matched molecular weight (relative abundance: 9.51%) for C

GHS hazard classification: none of the hazards selected for correlation

EI-MS matched molecular weight (relative abundance: 8.21%) for C#CO

GHS hazard classification: none of the hazards selected for correlation

NN-rank 19: ['C#CC', 'O']

EI-MS matched molecular weight (relative abundance: 6.61%) for C#CC

GHS hazard classification: Irritant

NN-rank 20: ['C#CC', 'O']

EI-MS matched molecular weight (relative abundance: 6.61%) for C#CC

GHS hazard classification: Irritant

NN-rank 21: ['C', 'CC=O']

EI-MS matched molecular weight (relative abundance: 9.51%) for C

GHS hazard classification: none of the hazards selected for correlation

EI-MS matched molecular weight (relative abundance: 19.12%) for CC=O

GHS hazard classification: Health Hazard, Irritant

NN-rank 22: ['C', 'CC=O']

EI-MS matched molecular weight (relative abundance: 9.51%) for C

GHS hazard classification: none of the hazards selected for correlation

EI-MS matched molecular weight (relative abundance: 19.12%) for CC=O

GHS hazard classification: Health Hazard, Irritant

NN-rank 23: ['CCOC']

NN-rank 24: ['CCOC']

NN-rank 25: ['C', 'COC']

EI-MS matched molecular weight (relative abundance: 9.51%) for C

GHS hazard classification: none of the hazards selected for correlation

EI-MS matched molecular weight (relative abundance: 99.99%) for COC

GHS hazard classification: none of the hazards selected for correlation

NN/MS matches corresponding with tox GHS classification highlight in Bold font.

17. 2,3-Dimethylpyrazine: CC1=NC=CN=C1C

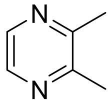

NN-rank 1: ['C', 'Cc1cnccn1']

NN-rank 2: ['C', 'Cc1cnccn1']

NN-rank 3: ['c1cnc2c(n1)CC2']

NN-rank 4: ['CC1=NC2=CN=C1C2']

NN-rank 5: ['CC1=NC2=CN=C1C2']

NN-rank 6: ['Cc1ncc2nc1C2']

NN-rank 7: ['Cc1ncc2nc1C2']

NN-rank 8: ['Cc1c2ncc[n+]1C2']

NN-rank 9: ['Cc1c2ncc[n+]1C2']

NN-rank 10: ['CCc1cnccn1']

NN-rank 11: ['CCc1cnccn1']

NN-rank 12: ['Cc1ncc[n+]2c1C2']

NN-rank 13: ['Cc1cncc(C)n1']

NN-rank 14: ['Cc1cncc(C)n1']

NN-rank 15: ['Cc1cnc(C)cn1']

NN-rank 16: ['Cc1cnc(C)cn1']

NN-rank 17: ['Cc1cncc[n+]1C']

EI-MS matched molecular weight (relative abundance: 90.7%) for Cc1cncc[n+]1C

GHS hazard classification: none of the hazards selected for correlation

NN-rank 18: ['Cc1cncc[n+]1C']

EI-MS matched molecular weight (relative abundance: 90.7%) for Cc1cncc[n+]1C

GHS hazard classification: none of the hazards selected for correlation

NN-rank 19: ['Cc1c[n+](C)ccn1']

EI-MS matched molecular weight (relative abundance: 90.7%) for Cc1c[n+](C)ccn1

GHS hazard classification: none of the hazards selected for correlation

NN-rank 20: ['CC', 'c1cnccn1']

NN-rank 21: ['C1=C2N=C3C(=N1)CC23']

NN-rank 22: ['C1=C2N=C3C(=N1)CC23']

NN-rank 23: ['C1=NC2=C3CC2C1=N3']

NN/MS matches corresponding with tox GHS classification highlight in Bold font.

NN-rank 24: ['C1=NC2=C3CC2C1=N3']

NN-rank 25: ['c1c[n+]2c3c(n1)CC32']

NN/MS matches corresponding with tox GHS classification highlight in Bold font.

18. 2,3,5-Trimethylpyrazine: CC1=CN=C(C(=N1)C)C

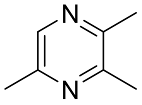

NN-rank 1: ['C', 'Cc1cncc(C)n1']

NN-rank 2: ['C', 'Cc1cnc(C)cn1']

NN-rank 3: ['C', 'Cc1nccnc1C']

NN-rank 4: ['Cc1nc2cnc1CC2']

NN-rank 5: ['Cc1ncc2nc1CC2']

NN-rank 6: ['CCc1cncc(C)n1']

NN-rank 7: ['Cc1cnc2c(n1)CC2']

NN-rank 8: ['CCc1cnc(C)cn1']

NN-rank 9: ['CCc1nccnc1C']

NN-rank 10: ['CCc1nccnc1C']

NN-rank 11: ['C', 'CC1=NC2=CN=C1C2']

NN-rank 12: ['C', 'Cc1ncc2nc1C2']

NN-rank 13: ['CCc1cncc(C)n1']

NN-rank 14: ['Cc1ncc2[n+](c1C)C2']

NN-rank 15: ['CCc1cnc(C)cn1']

NN-rank 16: ['Cc1nc2c(nc1C)C2']

NN-rank 17: ['Cc1ncc(C)[n+]2c1C2']

NN-rank 18: ['Cc1cnc(C)c(C)n1']

NN-rank 19: ['c1nc2c3nc1C(C2)C3']

NN-rank 20: ['CC', 'Cc1cnccn1']

NN-rank 21: ['C', 'Cc1cncc(C)n1']

NN-rank 22: ['Cc1c[n+](C)c(C)cn1']

EI-MS matched molecular weight (relative abundance: 66.49%) for Cc1c[n+](C)c(C)cn1

GHS hazard classification: none of the hazards selected for correlation

NN-rank 23: ['Cc1ncc[n+](C)c1C']

EI-MS matched molecular weight (relative abundance: 66.49%) for Cc1ncc[n+](C)c1C

GHS hazard classification: none of the hazards selected for correlation

NN/MS matches corresponding with tox GHS classification highlight in Bold font.

NN-rank 24: ['C', 'Cc1cnc(C)cn1']

NN-rank 25: ['C', 'Cc1cncc(C)n1']

NN/MS matches corresponding with tox GHS classification highlight in Bold font.

19. 2,3,5,6-Tetramethylpyrazine: CC1=C(N=C(C(=N1)C)C)C

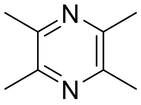

NN-rank 1: ['C', 'Cc1cnc(C)c(C)n1']

NN-rank 2: ['C', 'Cc1cnc(C)c(C)n1']

NN-rank 3: ['C', 'Cc1cnc(C)c(C)n1']

NN-rank 4: ['C', 'Cc1cnc(C)c(C)n1']

NN-rank 5: ['Cc1nc2c(C)nc1CC2']

NN-rank 6: ['Cc1nc2c(C)nc1CC2']

NN-rank 7: ['Cc1nc(C)c2nc1CC2']

NN-rank 8: ['Cc1nc(C)c2nc1CC2']

NN-rank 9: ['Cc1nc2c(nc1C)CC2']

NN-rank 10: ['Cc1nc2c(nc1C)CC2']

NN-rank 11: ['CCc1nc(C)cnc1C']

NN-rank 12: ['CCc1nc(C)cnc1C']

NN-rank 13: ['CCc1nc(C)cnc1C']

NN-rank 14: ['CCc1nc(C)cnc1C']

NN-rank 15: ['CCc1ncc(C)nc1C']

NN-rank 16: ['CCc1ncc(C)nc1C']

NN-rank 17: ['CCc1ncc(C)nc1C']

NN-rank 18: ['CCc1ncc(C)nc1C']

NN-rank 19: ['CCc1cnc(C)c(C)n1']

NN-rank 20: ['CCc1cnc(C)c(C)n1']

NN-rank 21: ['CCc1cnc(C)c(C)n1']

NN-rank 22: ['CCc1cnc(C)c(C)n1']

NN-rank 23: ['Cc1nc(C)c(C)nc1C']

NN-rank 24: ['Cc1nc(C)c(C)nc1C']

NN-rank 25: ['C', 'Cc1cnc(C)c(C)n1']

NN/MS matches corresponding with tox GHS classification highlight in Bold font.

20. 2,5-Dimethylpyrazine: CC1=CN=C(C=N1)C

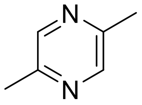

NN-rank 1: ['C', 'Cc1cnccn1']

NN-rank 2: ['C', 'Cc1cnccn1']

NN-rank 3: ['c1nc2cnc1CC2']

NN-rank 4: ['CCc1cnccn1']

NN-rank 5: ['CCc1cnccn1']

NN-rank 6: ['C', 'C1=NC2=CN=C1C2']

NN-rank 7: ['C', 'C1=NC2=CN=C1C2']

NN-rank 8: ['Cc1c[n+]2c(C)c-2n1']

NN-rank 9: ['Cc1c[n+]2c(C)c-2n1']

NN-rank 10: ['C', 'Cc1ncc2nc1-2']

NN-rank 11: ['C', 'Cc1ncc2nc1-2']

NN-rank 12: ['CC', 'c1cnccn1']

NN-rank 13: ['C', 'C', 'c1nc2cnc1-2']

NN-rank 14: ['C', 'Cc1cnccn1']

NN-rank 15: ['C', 'Cc1cnccn1']

NN-rank 16: ['CC1C2=CN=C1C=N2']

NN-rank 17: ['CC1C2=CN=C1C=N2']

NN-rank 18: ['CCc1cnccn1']

NN-rank 19: ['CCc1cnccn1']

NN-rank 20: ['CCc1ncc2nc1-2']

NN-rank 21: ['CCc1ncc2nc1-2']

NN-rank 22: ['CC', 'c1nc2cnc1-2']

NN-rank 23: ['Cc1cnc(C)cn1']

NN-rank 24: ['C', 'Cc1ncc2nc1-2']

NN-rank 25: ['C', 'Cc1ncc2nc1-2']

NN/MS matches corresponding with tox GHS classification highlight in Bold font.

21. 2,6-Dimethyl-5-heptenal: CC(CCC=C(C)C)C=O

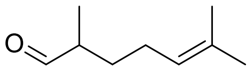

Summary Report of GHS Classification for NN/MS Matches:

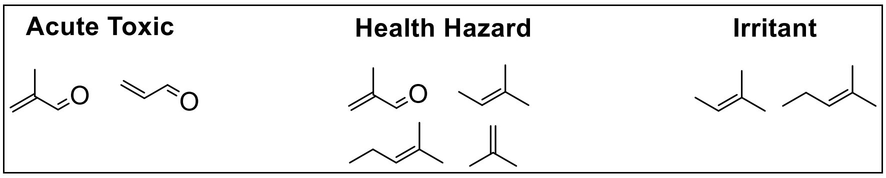

NN-rank 1: ['C=O', 'CC=CCC=C(C)C']

NN-rank 2: ['CC(C)=CCC=C(C)C=O']

NN-rank 3: ['C=O', 'C=CCCC=C(C)C']

NN-rank 4: ['C=C(C=O)CCC=C(C)C']

NN-rank 5: ['CC(C)=CCCC(C)CO']

NN-rank 6: ['C=O', 'CCCCC=C(C)C']

NN-rank 7: ['C=O', 'C=C=CCC=C(C)C']

NN-rank 8: ['C', 'CC(C)=CCCCC=O']

NN-rank 9: ['CC=CCC=C(C)C', 'CO']

NN-rank 10: ['CC(C)=CC=CC(C)C=O']

NN-rank 11: ['C', 'CC(C)=CCC=CC=O']

NN-rank 12: ['C=CCCC=C(C)C', 'CO']

NN-rank 13: ['C=C(C)C=O', 'CC=C(C)C']

EI-MS matched molecular weight (relative abundance: 32.43%) for C=C(C)C=O

GHS hazard classification: Acute Toxic, Health Hazard

EI-MS matched molecular weight (relative abundance: 32.43%) for CC=C(C)C

GHS hazard classification: Health Hazard, Irritant

NN-rank 14: ['CC(C)C=O', 'CC=C(C)C']

EI-MS matched molecular weight (relative abundance: 32.43%) for CC=C(C)C

GHS hazard classification: Health Hazard, Irritant

NN-rank 15: ['C', 'C=O', 'C=CCC=C(C)C']

NN-rank 16: ['CC(C)=C=CCC(C)C=O']

NN-rank 17: ['CCC=C(C)C', 'CCC=O']

EI-MS matched molecular weight (relative abundance: 12.51%) for CCC=C(C)C

NN/MS matches corresponding with tox GHS classification highlight in Bold font.

GHS hazard classification: Health Hazard, Irritant

NN-rank 18: ['C=O', 'C=CC', 'CC=C(C)C']

EI-MS matched molecular weight (relative abundance: 39.34%) for C=CC

GHS hazard classification: none of the hazards selected for correlation

EI-MS matched molecular weight (relative abundance: 32.43%) for CC=C(C)C

GHS hazard classification: Health Hazard, Irritant

NN-rank 19: ['C=O', 'CC=C=CC=C(C)C']

NN-rank 20: ['C', 'CC=CCCC(C)C=O']

NN-rank 21: ['C', 'CC=CCCC(C)C=O']

NN-rank 22: ['C=C(C)C', 'CCC(C)C=O']

EI-MS matched molecular weight (relative abundance: 20.82%) for C=C(C)C

GHS hazard classification: Health Hazard

NN-rank 23: ['CC(=C=CC=C(C)C)C=O']

NN-rank 24: ['C=CC=O', 'CCC=C(C)C']

EI-MS matched molecular weight (relative abundance: 20.82%) for C=CC=O

GHS hazard classification: Acute Toxic

EI-MS matched molecular weight (relative abundance: 12.51%) for CCC=C(C)C

GHS hazard classification: Health Hazard, Irritant

NN-rank 25: ['CC(C)=CCCC(C)C', 'O']

NN/MS matches corresponding with tox GHS classification highlight in Bold font.

22. 4-(4-Hydroxyphenyl)-2-butanone: CC(=O)CCC1=CC=C(C=C1)O

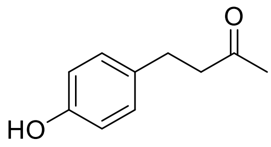

Summary Report of GHS Classification for NN/MS Matches:

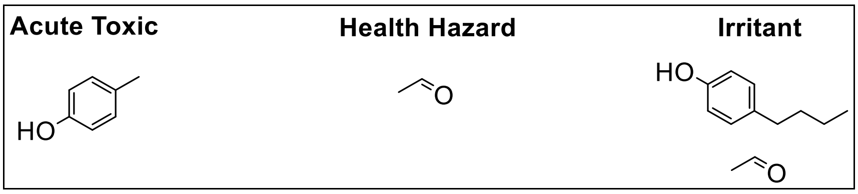

NN-rank 1: ['CCCCc1ccc(O)cc1', 'O']

EI-MS matched molecular weight (relative abundance: 5.65%) for CCCCc1ccc(O)cc1

GHS hazard classification: Irritant

NN-rank 2: ['CC(O)CCc1ccc(O)cc1']

NN-rank 3: ['CC(C)=O', 'Cc1ccc(O)cc1']

EI-MS matched molecular weight (relative abundance: 99.99%) for Cc1ccc(O)cc1

GHS hazard classification: Acute Toxic

NN-rank 4: ['CC(=O)C=Cc1ccc(O)cc1']

NN-rank 5: ['CC=CCc1ccc(O)cc1', 'O']

NN-rank 6: ['CC=O', 'CCc1ccc(O)cc1']

EI-MS matched molecular weight (relative abundance: 40.84%) for CC=O

GHS hazard classification: Health Hazard, Irritant

EI-MS matched molecular weight (relative abundance: 12.48%) for CCc1ccc(O)cc1

GHS hazard classification: none of the hazards selected for correlation

NN-rank 7: ['CCC(C)=O', 'Oc1ccccc1']

NN-rank 8: ['CC(=O)C#Cc1ccc(O)cc1']

NN-rank 9: ['CC(O)=CCc1ccc(O)cc1']

NN-rank 10: ['CC1CCc2ccc(O)cc21', 'O']

NN-rank 11: ['CC1CCc2ccc(O)cc21', 'O']

NN-rank 12: ['C=CCCc1ccc(O)cc1', 'O']

NN-rank 13: ['C=C(O)CCc1ccc(O)cc1']

NN-rank 14: ['CC1(O)CCc2ccc(O)cc21']

NN/MS matches corresponding with tox GHS classification highlight in Bold font.

NN-rank 15: ['CC1(O)CCc2ccc(O)cc21']

NN-rank 16: ['C', 'OCCCc1ccc(O)cc1']

NN-rank 17: ['C=Cc1ccc(O)cc1', 'CC=O']

EI-MS matched molecular weight (relative abundance: 40.84%) for CC=O

GHS hazard classification: Health Hazard, Irritant

NN-rank 18: ['CC1CCc2ccc(O)c1c2', 'O']

NN-rank 19: ['C=C1CCc2ccc(O)cc21', 'O']

NN-rank 20: ['C=C1CCc2ccc(O)cc21', 'O']

NN-rank 21: ['CC(=O)CCc1ccccc1', 'O']

NN-rank 22: ['CC1=CCc2ccc(O)cc21', 'O']

NN-rank 23: ['CC1=CCc2ccc(O)cc21', 'O']

NN-rank 24: ['CC1(O)CCc2ccc(O)c1c2']

NN-rank 25: ['CCC(O)Cc1ccc(O)cc1']

NN/MS matches corresponding with tox GHS classification highlight in Bold font.

23. 4-(4-Methoxyphenyl)butan-2-one: CC(=O)CCC1=CC=C(C=C1)OC

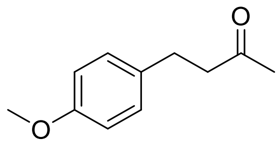

Summary Report of GHS Classification for NN/MS Matches:

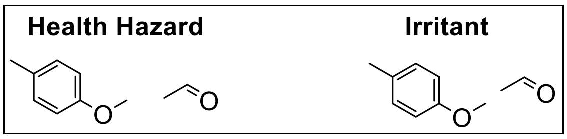

NN-rank 1: ['C', 'CC(=O)CCc1ccc(O)cc1']

NN-rank 2: ['COc1ccc(CCC(C)O)cc1']

NN-rank 3: ['CCCCc1ccc(OC)cc1', 'O']

NN-rank 4: ['CC(C)=O', 'COc1ccc(C)cc1']

EI-MS matched molecular weight (relative abundance: 99.99%) for COc1ccc(C)cc1

GHS hazard classification: Health Hazard, Irritant

NN-rank 5: ['COc1ccc(C=CC(C)=O)cc1']

NN-rank 6: ['COc1ccc(CCC2CO2)cc1']

NN-rank 7: ['CC=O', 'CCc1ccc(OC)cc1']

EI-MS matched molecular weight (relative abundance: 16.59%) for CC=O

GHS hazard classification: Health Hazard, Irritant

EI-MS matched molecular weight (relative abundance: 7.69%) for CCc1ccc(OC)cc1

GHS hazard classification: none of the hazards selected for correlation

NN-rank 8: ['C', 'COc1ccc(CCCO)cc1']

NN-rank 9: ['COc1ccc2c(c1)C(C)(O)CC2']

NN-rank 10: ['COc1ccc2c(c1)C(C)(O)CC2']

NN-rank 11: ['COc1ccc(CC=C(C)O)cc1']

NN-rank 12: ['CC(=O)CCc1ccccc1', 'CO']

NN-rank 13: ['CCC(C)=O', 'COc1ccccc1']

NN-rank 14: ['CC=CCc1ccc(OC)cc1', 'O']

NN-rank 15: ['COc1ccc(CC2OC2C)cc1']

NN-rank 16: ['COc1ccc(C#CC(C)=O)cc1']

NN-rank 17: ['COc1ccc(CCCCO)cc1']

NN/MS matches corresponding with tox GHS classification highlight in Bold font.

NN-rank 18: ['CC=O', 'C=Cc1ccc(OC)cc1']

EI-MS matched molecular weight (relative abundance: 16.59%) for CC=O

GHS hazard classification: Health Hazard, Irritant

NN-rank 19: ['C=C(O)CCc1ccc(OC)cc1']

NN-rank 20: ['COc1ccc2c(c1)C1(CC2)CO1']

NN-rank 21: ['COc1ccc2c(c1)C1(CC2)CO1']

NN-rank 22: ['COc1ccc2c(c1)C(C)CC2', 'O']

NN-rank 23: ['COc1ccc2c(c1)C(C)CC2', 'O']

NN-rank 24: ['CCO', 'CCc1ccc(OC)cc1']

EI-MS matched molecular weight (relative abundance: 7.69%) for CCc1ccc(OC)cc1

GHS hazard classification: none of the hazards selected for correlation

NN-rank 25: ['COc1ccc(C=C2OC2C)cc1']

NN/MS matches corresponding with tox GHS classification highlight in Bold font.

24. 4-Methyl-5-Thiazole Ethanol: CC1=C(SC=N1)CCO

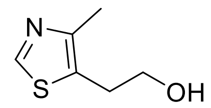

Summary Report of GHS Classification for NN/MS Matches:

Acute Toxic	Health Hazard	Irritant
−OH	−OH	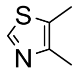

NN-rank 1: ['C=Cc1scnc1C', 'O']

NN-rank 2: ['CCc1scnc1C', 'O']

NN-rank 3: ['C#Cc1scnc1C', 'O']

NN-rank 4: ['Cc1ncsc1CC=O']

NN-rank 5: ['Cc1ncsc1C=CO']

NN-rank 6: ['CO', 'Cc1ncsc1C']

> **EI-MS matched molecular weight (relative abundance: 7.49%) for CO**
>
> **GHS hazard classification: Acute Toxic, Health Hazard**
>
> **EI-MS matched molecular weight (relative abundance: 99.99%) for Cc1ncsc1C**
>
> **GHS hazard classification: Irritant**

NN-rank 7: ['Cc1ncsc1C(C)O']

NN-rank 8: ['Cc1ncsc1C#CO']

NN-rank 9: ['Cc1ncsc1C1CO1']

NN-rank 10: ['C', 'OCCc1cncs1']

NN-rank 11: ['c1nc2c(s1)CCC2', 'O']

NN-rank 12: ['C=C(O)c1scnc1C']

NN-rank 13: ['C', 'Cc1ncsc1C', 'O']

> **EI-MS matched molecular weight (relative abundance: 99.99%) for Cc1ncsc1C**
>
> **GHS hazard classification: Irritant**

NN-rank 14: ['c1nc2c(s1)CCOC2']

NN-rank 15: ['CCc1scnc1CO']

NN-rank 16: ['Cc1ncsc1C1=CO1']

NN-rank 17: ['OC1Cc2ncsc2C1']

NN-rank 18: ['C=O', 'Cc1ncsc1C']

NN/MS matches corresponding with tox GHS classification highlight in Bold font.

EI-MS matched molecular weight (relative abundance: 99.99%) for Cc1ncsc1C

GHS hazard classification: Irritant

NN-rank 19: ['Cc1ncsc1C=C=O']

NN-rank 20: ['C1=Cc2scnc2C1', 'O']

NN-rank 21: ['COCc1scnc1C']

NN-rank 22: ['C', 'c1nc2c(s1)CCO2']

NN-rank 23: ['C', 'Cc1ncsc1CO']

NN-rank 24: ['C=Cc1scnc1CO']

NN-rank 25: ['C', 'CCc1scnc1O']

NN/MS matches corresponding with tox GHS classification highlight in Bold font.

25. 4-Terpinenol: CC1=CCC(CC1)(C(C)C)O

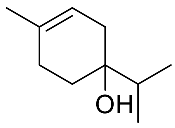

Summary Report of GHS Classification for NN/MS Matches:

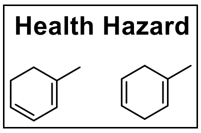

NN-rank 1: ['CC1=CC=C(C(C)C)CC1', 'O']

NN-rank 2: ['CC1=CCC(C(C)C)=CC1', 'O']

NN-rank 3: ['CC1C=C=C(C(C)C)CC1', 'O']

NN-rank 4: ['CC(C)C1=CCC(C)(O)CC1']

NN-rank 5: ['CC1=C=C=C(C(C)C)CC1', 'O']

NN-rank 6: ['C', 'CC(C)C1=C=CCCC1', 'O']

NN-rank 7: ['CC(C)C1=CCC(C)(O)CC1']

NN-rank 8: ['CC1=CCC(C(C)C)=C=C1', 'O']

NN-rank 9: ['CC(C)C1=CCC(C)(O)C=C1']

NN-rank 10: ['CC1=CC=C=CC1', 'CCC', 'O']

EI-MS matched molecular weight (relative abundance: 7.69%) for CC1=CC=C=CC1

GHS hazard classification: none of the hazards selected for correlation

EI-MS matched molecular weight (relative abundance: 29.09%) for CCC

GHS hazard classification: none of the hazards selected for correlation

NN-rank 11: ['CC(C)C1=C=CC(C)(O)CC1']

NN-rank 12: ['CC1C=CC(O)(C(C)C)CC1']

NN-rank 13: ['CC1=CCC(C(C)C)=C=C1', 'O']

NN-rank 14: ['C=C1C=C=C(C(C)C)CC1', 'O']

NN-rank 15: ['C=C(CC=C(C)C)C(C)C', 'O']

NN-rank 16: ['C=C(C)CCC(=C)C(C)C', 'O']

NN-rank 17: ['CC1=CC=CCC1', 'CCC', 'O']

EI-MS matched molecular weight (relative abundance: 43.49%) for CC1=CC=CCC1

GHS hazard classification: Health Hazard

EI-MS matched molecular weight (relative abundance: 29.09%) for CCC

NN/MS matches corresponding with tox GHS classification highlight in Bold font.

GHS hazard classification: none of the hazards selected for correlation

NN-rank 18: ['CC1CCC(O)(C(C)C)CC1']

NN-rank 19: ['CC1=CCC(C(C)C)CC1', 'O']

NN-rank 20: ['CC1=CCC=CC1', 'CCC', 'O']

EI-MS matched molecular weight (relative abundance: 43.49%) for CC1=CCC=CC1

GHS hazard classification: Irritant

EI-MS matched molecular weight (relative abundance: 29.09%) for CCC

GHS hazard classification: none of the hazards selected for correlation

NN-rank 21: ['C', 'CC(C)C1=CCC(O)CC1']

NN-rank 22: ['CC1=CCC(=C(C)C)CC1', 'O']

NN-rank 23: ['CC1C=C=CCC1', 'CCC', 'O']

EI-MS matched molecular weight (relative abundance: 43.49%) for CC1C=C=CCC1

GHS hazard classification: none of the hazards selected for correlation

EI-MS matched molecular weight (relative abundance: 29.09%) for CCC

GHS hazard classification: none of the hazards selected for correlation

NN-rank 24: ['CC(C)C12C=CC(C)(CC1)O2']

NN-rank 25: ['C', 'CC(C)C1=CC=C(O)CC1']

NN/MS matches corresponding with tox GHS classification highlight in Bold font.

26. 4,5-Dimethyl-3-Hydroxy-2,5-Dihydrofuran-2-One: CC1C(=C(C(=O)O1)O)C

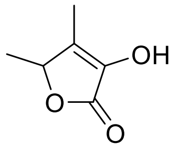

NN-rank 1: ['CC(=C(O)CO)C(C)O']

NN-rank 2: ['CCC(C)=C(O)C(=O)O']

EI-MS matched molecular weight (relative abundance: 17.36%) for CCC(C)=C(O)C(=O)O

GHS hazard classification: none of the hazards selected for correlation

NN-rank 3: ['CC(=C(O)C=O)C(C)O']

EI-MS matched molecular weight (relative abundance: 17.36%) for CC(=C(O)C=O)C(C)O

GHS hazard classification: unknown

NN-rank 4: ['CC1=C(O)COC1C', 'O']

EI-MS matched molecular weight (relative abundance: 10.18%) for CC1=C(O)COC1C

GHS hazard classification: none of the hazards selected for correlation

NN-rank 5: ['C=O', 'CC(=CO)C(C)O']

NN-rank 6: ['C', 'CC1C=C(O)C(=O)O1']

EI-MS matched molecular weight (relative abundance: 10.18%) for CC1C=C(O)C(=O)O1

GHS hazard classification: none of the hazards selected for correlation

NN-rank 7: ['CC=C(O)C(=O)OCC']

EI-MS matched molecular weight (relative abundance: 17.36%) for CC=C(O)C(=O)OCC

GHS hazard classification: none of the hazards selected for correlation

NN-rank 8: ['CC1=C(O)C(O)OC1C']

EI-MS matched molecular weight (relative abundance: 17.36%) for CC1=C(O)C(O)OC1C

GHS hazard classification: none of the hazards selected for correlation

NN-rank 9: ['CCC(C)=C(O)C(=O)O']

EI-MS matched molecular weight (relative abundance: 17.36%) for CCC(C)=C(O)C(=O)O

NN/MS matches corresponding with tox GHS classification highlight in Bold font.

GHS hazard classification: none of the hazards selected for correlation

NN-rank 10: ['CC1=CC(=O)OC1C', 'O']

NN-rank 11: ['CCC(C)=C(O)CO', 'O']

NN-rank 12: ['CC', 'CC=C(O)C(=O)O']

NN-rank 13: ['CC1OC(=O)C(O)C1C']

EI-MS matched molecular weight (relative abundance: 17.36%) for CC1OC(=O)C(O)C1C

GHS hazard classification: none of the hazards selected for correlation

NN-rank 14: ['CC(=O)C(C)=C(O)CO']

EI-MS matched molecular weight (relative abundance: 17.36%) for CC(=O)C(C)=C(O)CO

GHS hazard classification: unknown

NN-rank 15: ['C', 'CC1=C(O)C(=O)OC1']

EI-MS matched molecular weight (relative abundance: 10.18%) for CC1=C(O)C(=O)OC1

GHS hazard classification: none of the hazards selected for correlation

NN-rank 16: ['CC(=O)C(C)=C(O)C=O']

NN-rank 17: ['CCC(C)=C(O)C=O', 'O']

EI-MS matched molecular weight (relative abundance: 10.18%) for CCC(C)=C(O)C=O

GHS hazard classification: none of the hazards selected for correlation

NN-rank 18: ['C', 'CC1CC(O)C(=O)O1']

NN-rank 19: ['C', 'C=C(O)C(=O)OCC']

NN-rank 20: ['CCC(C)OC(=O)CO']

NN-rank 21: ['CCOC(=O)C(O)CC']

NN-rank 22: ['C=O', 'CC(O)C(C)CO']

NN-rank 23: ['CC(=CO)C(C)OC=O']

EI-MS matched molecular weight (relative abundance: 17.36%) for CC(=CO)C(C)OC=O

GHS hazard classification: unknown

NN-rank 24: ['CC(O)=C(C)C(C)O', 'O']

NN-rank 25: ['C=O', 'CC(=O)C(C)=CO']

NN/MS matches corresponding with tox GHS classification highlight in Bold font.

27. 5-methyl-2-Phenyl-2-Hexenal: CC(C)C/C=C(/C=O)\C1=CC=CC=C1

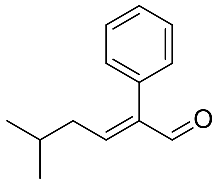

Summary Report of GHS Classification for NN/MS Matches:

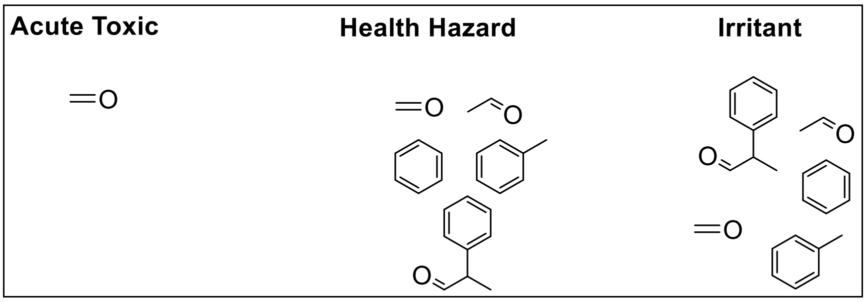

NN-rank 1: ['C=O', 'CC(C)CCCc1ccccc1']

EI-MS matched molecular weight (relative abundance: 8.29%) for C=O

GHS hazard classification: Acute Toxic, Health Hazard, Irritant

NN-rank 2: ['CC(C)CC=CC=O', 'c1ccccc1']

EI-MS matched molecular weight (relative abundance: 16.8%) for c1ccccc1

GHS hazard classification: Health Hazard, Irritant

NN-rank 3: ['CC(C)CCC(C=O)c1ccccc1']

NN-rank 4: ['CCC(C)C', 'O=CCc1ccccc1']

NN-rank 5: ['C=O', 'CC(C)C/C=C\\c1ccccc1']

EI-MS matched molecular weight (relative abundance: 8.29%) for C=O

GHS hazard classification: Acute Toxic, Health Hazard, Irritant

NN-rank 6: ['CC(C)C/C=C(/CO)c1ccccc1']

NN-rank 7: ['C', 'CCC/C=C(/C=O)c1ccccc1']

EI-MS matched molecular weight (relative abundance: 17.67%) for CCC/C=C(/C=O)c1ccccc1

GHS hazard classification: none of the hazards selected for correlation

NN-rank 8: ['C', 'CCC/C=C(/C=O)c1ccccc1']

EI-MS matched molecular weight (relative abundance: 17.67%) for CCC/C=C(/C=O)c1ccccc1

NN/MS matches corresponding with tox GHS classification highlight in Bold font.

GHS hazard classification: none of the hazards selected for correlation

NN-rank 9: ['CC(C)CCCC=O', 'c1ccccc1']

EI-MS matched molecular weight (relative abundance: 16.8%) for c1ccccc1

GHS hazard classification: Health Hazard, Irritant

NN-rank 10: ['C=O', 'CC(C)C=CCc1ccccc1']

EI-MS matched molecular weight (relative abundance: 8.29%) for C=O

GHS hazard classification: Acute Toxic, Health Hazard, Irritant

NN-rank 11: ['CC(C)C=CC(C=O)c1ccccc1']

NN-rank 12: ['CC=O', 'CCC(C)C', 'c1ccccc1']

EI-MS matched molecular weight (relative abundance: 29.3%) for CC=O

GHS hazard classification: Health Hazard, Irritant

EI-MS matched molecular weight (relative abundance: 16.8%) for c1ccccc1

GHS hazard classification: Health Hazard, Irritant

NN-rank 13: ['CC(C)CCC(=C=O)c1ccccc1']

NN-rank 14: ['C=O', 'CCC(C)C', 'Cc1ccccc1']

EI-MS matched molecular weight (relative abundance: 8.29%) for C=O

GHS hazard classification: Acute Toxic, Health Hazard, Irritant

EI-MS matched molecular weight (relative abundance: 48.7%) for Cc1ccccc1

GHS hazard classification: Health Hazard, Irritant

NN-rank 15: ['CC(C)C=C=C(C=O)c1ccccc1']

NN-rank 16: ['CCC(C)C', 'O=C=Cc1ccccc1']

EI-MS matched molecular weight (relative abundance: 99.99%) for O=C=Cc1ccccc1

GHS hazard classification: none of the hazards selected for correlation

NN-rank 17: ['C=O', 'C=CCC(C)C', 'c1ccccc1']

EI-MS matched molecular weight (relative abundance: 8.29%) for C=O

GHS hazard classification: Acute Toxic, Health Hazard, Irritant

EI-MS matched molecular weight (relative abundance: 16.8%) for c1ccccc1

GHS hazard classification: Health Hazard, Irritant

NN-rank 18: ['CC(C)=C/C=C(/C=O)c1ccccc1']

NN-rank 19: ['C=CC(C)C', 'O=CCc1ccccc1']

NN-rank 20: ['C/C=C(/C=O)c1ccccc1', 'CCC']

EI-MS matched molecular weight (relative abundance: 28.23%) for C/C=C(/C=O)c1ccccc1

GHS hazard classification: Irritant

EI-MS matched molecular weight (relative abundance: 29.3%) for CCC

NN/MS matches corresponding with tox GHS classification highlight in Bold font.

GHS hazard classification: none of the hazards selected for correlation

NN-rank 21: ['C=C(C=O)c1ccccc1', 'CC(C)C']

EI-MS matched molecular weight (relative abundance: 14.86%) for C=C(C=O)c1ccccc1

GHS hazard classification: none of the hazards selected for correlation

NN-rank 22: ['C=O', 'CCCC(C)C', 'c1ccccc1']

EI-MS matched molecular weight (relative abundance: 8.29%) for C=O

GHS hazard classification: Acute Toxic, Health Hazard, Irritant

EI-MS matched molecular weight (relative abundance: 16.8%) for c1ccccc1

GHS hazard classification: Health Hazard, Irritant

NN-rank 23: ['CC(C)CCC(=CO)c1ccccc1']

NN-rank 24: ['CC(C)C', 'CC(C=O)c1ccccc1']

EI-MS matched molecular weight (relative abundance: 11.09%) for CC(C=O)c1ccccc1

GHS hazard classification: Health Hazard, Irritant

NN-rank 25: ['C', 'C', 'CC/C=C(/C=O)c1ccccc1']

NN/MS matches corresponding with tox GHS classification highlight in Bold font.

28. 5-Methyl Furfural: CC1=CC=C(O1)C=O

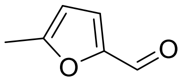

Summary Report of GHS Classification for NN/MS Matches:

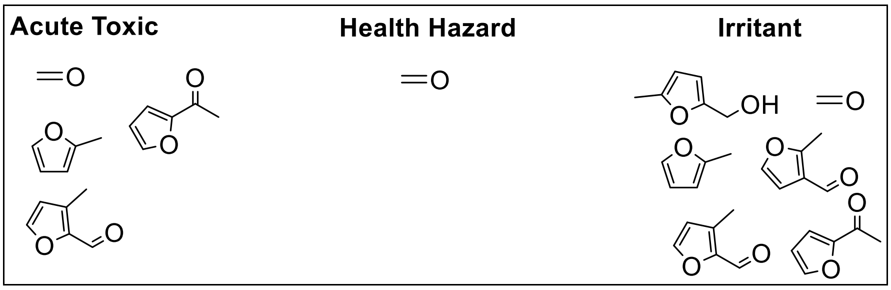

NN-rank 1: ['Cc1ccc(CO)o1']

EI-MS matched molecular weight (relative abundance: 6.99%) for Cc1ccc(CO)o1

GHS hazard classification: Irritant

NN-rank 2: ['C=O', 'Cc1ccco1']

EI-MS matched molecular weight (relative abundance: 5.89%) for C=O

GHS hazard classification: Acute Toxic, Health Hazard, Irritant

EI-MS matched molecular weight (relative abundance: 10.29%) for Cc1ccco1

GHS hazard classification: Acute Toxic, Irritant

NN-rank 3: ['C', 'O=Cc1ccco1']

NN-rank 4: ['c1cc2oc1COC2']

EI-MS matched molecular weight (relative abundance: 78.89%) for c1cc2oc1COC2

GHS hazard classification: unknown

NN-rank 5: ['CO', 'Cc1ccco1']

EI-MS matched molecular weight (relative abundance: 10.29%) for Cc1ccco1

GHS hazard classification: Acute Toxic, Irritant

NN-rank 6: ['Cc1oc2cc1C2=O']

NN-rank 7: ['OC1Cc2ccc1o2']

EI-MS matched molecular weight (relative abundance: 78.89%) for OC1Cc2ccc1o2

GHS hazard classification: unknown

NN-rank 8: ['O=C1Cc2ccc1o2']

NN-rank 9: ['Cc1oc2cc1C2O']

EI-MS matched molecular weight (relative abundance: 78.89%) for Cc1oc2cc1C2O

NN/MS matches corresponding with tox GHS classification highlight in Bold font.

GHS hazard classification: unknown

NN-rank 10: ['O=Cc1oc2cc1C2']

NN-rank 11: ['Cc1ccc(C)o1', 'O']

NN-rank 12: ['Oc1cc2ccc1o2']

NN-rank 13: ['O=Cc1cc2c(o1)C2']

NN-rank 14: ['Cc1oc2cc1OC2']

EI-MS matched molecular weight (relative abundance: 78.89%) for Cc1oc2cc1OC2

GHS hazard classification: unknown

NN-rank 15: ['Cc1cc2c(=O)c2o1']

NN-rank 16: ['Cc1occc1C=O']

EI-MS matched molecular weight (relative abundance: 78.89%) for Cc1occc1C=O

GHS hazard classification: Irritant

NN-rank 17: ['Cc1cc2c(o1)C2O']

EI-MS matched molecular weight (relative abundance: 78.89%) for Cc1cc2c(o1)C2O

GHS hazard classification: unknown

NN-rank 18: ['COCc1ccco1']

EI-MS matched molecular weight (relative abundance: 6.99%) for COCc1ccco1

GHS hazard classification: none of the hazards selected for correlation

NN-rank 19: ['Cc1ccoc1C=O']

EI-MS matched molecular weight (relative abundance: 78.89%) for Cc1ccoc1C=O

GHS hazard classification: Acute Toxic, Irritant

NN-rank 20: ['CC(=O)c1ccco1']

EI-MS matched molecular weight (relative abundance: 78.89%) for CC(=O)c1ccco1

GHS hazard classification: Acute Toxic, Irritant

NN-rank 21: ['c1cc2ccc1o2', 'O']

NN-rank 22: ['Cc1ccc(CO)o1']

EI-MS matched molecular weight (relative abundance: 6.99%) for Cc1ccc(CO)o1

GHS hazard classification: Irritant

NN-rank 23: ['COCc1ccco1']

EI-MS matched molecular weight (relative abundance: 6.99%) for COCc1ccco1

GHS hazard classification: none of the hazards selected for correlation

NN-rank 24: ['O=CCc1ccco1']

EI-MS matched molecular weight (relative abundance: 78.89%) for O=CCc1ccco1

GHS hazard classification: none of the hazards selected for correlation

NN-rank 25: ['O=C=Cc1ccco1']

NN/MS matches corresponding with tox GHS classification highlight in Bold font.

NN/MS matches corresponding with tox GHS classification highlight in Bold font.

29. 6-Methyl-5-Hepten-2-one: CC(=CCCC(=O)C)C

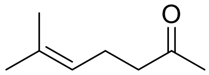

Summary Report of GHS Classification for NN/MS Matches:

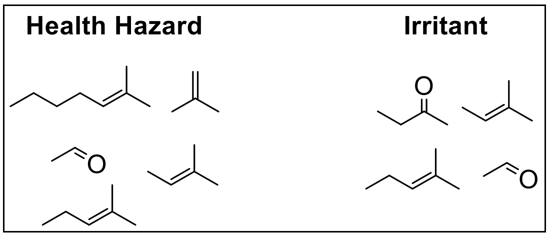

NN-rank 1: ['CC(C)=CCCC(C)O']

NN-rank 2: ['CCCCC=C(C)C', 'O']

EI-MS matched molecular weight (relative abundance: 16.51%) for CCCCC=C(C)C

GHS hazard classification: Health Hazard

NN-rank 3: ['CC(=O)CCCC(C)C']

NN-rank 4: ['CC=O', 'CCC=C(C)C']

EI-MS matched molecular weight (relative abundance: 99.99%) for CC=O

GHS hazard classification: Health Hazard, Irritant

EI-MS matched molecular weight (relative abundance: 8.91%) for CCC=C(C)C

GHS hazard classification: Health Hazard, Irritant

NN-rank 5: ['C', 'CCCCCC(C)=O']

NN-rank 6: ['C', 'CCCCCC(C)=O']

NN-rank 7: ['C', 'CC=CCCC(C)=O']

EI-MS matched molecular weight (relative abundance: 16.51%) for CC=CCCC(C)=O

GHS hazard classification: none of the hazards selected for correlation

NN-rank 8: ['C', 'CC=CCCC(C)=O']

EI-MS matched molecular weight (relative abundance: 16.51%) for CC=CCCC(C)=O

GHS hazard classification: none of the hazards selected for correlation

NN-rank 9: ['C=C(C)CCCC(C)=O']

NN-rank 10: ['C=C(C)CCCC(C)=O']

NN-rank 11: ['CC(C)=O', 'CC=C(C)C']

EI-MS matched molecular weight (relative abundance: 34.13%) for CC=C(C)C

GHS hazard classification: Health Hazard, Irritant

NN/MS matches corresponding with tox GHS classification highlight in Bold font.

NN-rank 12: ['C', 'C', 'CCCCC(C)=O']

NN-rank 13: ['CC(=O)CC=CC(C)C']

NN-rank 14: ['C', 'C', 'C=CCCC(C)=O']

NN-rank 15: ['C', 'CC(C)=CCCCO']

NN-rank 16: ['CC1CCCC(C)(C)O1']

NN-rank 17: ['C=C(C)C', 'CCC(C)=O']

EI-MS matched molecular weight (relative abundance: 33.03%) for C=C(C)C

GHS hazard classification: Health Hazard

EI-MS matched molecular weight (relative abundance: 12.91%) for CCC(C)=O

GHS hazard classification: Irritant

NN-rank 18: ['CC(C)=CCCC1CO1']

NN-rank 19: ['CCCCCC(C)(C)O']

NN-rank 20: ['CC(=O)CC=C=C(C)C']

NN-rank 21: ['C', 'CCC=CCC(C)=O']

EI-MS matched molecular weight (relative abundance: 16.51%) for CCC=CCC(C)=O

GHS hazard classification: none of the hazards selected for correlation

NN-rank 22: ['C', 'CCC=CCC(C)=O']

EI-MS matched molecular weight (relative abundance: 16.51%) for CCC=CCC(C)=O

GHS hazard classification: none of the hazards selected for correlation

NN-rank 23: ['CCC', 'CCCC(C)=O']

EI-MS matched molecular weight (relative abundance: 99.99%) for CCC

GHS hazard classification: none of the hazards selected for correlation

NN-rank 24: ['CCC=C(C)C', 'CCO']

EI-MS matched molecular weight (relative abundance: 8.91%) for CCC=C(C)C

GHS hazard classification: Health Hazard, Irritant

NN-rank 25: ['C', 'CC1=CCCC(C)O1']

EI-MS matched molecular weight (relative abundance: 16.51%) for CC1=CCCC(C)O1

GHS hazard classification: none of the hazards selected for correlation

NN/MS matches corresponding with tox GHS classification highlight in Bold font.

30. 6-Methyl Coumarin: CC1=CC2=C(C=C1)OC(=O)C=C2

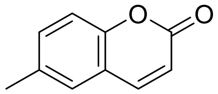

NN-rank 1: ['C', 'O=c1ccc2ccccc2o1']

NN-rank 2: ['O=c1ccc2c3c(ccc2o1)C3']

NN-rank 3: [No prediction]

NN-rank 4: [No prediction]

NN-rank 5: ['O=c1cc2c3cc(ccc3o1)C2']

NN-rank 6: ['C1=Cc2cc3ccc2OC1=C3', 'O']

NN-rank 7: ['Cc1cccc2oc(=O)ccc12']

NN-rank 8: ['O=c1ccc2cc3c(cc2o1)C3']

NN-rank 9: ['Cc1cc(=O)oc2ccccc12']

NN-rank 10: [No prediction]

NN-rank 11: [No prediction]

NN-rank 12: ['C=C1C=Cc2ccccc2O1', 'O']

NN-rank 13: ['Cc1ccc2oc(=O)c3cc2c13']

NN-rank 14: [No prediction]

NN-rank 15: [No prediction]

NN-rank 16: ['Cc1c2cc3oc(=O)ccc3c1-2']

NN-rank 17: ['Cc1ccc2ccc(=O)oc2c1']

NN-rank 18: ['Cc1ccc2oc(=O)ccc2c1']

NN-rank 19: ['C', 'O=c1ccc2ccccc2o1']

NN-rank 20: ['O', 'c1cc2c3c4ccc(c1o4)c23']

NN-rank 21: ['Cc1ccc2c3c1C(O)(C=C3)O2']

NN-rank 22: [No prediction]

NN-rank 23: ['O=c1cc2c3c4c(ccc3o1)C24']

NN-rank 24: [No prediction]

NN-rank 25: [No prediction]

31. Veratraldehyde: COC1=C(C=C(C=C1)C=O)OC

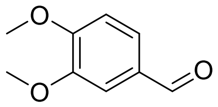

Summary Report of GHS Classification for NN/MS Matches:

Acute Toxic	Health Hazard	Irritant
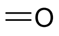	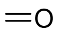	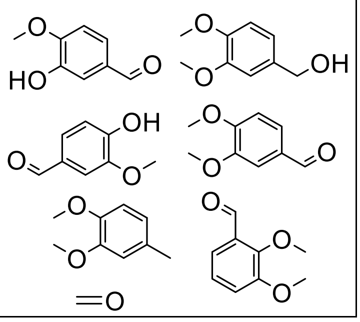

NN-rank 1: ['C', 'COc1ccc(C=O)cc1O']

EI-MS matched molecular weight (relative abundance: 5.71%) for C

GHS hazard classification: none of the hazards selected for correlation

EI-MS matched molecular weight (relative abundance: 12.01%) for COc1ccc(C=O)cc1O

GHS hazard classification: Irritant

NN-rank 2: ['C', 'COc1cc(C=O)ccc1O']

EI-MS matched molecular weight (relative abundance: 5.71%) for C

GHS hazard classification: none of the hazards selected for correlation

EI-MS matched molecular weight (relative abundance: 12.01%) for COc1cc(C=O)ccc1O

GHS hazard classification: Irritant

NN-rank 3: ['C=O', 'COc1ccccc1OC']

EI-MS matched molecular weight (relative abundance: 6.41%) for C=O

GHS hazard classification: Acute Toxic, Health Hazard, Irritant

NN-rank 4: ['COc1ccc(CO)cc1OC']

EI-MS matched molecular weight (relative abundance: 10.01%) for COc1ccc(CO)cc1OC

GHS hazard classification: Irritant

NN/MS matches corresponding with tox GHS classification highlight in Bold font.

NN-rank 5: ['CO', 'COc1ccc(C=O)cc1']

NN-rank 6: ['CO', 'COc1cccc(C=O)c1']

NN-rank 7: ['COc1ccc(C)cc1OC', 'O']

EI-MS matched molecular weight (relative abundance: 12.01%) for COc1ccc(C)cc1OC

GHS hazard classification: Irritant

NN-rank 8: ['CO', 'COc1ccccc1OC']

NN-rank 9: ['COc1c(C)cc(C=O)cc1O']

EI-MS matched molecular weight (relative abundance: 63.86%) for COc1c(C)cc(C=O)cc1O

GHS hazard classification: none of the hazards selected for correlation

NN-rank 10: ['COc1cc2cc(c1OC)C2=O']

NN-rank 11: ['COc1c2cc(C=O)cc1OC2']

NN-rank 12: ['COc1cc2cc(c1OC)C2O']

EI-MS matched molecular weight (relative abundance: 63.86%) for COc1cc2cc(c1OC)C2O

GHS hazard classification: unknown

NN-rank 13: ['COc1cc(C=O)cc2c1OC2']

NN-rank 14: ['COc1cc(C=O)cc(C)c1O']

EI-MS matched molecular weight (relative abundance: 63.86%) for COc1cc(C=O)cc(C)c1O

GHS hazard classification: none of the hazards selected for correlation

NN-rank 15: ['COc1cc2c(=O)c2cc1OC']

NN-rank 16: ['COc1cccc(C=O)c1OC']

EI-MS matched molecular weight (relative abundance: 63.86%) for COc1cccc(C=O)c1OC

GHS hazard classification: Irritant

NN-rank 17: ['COc1ccc2c(=O)c2c1OC']

NN-rank 18: ['COCc1ccc(OC)c(O)c1']

EI-MS matched molecular weight (relative abundance: 10.01%) for COCc1ccc(OC)c(O)c1

GHS hazard classification: none of the hazards selected for correlation

NN-rank 19: ['COc1cc2c(cc1OC)C2O']

EI-MS matched molecular weight (relative abundance: 63.86%) for COc1cc2c(cc1OC)C2O

NN/MS matches corresponding with tox GHS classification highlight in Bold font.

GHS hazard classification: unknown

NN-rank 20: ['COc1ccc2c(c1OC)C2O']

EI-MS matched molecular weight (relative abundance: 63.86%) for COc1ccc2c(c1OC)C2O

GHS hazard classification: unknown

NN-rank 21: ['COCc1ccc(O)c(OC)c1']

EI-MS matched molecular weight (relative abundance: 10.01%) for COCc1ccc(O)c(OC)c1

GHS hazard classification: none of the hazards selected for correlation

NN-rank 22: ['COc1cc2ccc1OCOC2']

EI-MS matched molecular weight (relative abundance: 63.86%) for COc1cc2ccc1OCOC2

GHS hazard classification: unknown

NN-rank 23: ['COc1ccc2cc1OCOC2']

EI-MS matched molecular weight (relative abundance: 63.86%) for COc1ccc2cc1OCOC2

GHS hazard classification: unknown

NN-rank 24: ['COc1cc2cc(c1OC)C2', 'O']

NN-rank 25: ['COc1ccc(C=O)cc1OC']

EI-MS matched molecular weight (relative abundance: 63.86%) for COc1ccc(C=O)cc1OC

GHS hazard classification: Irritant

NN/MS matches corresponding with tox GHS classification highlight in Bold font.

32. Vanillin Propylene Glycol Acetal: CC1COC(O1)C2=CC(=C(C=C2)O)OC

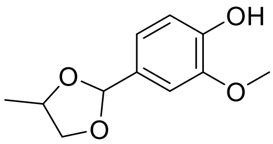

Summary Report of GHS Classification for NN/MS Matches:

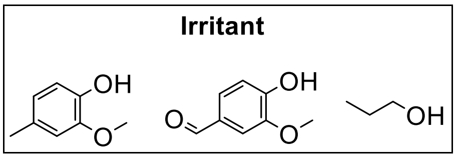

NN-rank 1: ['CC(O)CO', 'COc1cc(C)ccc1O']

EI-MS matched molecular weight (relative abundance: 26.0%) for COc1cc(C)ccc1O

GHS hazard classification: Irritant

NN-rank 2: ['COc1cc(COC(C)CO)ccc1O']

NN-rank 3: ['COc1cc(COCC(C)O)ccc1O']

NN-rank 4: ['CC(C)O', 'COc1cc(C=O)ccc1O']

EI-MS matched molecular weight (relative abundance: 23.0%) for CC(C)O

GHS hazard classification: none of the hazards selected for correlation

EI-MS matched molecular weight (relative abundance: 99.9%) for COc1cc(C=O)ccc1O

GHS hazard classification: Irritant

NN-rank 5: ['CCCO', 'COc1cc(C=O)ccc1O']

EI-MS matched molecular weight (relative abundance: 23.0%) for CCCO

GHS hazard classification: Irritant

EI-MS matched molecular weight (relative abundance: 99.9%) for COc1cc(C=O)ccc1O

GHS hazard classification: Irritant

NN-rank 6: ['C', 'CC1COC(c2ccc(O)c(O)c2)O1']

NN-rank 7: ['COc1cc(COC(C)C=O)ccc1O']

EI-MS matched molecular weight (relative abundance: 56.0%) for COc1cc(COC(C)C=O)ccc1O

GHS hazard classification: unknown

NN/MS matches corresponding with tox GHS classification highlight in Bold font.

NN-rank 8: ['CC(O)C=O', 'COc1cc(C)ccc1O']

EI-MS matched molecular weight (relative abundance: 26.0%) for COc1cc(C)ccc1O

GHS hazard classification: Irritant

NN-rank 9: ['CCCOC(O)c1ccc(O)c(OC)c1']

NN-rank 10: ['COc1cc(C(O)OC(C)C)ccc1O']

NN-rank 11: ['CC1COC(c2ccc(O)cc2)O1', 'CO']

NN-rank 12: ['CC(=O)CO', 'COc1cc(C)ccc1O']

EI-MS matched molecular weight (relative abundance: 26.0%) for COc1cc(C)ccc1O

GHS hazard classification: Irritant

NN-rank 13: ['CC1COCOc2cc(ccc2O)CO1']

EI-MS matched molecular weight (relative abundance: 56.0%) for CC1COCOc2cc(ccc2O)CO1

GHS hazard classification: unknown

NN-rank 14: ['CCCOC(=O)c1ccc(O)c(OC)c1']

EI-MS matched molecular weight (relative abundance: 56.0%) for CCCOC(=O)c1ccc(O)c(OC)c1

GHS hazard classification: none of the hazards selected for correlation

NN-rank 15: ['Cc1ccc(O)c(OCOCC(C)O)c1']

NN-rank 16: ['COc1cc(COC2(C)CO2)ccc1O']

EI-MS matched molecular weight (relative abundance: 56.0%) for COc1cc(COC2(C)CO2)ccc1O

GHS hazard classification: unknown

NN-rank 17: ['COc1cc(C(=O)OC(C)C)ccc1O']

EI-MS matched molecular weight (relative abundance: 56.0%) for COc1cc(C(=O)OC(C)C)ccc1O

GHS hazard classification: none of the hazards selected for correlation

NN-rank 18: ['COc1cc(COCC(C)=O)ccc1O']

EI-MS matched molecular weight (relative abundance: 56.0%) for COc1cc(COCC(C)=O)ccc1O

GHS hazard classification: unknown

NN-rank 19: ['CCCO', 'COc1cc(C)ccc1O', 'O']

EI-MS matched molecular weight (relative abundance: 23.0%) for CCCO

GHS hazard classification: Irritant

EI-MS matched molecular weight (relative abundance: 26.0%) for COc1cc(C)ccc1O

GHS hazard classification: Irritant

NN/MS matches corresponding with tox GHS classification highlight in Bold font.

NN-rank 20: ['CCC=O', 'COc1cc(C=O)ccc1O']

EI-MS matched molecular weight (relative abundance: 99.9%) for COc1cc(C=O)ccc1O

GHS hazard classification: Irritant

NN-rank 21: ['CC1CO1', 'COc1cc(CO)ccc1O']

NN-rank 22: ['C', 'COc1cc(C2OCCO2)ccc1O']

NN-rank 23: ['CC1(O)CO1', 'COc1cc(C)ccc1O']

EI-MS matched molecular weight (relative abundance: 26.0%) for COc1cc(C)ccc1O

GHS hazard classification: Irritant

NN-rank 24: ['CC(C)O', 'Cc1ccc(O)c(OCO)c1']

EI-MS matched molecular weight (relative abundance: 23.0%) for CC(C)O

GHS hazard classification: none of the hazards selected for correlation

NN-rank 25: ['CC1CO1', 'COc1cc(C=O)ccc1O']

EI-MS matched molecular weight (relative abundance: 99.9%) for COc1cc(C=O)ccc1O

GHS hazard classification: Irritant

NN/MS matches corresponding with tox GHS classification highlight in Bold font.

33. Acetic Acid: CC(=O)O

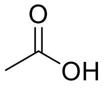

Summary Report of GHS Classification for NN/MS Matches:

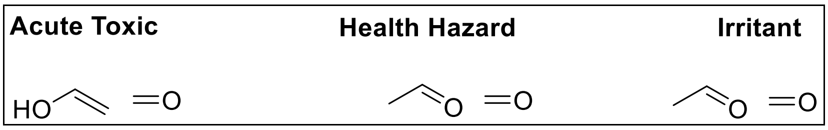

NN-rank 1: ['CC(=O)O']

NN-rank 2: ['C', 'O=CO']

EI-MS matched molecular weight (relative abundance: 17.09%) for C

GHS hazard classification: none of the hazards selected for correlation

EI-MS matched molecular weight (relative abundance: 90.39%) for O=CO

GHS hazard classification: none of the hazards selected for correlation

NN-rank 3: ['CC=O', 'O']

EI-MS matched molecular weight (relative abundance: 99.99%) for CC=O

GHS hazard classification: Health Hazard, Irritant

NN-rank 4: ['C', 'O=C=O']

EI-MS matched molecular weight (relative abundance: 17.09%) for C

GHS hazard classification: none of the hazards selected for correlation

EI-MS matched molecular weight (relative abundance: 13.09%) for O=C=O

GHS hazard classification: none of the hazards selected for correlation

NN-rank 5: ['C', 'O=CO']

EI-MS matched molecular weight (relative abundance: 17.09%) for C

GHS hazard classification: none of the hazards selected for correlation

EI-MS matched molecular weight (relative abundance: 90.39%) for O=CO

GHS hazard classification: none of the hazards selected for correlation

NN-rank 6: ['O=C1CO1']

NN-rank 7: ['CCO', 'O']

EI-MS matched molecular weight (relative abundance: 90.39%) for CCO

GHS hazard classification: none of the hazards selected for correlation

NN-rank 8: ['C', 'C=O', 'O']

EI-MS matched molecular weight (relative abundance: 17.09%) for C

NN/MS matches corresponding with tox GHS classification highlight in Bold font.

GHS hazard classification: none of the hazards selected for correlation

EI-MS matched molecular weight (relative abundance: 8.49%) for C=O

GHS hazard classification: Acute Toxic, Health Hazard, Irritant

NN-rank 9: ['C=C(O)O']

NN-rank 10: ['COC=O']

NN-rank 11: ['C=C=O', 'O']

NN-rank 12: ['O=C1CO1']

NN-rank 13: ['CCO', 'O']

EI-MS matched molecular weight (relative abundance: 90.39%) for CCO

GHS hazard classification: none of the hazards selected for correlation

NN-rank 14: ['CC(O)O']

NN-rank 15: ['COCO']

NN-rank 16: ['C=CO', 'O']

EI-MS matched molecular weight (relative abundance: 99.99%) for C=CO

GHS hazard classification: Acute Toxic

NN-rank 17: ['OC1CO1']

NN-rank 18: ['CC=O', 'O']

EI-MS matched molecular weight (relative abundance: 99.99%) for CC=O

GHS hazard classification: Health Hazard, Irritant

NN-rank 19: ['O=CC=O']

NN-rank 20: ['COC=O']

NN-rank 21: ['C=CO', 'O']

EI-MS matched molecular weight (relative abundance: 99.99%) for C=CO

GHS hazard classification: Acute Toxic

NN-rank 22: ['C', 'C=O', 'O']

EI-MS matched molecular weight (relative abundance: 17.09%) for C

GHS hazard classification: none of the hazards selected for correlation

EI-MS matched molecular weight (relative abundance: 8.49%) for C=O

GHS hazard classification: Acute Toxic, Health Hazard, Irritant

NN-rank 23: ['C', 'OCO']

EI-MS matched molecular weight (relative abundance: 17.09%) for C

GHS hazard classification: none of the hazards selected for correlation

NN-rank 24: ['C=C=O', 'O']

NN-rank 25: ['COCO']

NN/MS matches corresponding with tox GHS classification highlight in Bold font.

NN/MS matches corresponding with tox GHS classification highlight in Bold font.

34. Acetoin: CC(C(=O)C)O

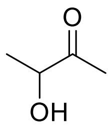

NN-rank 1: ['CC(=O)C(C)=O']

NN-rank 2: ['CC(O)C(C)O']

NN-rank 3: ['CC=O', 'CCO']

EI-MS matched molecular weight (relative abundance: 56.8%) for CC=O

GHS hazard classification: Health Hazard, Irritant

EI-MS matched molecular weight (relative abundance: 99.99%) for CCO

GHS hazard classification: none of the hazards selected for correlation

NN-rank 4: ['CC=O', 'CC=O']

EI-MS matched molecular weight (relative abundance: 56.8%) for CC=O

GHS hazard classification: Health Hazard, Irritant

NN-rank 5: ['C', 'CC(O)CO']

NN-rank 6: ['CCC(C)O', 'O']

NN-rank 7: ['C', 'CC(=O)C=O']

NN-rank 8: ['C', 'CC(O)C=O']

NN-rank 9: ['C', 'CC(=O)CO']

NN-rank 10: ['CC1OCC1=O']

NN-rank 11: ['C=C(O)C(C)O']

NN-rank 12: ['C', 'CC1OC1=O']

NN-rank 13: ['C=C(O)C(C)=O']

NN-rank 14: ['CCO', 'CCO']

EI-MS matched molecular weight (relative abundance: 99.99%) for CCO

GHS hazard classification: none of the hazards selected for correlation

NN-rank 15: ['CC1OC1(C)O']

NN-rank 16: ['CC=O', 'CCO']

EI-MS matched molecular weight (relative abundance: 56.8%) for CC=O

GHS hazard classification: Health Hazard, Irritant

EI-MS matched molecular weight (relative abundance: 99.99%) for CCO

GHS hazard classification: none of the hazards selected for correlation

NN-rank 17: ['C=CC(C)O', 'O']

NN/MS matches corresponding with tox GHS classification highlight in Bold font.

NN-rank 18: ['C', 'CC(C)O', 'O']

NN-rank 19: ['CC1OC1C', 'O']

NN-rank 20: ['COC(C)C=O']

NN-rank 21: ['C', 'C=O', 'CCO']

EI-MS matched molecular weight (relative abundance: 99.99%) for CCO

GHS hazard classification: none of the hazards selected for correlation

EI-MS matched molecular weight (relative abundance: 5.07%) for C=O

GHS hazard classification: Acute Toxic, Health Hazard, Irritant

NN-rank 22: ['C', 'C=O', 'CC=O']

EI-MS matched molecular weight (relative abundance: 5.07%) for C=O

GHS hazard classification: Acute Toxic, Health Hazard, Irritant

EI-MS matched molecular weight (relative abundance: 56.8%) for CC=O

GHS hazard classification: Health Hazard, Irritant

NN-rank 23: ['CCC(C)=O', 'O']

NN-rank 24: ['CC1(O)CC1O']

NN-rank 25: ['CC1OCC1O']

NN/MS matches corresponding with tox GHS classification highlight in Bold font.

35. Allyl Hexanoate: CCCCCC(=O)OCC=C

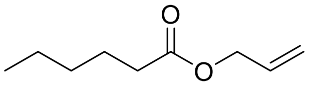

Summary Report of GHS Classification for NN/MS Matches:

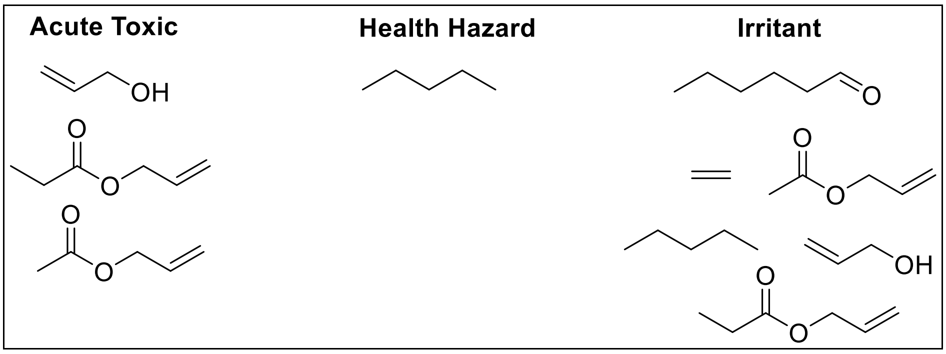

NN-rank 1: ['C=CC', 'CCCCCC(=O)O']

EI-MS matched molecular weight (relative abundance: 87.59%) for C=CC

GHS hazard classification: none of the hazards selected for correlation

NN-rank 2: ['C=CC', 'CCCCCC(=O)O']

EI-MS matched molecular weight (relative abundance: 87.59%) for C=CC

GHS hazard classification: none of the hazards selected for correlation

NN-rank 3: ['C=C=C', 'CCCCCC(=O)O']

EI-MS matched molecular weight (relative abundance: 31.29%) for C=C=C

GHS hazard classification: none of the hazards selected for correlation

NN-rank 4: ['C=CCO', 'CCCCCCO']

EI-MS matched molecular weight (relative abundance: 10.29%) for C=CCO

GHS hazard classification: Acute Toxic, Irritant

NN-rank 5: ['C=CC', 'CCCCCC=O', 'O']

EI-MS matched molecular weight (relative abundance: 87.59%) for C=CC

GHS hazard classification: none of the hazards selected for correlation

EI-MS matched molecular weight (relative abundance: 99.99%) for CCCCCC=O

GHS hazard classification: Irritant

NN-rank 6: ['C', 'C=CCOC(=O)CCCC']

NN-rank 7: ['C=CCO', 'CCCCCC=O']

EI-MS matched molecular weight (relative abundance: 10.29%) for C=CCO

NN/MS matches corresponding with tox GHS classification highlight in Bold font.

GHS hazard classification: Acute Toxic, Irritant

EI-MS matched molecular weight (relative abundance: 99.99%) for CCCCCC=O

GHS hazard classification: Irritant

NN-rank 8: ['C=CC', 'CCCCCC(=O)O']

EI-MS matched molecular weight (relative abundance: 87.59%) for C=CC

GHS hazard classification: none of the hazards selected for correlation

NN-rank 9: ['C=C=C', 'CCCCCC(=O)O']

EI-MS matched molecular weight (relative abundance: 31.29%) for C=C=C

GHS hazard classification: none of the hazards selected for correlation

NN-rank 10: ['C=CC', 'CCCCCC(=O)O']

EI-MS matched molecular weight (relative abundance: 87.59%) for C=CC

GHS hazard classification: none of the hazards selected for correlation

NN-rank 11: ['CC', 'C=CCOC(=O)CCC']

EI-MS matched molecular weight (relative abundance: 24.19%) for CC

GHS hazard classification: none of the hazards selected for correlation

NN-rank 12: ['C=CCOCCCCCC', 'O']

NN-rank 13: ['C=C', 'CCCCCC(=O)OC']

EI-MS matched molecular weight (relative abundance: 23.69%) for C=C

GHS hazard classification: Irritant

NN-rank 14: ['CCC', 'C=CCOC(=O)CC']

EI-MS matched molecular weight (relative abundance: 97.69%) for CCC

GHS hazard classification: none of the hazards selected for correlation

EI-MS matched molecular weight (relative abundance: 10.59%) for C=CCOC(=O)CC

GHS hazard classification: Acute Toxic, Irritant

NN-rank 15: ['C=C=COC(=O)CCCCC']

NN-rank 16: ['C=CCC(=O)CCCCC', 'O']

NN-rank 17: ['CCCCCC(=O)OCCC']

NN-rank 18: ['C=CCOC(C)=O', 'CCCC']

EI-MS matched molecular weight (relative abundance: 99.99%) for C=CCOC(C)=O

GHS hazard classification: Acute Toxic, Irritant

EI-MS matched molecular weight (relative abundance: 10.29%) for CCCC

GHS hazard classification: none of the hazards selected for correlation

NN-rank 19: ['C=CCOC=O', 'CCCCC']

EI-MS matched molecular weight (relative abundance: 61.09%) for CCCCC

GHS hazard classification: Health Hazard, Irritant

NN/MS matches corresponding with tox GHS classification highlight in Bold font.

NN-rank 20: ['C=CC', 'CCCCC', 'O=CO']

EI-MS matched molecular weight (relative abundance: 87.59%) for C=CC

GHS hazard classification: none of the hazards selected for correlation

EI-MS matched molecular weight (relative abundance: 61.09%) for CCCCC

GHS hazard classification: Health Hazard, Irritant

NN-rank 21: ['C', 'C=C', 'CCCCCC(=O)O']

EI-MS matched molecular weight (relative abundance: 23.69%) for C=C

GHS hazard classification: Irritant

NN-rank 22: ['CCCCC1C=CCOC1=O']

NN-rank 23: ['C=C=C', 'CCCCCC=O', 'O']

EI-MS matched molecular weight (relative abundance: 31.29%) for C=C=C

GHS hazard classification: none of the hazards selected for correlation

EI-MS matched molecular weight (relative abundance: 99.99%) for CCCCCC=O

GHS hazard classification: Irritant

NN-rank 24: ['CC=COC(=O)CCCCC']

NN-rank 25: ['C', 'C=C', 'CCCCCC(=O)O']

EI-MS matched molecular weight (relative abundance: 23.69%) for C=C

GHS hazard classification: Irritant

NN/MS matches corresponding with tox GHS classification highlight in Bold font.

36. alpha-Damascenone: C/C=C/C(=O)C1C(=CC=CC1(C)C)C

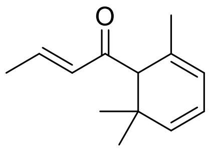

NN-rank 1: ['CCCC(=O)C1C(C)=CC=CC1(C)C']

NN-rank 2: ['CC1CC(=O)C2C(=CC=CC2(C)C)C1']

NN-rank 3: ['C/C=C/C(=O)C1=C(C)CC=CC1(C)C']

NN-rank 4: ['CC1=CC(=O)C2C(=CC=CC2(C)C)C1']

NN-rank 5: ['C/C=C/C(=O)C1C(C)=CCCC1(C)C']

NN-rank 6: ['CC1=CC=CC(C)(C)C12C(=O)CC2C']

NN-rank 7: ['C/C=C/C(O)C1C(C)=CC=CC1(C)C']

NN-rank 8: ['C/C=C/C(=O)C1C(C)CC=CC1(C)C']

NN-rank 9: ['CC1=CC=CC(C)(C)C12C(=O)C=C2C']

NN-rank 10: ['C/C=C/C(=O)C1C(C)=C=C=CC1(C)C']

NN-rank 11: ['C=CCC(=O)C1C(C)=CC=CC1(C)C']

NN-rank 12: ['CC1=C2C=CC(C)(C)C1C(=O)CC2C']

NN-rank 13: ['CC1=CC=C2CC=CC(=O)C1C2(C)C']

NN-rank 14: ['C', 'CCC(=O)C1C(C)=CC=CC1(C)C']

NN-rank 15: ['C', 'C=CC(=O)C1C(C)=CC=CC1(C)C']

EI-MS matched molecular weight (relative abundance: 6.5%) for C=CC(=O)C1C(C)=CC=CC1(C)C

GHS hazard classification: unknown

NN-rank 16: ['CC1=CC(=O)C2C(C)=C1C=CC2(C)C']

NN-rank 17: ['CC1=CC2=CC(C)(C)C1C(=O)CC2C']

NN-rank 18: ['C', 'C/C=C/C(=O)C1C(C)=CC=CC1C']

EI-MS matched molecular weight (relative abundance: 6.5%) for C/C=C/C(=O)C1C(C)=CC=CC1C

GHS hazard classification: unknown

NN-rank 19: ['C=C=CC(=O)C1C(C)=CC=CC1(C)C']

NN-rank 20: ['CC1=CC(=O)C2C(C)=CC1=CC2(C)C']

NN-rank 21: ['C', 'CC1(C)C=CC=C2CC=CC(=O)C21']

NN-rank 22: ['C', 'CC1=CC=CC(C)(C)C12C=CC2=O']

NN/MS matches corresponding with tox GHS classification highlight in Bold font.

NN-rank 23: ['C=CC(=O)C1C(C)=CC=C(C)C1(C)C']

NN-rank 24: ['C=C1CC(=O)C2C(=CC=CC2(C)C)C1']

NN-rank 25: ['CC1=CCC2CC=CC(=O)C1C2(C)C']

NN/MS matches corresponding with tox GHS classification highlight in Bold font.

37. alpha-Damascone: C/C=C/C(=O)C1C(=CCCC1(C)C)C

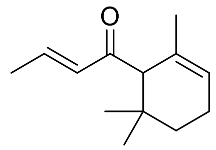

NN-rank 1: ['CCCC(=O)C1C(C)=CCCC1(C)C']

NN-rank 2: ['C/C=C/C(=O)C1C(C)C=CCC1(C)C']

NN-rank 3: ['CC1=C2CCC(C)(C)C1C(=O)CC2C']

NN-rank 4: ['CC1CC(=O)C2C(C)C1CCC2(C)C']

NN-rank 5: ['C/C=C/C(=O)C1C(C)CCCC1(C)C']

NN-rank 6: ['C/C=C/C(=O)C1C(C)=C=CCC1(C)C']

NN-rank 7: ['CC1=CCCC(C)(C)C12C(=O)CC2C']

NN-rank 8: ['CC1CC(=O)C2C(=CCCC2(C)C)C1']

NN-rank 9: ['C/C=C/C(=O)C1=C(C)C=CCC1(C)C']

NN-rank 10: ['C=C1C=CCC(C)(C)C1C(=O)/C=C/C']

NN-rank 11: ['CC1=CC2CC(C)(C)C1C(=O)CC2C']

NN-rank 12: ['CC1CC(=O)C2C(C)C1=CCC2(C)C']

NN-rank 13: ['C=C1CCCC(C)(C)C1C(=O)/C=C/C']

NN-rank 14: ['C/C=C/C(=O)C1=C(C)CCCC1(C)C']

NN-rank 15: ['CC1=CC(=O)C2C(C)=C1CCC2(C)C']

NN-rank 16: ['C/C=C/C(O)C1C(C)=CCCC1(C)C']

NN-rank 17: ['C/C=C/C(=O)C1C(C)=CC=CC1(C)C']

NN-rank 18: ['C=C1C2CCC(C)(C)C1C(=O)CC2C']

NN-rank 19: ['CC1=C2C(=O)CC(C)C1CCC2(C)C']

NN-rank 20: ['CC1=CC(=O)C2C(C)C1CCC2(C)C']

NN-rank 21: ['CC1=CCCC(C)(C)C12C(=O)C=C2C']

NN-rank 22: ['C', 'C/C=C/C(=O)C1CCCCC1(C)C']

NN-rank 23: ['CC1=CC(=O)C2C(C)C1=CCC2(C)C']

NN-rank 24: ['CC1=CC(=O)C2C(=CCCC2(C)C)C1']

NN-rank 25: ['CC1CC(=O)C2C(C)C=C1CC2(C)C']

NN/MS matches corresponding with tox GHS classification highlight in Bold font.

38. Alpha-Ionone: CC1=CCCC(C1/C=C/C(=O)C)(C)C

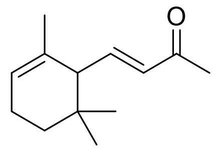

NN-rank 1: ['CC1=CCCC(C)(C)C1/C=C/C(C)O']

NN-rank 2: ['C=C1CCCC(C)(C)C1/C=C/C(C)=O']

NN-rank 3: ['CC(=O)/C=C/C1=C(C)CCCC1(C)C']

NN-rank 4: ['C=C1C=CCC(C)(C)C1/C=C/C(C)=O']

NN-rank 5: ['CC(=O)/C=C/C1C(C)CCCC1(C)C']

NN-rank 6: ['CC(=O)CCC1C(C)=CCCC1(C)C']

NN-rank 7: ['CC(=O)C=C=C1C(C)=CCCC1(C)C']

NN-rank 8: ['CC(=O)/C=C/C1C(C)=C=CCC1(C)C']

NN-rank 9: ['CC(=O)/C=C/C1C(C)C=CCC1(C)C']

NN-rank 10: ['CC(=O)CC=C1C(C)=CCCC1(C)C']

NN-rank 11: ['CC(=O)/C=C/C1=C(C)C=CCC1(C)C']

NN-rank 12: ['CC(=O)/C=C/C1C(C)=CC=CC1(C)C']

NN-rank 13: ['C=C(O)/C=C/C1C(C)=CCCC1(C)C']

NN-rank 14: ['C', 'CC1=CCCC(C)(C)C1/C=C/CO']

NN-rank 15: ['CC1=CCCC(C)(C)C1C1=CC(=O)C1']

NN-rank 16: ['CC(=O)/C=C/C=C(C)CCCC(C)C']

NN-rank 17: ['CC(=O)C=C=CC(C)=CCCC(C)C']

NN-rank 18: ['CC(=O)/C=C/CC(C)=CCCC(C)C']

NN-rank 19: ['C', 'CC(=O)/C=C/C1=C=CCCC1(C)C']

NN-rank 20: ['CC1=CCCC(C)(C)C1C1CC(=O)C1']

NN-rank 21: ['C=C(C)C(/C=C/C(C)=O)C(C)(C)CC']

NN-rank 22: ['C', 'CC(=O)/C=C/C1CCCCC1(C)C']

NN-rank 23: ['CCC(C)(C)C(/C=C/C(C)=O)C(C)C']

NN-rank 24: ['CCC=C(C)C(/C=C/C(C)=O)C(C)C']

NN-rank 25: ['CCC(C)(C)C(/C=C/C(C)=O)=C(C)C']

NN/MS matches corresponding with tox GHS classification highlight in Bold font.

39. alpha-Methylbenzyl acetate: CC(C1=CC=CC=C1)OC(=O)C

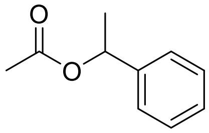

Summary Report of GHS Classification for NN/MS Matches:

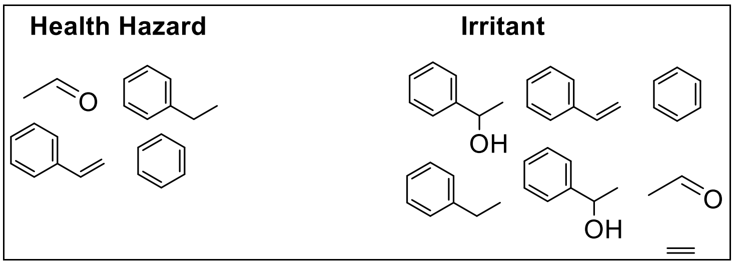

NN-rank 1: ['C=Cc1ccccc1', 'CC(=O)O']

EI-MS matched molecular weight (relative abundance: 28.92%) for C=Cc1ccccc1

GHS hazard classification: Health Hazard, Irritant

NN-rank 2: ['C=C(OC(C)=O)c1ccccc1']

NN-rank 3: ['CC(O)c1ccccc1', 'CC=O']

EI-MS matched molecular weight (relative abundance: 8.36%) for CC(O)c1ccccc1

GHS hazard classification: Irritant

EI-MS matched molecular weight (relative abundance: 76.02%) for CC=O

GHS hazard classification: Health Hazard, Irritant

NN-rank 4: ['CC(=O)O', 'CCc1ccccc1']

EI-MS matched molecular weight (relative abundance: 66.1%) for CCc1ccccc1

GHS hazard classification: Health Hazard, Irritant

NN-rank 5: ['C', 'CC(=O)OCc1ccccc1']

NN-rank 6: ['C=COC(C)=O', 'c1ccccc1']

EI-MS matched molecular weight (relative abundance: 40.69%) for c1ccccc1

GHS hazard classification: Health Hazard, Irritant

NN-rank 7: ['C=C1c2ccccc21', 'CC(=O)O']

NN-rank 8: ['C=C1c2ccccc21', 'CC(=O)O']

NN-rank 9: ['CCOC(C)=O', 'c1ccccc1']

EI-MS matched molecular weight (relative abundance: 40.69%) for c1ccccc1

GHS hazard classification: Health Hazard, Irritant

NN/MS matches corresponding with tox GHS classification highlight in Bold font.

NN-rank 10: ['C=C1C2=CC=C1C=C2', 'CC(=O)O']

NN-rank 11: ['CC(=O)O', 'CC1c2ccccc21']

EI-MS matched molecular weight (relative abundance: 28.92%) for CC1c2ccccc21

GHS hazard classification: none of the hazards selected for correlation

NN-rank 12: ['CC(=O)O', 'CC1c2ccccc21']

EI-MS matched molecular weight (relative abundance: 28.92%) for CC1c2ccccc21

GHS hazard classification: none of the hazards selected for correlation

NN-rank 13: ['C=C', 'CC(=O)O', 'c1ccccc1']

EI-MS matched molecular weight (relative abundance: 6.5%) for C=C

GHS hazard classification: Irritant

EI-MS matched molecular weight (relative abundance: 40.69%) for c1ccccc1

GHS hazard classification: Health Hazard, Irritant

NN-rank 14: ['C=C=O', 'CC(O)c1ccccc1']

EI-MS matched molecular weight (relative abundance: 8.36%) for CC(O)c1ccccc1

GHS hazard classification: Irritant

NN-rank 15: ['CC1(c2ccccc2)CC(=O)O1']

NN-rank 16: ['C', 'CC(=O)O', 'Cc1ccccc1']

NN-rank 17: ['CC(=O)O', 'CC1C2=CC=C1C=C2']

EI-MS matched molecular weight (relative abundance: 28.92%) for CC1C2=CC=C1C=C2

GHS hazard classification: none of the hazards selected for correlation

NN-rank 18: ['C', 'CC(OC=O)c1ccccc1']

NN-rank 19: ['CC(=O)c1ccccc1', 'CC=O']

EI-MS matched molecular weight (relative abundance: 76.02%) for CC=O

GHS hazard classification: Health Hazard, Irritant

NN-rank 20: ['CC(=O)OC1(C)c2ccccc21']

NN-rank 21: ['CC(=O)OC1(C)c2ccccc21']

NN-rank 22: ['C', 'C=O', 'CC(O)c1ccccc1']

EI-MS matched molecular weight (relative abundance: 8.36%) for CC(O)c1ccccc1

GHS hazard classification: Irritant

NN-rank 23: ['C=C(CC(=O)O)c1ccccc1']

NN-rank 24: ['CC(=O)OC1(C)C2=CC=C1C=C2']

NN-rank 25: ['CC(=CC(=O)O)c1ccccc1']

NN/MS matches corresponding with tox GHS classification highlight in Bold font.

40. alpha-Pinene: CC1=CCC2CC1C2(C)C

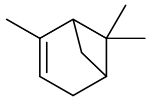

NN-rank 1: ['C=C1C=CC2CC1C2(C)C']

NN-rank 2: ['CC1C=CC2CC1C2(C)C']

NN-rank 3: ['C=C1CCC2CC1C2(C)C']

NN-rank 4: ['CC1=C2CC(C=C1)C2(C)C']

NN-rank 5: ['CC1CCC2CC1C2(C)C']

NN-rank 6: ['CC1=C2CC(CC1)C2(C)C']

NN-rank 7: ['C', 'CC1(C)C2C=CCC1C2']

EI-MS matched molecular weight (relative abundance: 6.78%) for C

GHS hazard classification: none of the hazards selected for correlation

EI-MS matched molecular weight (relative abundance: 11.57%) for CC1(C)C2C=CCC1C2

GHS hazard classification: none of the hazards selected for correlation

NN-rank 8: ['CC1C=C=C2CC1C2(C)C']

NN-rank 9: ['C=C1C=C=C2CC1C2(C)C']

NN-rank 10: ['C', 'CC1(C)C2CCCC1C2']

EI-MS matched molecular weight (relative abundance: 6.78%) for C

GHS hazard classification: none of the hazards selected for correlation

NN-rank 11: ['CC1=C2CC(=C=C1)C2(C)C']

NN-rank 12: ['CC1=C=CC2CC1C2(C)C']

NN-rank 13: ['C', 'CC1(C)C2=CCCC1C2']

EI-MS matched molecular weight (relative abundance: 6.78%) for C

GHS hazard classification: none of the hazards selected for correlation

EI-MS matched molecular weight (relative abundance: 11.57%) for CC1(C)C2=CCCC1C2

GHS hazard classification: none of the hazards selected for correlation

NN-rank 14: ['C', 'CC1(C)C2=CC=CC1C2']

EI-MS matched molecular weight (relative abundance: 6.78%) for C

GHS hazard classification: none of the hazards selected for correlation

NN-rank 15: ['CC12C=C1C1CC2C1(C)C']

NN/MS matches corresponding with tox GHS classification highlight in Bold font.

NN-rank 16: ['C=CC(C)C1CCC1(C)C']

NN-rank 17: ['C=CC(=C)C1CCC1(C)C']

NN-rank 18: ['CC(C)C1CC(C)C1(C)C']

NN-rank 19: ['CC1(C)C2CC1C1(C)CC21']

NN-rank 20: ['C=C(C)C1CC(C)C1(C)C']

NN-rank 21: ['C=CC(C)=C1CCC1(C)C']

NN-rank 22: ['C=C1C2=CC3C2C1C3(C)C']

NN-rank 23: ['CC1C2=CC3C2C1C3(C)C']

NN-rank 24: ['C=C1C2CC3C2C1C3(C)C']

NN-rank 25: ['CC1(C)C2C=CC3(C)C2C13']

NN/MS matches corresponding with tox GHS classification highlight in Bold font.

41. alpha-Terpineol: CC1=CCC(CC1)C(C)(C)O

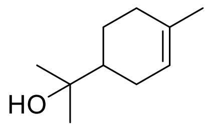

Summary Report of GHS Classification for NN/MS Matches:

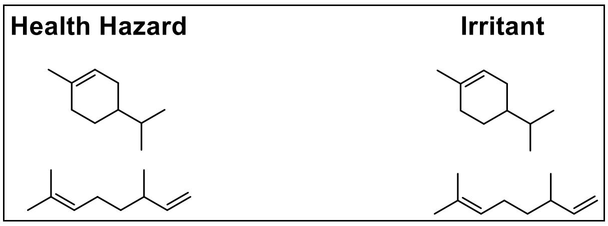

NN-rank 1: ['CC1CCC(C(C)(C)O)CC1']

NN-rank 2: ['C=C(C)C1CC=C(C)CC1', 'O']

NN-rank 3: ['C=C(C)C1CC=C(C)CC1', 'O']

NN-rank 4: ['CC12CCC(CC1)C(C)(C)O2']

NN-rank 5: ['CC12C=CC(CC1)C(C)(C)O2']

NN-rank 6: ['CC1=CCC(=C(C)C)CC1', 'O']

NN-rank 7: ['C=C(C)C1CCC(C)(O)CC1']

NN-rank 8: ['C=C(C)C1CCC(C)(O)CC1']

NN-rank 9: ['C', 'CC(C)(O)C1CCCCC1']

NN-rank 10: ['CC1=CCC(C(C)(C)O)CC1']

NN-rank 11: ['CC1C=CC(C(C)(C)O)CC1']

NN-rank 12: ['C=CC(C)CCC=C(C)C', 'O']

EI-MS matched molecular weight (relative abundance: 5.06%) for C=CC(C)CCC=C(C)C

GHS hazard classification: Health Hazard, Irritant

NN-rank 13: ['CC(C)C1CCC(C)(O)CC1']

NN-rank 14: ['CC1=CCC(C(C)C)CC1', 'O']

EI-MS matched molecular weight (relative abundance: 5.06%) for CC1=CCC(C(C)C)CC1

GHS hazard classification: Health Hazard, Irritant

NN-rank 15: ['CC(C)C1C=CC(C)(O)CC1']

NN-rank 16: ['C=C(C)C1CCC2(C)OC2C1']

NN-rank 17: ['C=C(C)C1CCC2(C)OC2C1']

NN/MS matches corresponding with tox GHS classification highlight in Bold font.

NN-rank 18: ['C=C(C)C1C=CC(C)(O)CC1']

NN-rank 19: ['C=C(C)C1C=CC(C)(O)CC1']

NN-rank 20: ['CC(C)C1CCC2(C)OC2C1']

NN-rank 21: ['C', 'CC1(C)OC2CCC1CC2']

EI-MS matched molecular weight (relative abundance: 6.53%) for CC1(C)OC2CCC1CC2

GHS hazard classification: none of the hazards selected for correlation

NN-rank 22: ['CC1CCC2CC1OC2(C)C']

NN-rank 23: ['C=C1CCC(C(C)(C)O)CC1']

NN-rank 24: ['CC(C)=C1CCC(C)(O)CC1']

NN-rank 25: ['C', 'CC(C)(O)C1CC=CCC1']

EI-MS matched molecular weight (relative abundance: 6.53%) for CC(C)(O)C1CC=CCC1

GHS hazard classification: none of the hazards selected for correlation

NN/MS matches corresponding with tox GHS classification highlight in Bold font.

42. Amyl acetate: CCCCCOC(=O)C

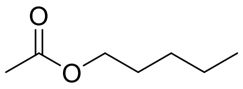

Summary Report of GHS Classification for NN/MS Matches:

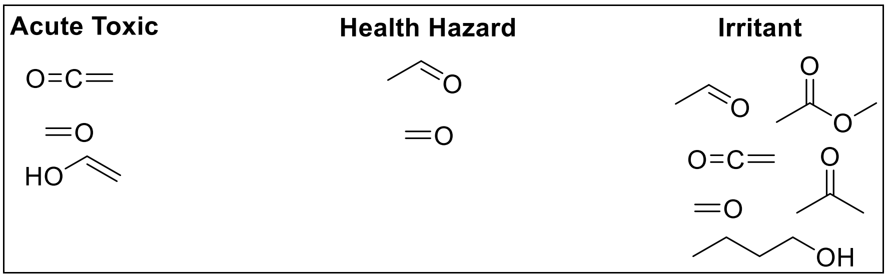

NN-rank 1: ['CC=O', 'CCCCCO']

EI-MS matched molecular weight (relative abundance: 99.99%) for CC=O

GHS hazard classification: Health Hazard, Irritant

NN-rank 2: ['CC(=O)O', 'CCCCC']

NN-rank 3: ['CCCCCO', 'CCO']

NN-rank 4: ['CC=O', 'CCCCC=O']

EI-MS matched molecular weight (relative abundance: 99.99%) for CC=O

GHS hazard classification: Health Hazard, Irritant

NN-rank 5: ['C', 'CCCCOC(C)=O']

NN-rank 6: ['C=C=O', 'CCCCCO']

EI-MS matched molecular weight (relative abundance: 23.19%) for C=C=O

GHS hazard classification: Acute Toxic, Irritant

NN-rank 7: ['CC(=O)CCCCCO']

NN-rank 8: ['CCCC', 'COC(C)=O']

EI-MS matched molecular weight (relative abundance: 5.99%) for CCCC

GHS hazard classification: none of the hazards selected for correlation

EI-MS matched molecular weight (relative abundance: 13.49%) for COC(C)=O

GHS hazard classification: Irritant

NN-rank 9: ['C1CCOCC1', 'CC=O']

EI-MS matched molecular weight (relative abundance: 99.99%) for CC=O

GHS hazard classification: Health Hazard, Irritant

NN-rank 10: ['CCC', 'CCOC(C)=O']

NN/MS matches corresponding with tox GHS classification highlight in Bold font.

EI-MS matched molecular weight (relative abundance: 99.99%) for CCC

GHS hazard classification: none of the hazards selected for correlation

NN-rank 11: ['CC(C)=O', 'CCCCO']

EI-MS matched molecular weight (relative abundance: 5.99%) for CC(C)=O

GHS hazard classification: Irritant

EI-MS matched molecular weight (relative abundance: 13.49%) for CCCCO

GHS hazard classification: Irritant

NN-rank 12: ['CC(=O)O', 'C=CCCC']

EI-MS matched molecular weight (relative abundance: 8.39%) for C=CCCC

GHS hazard classification: none of the hazards selected for correlation

NN-rank 13: ['CC', 'CCCOC(C)=O']

EI-MS matched molecular weight (relative abundance: 14.29%) for CC

GHS hazard classification: none of the hazards selected for correlation

NN-rank 14: ['CC=O', 'CCCCCO']

EI-MS matched molecular weight (relative abundance: 99.99%) for CC=O

GHS hazard classification: Health Hazard, Irritant

NN-rank 15: ['CCCCCOCC=O']

NN-rank 16: ['CCCCCOC(C)O']

NN-rank 17: ['CC=O', 'CCCCOC']

EI-MS matched molecular weight (relative abundance: 99.99%) for CC=O

GHS hazard classification: Health Hazard, Irritant

NN-rank 18: ['CCCC=COC(C)=O']

NN-rank 19: ['C', 'C=O', 'CCCCCO']

EI-MS matched molecular weight (relative abundance: 14.29%) for C=O

GHS hazard classification: Acute Toxic, Health Hazard, Irritant

NN-rank 20: ['C', 'CCCCCOC=O']

NN-rank 21: ['CCCCC', 'O=CCO']

NN-rank 22: ['C=CO', 'CCCCCO']

EI-MS matched molecular weight (relative abundance: 99.99%) for C=CO

GHS hazard classification: Acute Toxic

NN-rank 23: ['CCCCCOC(C)=O']

NN-rank 24: ['CCCCC=O', 'CCO']

NN-rank 25: ['CCOCCCCCO']

NN/MS matches corresponding with tox GHS classification highlight in Bold font.

43. Amyl Butyrate: CCCCCOC(=O)CCC

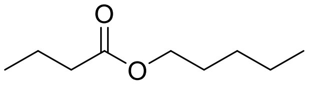

Summary Report of GHS Classification for NN/MS Matches:

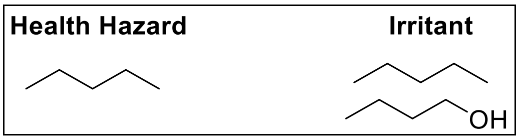

NN-rank 1: ['CCCC(=O)O', 'CCCCC']

EI-MS matched molecular weight (relative abundance: 91.09%) for CCCCC

GHS hazard classification: Health Hazard, Irritant

NN-rank 2: ['CCCC(=O)O', 'CCCCC']

EI-MS matched molecular weight (relative abundance: 91.09%) for CCCCC

GHS hazard classification: Health Hazard, Irritant

NN-rank 3: ['CCCCCO', 'CCCCO']

EI-MS matched molecular weight (relative abundance: 5.79%) for CCCCO

GHS hazard classification: Irritant

NN-rank 4: ['CCCC=O', 'CCCCCO']

EI-MS matched molecular weight (relative abundance: 91.09%) for CCCC=O

GHS hazard classification: none of the hazards selected for correlation

NN-rank 5: ['CCCC=O', 'CCCCC', 'O']

EI-MS matched molecular weight (relative abundance: 91.09%) for CCCC=O

GHS hazard classification: none of the hazards selected for correlation

EI-MS matched molecular weight (relative abundance: 91.09%) for CCCCC

GHS hazard classification: Health Hazard, Irritant

NN-rank 6: ['CCCC', 'CCCC(=O)OC']

NN-rank 7: ['C', 'CCCCOC(=O)CCC']

NN-rank 8: ['C', 'CCCCCOC(=O)CC']

NN-rank 9: ['C', 'CCCC', 'CCCC(=O)O']

NN-rank 10: ['CC1CCCCCOC(=O)C1']

NN-rank 11: ['CCCCOC(=O)CC(C)C']

NN-rank 12: ['CCCCCOCCCC', 'O']

NN-rank 13: ['CCC', 'CCCC(=O)OCC']

EI-MS matched molecular weight (relative abundance: 99.99%) for CCC

NN/MS matches corresponding with tox GHS classification highlight in Bold font.

GHS hazard classification: none of the hazards selected for correlation

NN-rank 14: ['CCCCOC(=O)CCCC']

NN-rank 15: ['O=C1CCCCCCCCO1']

NN-rank 16: ['C', 'CCCC', 'CCCC(=O)O']

NN-rank 17: ['CCCCCCOC(=O)CC']

NN-rank 18: ['CCCC', 'CCCCCO', 'O']

NN-rank 19: ['CCCCOC(=O)C(C)CC']

NN-rank 20: ['CC1CCCOC(=O)CCC1']

NN-rank 21: ['CC', 'CCCOC(=O)CCC']

EI-MS matched molecular weight (relative abundance: 15.19%) for CC

GHS hazard classification: none of the hazards selected for correlation

NN-rank 22: ['CCC(=O)OCCCC(C)C']

NN-rank 23: ['CCC1CCCCCOC1=O']

NN-rank 24: ['C', 'O=C1CCCCCCCO1']

NN-rank 25: ['CC', 'CCCCCOC(C)=O']

EI-MS matched molecular weight (relative abundance: 15.19%) for CC

GHS hazard classification: none of the hazards selected for correlation

NN/MS matches corresponding with tox GHS classification highlight in Bold font.

44. Anethole: C/C=C/C1=CC=C(C=C1)OC

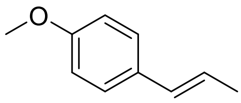

Summary Report of GHS Classification for NN/MS Matches:

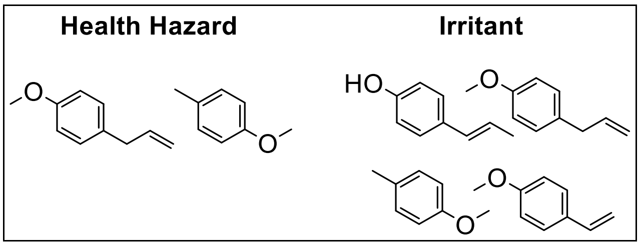

NN-rank 1: ['C', 'C/C=C/c1ccc(O)cc1']

EI-MS matched molecular weight (relative abundance: 24.7%) for C/C=C/c1ccc(O)cc1

GHS hazard classification: Irritant

NN-rank 2: ['CCCc1ccc(OC)cc1']

EI-MS matched molecular weight (relative abundance: 11.2%) for CCCc1ccc(OC)cc1

GHS hazard classification: none of the hazards selected for correlation

NN-rank 3: ['C=CCc1ccc(OC)cc1']

EI-MS matched molecular weight (relative abundance: 53.59%) for C=CCc1ccc(OC)cc1

GHS hazard classification: Health Hazard, Irritant

NN-rank 4: ['C', 'CCc1ccc(OC)cc1']

NN-rank 5: ['C#CCc1ccc(OC)cc1']

NN-rank 6: ['COc1ccc(C2CC2)cc1']

EI-MS matched molecular weight (relative abundance: 53.59%) for COc1ccc(C2CC2)cc1

GHS hazard classification: none of the hazards selected for correlation

NN-rank 7: ['C', 'CC1Cc2ccc(cc2)O1']

EI-MS matched molecular weight (relative abundance: 24.7%) for CC1Cc2ccc(cc2)O1

GHS hazard classification: unknown

NN-rank 8: ['CC1COc2ccc(cc2)C1']

EI-MS matched molecular weight (relative abundance: 53.59%) for CC1COc2ccc(cc2)C1

NN/MS matches corresponding with tox GHS classification highlight in Bold font.

GHS hazard classification: unknown

NN-rank 9: ['C', 'C=Cc1ccc(OC)cc1']

EI-MS matched molecular weight (relative abundance: 24.7%) for C=Cc1ccc(OC)cc1

GHS hazard classification: Irritant

NN-rank 10: ['CC(C)=Cc1ccc(O)cc1']

EI-MS matched molecular weight (relative abundance: 53.59%) for CC(C)=Cc1ccc(O)cc1

GHS hazard classification: none of the hazards selected for correlation

NN-rank 11: ['COc1ccc(C2C#C2)cc1']

NN-rank 12: ['C/C=C/c1ccccc1', 'CO']

EI-MS matched molecular weight (relative abundance: 32.6%) for C/C=C/c1ccccc1

GHS hazard classification: none of the hazards selected for correlation

NN-rank 13: ['COc1ccc(C2=CC2)cc1']

NN-rank 14: ['COc1ccc(C2C=C2)cc1']

NN-rank 15: ['CCC1COc2ccc1cc2']

EI-MS matched molecular weight (relative abundance: 53.59%) for CCC1COc2ccc1cc2

GHS hazard classification: unknown

NN-rank 16: ['CC(C)Cc1ccc(O)cc1']

EI-MS matched molecular weight (relative abundance: 11.2%) for CC(C)Cc1ccc(O)cc1

GHS hazard classification: none of the hazards selected for correlation

NN-rank 17: ['C/C=C(\\C)c1ccc(O)cc1']

EI-MS matched molecular weight (relative abundance: 53.59%) for C/C=C(\C)c1ccc(O)cc1

GHS hazard classification: none of the hazards selected for correlation

NN-rank 18: ['C', 'CC1=Cc2ccc(cc2)O1']

EI-MS matched molecular weight (relative abundance: 5.3%) for CC1=Cc2ccc(cc2)O1

GHS hazard classification: unknown

NN-rank 19: ['C/C1=C/c2ccc(cc2)OC1']

NN-rank 20: ['C', 'C1=Cc2ccc(cc2)OC1']

EI-MS matched molecular weight (relative abundance: 5.3%) for C1=Cc2ccc(cc2)OC1

GHS hazard classification: none of the hazards selected for correlation

NN-rank 21: ['C=C=Cc1ccc(OC)cc1']

NN-rank 22: ['C', 'c1cc2ccc1OC1CC21']

EI-MS matched molecular weight (relative abundance: 5.3%) for c1cc2ccc1OC1CC21

NN/MS matches corresponding with tox GHS classification highlight in Bold font.

GHS hazard classification: unknown

NN-rank 23: ['CC', 'COc1ccc(C)cc1']

EI-MS matched molecular weight (relative abundance: 18.2%) for COc1ccc(C)cc1

GHS hazard classification: Health Hazard, Irritant

NN-rank 24: ['COc1ccc2cc1C(C)C2']

EI-MS matched molecular weight (relative abundance: 53.59%) for COc1ccc2cc1C(C)C2

GHS hazard classification: unknown

NN-rank 25: ['COc1ccc2cc1C(C)C2']

EI-MS matched molecular weight (relative abundance: 53.59%) for COc1ccc2cc1C(C)C2

GHS hazard classification: unknown

NN/MS matches corresponding with tox GHS classification highlight in Bold font.

45. Anisaldehyde: COC1=CC=C(C=C1)C=O

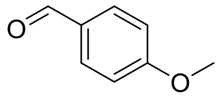

Summary Report of GHS Classification for NN/MS Matches:

Irritant

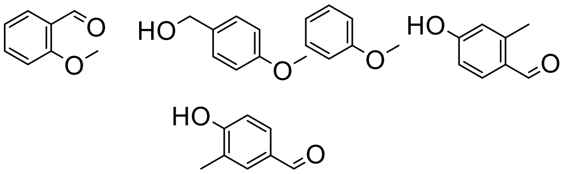

NN-rank 1: ['C', 'O=Cc1ccc(O)cc1']

NN-rank 2: ['C=O', 'COc1ccccc1']

EI-MS matched molecular weight (relative abundance: 17.38%) for COc1ccccc1

GHS hazard classification: Irritant

NN-rank 3: ['COc1ccc(CO)cc1']

EI-MS matched molecular weight (relative abundance: 5.66%) for COc1ccc(CO)cc1

GHS hazard classification: Irritant

NN-rank 4: ['CO', 'O=Cc1ccccc1']

NN-rank 5: ['C', 'O=Cc1ccc2c(c1)O2']

NN-rank 6: ['C', 'O=Cc1ccc2c(c1)O2']

NN-rank 7: ['COc1ccc(C)cc1', 'O']

NN-rank 8: ['Cc1cc(C=O)ccc1O']

EI-MS matched molecular weight (relative abundance: 99.99%) for Cc1cc(C=O)ccc1O

GHS hazard classification: Irritant

NN-rank 9: ['Cc1cc(C=O)ccc1O']

EI-MS matched molecular weight (relative abundance: 99.99%) for Cc1cc(C=O)ccc1O

GHS hazard classification: Irritant

NN-rank 10: ['O=Cc1ccc2c(c1)CO2']

NN-rank 11: ['O=Cc1ccc2c(c1)CO2']

NN-rank 12: ['CO', 'COc1ccccc1']

EI-MS matched molecular weight (relative abundance: 17.38%) for COc1ccccc1

GHS hazard classification: Irritant

NN/MS matches corresponding with tox GHS classification highlight in Bold font.

NN-rank 13: ['Cc1cc(O)ccc1C=O']

EI-MS matched molecular weight (relative abundance: 99.99%) for Cc1cc(O)ccc1C=O

GHS hazard classification: Irritant

NN-rank 14: ['Cc1cc(O)ccc1C=O']

EI-MS matched molecular weight (relative abundance: 99.99%) for Cc1cc(O)ccc1C=O

GHS hazard classification: Irritant

NN-rank 15: ['COc1ccc2cc1C2=O']

NN-rank 16: ['COc1ccc2cc1C2=O']

NN-rank 17: ['O=Cc1ccc2cc1CO2']

NN-rank 18: ['O=Cc1ccc2cc1CO2']

NN-rank 19: ['COc1ccc2cc1C2O']

EI-MS matched molecular weight (relative abundance: 99.99%) for COc1ccc2cc1C2O

GHS hazard classification: unknown

NN-rank 20: ['COc1ccc2cc1C2O']

EI-MS matched molecular weight (relative abundance: 99.99%) for COc1ccc2cc1C2O

GHS hazard classification: unknown

NN-rank 21: ['COc1ccccc1C=O']

EI-MS matched molecular weight (relative abundance: 99.99%) for COc1ccccc1C=O

GHS hazard classification: Irritant

NN-rank 22: ['COc1ccccc1C=O']

EI-MS matched molecular weight (relative abundance: 99.99%) for COc1ccccc1C=O

GHS hazard classification: Irritant

NN-rank 23: ['C', 'O=Cc1cccc(O)c1']

NN-rank 24: ['C', 'O=Cc1cccc(O)c1']

NN-rank 25: ['COCc1ccc(O)cc1']

EI-MS matched molecular weight (relative abundance: 5.66%) for COCc1ccc(O)cc1

GHS hazard classification: none of the hazards selected for correlation

NN/MS matches corresponding with tox GHS classification highlight in Bold font.

46. Anisyl Acetate: CC(=O)OCC1=CC=C(C=C1)OC

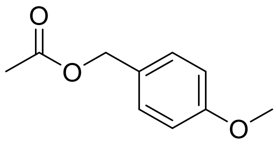

Summary Report of GHS Classification for NN/MS Matches:

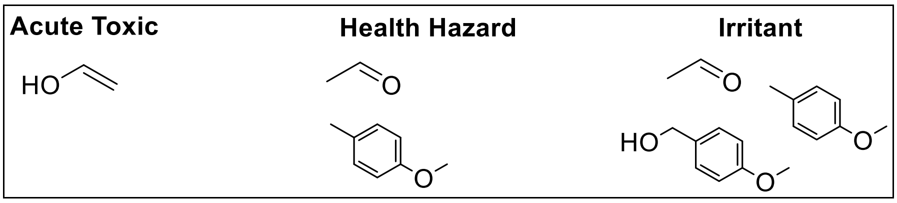

NN-rank 1: ['CC=O', 'COc1ccc(CO)cc1']

EI-MS matched molecular weight (relative abundance: 18.59%) for CC=O

GHS hazard classification: Health Hazard, Irritant

EI-MS matched molecular weight (relative abundance: 6.39%) for COc1ccc(CO)cc1

GHS hazard classification: Irritant

NN-rank 2: ['CC(=O)O', 'COc1ccc(C)cc1']

EI-MS matched molecular weight (relative abundance: 99.99%) for COc1ccc(C)cc1

GHS hazard classification: Health Hazard, Irritant

NN-rank 3: ['C', 'CC(=O)OCc1ccc(O)cc1']

NN-rank 4: ['C=C=O', 'COc1ccc(CO)cc1']

EI-MS matched molecular weight (relative abundance: 6.39%) for COc1ccc(CO)cc1

GHS hazard classification: Irritant

NN-rank 5: ['CCO', 'COc1ccc(CO)cc1']

EI-MS matched molecular weight (relative abundance: 6.39%) for COc1ccc(CO)cc1

GHS hazard classification: Irritant

NN-rank 6: ['CC(=O)OCc1ccccc1', 'CO']

NN-rank 7: ['COc1ccc(COCC=O)cc1']

NN-rank 8: ['C', 'COc1ccc(COC=O)cc1']

NN-rank 9: ['C', 'C=O', 'COc1ccc(CO)cc1']

EI-MS matched molecular weight (relative abundance: 6.39%) for COc1ccc(CO)cc1

GHS hazard classification: Irritant

NN-rank 10: ['COC(C)=O', 'COc1ccccc1']

NN/MS matches corresponding with tox GHS classification highlight in Bold font.

NN-rank 11: ['COc1ccc(COC(C)O)cc1']

NN-rank 12: ['COc1ccc2cc1CC(=O)OC2']

NN-rank 13: ['COc1ccc2cc1CC(=O)OC2']

NN-rank 14: ['COc1ccc(C)cc1', 'O=CCO']

EI-MS matched molecular weight (relative abundance: 99.99%) for COc1ccc(C)cc1

GHS hazard classification: Health Hazard, Irritant

NN-rank 15: ['O=C1CCOc2ccc(cc2)CO1']

NN-rank 16: ['COc1ccc(C)cc1', 'O=C1CO1']

EI-MS matched molecular weight (relative abundance: 99.99%) for COc1ccc(C)cc1

GHS hazard classification: Health Hazard, Irritant

NN-rank 17: ['CC=O', 'COc1ccc(C)cc1', 'O']

EI-MS matched molecular weight (relative abundance: 18.59%) for CC=O

GHS hazard classification: Health Hazard, Irritant

EI-MS matched molecular weight (relative abundance: 99.99%) for COc1ccc(C)cc1

GHS hazard classification: Health Hazard, Irritant

NN-rank 18: ['CCC(=O)OCc1ccc(O)cc1']

NN-rank 19: ['C=CO', 'COc1ccc(CO)cc1']

EI-MS matched molecular weight (relative abundance: 18.59%) for C=CO

GHS hazard classification: Acute Toxic

EI-MS matched molecular weight (relative abundance: 6.39%) for COc1ccc(CO)cc1

GHS hazard classification: Irritant

NN-rank 20: ['C', 'CO', 'COc1ccc(CO)cc1']

EI-MS matched molecular weight (relative abundance: 6.39%) for COc1ccc(CO)cc1

GHS hazard classification: Irritant

NN-rank 21: ['COc1ccc(COC=O)cc1C']

NN-rank 22: ['COc1ccc(COC=O)cc1C']

NN-rank 23: ['CCOc1ccc(COC=O)cc1']

NN-rank 24: ['C=C(O)OCc1ccc(OC)cc1']

NN-rank 25: ['COc1ccc(CO)cc1C=C=O']

NN/MS matches corresponding with tox GHS classification highlight in Bold font.

47. Anisyl Alcohol: COC1=CC=C(C=C1)CO

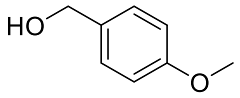

Summary Report of GHS Classification for NN/MS Matches:

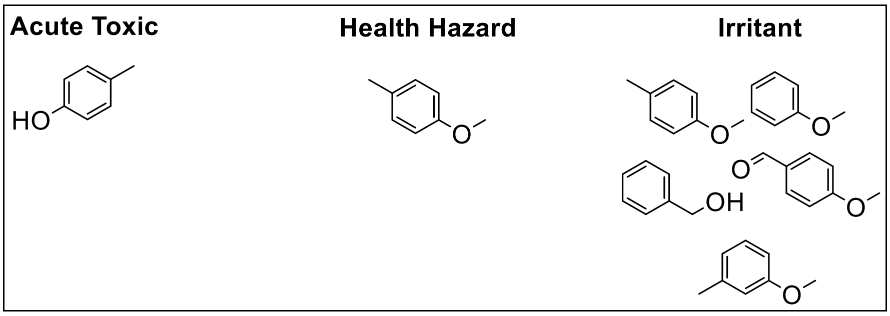

NN-rank 1: ['COc1ccc(C)cc1', 'O']

EI-MS matched molecular weight (relative abundance: 45.09%) for COc1ccc(C)cc1

GHS hazard classification: Health Hazard, Irritant

NN-rank 2: ['C', 'OCc1ccc(O)cc1']

NN-rank 3: ['COc1ccc2c(c1)C2', 'O']

NN-rank 4: ['CO', 'COc1ccccc1']

EI-MS matched molecular weight (relative abundance: 25.29%) for COc1ccccc1

GHS hazard classification: Irritant

NN-rank 5: ['C', 'COc1ccccc1', 'O']

EI-MS matched molecular weight (relative abundance: 25.29%) for COc1ccccc1

GHS hazard classification: Irritant

NN-rank 6: ['CO', 'OCc1ccccc1']

EI-MS matched molecular weight (relative abundance: 25.29%) for OCc1ccccc1

GHS hazard classification: Irritant

NN-rank 7: ['COc1ccc(C=O)cc1']

EI-MS matched molecular weight (relative abundance: 6.19%) for COc1ccc(C=O)cc1

GHS hazard classification: Irritant

NN-rank 8: ['C', 'c1cc2ccc1CO2', 'O']

EI-MS matched molecular weight (relative abundance: 11.89%) for c1cc2ccc1CO2

GHS hazard classification: none of the hazards selected for correlation

NN-rank 9: ['c1cc2ccc1CO2', 'CO']

NN/MS matches corresponding with tox GHS classification highlight in Bold font.

EI-MS matched molecular weight (relative abundance: 11.89%) for c1cc2ccc1CO2

GHS hazard classification: none of the hazards selected for correlation

NN-rank 10: ['OCc1ccc2c(c1)CO2']

EI-MS matched molecular weight (relative abundance: 6.19%) for OCc1ccc2c(c1)CO2

GHS hazard classification: unknown

NN-rank 11: ['OCc1ccc2c(c1)CO2']

EI-MS matched molecular weight (relative abundance: 6.19%) for OCc1ccc2c(c1)CO2

GHS hazard classification: unknown

NN-rank 12: ['COc1cccc(C)c1', 'O']

EI-MS matched molecular weight (relative abundance: 45.09%) for COc1cccc(C)c1

GHS hazard classification: Irritant

NN-rank 13: ['COc1ccc2c(c1)C2O']

EI-MS matched molecular weight (relative abundance: 6.19%) for COc1ccc2c(c1)C2O

GHS hazard classification: unknown

NN-rank 14: ['C', 'OCc1ccc2c(c1)O2']

EI-MS matched molecular weight (relative abundance: 45.09%) for OCc1ccc2c(c1)O2

GHS hazard classification: none of the hazards selected for correlation

NN-rank 15: ['C', 'OCc1ccc2c(c1)O2']

EI-MS matched molecular weight (relative abundance: 45.09%) for OCc1ccc2c(c1)O2

GHS hazard classification: none of the hazards selected for correlation

NN-rank 16: ['C', 'OCc1ccc2cc1O2']

EI-MS matched molecular weight (relative abundance: 45.09%) for OCc1ccc2cc1O2

GHS hazard classification: unknown

NN-rank 17: ['C', 'OCc1ccc2cc1O2']

EI-MS matched molecular weight (relative abundance: 45.09%) for OCc1ccc2cc1O2

GHS hazard classification: unknown

NN-rank 18: ['C=O', 'OCc1ccccc1']

EI-MS matched molecular weight (relative abundance: 25.29%) for OCc1ccccc1

GHS hazard classification: Irritant

NN-rank 19: ['Cc1cc(CO)ccc1O']

EI-MS matched molecular weight (relative abundance: 58.89%) for Cc1cc(CO)ccc1O

GHS hazard classification: none of the hazards selected for correlation

NN-rank 20: ['Cc1cc(CO)ccc1O']

EI-MS matched molecular weight (relative abundance: 58.89%) for Cc1cc(CO)ccc1O

GHS hazard classification: none of the hazards selected for correlation

NN/MS matches corresponding with tox GHS classification highlight in Bold font.

NN-rank 21: ['C=O', 'COc1ccccc1']

EI-MS matched molecular weight (relative abundance: 25.29%) for COc1ccccc1

GHS hazard classification: Irritant

NN-rank 22: ['C', 'Cc1ccc(O)cc1', 'O']

EI-MS matched molecular weight (relative abundance: 25.29%) for Cc1ccc(O)cc1

GHS hazard classification: Acute Toxic

NN-rank 23: ['CO', 'Cc1ccc(O)cc1']

EI-MS matched molecular weight (relative abundance: 25.29%) for Cc1ccc(O)cc1

GHS hazard classification: Acute Toxic

NN-rank 24: ['C', 'C', 'O', 'c1cc2ccc1o2']

EI-MS matched molecular weight (relative abundance: 6.39%) for c1cc2ccc1o2

GHS hazard classification: none of the hazards selected for correlation

NN-rank 25: ['C', 'CO', 'c1cc2ccc1o2']

EI-MS matched molecular weight (relative abundance: 6.39%) for c1cc2ccc1o2

GHS hazard classification: none of the hazards selected for correlation

NN/MS matches corresponding with tox GHS classification highlight in Bold font.

48. Benzaldehyde: C1=CC=C(C=C1)C=O

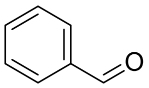

Summary Report of GHS Classification for NN/MS Matches:

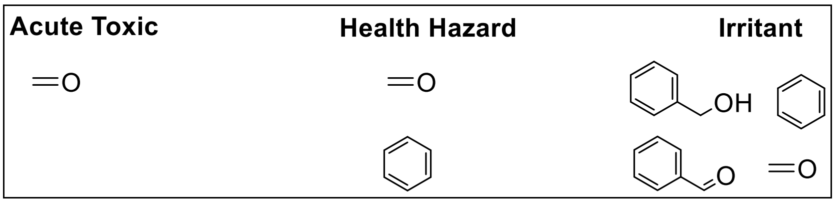

NN-rank 1: ['OCc1ccccc1']

EI-MS matched molecular weight (relative abundance: 7.58%) for OCc1ccccc1

GHS hazard classification: Irritant

NN-rank 2: ['C=O', 'c1ccccc1']

EI-MS matched molecular weight (relative abundance: 5.07%) for C=O

GHS hazard classification: Acute Toxic, Health Hazard, Irritant

EI-MS matched molecular weight (relative abundance: 99.99%) for c1ccccc1

GHS hazard classification: Health Hazard, Irritant

NN-rank 3: ['Cc1ccccc1', 'O']

NN-rank 4: ['CO', 'c1ccccc1']

EI-MS matched molecular weight (relative abundance: 99.99%) for c1ccccc1

GHS hazard classification: Health Hazard, Irritant

NN-rank 5: ['O=C1C2=CC=C1C=C2']

NN-rank 6: ['OC1C2=CC=C1C=C2']

EI-MS matched molecular weight (relative abundance: 88.97%) for OC1C2=CC=C1C=C2

GHS hazard classification: none of the hazards selected for correlation

NN-rank 7: ['c1ccc2c(c1)CO2']

EI-MS matched molecular weight (relative abundance: 88.97%) for c1ccc2c(c1)CO2

GHS hazard classification: none of the hazards selected for correlation

NN-rank 8: ['c1ccc2c(c1)CO2']

EI-MS matched molecular weight (relative abundance: 88.97%) for c1ccc2c(c1)CO2

GHS hazard classification: none of the hazards selected for correlation

NN-rank 9: ['O=c1c2ccccc12']

NN/MS matches corresponding with tox GHS classification highlight in Bold font.

NN-rank 10: ['O=c1c2ccccc12']

NN-rank 11: ['c1cc2ccc1CO2']

EI-MS matched molecular weight (relative abundance: 88.97%) for c1cc2ccc1CO2

GHS hazard classification: none of the hazards selected for correlation

NN-rank 12: ['OC1c2ccccc21']

EI-MS matched molecular weight (relative abundance: 88.97%) for OC1c2ccccc21

GHS hazard classification: none of the hazards selected for correlation

NN-rank 13: ['OC1c2ccccc21']

EI-MS matched molecular weight (relative abundance: 88.97%) for OC1c2ccccc21

GHS hazard classification: none of the hazards selected for correlation

NN-rank 14: ['c1cc2cc(c1)OC2']

EI-MS matched molecular weight (relative abundance: 88.97%) for c1cc2cc(c1)OC2

GHS hazard classification: none of the hazards selected for correlation

NN-rank 15: ['c1cc2cc(c1)OC2']

EI-MS matched molecular weight (relative abundance: 88.97%) for c1cc2cc(c1)OC2

GHS hazard classification: none of the hazards selected for correlation

NN-rank 16: ['O=Cc1cc2ccc1-2']

NN-rank 17: ['O=Cc1cc2ccc1-2']

NN-rank 18: ['C1=CC2=CC=C1C2', 'O']

NN-rank 19: ['O=Cc1ccccc1']

EI-MS matched molecular weight (relative abundance: 88.97%) for O=Cc1ccccc1

GHS hazard classification: Irritant

NN-rank 20: ['O=Cc1ccccc1']

EI-MS matched molecular weight (relative abundance: 88.97%) for O=Cc1ccccc1

GHS hazard classification: Irritant

NN-rank 21: ['O=Cc1ccccc1']

EI-MS matched molecular weight (relative abundance: 88.97%) for O=Cc1ccccc1

GHS hazard classification: Irritant

NN-rank 22: ['c1ccc2c(c1)C2', 'O']

NN-rank 23: ['c1ccc2c(c1)C2', 'O']

NN-rank 24: ['OCc1ccccc1']

EI-MS matched molecular weight (relative abundance: 7.58%) for OCc1ccccc1

GHS hazard classification: Irritant

NN-rank 25: ['OCc1ccccc1']

EI-MS matched molecular weight (relative abundance: 7.58%) for OCc1ccccc1

NN/MS matches corresponding with tox GHS classification highlight in Bold font.

GHS hazard classification: Irritant

NN/MS matches corresponding with tox GHS classification highlight in Bold font.

49. Benzaldehyde propylene glycol acetal: CC1COC(O1)C2=CC=CC=C2

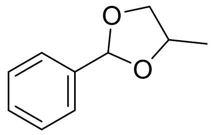

Summary Report of GHS Classification for NN/MS Matches:

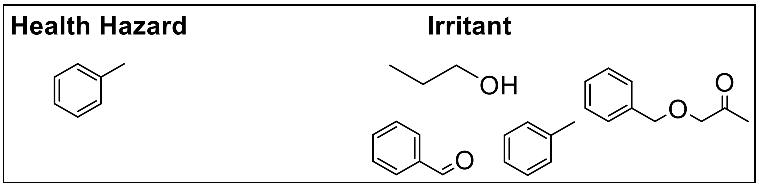

NN-rank 1: ['CC(O)CO', 'Cc1ccccc1']

EI-MS matched molecular weight (relative abundance: 34.0%) for Cc1ccccc1

GHS hazard classification: Health Hazard, Irritant

NN-rank 2: ['CC(O)COCc1ccccc1']

NN-rank 3: ['CC(CO)OCc1ccccc1']

NN-rank 4: ['CC(C)O', 'O=Cc1ccccc1']

EI-MS matched molecular weight (relative abundance: 13.5%) for CC(C)O

GHS hazard classification: none of the hazards selected for correlation

EI-MS matched molecular weight (relative abundance: 75.0%) for O=Cc1ccccc1

GHS hazard classification: Irritant

NN-rank 5: ['CCCO', 'O=Cc1ccccc1']

EI-MS matched molecular weight (relative abundance: 13.5%) for CCCO

GHS hazard classification: Irritant

EI-MS matched molecular weight (relative abundance: 75.0%) for O=Cc1ccccc1

GHS hazard classification: Irritant

NN-rank 6: ['CC(O)C=O', 'Cc1ccccc1']

EI-MS matched molecular weight (relative abundance: 34.0%) for Cc1ccccc1

GHS hazard classification: Health Hazard, Irritant

NN-rank 7: ['CC(=O)CO', 'Cc1ccccc1']

EI-MS matched molecular weight (relative abundance: 34.0%) for Cc1ccccc1

GHS hazard classification: Health Hazard, Irritant

NN-rank 8: ['CC(C=O)OCc1ccccc1']

EI-MS matched molecular weight (relative abundance: 99.9%) for CC(C=O)OCc1ccccc1

NN/MS matches corresponding with tox GHS classification highlight in Bold font.

GHS hazard classification: none of the hazards selected for correlation

NN-rank 9: ['CCCOC(O)c1ccccc1']

NN-rank 10: ['CC(=O)COCc1ccccc1']

EI-MS matched molecular weight (relative abundance: 99.9%) for CC(=O)COCc1ccccc1

GHS hazard classification: Irritant

NN-rank 11: ['CCCO', 'Cc1ccccc1', 'O']

EI-MS matched molecular weight (relative abundance: 13.5%) for CCCO

GHS hazard classification: Irritant

EI-MS matched molecular weight (relative abundance: 34.0%) for Cc1ccccc1

GHS hazard classification: Health Hazard, Irritant

NN-rank 12: ['CCC=O', 'O=Cc1ccccc1']

EI-MS matched molecular weight (relative abundance: 75.0%) for O=Cc1ccccc1

GHS hazard classification: Irritant

NN-rank 13: ['CCCOC(=O)c1ccccc1']

EI-MS matched molecular weight (relative abundance: 99.9%) for CCCOC(=O)c1ccccc1

GHS hazard classification: none of the hazards selected for correlation

NN-rank 14: ['CC(C)OC(O)c1ccccc1']

NN-rank 15: ['CC(CO)c1ccccc1C=O']

EI-MS matched molecular weight (relative abundance: 99.9%) for CC(CO)c1ccccc1C=O

GHS hazard classification: none of the hazards selected for correlation

NN-rank 16: ['CC(CO)c1ccccc1C=O']

EI-MS matched molecular weight (relative abundance: 99.9%) for CC(CO)c1ccccc1C=O

GHS hazard classification: none of the hazards selected for correlation

NN-rank 17: ['CC(C)=O', 'O=Cc1ccccc1']

EI-MS matched molecular weight (relative abundance: 75.0%) for O=Cc1ccccc1

GHS hazard classification: Irritant

NN-rank 18: ['CC1COC(O)c2ccccc21']

EI-MS matched molecular weight (relative abundance: 99.9%) for CC1COC(O)c2ccccc21

GHS hazard classification: unknown

NN-rank 19: ['CC1COC(O)c2ccccc21']

NN/MS matches corresponding with tox GHS classification highlight in Bold font.

EI-MS matched molecular weight (relative abundance: 99.9%) for CC1COC(O)c2ccccc21

GHS hazard classification: unknown

NN-rank 20: ['CC(C)OC(=O)c1ccccc1']

EI-MS matched molecular weight (relative abundance: 99.9%) for CC(C)OC(=O)c1ccccc1

GHS hazard classification: none of the hazards selected for correlation

NN-rank 21: ['CCCOCc1ccccc1', 'O']

NN-rank 22: ['CC12COC(O1)c1ccccc12']

NN-rank 23: ['CC12COC(O1)c1ccccc12']

NN-rank 24: ['CC1(OCc2ccccc2)CO1']

EI-MS matched molecular weight (relative abundance: 99.9%) for CC1(OCc2ccccc2)CO1

GHS hazard classification: none of the hazards selected for correlation

NN-rank 25: ['c1ccc2c(c1)CC1COC2O1']

NN/MS matches corresponding with tox GHS classification highlight in Bold font.

50. Benzyl Acetate: CC(=O)OCC1=CC=CC=C1

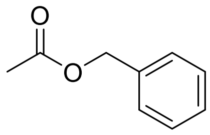

Summary Report of GHS Classification for NN/MS Matches:

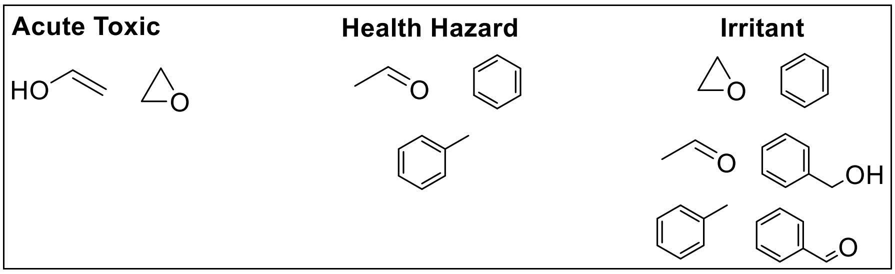

NN-rank 1: ['CC=O', 'OCc1ccccc1']

EI-MS matched molecular weight (relative abundance: 37.63%) for CC=O

GHS hazard classification: Health Hazard, Irritant

EI-MS matched molecular weight (relative abundance: 19.52%) for OCc1ccccc1

GHS hazard classification: Irritant

NN-rank 2: ['CC(=O)O', 'Cc1ccccc1']

EI-MS matched molecular weight (relative abundance: 71.66%) for Cc1ccccc1

GHS hazard classification: Health Hazard, Irritant

NN-rank 3: ['CCO', 'OCc1ccccc1']

EI-MS matched molecular weight (relative abundance: 19.52%) for OCc1ccccc1

GHS hazard classification: Irritant

NN-rank 4: ['C=C=O', 'OCc1ccccc1']

EI-MS matched molecular weight (relative abundance: 19.52%) for OCc1ccccc1

GHS hazard classification: Irritant

NN-rank 5: ['O=CCOCc1ccccc1']

NN-rank 6: ['COC(C)=O', 'c1ccccc1']

EI-MS matched molecular weight (relative abundance: 24.62%) for c1ccccc1

GHS hazard classification: Health Hazard, Irritant

NN-rank 7: ['C', 'C=O', 'OCc1ccccc1']

EI-MS matched molecular weight (relative abundance: 19.52%) for OCc1ccccc1

GHS hazard classification: Irritant

NN-rank 8: ['C', 'O=COCc1ccccc1']

NN/MS matches corresponding with tox GHS classification highlight in Bold font.

NN-rank 9: ['Cc1ccccc1', 'O=CCO']

EI-MS matched molecular weight (relative abundance: 71.66%) for Cc1ccccc1

GHS hazard classification: Health Hazard, Irritant

NN-rank 10: ['CC=O', 'O=Cc1ccccc1']

EI-MS matched molecular weight (relative abundance: 37.63%) for CC=O

GHS hazard classification: Health Hazard, Irritant

EI-MS matched molecular weight (relative abundance: 5.2%) for O=Cc1ccccc1

GHS hazard classification: Irritant

NN-rank 11: ['O=C1Cc2ccccc2CO1']

NN-rank 12: ['O=C1Cc2ccccc2CO1']

NN-rank 13: ['c1ccc2c(c1)C2', 'CC(=O)O']

EI-MS matched molecular weight (relative abundance: 17.92%) for c1ccc2c(c1)C2

GHS hazard classification: none of the hazards selected for correlation

NN-rank 14: ['c1ccc2c(c1)C2', 'CC(=O)O']

EI-MS matched molecular weight (relative abundance: 17.92%) for c1ccc2c(c1)C2

GHS hazard classification: none of the hazards selected for correlation

NN-rank 15: ['CC(O)OCc1ccccc1']

NN-rank 16: ['Cc1ccccc1', 'O=C1CO1']

EI-MS matched molecular weight (relative abundance: 71.66%) for Cc1ccccc1

GHS hazard classification: Health Hazard, Irritant

NN-rank 17: ['C=CO', 'OCc1ccccc1']

EI-MS matched molecular weight (relative abundance: 37.63%) for C=CO

GHS hazard classification: Acute Toxic

EI-MS matched molecular weight (relative abundance: 19.52%) for OCc1ccccc1

GHS hazard classification: Irritant

NN-rank 18: ['CC=O', 'Cc1ccccc1', 'O']

EI-MS matched molecular weight (relative abundance: 37.63%) for CC=O

GHS hazard classification: Health Hazard, Irritant

EI-MS matched molecular weight (relative abundance: 71.66%) for Cc1ccccc1

GHS hazard classification: Health Hazard, Irritant

NN-rank 19: ['C1CO1', 'OCc1ccccc1']

EI-MS matched molecular weight (relative abundance: 37.63%) for C1CO1

GHS hazard classification: Acute Toxic, Health Hazard, Irritant

EI-MS matched molecular weight (relative abundance: 19.52%) for OCc1ccccc1

GHS hazard classification: Irritant

NN/MS matches corresponding with tox GHS classification highlight in Bold font.

NN-rank 20: ['OCCOCc1ccccc1']

NN-rank 21: ['CCOCc1ccccc1', 'O']

NN-rank 22: ['CC(=O)OC1c2ccccc21']

NN-rank 23: ['CC(=O)OC1c2ccccc21']

NN-rank 24: ['Cc1ccccc1COC=O']

NN-rank 25: ['Cc1ccccc1COC=O']

NN/MS matches corresponding with tox GHS classification highlight in Bold font.

51. Benzyl Alcohol: C1=CC=C(C=C1)CO

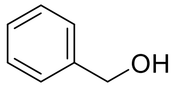

Summary Report of GHS Classification for NN/MS Matches:

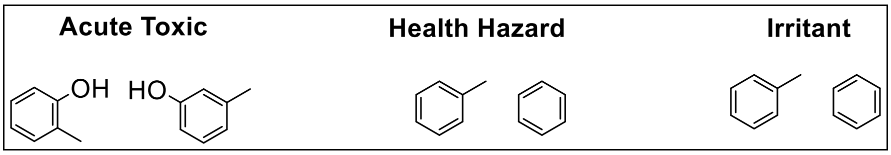

NN-rank 1: ['Cc1ccccc1', 'O']

EI-MS matched molecular weight (relative abundance: 18.0%) for Cc1ccccc1

GHS hazard classification: Health Hazard, Irritant

NN-rank 2: ['c1ccc2c(c1)C2', 'O']

NN-rank 3: ['c1ccc2c(c1)C2', 'O']

NN-rank 4: ['O=Cc1ccccc1']

NN-rank 5: ['CO', 'c1ccccc1']

EI-MS matched molecular weight (relative abundance: 54.0%) for c1ccccc1

GHS hazard classification: Health Hazard, Irritant

NN-rank 6: ['C', 'O', 'c1ccccc1']

EI-MS matched molecular weight (relative abundance: 54.0%) for c1ccccc1

GHS hazard classification: Health Hazard, Irritant

NN-rank 7: ['Cc1ccccc1O']

EI-MS matched molecular weight (relative abundance: 70.0%) for Cc1ccccc1O

GHS hazard classification: Acute Toxic

NN-rank 8: ['Cc1ccccc1O']

EI-MS matched molecular weight (relative abundance: 70.0%) for Cc1ccccc1O

GHS hazard classification: Acute Toxic

NN-rank 9: ['Cc1cccc(O)c1']

EI-MS matched molecular weight (relative abundance: 70.0%) for Cc1cccc(O)c1

GHS hazard classification: Acute Toxic

NN-rank 10: ['Cc1cccc(O)c1']

EI-MS matched molecular weight (relative abundance: 70.0%) for Cc1cccc(O)c1

GHS hazard classification: Acute Toxic

NN-rank 11: ['OC1c2ccccc21']

NN/MS matches corresponding with tox GHS classification highlight in Bold font.

NN-rank 12: ['OC1c2ccccc21']

NN-rank 13: ['c1cc2cc(c1)OC2']

NN-rank 14: ['c1cc2cc(c1)OC2']

NN-rank 15: ['c1ccc2c(c1)CO2']

NN-rank 16: ['c1ccc2c(c1)CO2']

NN-rank 17: ['Cc1ccccc1', 'O']

EI-MS matched molecular weight (relative abundance: 18.0%) for Cc1ccccc1

GHS hazard classification: Health Hazard, Irritant

NN-rank 18: ['Cc1ccccc1', 'O']

EI-MS matched molecular weight (relative abundance: 18.0%) for Cc1ccccc1

GHS hazard classification: Health Hazard, Irritant

NN-rank 19: ['Cc1ccccc1', 'O']

EI-MS matched molecular weight (relative abundance: 18.0%) for Cc1ccccc1

GHS hazard classification: Health Hazard, Irritant

NN-rank 20: ['OCc1cc2ccc1-2']

NN-rank 21: ['OCc1cc2ccc1-2']

NN-rank 22: ['C=O', 'c1ccccc1']

EI-MS matched molecular weight (relative abundance: 54.0%) for c1ccccc1

GHS hazard classification: Health Hazard, Irritant

NN-rank 23: ['OCc1ccccc1']

EI-MS matched molecular weight (relative abundance: 70.0%) for OCc1ccccc1

GHS hazard classification: Irritant

NN-rank 24: ['OCc1ccccc1']

EI-MS matched molecular weight (relative abundance: 70.0%) for OCc1ccccc1

GHS hazard classification: Irritant

NN-rank 25: ['OCc1ccccc1']

EI-MS matched molecular weight (relative abundance: 70.0%) for OCc1ccccc1

GHS hazard classification: Irritant

NN/MS matches corresponding with tox GHS classification highlight in Bold font.

52. Benzyl Benzoate: C1=CC=C(C=C1)COC(=O)C2=CC=CC=C2

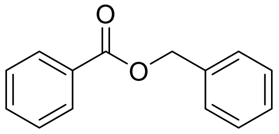

Summary Report of GHS Classification for NN/MS Matches:

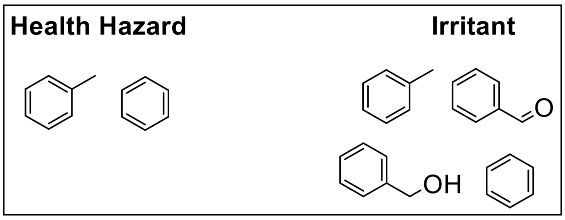

NN-rank 1: ['Cc1ccccc1', 'O=C(O)c1ccccc1']

EI-MS matched molecular weight (relative abundance: 46.17%) for Cc1ccccc1

GHS hazard classification: Health Hazard, Irritant

NN-rank 2: ['Cc1ccccc1', 'O=C(O)c1ccccc1']

EI-MS matched molecular weight (relative abundance: 46.17%) for Cc1ccccc1

GHS hazard classification: Health Hazard, Irritant

NN-rank 3: ['O=Cc1ccccc1', 'OCc1ccccc1']

EI-MS matched molecular weight (relative abundance: 99.99%) for O=Cc1ccccc1

GHS hazard classification: Irritant

EI-MS matched molecular weight (relative abundance: 5.64%) for OCc1ccccc1

GHS hazard classification: Irritant

NN-rank 4: ['OCc1ccccc1', 'OCc1ccccc1']

EI-MS matched molecular weight (relative abundance: 5.64%) for OCc1ccccc1

GHS hazard classification: Irritant

EI-MS matched molecular weight (relative abundance: 5.64%) for OCc1ccccc1

GHS hazard classification: Irritant

NN-rank 5: ['Cc1ccccc1', 'O', 'O=Cc1ccccc1']

EI-MS matched molecular weight (relative abundance: 46.17%) for Cc1ccccc1

GHS hazard classification: Health Hazard, Irritant

EI-MS matched molecular weight (relative abundance: 99.99%) for O=Cc1ccccc1

GHS hazard classification: Irritant

NN-rank 6: ['COC(=O)c1ccccc1', 'c1ccccc1']

EI-MS matched molecular weight (relative abundance: 32.54%) for c1ccccc1

NN/MS matches corresponding with tox GHS classification highlight in Bold font.

GHS hazard classification: Health Hazard, Irritant

NN-rank 7: ['c1ccc(COCc2ccccc2)cc1', 'O']

NN-rank 8: ['c1ccc2c(c1)C2', 'O=C(O)c1ccccc1']

EI-MS matched molecular weight (relative abundance: 5.46%) for c1ccc2c(c1)C2

GHS hazard classification: none of the hazards selected for correlation

NN-rank 9: ['c1ccc2c(c1)C2', 'O=C(O)c1ccccc1']

EI-MS matched molecular weight (relative abundance: 5.46%) for c1ccc2c(c1)C2

GHS hazard classification: none of the hazards selected for correlation

NN-rank 10: ['O=COCc1ccccc1', 'c1ccccc1']

EI-MS matched molecular weight (relative abundance: 32.54%) for c1ccccc1

GHS hazard classification: Health Hazard, Irritant

NN-rank 11: ['Cc1ccccc1', 'O', 'OCc1ccccc1']

EI-MS matched molecular weight (relative abundance: 46.17%) for Cc1ccccc1

GHS hazard classification: Health Hazard, Irritant

EI-MS matched molecular weight (relative abundance: 5.64%) for OCc1ccccc1

GHS hazard classification: Irritant

NN-rank 12: ['C', 'O=C(O)c1ccccc1', 'c1ccccc1']

EI-MS matched molecular weight (relative abundance: 32.54%) for c1ccccc1

GHS hazard classification: Health Hazard, Irritant

NN-rank 13: ['Cc1ccccc1', 'O', 'OCc1ccccc1']

EI-MS matched molecular weight (relative abundance: 46.17%) for Cc1ccccc1

GHS hazard classification: Health Hazard, Irritant

EI-MS matched molecular weight (relative abundance: 5.64%) for OCc1ccccc1

GHS hazard classification: Irritant

NN-rank 14: ['Cc1ccccc1C(=O)c1ccccc1', 'O']

NN-rank 15: ['Cc1ccccc1C(=O)c1ccccc1', 'O']

NN-rank 16: ['Cc1ccccc1', 'O=CO', 'c1ccccc1']

EI-MS matched molecular weight (relative abundance: 46.17%) for Cc1ccccc1

GHS hazard classification: Health Hazard, Irritant

EI-MS matched molecular weight (relative abundance: 32.54%) for c1ccccc1

GHS hazard classification: Health Hazard, Irritant

NN-rank 17: ['C', 'O=C(O)c1ccccc1', 'c1ccccc1']

EI-MS matched molecular weight (relative abundance: 32.54%) for c1ccccc1

GHS hazard classification: Health Hazard, Irritant

NN-rank 18: ['c1ccc2c(c1)C2', 'O=C(O)c1ccccc1']

NN/MS matches corresponding with tox GHS classification highlight in Bold font.

EI-MS matched molecular weight (relative abundance: 5.46%) for c1ccc2c(c1)C2

GHS hazard classification: none of the hazards selected for correlation

NN-rank 19: ['c1ccc2c(c1)C2', 'O=C(O)c1ccccc1']

EI-MS matched molecular weight (relative abundance: 5.46%) for c1ccc2c(c1)C2

GHS hazard classification: none of the hazards selected for correlation

NN-rank 20: ['O=C(c1ccccc1)c1ccccc1CO']

NN-rank 21: ['O=C(c1ccccc1)c1ccccc1CO']

NN-rank 22: ['Cc1ccccc1', 'O=C=O', 'c1ccccc1']

EI-MS matched molecular weight (relative abundance: 46.17%) for Cc1ccccc1

GHS hazard classification: Health Hazard, Irritant

EI-MS matched molecular weight (relative abundance: 32.54%) for c1ccccc1

GHS hazard classification: Health Hazard, Irritant

NN-rank 23: ['Cc1ccccc1', 'O', 'O=Cc1ccccc1']

EI-MS matched molecular weight (relative abundance: 46.17%) for Cc1ccccc1

GHS hazard classification: Health Hazard, Irritant

EI-MS matched molecular weight (relative abundance: 99.99%) for O=Cc1ccccc1

GHS hazard classification: Irritant

NN-rank 24: ['Cc1ccccc1', 'O', 'O=c1c2ccccc12']

EI-MS matched molecular weight (relative abundance: 46.17%) for Cc1ccccc1

GHS hazard classification: Health Hazard, Irritant

NN-rank 25: ['Cc1ccccc1', 'O', 'O=C1C2=CC=C1C=C2']

EI-MS matched molecular weight (relative abundance: 46.17%) for Cc1ccccc1

GHS hazard classification: Health Hazard, Irritant

NN/MS matches corresponding with tox GHS classification highlight in Bold font.

53. Benzyl Butyrate: CCCC(=O)OCC1=CC=CC=C1

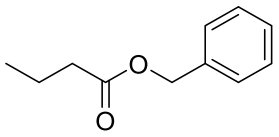

Summary Report of GHS Classification for NN/MS Matches:

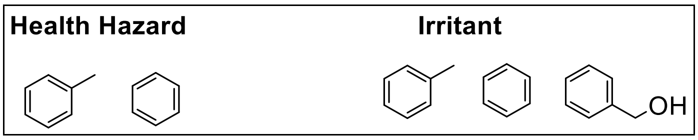

NN-rank 1: ['CCCC(=O)O', 'Cc1ccccc1']

EI-MS matched molecular weight (relative abundance: 92.78%) for Cc1ccccc1

GHS hazard classification: Health Hazard, Irritant

NN-rank 2: ['CCCC(=O)O', 'Cc1ccccc1']

EI-MS matched molecular weight (relative abundance: 92.78%) for Cc1ccccc1

GHS hazard classification: Health Hazard, Irritant

NN-rank 3: ['CCCC=O', 'Cc1ccccc1', 'O']

EI-MS matched molecular weight (relative abundance: 22.92%) for CCCC=O

GHS hazard classification: none of the hazards selected for correlation

EI-MS matched molecular weight (relative abundance: 92.78%) for Cc1ccccc1

GHS hazard classification: Health Hazard, Irritant

NN-rank 4: ['CCCC(=O)c1ccccc1C', 'O']

NN-rank 5: ['CCCC(=O)c1ccccc1C', 'O']

NN-rank 6: ['CCC1OC1=O', 'Cc1ccccc1']

EI-MS matched molecular weight (relative abundance: 92.78%) for Cc1ccccc1

GHS hazard classification: Health Hazard, Irritant

NN-rank 7: ['CCC', 'Cc1ccccc1', 'O=CO']

EI-MS matched molecular weight (relative abundance: 14.91%) for CCC

GHS hazard classification: none of the hazards selected for correlation

EI-MS matched molecular weight (relative abundance: 92.78%) for Cc1ccccc1

GHS hazard classification: Health Hazard, Irritant

NN-rank 8: ['CCC(O)C=O', 'Cc1ccccc1']

EI-MS matched molecular weight (relative abundance: 92.78%) for Cc1ccccc1

GHS hazard classification: Health Hazard, Irritant

NN-rank 9: ['C', 'CCC(=O)OCc1ccccc1']

NN/MS matches corresponding with tox GHS classification highlight in Bold font.

NN-rank 10: ['C=O', 'CCC', 'Cc1ccccc1', 'O']

EI-MS matched molecular weight (relative abundance: 14.91%) for CCC

GHS hazard classification: none of the hazards selected for correlation

EI-MS matched molecular weight (relative abundance: 92.78%) for Cc1ccccc1

GHS hazard classification: Health Hazard, Irritant

NN-rank 11: ['C', 'CCCC(=O)O', 'c1ccccc1']

EI-MS matched molecular weight (relative abundance: 6.61%) for c1ccccc1

GHS hazard classification: Health Hazard, Irritant

NN-rank 12: ['CC', 'Cc1ccccc1', 'O=C1CO1']

EI-MS matched molecular weight (relative abundance: 92.78%) for Cc1ccccc1

GHS hazard classification: Health Hazard, Irritant

NN-rank 13: ['CCC', 'Cc1ccccc1', 'O=C=O']

EI-MS matched molecular weight (relative abundance: 14.91%) for CCC

GHS hazard classification: none of the hazards selected for correlation

EI-MS matched molecular weight (relative abundance: 92.78%) for Cc1ccccc1

GHS hazard classification: Health Hazard, Irritant

NN-rank 14: ['CCCC=O', 'OCc1ccccc1']

EI-MS matched molecular weight (relative abundance: 22.92%) for CCCC=O

GHS hazard classification: none of the hazards selected for correlation

EI-MS matched molecular weight (relative abundance: 9.41%) for OCc1ccccc1

GHS hazard classification: Irritant

NN-rank 15: ['CCCCOCc1ccccc1', 'O']

NN-rank 16: ['C', 'CCCC(=O)O', 'c1ccccc1']

EI-MS matched molecular weight (relative abundance: 6.61%) for c1ccccc1

GHS hazard classification: Health Hazard, Irritant

NN-rank 17: ['CCCCO', 'OCc1ccccc1']

EI-MS matched molecular weight (relative abundance: 9.41%) for OCc1ccccc1

GHS hazard classification: Irritant

NN-rank 18: ['CCCC(=O)OC', 'c1ccccc1']

EI-MS matched molecular weight (relative abundance: 6.61%) for c1ccccc1

GHS hazard classification: Health Hazard, Irritant

NN-rank 19: ['C', 'CCCC=O', 'O', 'c1ccccc1']

EI-MS matched molecular weight (relative abundance: 22.92%) for CCCC=O

GHS hazard classification: none of the hazards selected for correlation

EI-MS matched molecular weight (relative abundance: 6.61%) for c1ccccc1

NN/MS matches corresponding with tox GHS classification highlight in Bold font.

GHS hazard classification: Health Hazard, Irritant

NN-rank 20: ['CCC(=O)OCc1ccccc1C']

NN-rank 21: ['CCC(=O)OCc1ccccc1C']

NN-rank 22: ['CC', 'CC(=O)OCc1ccccc1']

NN-rank 23: ['CCC', 'O=COCc1ccccc1']

EI-MS matched molecular weight (relative abundance: 14.91%) for CCC

GHS hazard classification: none of the hazards selected for correlation

NN-rank 24: ['CC', 'Cc1ccccc1', 'O=CCO']

EI-MS matched molecular weight (relative abundance: 92.78%) for Cc1ccccc1

GHS hazard classification: Health Hazard, Irritant

NN-rank 25: ['CCC', 'Cc1ccccc1C(=O)O']

EI-MS matched molecular weight (relative abundance: 14.91%) for CCC

GHS hazard classification: none of the hazards selected for correlation

NN/MS matches corresponding with tox GHS classification highlight in Bold font.

54. beta-Caryophyllene: C/C/1=C\CCC(=C)[C@H]2CC([C@@H]2CC1)(C)C

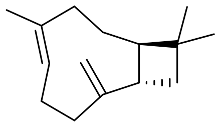

NN-rank 1: ['C=C1CC=CC(C)CC[C]2[C]1CC2(C)C']

NN-rank 2: ['C=C1CCCC(C)CC[C]2[C]1CC2(C)C']

NN-rank 3: ['C=C1CCCC(C)=CC[C]2[C]1CC2(C)C']

NN-rank 4: ['C', 'C=C1CCCCCC[C]2[C]1CC2(C)C']

EI-MS matched molecular weight (relative abundance: 16.6%) for C=C1CCCCCC[C]2[C]1CC2(C)C

GHS hazard classification: unknown

NN-rank 5: ['C', 'C=C1CC=CCCC[C]2[C]1CC2(C)C']

NN-rank 6: ['C=C1CC=CC(C)=CC[C]2[C]1CC2(C)C']

NN-rank 7: ['C', 'C=C1CCCC=CC[C]2[C]1CC2(C)C']

NN-rank 8: ['CC1CC[C]2[C](CC2(C)C)C2CCC1C2']

NN-rank 9: ['CC1CC[C]2[C](CC2(C)C)C2=CC1CC2']

NN-rank 10: ['CC1=CC[C]2[C](CC2(C)C)C2=CC1CC2']

NN-rank 11: ['CC1=CC[C]2[C](CC2(C)C)C2CCC1C2']

NN-rank 12: ['C=C(C)CCCC(=C)[C@]1CC(C)(C)[C@@]1C']

NN-rank 13: ['C=C(C)CCCC(=C)[C]1CC(C)(C)C1=C']

NN-rank 14: ['C=C1CCCC(C)=C=C[C]2[C]1CC2(C)C']

NN-rank 15: ['C', 'C=C1CC=CC=CC[C]2[C]1CC2(C)C']

NN-rank 16: ['C', 'CC1(C)C[C]2[C]1CCCC1CCC2C1']

EI-MS matched molecular weight (relative abundance: 16.6%) for CC1(C)C[C]2[C]1CCCC1CCC2C1

GHS hazard classification: unknown

NN-rank 17: ['CC1CC[C]2C(=C3CCC1C3)CC2(C)C']

NN-rank 18: ['C=C1CC/C=C(\\C)CC[C]2C1=CC2(C)C']

NN-rank 19: ['CC1CC[C]2[C](CC2(C)C)C2=CC1=CC2']

NN-rank 20: ['C=C1CC=C=C(C)CC[C]2[C]1CC2(C)C']

NN-rank 21: ['C/C1=C\\CCC(C)[C]2CC(C)(C)[C]2CC1']

NN-rank 22: ['CC1CC[C]2[C](CC2(C)C)C2CC=C1C2']

NN-rank 23: ['C=C(C)C=CCC(=C)[C@]1CC(C)(C)[C@@]1C']

NN-rank 24: ['C=C(C)C=CCC(=C)[C]1CC(C)(C)C1=C']

NN/MS matches corresponding with tox GHS classification highlight in Bold font.

NN-rank 25: ['C', 'CC1(C)C[C]2[C]1CCCC1C=C2CC1']

55. beta-Damascenone: C/C=C/C(=O)C1=C(C=CCC1(C)C)C

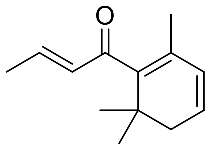

NN-rank 1: ['C/C=C/C(=O)C1=C(C)C=C=CC1(C)C']

NN-rank 2: ['C/C=C/C(=O)C1=C(C)CC=CC1(C)C']

NN-rank 3: ['C/C=C/C(=O)C1C(C)C=CCC1(C)C']

NN-rank 4: ['CCCC(=O)C1=C(C)C=CCC1(C)C']

NN-rank 5: ['C/C=C/C(=O)C1=C(C)CCCC1(C)C']

NN-rank 6: ['C=C=CC(C)=C(C(=O)/C=C/C)C(C)C']

NN-rank 7: ['C', 'C=CC(=O)C1=C(C)C=CCC1(C)C']

NN-rank 8: ['C', 'CCC(=O)C1=C(C)C=CCC1(C)C']

NN-rank 9: ['C=CCC(=O)C1=C(C)C=CCC1(C)C']

NN-rank 10: ['C', 'C/C=C/C(=O)C1=C(C)C=CCC1C']

NN-rank 11: ['C', 'C/C=C/C(=O)C1=C(C)C=CCC1C']

NN-rank 12: ['C=C=CC(=O)C1=C(C)C=CCC1(C)C']

NN-rank 13: ['C/C=C/C(O)C1=C(C)C=CCC1(C)C']

NN-rank 14: ['CC1CC(=O)C2=C(C=CCC2(C)C)C1']

NN-rank 15: ['CC1=CC(=O)C2=C(C=CCC2(C)C)C1']

NN-rank 16: ['C=C1C=CCC(C)(C)C1C(=O)/C=C/C']

NN-rank 17: ['C=CCC(C)(C)C(=CC)C(=O)/C=C/C']

NN-rank 18: ['C=CCC(C)=C(C(=O)/C=C/C)C(C)C']

NN-rank 19: ['C=CCC(C)(C)C(CC)C(=O)/C=C/C']

NN-rank 20: ['CC1CC(=O)C2C(C)(C)CC=CC12C']

NN-rank 21: ['CC=CC(C)(C)C(=CC)C(=O)/C=C/C']

NN-rank 22: ['C/C=C/C(=O)C1C(C)=C=CCC1(C)C']

NN-rank 23: ['C', 'CC1(C)CC=CC2=C1C(=O)C=CC2']

NN-rank 24: ['CC=CC(C)=C(C(=O)/C=C/C)C(C)C']

NN-rank 25: ['CC1=CC(=O)C2C(C)(C)CC=CC12C']

NN/MS matches corresponding with tox GHS classification highlight in Bold font.

56. beta-Damascone: CC=CC(=O)C1=C(CCCC1(C)C)C

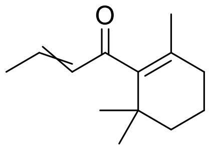

NN-rank 1: ['CC=CC(=O)C1C(C)CCCC1(C)C']

EI-MS matched molecular weight (relative abundance: 8.39%) for CC=CC(=O)C1C(C)CCCC1(C)C

GHS hazard classification: none of the hazards selected for correlation

NN-rank 2: ['CC=CC(=O)C1C(C)=CCCC1(C)C']

NN-rank 3: ['CCCC(=O)C1=C(C)CCCC1(C)C']

EI-MS matched molecular weight (relative abundance: 8.39%) for CCCC(=O)C1=C(C)CCCC1(C)C

GHS hazard classification: none of the hazards selected for correlation

NN-rank 4: ['CC1CC(=O)C2C(C)(C)CCCC12C']

EI-MS matched molecular weight (relative abundance: 8.39%) for CC1CC(=O)C2C(C)(C)CCCC12C

GHS hazard classification: unknown

NN-rank 5: ['CC1=C2C(=O)CC(C)C1CCC2(C)C']

NN-rank 6: ['C=C1CCCC(C)(C)C1C(=O)C=CC']

NN-rank 7: ['C', 'CCC(=O)C1=C(C)CCCC1(C)C']

NN-rank 8: ['CC1(C)CCCC2(C)CC=CC(=O)C12']

NN-rank 9: ['CC1=CC(=O)C2C(C)(C)CCCC12C']

NN-rank 10: ['CC1=C2CCC(C)(C)C1C(=O)CC2C']

NN-rank 11: ['CC=CC(=O)C1=C(C)C=CCC1(C)C']

NN-rank 12: ['C', 'CC=CC(=O)C1=C(C)CCCC1C']

EI-MS matched molecular weight (relative abundance: 99.99%) for CC=CC(=O)C1=C(C)CCCC1C

GHS hazard classification: unknown

NN-rank 13: ['C', 'CC=CC(=O)C1=C(C)CCCC1C']

EI-MS matched molecular weight (relative abundance: 99.99%) for CC=CC(=O)C1=C(C)CCCC1C

GHS hazard classification: unknown

NN-rank 14: ['C=C(C)C(C(=O)C=CC)C(C)(C)CC']

NN/MS matches corresponding with tox GHS classification highlight in Bold font.

EI-MS matched molecular weight (relative abundance: 8.39%) for C=C(C)C(C(=O)C=CC)C(C)(C)CC

GHS hazard classification: unknown

NN-rank 15: ['C=CCC(=O)C1=C(C)CCCC1(C)C']

NN-rank 16: ['C', 'C=CC(=O)C1=C(C)CCCC1(C)C']

EI-MS matched molecular weight (relative abundance: 99.99%) for C=CC(=O)C1=C(C)CCCC1(C)C

GHS hazard classification: none of the hazards selected for correlation

NN-rank 17: ['CC1=CC(=O)C2C(C)=C1CCC2(C)C']

NN-rank 18: ['CC=CC(=O)C(=C(C)CCC)C(C)C']

EI-MS matched molecular weight (relative abundance: 8.39%) for CC=CC(=O)C(=C(C)CCC)C(C)C

GHS hazard classification: unknown

NN-rank 19: ['CC1=CC(=O)C2=C(C)C1CCC2(C)C']

NN-rank 20: ['C=CC(=O)C1C(C)(C)CCCC1(C)C']

EI-MS matched molecular weight (relative abundance: 8.39%) for C=CC(=O)C1C(C)(C)CCCC1(C)C

GHS hazard classification: unknown

NN-rank 21: ['C', 'CC1=C2C(=O)C=CC1CCC2(C)C']

NN-rank 22: ['CC=CC(O)C1=C(C)CCCC1(C)C']

EI-MS matched molecular weight (relative abundance: 8.39%) for CC=CC(O)C1=C(C)CCCC1(C)C

GHS hazard classification: none of the hazards selected for correlation

NN-rank 23: ['CC=CC(=O)C1C(C)=C=CCC1(C)C']

NN-rank 24: ['C=C1CC(=O)C2C(C)(C)CCCC12C']

NN-rank 25: ['C', 'CC1(C)CCCC2(C)C=CC(=O)C12']

EI-MS matched molecular weight (relative abundance: 99.99%) for CC1(C)CCCC2(C)C=CC(=O)C12

GHS hazard classification: none of the hazards selected for correlation

NN/MS matches corresponding with tox GHS classification highlight in Bold font.

57. Beta-Ionone: CC1=C(C(CCC1)(C)C)/C=C/C(=O)C

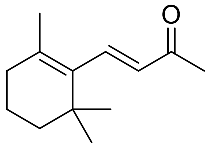

NN-rank 1: ['CC1=C(/C=C/C(C)O)C(C)(C)CCC1']

NN-rank 2: ['CC(=O)/C=C/C1C(C)=CCCC1(C)C']

NN-rank 3: ['CC(=O)/C=C/C1C(C)CCCC1(C)C']

NN-rank 4: ['CCCCCC(C)(C)C/C=C/C(C)=O']

NN-rank 5: ['CCCC(C)=C(/C=C/C(C)=O)C(C)C']

NN-rank 6: ['C', 'CC(=O)/C=C/C1=C(C)CCCC1C']

EI-MS matched molecular weight (relative abundance: 99.99%) for CC(=O)/C=C/C1=C(C)CCCC1C

GHS hazard classification: none of the hazards selected for correlation

NN-rank 7: ['C', 'CC(=O)/C=C/C1=C(C)CCCC1C']

EI-MS matched molecular weight (relative abundance: 99.99%) for CC(=O)/C=C/C1=C(C)CCCC1C

GHS hazard classification: none of the hazards selected for correlation

NN-rank 8: ['CC(=O)/C=C/CC(C)CCCC(C)C']

NN-rank 9: ['CC(=O)CCC1=C(C)CCCC1(C)C']

NN-rank 10: ['CC(=O)/C=C/C=C(C)CCCC(C)C']

NN-rank 11: ['CC=CCCC(C)(C)C/C=C/C(C)=O']

NN-rank 12: ['CC(=O)/C=C/CC(C)=CCCC(C)C']

NN-rank 13: ['CCCC(C)(C)C(/C=C/C(C)=O)CC']

NN-rank 14: ['CC1(C)CCCC2(C)CC(=O)C=CC12']

NN-rank 15: ['C=C(C)C(/C=C/C(C)=O)C(C)(C)CC']

NN-rank 16: ['C', 'CCCC(C)=C(/C=C/C(C)=O)CC']

NN-rank 17: ['C', 'CCCC(C)=C(/C=C/C(C)=O)CC']

NN-rank 18: ['CC/C=C/C1=C(C)CCCC1(C)C', 'O']

EI-MS matched molecular weight (relative abundance: 99.99%) for CC/C=C/C1=C(C)CCCC1(C)C

GHS hazard classification: none of the hazards selected for correlation

NN-rank 19: ['CC=C(/C=C/C(C)=O)C(C)(C)CCC']

NN-rank 20: ['C/C=C/C(C)=O', 'CCCCCC(C)C']

NN/MS matches corresponding with tox GHS classification highlight in Bold font.

NN-rank 21: ['CCC', 'CCCC(C)=C/C=C/C(C)=O']

EI-MS matched molecular weight (relative abundance: 42.44%) for CCC

GHS hazard classification: none of the hazards selected for correlation

NN-rank 22: ['C=C1CCCC(C)(C)C1/C=C/C(C)=O']

NN-rank 23: ['CCC', 'CCCC(C)C/C=C/C(C)=O']

EI-MS matched molecular weight (relative abundance: 42.44%) for CCC

GHS hazard classification: none of the hazards selected for correlation

NN-rank 24: ['C', 'C', 'CC(=O)/C=C/C1=C(C)CCCC1']

NN-rank 25: ['CCCC(C)(C)C1=C(C)CC(=O)C=C1']

NN/MS matches corresponding with tox GHS classification highlight in Bold font.

58. beta-Pinene: CC1(C2CCC(=C)C1C2)C

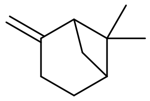

NN-rank 1: ['CC1=CCC2CC1C2(C)C']

NN-rank 2: ['CC1CCC2CC1C2(C)C']

NN-rank 3: ['CC1=C2CC(CC1)C2(C)C']

NN-rank 4: ['CC1(C)C2CC3CC3C1C2']

NN-rank 5: ['C=C(C)C1CC(C)C1(C)C']

NN-rank 6: ['CC1(C)C2CC3=C(C3)C1C2']

NN-rank 7: ['CC1(C)C2CCC3CC2C31']

NN-rank 8: ['CC1(C)C2CC1C1CC2C1']

NN-rank 9: ['C=C1C=CC2CC1C2(C)C']

NN-rank 10: ['C=C1CCC2CC1C2(C)C']

NN-rank 11: ['CC1=C=CC2CC1C2(C)C']

NN-rank 12: ['CC1CC(C2CC2)C1(C)C']

NN-rank 13: ['CC1(C)C2=C3CC3CC1C2']

NN-rank 14: ['CC1CCC2CC1C2(C)C']

NN-rank 15: ['CC1(C)C2CC3C=C3C1C2']

NN-rank 16: ['CC1(C)C2C=C3CC3C1C2']

NN-rank 17: ['CC1(C)C2CC1C1C=C2C1']

NN-rank 18: ['CC1(C)C2CC=C3CC2C31']

NN-rank 19: ['CCC1CC(CC)C1(C)C']

NN-rank 20: ['CC1(C)C2CCC3CC31C2']

NN-rank 21: ['C=C1CCC2C=C1C2(C)C']

NN-rank 22: ['C=C(C)C1CC(C)C1(C)C']

NN-rank 23: ['C', 'CC1(C)C2CCCC1C2']

NN-rank 24: ['CC1(C)C2=C3CC(C3)C1C2']

NN-rank 25: ['CC1=C2CC(CC1)C2(C)C']

NN/MS matches corresponding with tox GHS classification highlight in Bold font.

59. Butyl Acetate: CCCCOC(=O)C

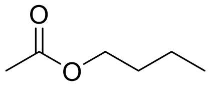

Summary Report of GHS Classification for NN/MS Matches:

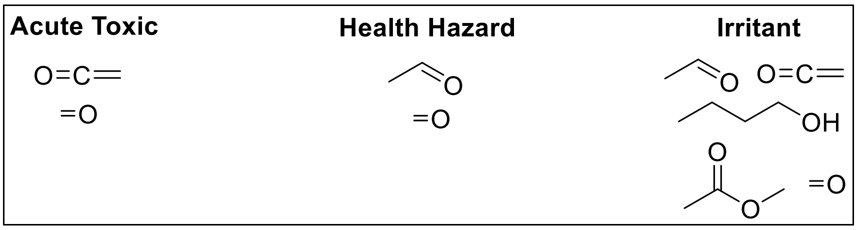

NN-rank 1: ['CC=O', 'CCCCO']

EI-MS matched molecular weight (relative abundance: 99.99%) for CC=O

GHS hazard classification: Health Hazard, Irritant

EI-MS matched molecular weight (relative abundance: 18.12%) for CCCCO

GHS hazard classification: Irritant

NN-rank 2: ['CC=O', 'CCCC=O']

EI-MS matched molecular weight (relative abundance: 99.99%) for CC=O

GHS hazard classification: Health Hazard, Irritant

NN-rank 3: ['CCC', 'COC(C)=O']

EI-MS matched molecular weight (relative abundance: 99.99%) for CCC

GHS hazard classification: none of the hazards selected for correlation

EI-MS matched molecular weight (relative abundance: 18.12%) for COC(C)=O

GHS hazard classification: Irritant

NN-rank 4: ['CC(=O)O', 'CCCC']

NN-rank 5: ['CCCCO', 'CCO']

EI-MS matched molecular weight (relative abundance: 18.12%) for CCCCO

GHS hazard classification: Irritant

NN-rank 6: ['C', 'CCCOC(C)=O']

EI-MS matched molecular weight (relative abundance: 7.51%) for C

GHS hazard classification: none of the hazards selected for correlation

NN-rank 7: ['C=C=O', 'CCCCO']

EI-MS matched molecular weight (relative abundance: 18.92%) for C=C=O

GHS hazard classification: Acute Toxic, Irritant

EI-MS matched molecular weight (relative abundance: 18.12%) for CCCCO

NN/MS matches corresponding with tox GHS classification highlight in Bold font.

GHS hazard classification: Irritant

NN-rank 8: ['CC', 'CCOC(C)=O']

EI-MS matched molecular weight (relative abundance: 12.91%) for CC

GHS hazard classification: none of the hazards selected for correlation

NN-rank 9: ['CC(=O)CCCCO']

NN-rank 10: ['C1CCOC1', 'CC=O']

EI-MS matched molecular weight (relative abundance: 99.99%) for CC=O

GHS hazard classification: Health Hazard, Irritant

NN-rank 11: ['CC(C)=O', 'CCCO']

NN-rank 12: ['CCCCOCC=O']

NN-rank 13: ['CCCCOC(C)O']

NN-rank 14: ['CC(=O)O', 'C=CCC']

EI-MS matched molecular weight (relative abundance: 7.21%) for C=CCC

GHS hazard classification: none of the hazards selected for correlation

NN-rank 15: ['CC=O', 'CCCCO']

EI-MS matched molecular weight (relative abundance: 99.99%) for CC=O

GHS hazard classification: Health Hazard, Irritant

EI-MS matched molecular weight (relative abundance: 18.12%) for CCCCO

GHS hazard classification: Irritant

NN-rank 16: ['CC=O', 'CCCOC']

EI-MS matched molecular weight (relative abundance: 99.99%) for CC=O

GHS hazard classification: Health Hazard, Irritant

EI-MS matched molecular weight (relative abundance: 18.12%) for CCCOC

GHS hazard classification: none of the hazards selected for correlation

NN-rank 17: ['C', 'CCCCOC=O']

EI-MS matched molecular weight (relative abundance: 7.51%) for C

GHS hazard classification: none of the hazards selected for correlation

NN-rank 18: ['C', 'C=O', 'CCCCO']

EI-MS matched molecular weight (relative abundance: 7.51%) for C

GHS hazard classification: none of the hazards selected for correlation

EI-MS matched molecular weight (relative abundance: 12.91%) for C=O

GHS hazard classification: Acute Toxic, Health Hazard, Irritant

EI-MS matched molecular weight (relative abundance: 18.12%) for CCCCO

GHS hazard classification: Irritant

NN-rank 19: ['CCC=COC(C)=O']

NN/MS matches corresponding with tox GHS classification highlight in Bold font.

NN-rank 20: ['CCCC', 'O=CCO']

NN-rank 21: ['C=O', 'CC=O', 'CCC']

EI-MS matched molecular weight (relative abundance: 12.91%) for C=O

GHS hazard classification: Acute Toxic, Health Hazard, Irritant

EI-MS matched molecular weight (relative abundance: 99.99%) for CC=O

GHS hazard classification: Health Hazard, Irritant

EI-MS matched molecular weight (relative abundance: 99.99%) for CCC

GHS hazard classification: none of the hazards selected for correlation

NN-rank 22: ['CCCC=O', 'CCO']

NN-rank 23: ['C=CO', 'CCCCO']

EI-MS matched molecular weight (relative abundance: 99.99%) for C=CO

GHS hazard classification: Acute Toxic

EI-MS matched molecular weight (relative abundance: 18.12%) for CCCCO

GHS hazard classification: Irritant

NN-rank 24: ['CC', 'C=COC(C)=O']

EI-MS matched molecular weight (relative abundance: 12.91%) for CC

GHS hazard classification: none of the hazards selected for correlation

NN-rank 25: ['CCOCCCCO']

NN/MS matches corresponding with tox GHS classification highlight in Bold font.

60. Butyl Butyrate: CCCCOC(=O)CCC

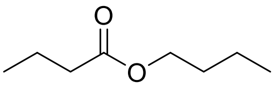

Summary Report of GHS Classification for NN/MS Matches:

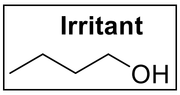

NN-rank 1: ['CCCC', 'CCCC(=O)O']

EI-MS matched molecular weight (relative abundance: 18.39%) for CCCC

GHS hazard classification: none of the hazards selected for correlation

NN-rank 2: ['CCCC', 'CCCC(=O)O']

EI-MS matched molecular weight (relative abundance: 18.39%) for CCCC

GHS hazard classification: none of the hazards selected for correlation

NN-rank 3: ['CCCC', 'CCCC=O', 'O']

EI-MS matched molecular weight (relative abundance: 18.39%) for CCCC

GHS hazard classification: none of the hazards selected for correlation

EI-MS matched molecular weight (relative abundance: 99.99%) for CCCC=O

GHS hazard classification: none of the hazards selected for correlation

NN-rank 4: ['CCCCO', 'CCCCO']

EI-MS matched molecular weight (relative abundance: 7.29%) for CCCCO

GHS hazard classification: Irritant

EI-MS matched molecular weight (relative abundance: 7.29%) for CCCCO

GHS hazard classification: Irritant

NN-rank 5: ['CCCC=O', 'CCCCO']

EI-MS matched molecular weight (relative abundance: 99.99%) for CCCC=O

GHS hazard classification: none of the hazards selected for correlation

EI-MS matched molecular weight (relative abundance: 7.29%) for CCCCO

GHS hazard classification: Irritant

NN-rank 6: ['C', 'CCC', 'CCCC(=O)O']

EI-MS matched molecular weight (relative abundance: 57.09%) for CCC

GHS hazard classification: none of the hazards selected for correlation

NN-rank 7: ['CCC', 'CCCC(=O)OC']

EI-MS matched molecular weight (relative abundance: 57.09%) for CCC

GHS hazard classification: none of the hazards selected for correlation

NN/MS matches corresponding with tox GHS classification highlight in Bold font.

NN-rank 8: ['C', 'CCC', 'CCCC(=O)O']

EI-MS matched molecular weight (relative abundance: 57.09%) for CCC

GHS hazard classification: none of the hazards selected for correlation

NN-rank 9: ['C', 'CCCOC(=O)CCC']

NN-rank 10: ['C', 'CCCCOC(=O)CC']

NN-rank 11: ['CC', 'CCCC(=O)OCC']

EI-MS matched molecular weight (relative abundance: 20.99%) for CC

GHS hazard classification: none of the hazards selected for correlation

NN-rank 12: ['CC1CCCCOC(=O)C1']

NN-rank 13: ['CCCCOCCCC', 'O']

NN-rank 14: ['CCCOC(=O)CC(C)C']

NN-rank 15: ['C', 'CCC', 'CCCC=O', 'O']

EI-MS matched molecular weight (relative abundance: 57.09%) for CCC

GHS hazard classification: none of the hazards selected for correlation

EI-MS matched molecular weight (relative abundance: 99.99%) for CCCC=O

GHS hazard classification: none of the hazards selected for correlation

NN-rank 16: ['CCCCC(=O)OCCC']

NN-rank 17: ['O=C1CCCCCCCO1']

NN-rank 18: ['CCCCCOC(=O)CC']

NN-rank 19: ['CCC', 'CCCC', 'O=CO']

EI-MS matched molecular weight (relative abundance: 57.09%) for CCC

GHS hazard classification: none of the hazards selected for correlation

EI-MS matched molecular weight (relative abundance: 18.39%) for CCCC

GHS hazard classification: none of the hazards selected for correlation

NN-rank 20: ['CCCC', 'CCCCO', 'O']

EI-MS matched molecular weight (relative abundance: 18.39%) for CCCC

GHS hazard classification: none of the hazards selected for correlation

EI-MS matched molecular weight (relative abundance: 7.29%) for CCCCO

GHS hazard classification: Irritant

NN-rank 21: ['CCCC', 'CCCCO', 'O']

EI-MS matched molecular weight (relative abundance: 18.39%) for CCCC

GHS hazard classification: none of the hazards selected for correlation

EI-MS matched molecular weight (relative abundance: 7.29%) for CCCCO

GHS hazard classification: Irritant

NN-rank 22: ['CCCOC(=O)C(C)CC']

NN/MS matches corresponding with tox GHS classification highlight in Bold font.

NN-rank 23: ['CC1CCCC(=O)OCC1']

NN-rank 24: ['CCC(=O)OCCC(C)C']

NN-rank 25: ['CCC1CCCCOC1=O']

NN/MS matches corresponding with tox GHS classification highlight in Bold font.

61. Butyl Butyryl Lactate: CCCCOC(=O)C(C)OC(=O)CCC

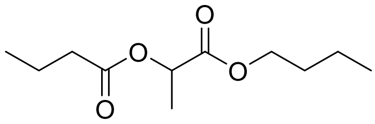

Summary Report of GHS Classification for NN/MS Matches:

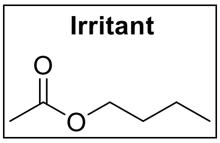

NN-rank 1: ['CCCC', 'CCCC(=O)OC(C)C(=O)O']

EI-MS matched molecular weight (relative abundance: 9.34%) for CCCC

GHS hazard classification: none of the hazards selected for correlation

NN-rank 2: ['CCCC(=O)O', 'CCCCOC(=O)CC']

NN-rank 3: ['CCCC=O', 'CCCCOC(=O)C(C)O']

EI-MS matched molecular weight (relative abundance: 99.99%) for CCCC=O

GHS hazard classification: none of the hazards selected for correlation

NN-rank 4: ['CCC', 'CCCC(=O)OC(C)C(=O)OC']

EI-MS matched molecular weight (relative abundance: 51.37%) for CCC

GHS hazard classification: none of the hazards selected for correlation

NN-rank 5: ['CCCC', 'CCCC(=O)OC(C)C(=O)O']

EI-MS matched molecular weight (relative abundance: 9.34%) for CCCC

GHS hazard classification: none of the hazards selected for correlation

NN-rank 6: ['CCCC(=O)OC(C)CO', 'CCCCO']

NN-rank 7: ['C', 'CCCOC(=O)C(C)OC(=O)CCC']

NN-rank 8: ['C', 'CCCCOC(=O)C(C)OC(=O)CC']

NN-rank 9: ['CCCC(=O)OC(C)C=O', 'CCCCO']

NN-rank 10: ['CC1CCCCOC(=O)C(C)OC(=O)C1']

NN-rank 11: ['CCCOC(=O)C(C)OC(=O)CC(C)C']

NN-rank 12: ['CC', 'CCCC(=O)OC(C)C(=O)OCC']

EI-MS matched molecular weight (relative abundance: 18.74%) for CC

GHS hazard classification: none of the hazards selected for correlation

NN-rank 13: ['CCCC', 'CCCC(=O)OC(C)C=O', 'O']

EI-MS matched molecular weight (relative abundance: 9.34%) for CCCC

GHS hazard classification: none of the hazards selected for correlation

NN/MS matches corresponding with tox GHS classification highlight in Bold font.

NN-rank 14: ['C', 'CCCCOC(=O)COC(=O)CCC']

NN-rank 15: ['CCCCO', 'CCCCOC(=O)C(C)O']

NN-rank 16: ['CC', 'CCCCOC(=O)C(C)OC(C)=O']

EI-MS matched molecular weight (relative abundance: 18.74%) for CC

GHS hazard classification: none of the hazards selected for correlation

NN-rank 17: ['CCCCOC(=O)C(C)OCCCC', 'O']

NN-rank 18: ['CCCCOCC(C)OC(=O)CCC', 'O']

NN-rank 19: ['C', 'CC1OC(=O)CCCCCCOC1=O']

NN-rank 20: ['C', 'CCC', 'CCCC(=O)OC(C)C(=O)O']

EI-MS matched molecular weight (relative abundance: 51.37%) for CCC

GHS hazard classification: none of the hazards selected for correlation

NN-rank 21: ['C', 'CCC', 'CCCC(=O)OC(C)C(=O)O']

EI-MS matched molecular weight (relative abundance: 51.37%) for CCC

GHS hazard classification: none of the hazards selected for correlation

NN-rank 22: ['CCCC', 'CCCCOC(=O)C(C)O', 'O']

EI-MS matched molecular weight (relative abundance: 9.34%) for CCCC

GHS hazard classification: none of the hazards selected for correlation

NN-rank 23: ['CCCCCCOC(=O)C(C)OC(C)=O']

NN-rank 24: ['C', 'CCCC(=O)O', 'CCCCOC(C)=O']

EI-MS matched molecular weight (relative abundance: 13.72%) for CCCCOC(C)=O

GHS hazard classification: Irritant

NN-rank 25: ['C', 'CC', 'CCCC(=O)OC(C)C(=O)OC']

EI-MS matched molecular weight (relative abundance: 18.74%) for CC

GHS hazard classification: none of the hazards selected for correlation

NN/MS matches corresponding with tox GHS classification highlight in Bold font.

62. Butyric Acid: CCCC(=O)O

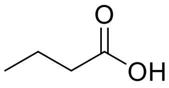

Summary Report of GHS Classification for NN/MS Matches:

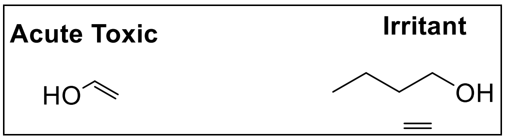

NN-rank 1: ['CC', 'CC(=O)O']

EI-MS matched molecular weight (relative abundance: 8.09%) for CC

GHS hazard classification: none of the hazards selected for correlation

NN-rank 2: ['CCCC(=O)O']

NN-rank 3: ['C', 'CCC(=O)O']

EI-MS matched molecular weight (relative abundance: 32.49%) for CCC(=O)O

GHS hazard classification: none of the hazards selected for correlation

NN-rank 4: ['CCCCO', 'O']

EI-MS matched molecular weight (relative abundance: 32.49%) for CCCCO

GHS hazard classification: Irritant

NN-rank 5: ['CCCCO', 'O']

EI-MS matched molecular weight (relative abundance: 32.49%) for CCCCO

GHS hazard classification: Irritant

NN-rank 6: ['CCC', 'O=CO']

EI-MS matched molecular weight (relative abundance: 14.09%) for CCC

GHS hazard classification: none of the hazards selected for correlation

EI-MS matched molecular weight (relative abundance: 9.89%) for O=CO

GHS hazard classification: none of the hazards selected for correlation

NN-rank 7: ['CC=CC(=O)O']

NN-rank 8: ['C=CCC(=O)O']

NN-rank 9: ['CCCC=O', 'O']

NN-rank 10: ['O=C1CCCO1']

NN-rank 11: ['CCCC=O', 'O']

NN-rank 12: ['CCC=CO', 'O']

NN-rank 13: ['CCC1OC1=O']

NN-rank 14: ['C', 'C=CC(=O)O']

NN/MS matches corresponding with tox GHS classification highlight in Bold font.

NN-rank 15: ['CCC', 'O=CO']

EI-MS matched molecular weight (relative abundance: 14.09%) for CCC

GHS hazard classification: none of the hazards selected for correlation

EI-MS matched molecular weight (relative abundance: 9.89%) for O=CO

GHS hazard classification: none of the hazards selected for correlation

NN-rank 16: ['CC1CC(=O)O1']

NN-rank 17: ['CC1CC1=O', 'O']

NN-rank 18: ['CC', 'O=C1CO1']

EI-MS matched molecular weight (relative abundance: 8.09%) for CC

GHS hazard classification: none of the hazards selected for correlation

NN-rank 19: ['CCC1OC1=O']

NN-rank 20: ['O=C(O)C1CC1']

NN-rank 21: ['CC', 'O=C1CO1']

EI-MS matched molecular weight (relative abundance: 8.09%) for CC

GHS hazard classification: none of the hazards selected for correlation

NN-rank 22: ['C=CO', 'CC', 'O']

EI-MS matched molecular weight (relative abundance: 14.09%) for C=CO

GHS hazard classification: Acute Toxic

EI-MS matched molecular weight (relative abundance: 8.09%) for CC

GHS hazard classification: none of the hazards selected for correlation

NN-rank 23: ['CCC(=O)OC']

NN-rank 24: ['C=C', 'CC(=O)O']

EI-MS matched molecular weight (relative abundance: 13.59%) for C=C

GHS hazard classification: Irritant

NN-rank 25: ['CCCC', 'O', 'O']

NN/MS matches corresponding with tox GHS classification highlight in Bold font.

63. Vanillin: COC1=C(C=CC(=C1)C=O)O

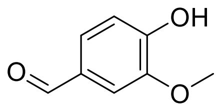

Summary Report of GHS Classification for NN/MS Matches:

Irritant

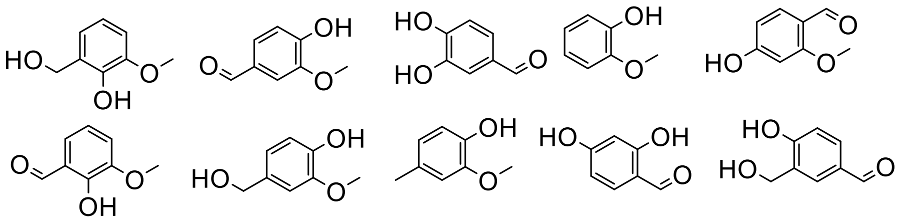

NN-rank 1: ['C', 'O=Cc1ccc(O)c(O)c1']

EI-MS matched molecular weight (relative abundance: 5.6%) for O=Cc1ccc(O)c(O)c1

GHS hazard classification: Irritant

NN-rank 2: ['C=O', 'COc1ccccc1O']

EI-MS matched molecular weight (relative abundance: 18.5%) for COc1ccccc1O

GHS hazard classification: Irritant

NN-rank 3: ['COc1cc(CO)ccc1O']

EI-MS matched molecular weight (relative abundance: 8.9%) for COc1cc(CO)ccc1O

GHS hazard classification: Irritant

NN-rank 4: ['CO', 'O=Cc1ccc(O)cc1']

NN-rank 5: ['C', 'O=Cc1cc2oc1cc2O']

NN-rank 6: ['C', 'O=Cc1cc2c(O)c(c1)O2']

NN-rank 7: ['COc1cc(C)ccc1O', 'O']

EI-MS matched molecular weight (relative abundance: 5.6%) for COc1cc(C)ccc1O

GHS hazard classification: Irritant

NN-rank 8: ['Cc1cc(C=O)cc(O)c1O']

EI-MS matched molecular weight (relative abundance: 99.99%) for Cc1cc(C=O)cc(O)c1O

GHS hazard classification: none of the hazards selected for correlation

NN-rank 9: ['COc1cc2cc(c1O)C2=O']

NN-rank 10: ['COc1cccc(C=O)c1', 'O']

NN-rank 11: ['COc1cc2cc(c1O)C2O']

NN/MS matches corresponding with tox GHS classification highlight in Bold font.

EI-MS matched molecular weight (relative abundance: 99.99%) for COc1cc2cc(c1O)C2O

GHS hazard classification: unknown

NN-rank 12: ['CO', 'COc1ccccc1O']

EI-MS matched molecular weight (relative abundance: 18.5%) for COc1ccccc1O

GHS hazard classification: Irritant

NN-rank 13: ['C', 'O=Cc1ccc(O)cc1O']

EI-MS matched molecular weight (relative abundance: 5.6%) for O=Cc1ccc(O)cc1O

GHS hazard classification: Irritant

NN-rank 14: ['COCc1ccc(O)c(O)c1']

EI-MS matched molecular weight (relative abundance: 8.9%) for COCc1ccc(O)c(O)c1

GHS hazard classification: none of the hazards selected for correlation

NN-rank 15: ['O=Cc1cc2c(O)c(c1)OC2']

NN-rank 16: ['COc1cccc(C=O)c1O']

EI-MS matched molecular weight (relative abundance: 99.99%) for COc1cccc(C=O)c1O

GHS hazard classification: Irritant

NN-rank 17: ['C', 'O=Cc1ccc(O)c(O)c1']

EI-MS matched molecular weight (relative abundance: 5.6%) for O=Cc1ccc(O)c(O)c1

GHS hazard classification: Irritant

NN-rank 18: ['COc1cc2cc(c1O)C2', 'O']

NN-rank 19: ['COc1cc(O)ccc1C=O']

EI-MS matched molecular weight (relative abundance: 99.99%) for COc1cc(O)ccc1C=O

GHS hazard classification: Irritant

NN-rank 20: ['C', 'O', 'O=Cc1ccc(O)cc1']

NN-rank 21: ['Oc1ccc2cc1OCOC2']

EI-MS matched molecular weight (relative abundance: 99.99%) for Oc1ccc2cc1OCOC2

GHS hazard classification: unknown

NN-rank 22: ['O=Cc1ccc(O)c(CO)c1']

EI-MS matched molecular weight (relative abundance: 99.99%) for O=Cc1ccc(O)c(CO)c1

GHS hazard classification: Irritant

NN-rank 23: ['COc1cc(C=O)ccc1O']

NN/MS matches corresponding with tox GHS classification highlight in Bold font.

EI-MS matched molecular weight (relative abundance: 99.99%) for COc1cc(C=O)ccc1O

GHS hazard classification: Irritant

NN-rank 24: ['C=O', 'O=Cc1ccc(O)cc1']

NN-rank 25: ['COc1cccc(CO)c1O']

EI-MS matched molecular weight (relative abundance: 8.9%) for COc1cccc(CO)c1O

GHS hazard classification: Irritant

NN/MS matches corresponding with tox GHS classification highlight in Bold font.

64. Carvone: CC1=CCC(CC1=O)C(=C)C

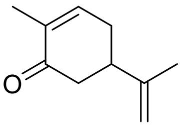

NN-rank 1: ['CC1=CCC(C(C)C)CC1=O']

NN-rank 2: ['C=C(C)C1=CC(=O)C(C)=CC1']

NN-rank 3: ['CC1=CCC(=C(C)C)CC1=O']

NN-rank 4: ['C=C(C)C1CCC(C)C(=O)C1']

NN-rank 5: ['C', 'C=C(C)C1CC=CC(=O)C1']

NN-rank 6: ['C', 'C=C(C)C1CCCC(=O)C1']

NN-rank 7: ['C=C(C)C1CC=C(C)C(O)C1']

NN-rank 8: ['C=C(C)C1=CC=C(C)C(=O)C1']

NN-rank 9: ['C=C1CC2=C(C)C(=O)CC1C2']

NN-rank 10: ['C=C(C)C1CC=C(C)C(=O)C1']

NN-rank 11: ['C=C(C)C(CC)CC(=O)CC']

NN-rank 12: ['C=C(C)CCC(=O)C(C)=CC']

NN-rank 13: ['C=C1CC2CC1CC(=O)C2C']

NN-rank 14: ['CC1=CC2=C(C)C(=O)CC1C2']

NN-rank 15: ['CC1=CCCCC1=O', 'C=CC']

EI-MS matched molecular weight (relative abundance: 22.8%) for C=CC

GHS hazard classification: none of the hazards selected for correlation

NN-rank 16: ['CC1=C2CC(C)C(CC1=O)C2']

NN-rank 17: ['CC=C(C)C(=O)CC=C(C)C']

NN-rank 18: ['C=C(C)C=CC(=O)C(C)=CC']

NN-rank 19: ['C=C(C)C1C=C(O)C(C)=CC1']

NN-rank 20: ['CC1=CCCCC1=O', 'CCC']

NN-rank 21: ['CC1=CCC=CC1=O', 'C=CC']

EI-MS matched molecular weight (relative abundance: 16.2%) for CC1=CCC=CC1=O

GHS hazard classification: none of the hazards selected for correlation

EI-MS matched molecular weight (relative abundance: 22.8%) for C=CC

GHS hazard classification: none of the hazards selected for correlation

NN-rank 22: ['C=C(C)CCC=C(C)C(C)=O']

NN-rank 23: ['C=C(C)C(CC=O)CC=CC']

NN-rank 24: ['C', 'C=C1CC2CC(=O)CC1C2']

NN/MS matches corresponding with tox GHS classification highlight in Bold font.

NN-rank 25: ['CC1CC2CC1CC(=O)C2C']

NN/MS matches corresponding with tox GHS classification highlight in Bold font.

65. Cassia oil-B: COC1=C(C=CC(=C1)CC=C)O

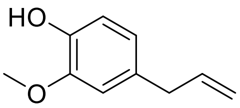

Summary Report of GHS Classification for NN/MS Matches:

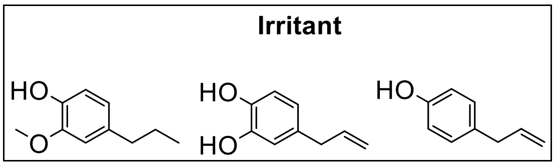

NN-rank 1: ['CC=Cc1ccc(O)c(OC)c1']

NN-rank 2: ['C', 'C=CCc1ccc(O)c(O)c1']

EI-MS matched molecular weight (relative abundance: 35.0%) for C=CCc1ccc(O)c(O)c1

GHS hazard classification: Irritant

NN-rank 3: ['C=C=Cc1ccc(O)c(OC)c1']

NN-rank 4: ['CCCc1ccc(O)c(OC)c1']

EI-MS matched molecular weight (relative abundance: 11.0%) for CCCc1ccc(O)c(OC)c1

GHS hazard classification: Irritant

NN-rank 5: ['COc1cc2c(cc1O)CC=C2']

NN-rank 6: ['C', 'Oc1ccc2cc1OCC=C2']

EI-MS matched molecular weight (relative abundance: 5.3%) for Oc1ccc2cc1OCC=C2

GHS hazard classification: unknown

NN-rank 7: ['COc1cc2cc(c1O)C(C)=C2']

NN-rank 8: ['COc1cc2c(cc1O)C(C)=C2']

NN-rank 9: ['COc1cc2cc(c1O)CC=C2']

NN-rank 10: ['COc1cc2ccc1OC(C)=C2']

NN-rank 11: ['COc1cc2cc(c1O)C(C)C2']

NN-rank 12: ['C', 'CC1=Cc2ccc(O)c(c2)O1']

EI-MS matched molecular weight (relative abundance: 5.3%) for CC1=Cc2ccc(O)c(c2)O1

GHS hazard classification: unknown

NN-rank 13: ['C=CC', 'COc1ccccc1O']

NN/MS matches corresponding with tox GHS classification highlight in Bold font.

EI-MS matched molecular weight (relative abundance: 5.6%) for C=CC

GHS hazard classification: none of the hazards selected for correlation

NN-rank 14: ['COc1cc2ccc1OC(C)C2']

NN-rank 15: ['COc1cc2c(cc1O)C(C)C2']

NN-rank 16: ['CC1=Cc2ccc(O)c(c2)OC1']

NN-rank 17: ['C', 'CC1Cc2ccc(O)c(c2)O1']

EI-MS matched molecular weight (relative abundance: 35.0%) for CC1Cc2ccc(O)c(c2)O1

GHS hazard classification: unknown

NN-rank 18: ['COc1cc2c(cc1O)CCC2']

NN-rank 19: ['C=C1Cc2cc(OC)c(O)c1c2']

NN-rank 20: ['C', 'Oc1ccc2cc1OCCC2']

EI-MS matched molecular weight (relative abundance: 35.0%) for Oc1ccc2cc1OCCC2

GHS hazard classification: unknown

NN-rank 21: ['C=C1Cc2cc(OC)c(O)cc21']

NN-rank 22: ['CC(C)=Cc1ccc(O)c(O)c1']

NN-rank 23: ['C=CCc1ccc(O)cc1', 'CO']

EI-MS matched molecular weight (relative abundance: 19.7%) for C=CCc1ccc(O)cc1

GHS hazard classification: Irritant

NN-rank 24: ['C=C=C', 'COc1ccccc1O']

EI-MS matched molecular weight (relative abundance: 12.6%) for C=C=C

GHS hazard classification: none of the hazards selected for correlation

NN-rank 25: ['C', 'OCC=Cc1ccc(O)cc1']

EI-MS matched molecular weight (relative abundance: 35.0%) for OCC=Cc1ccc(O)cc1

GHS hazard classification: none of the hazards selected for correlation

NN/MS matches corresponding with tox GHS classification highlight in Bold font.

66. Cedrol: C[C@@H]1CC[C@@H]2[C@]13CC[C@@]([C@H](C3)C2(C)C)(C)O

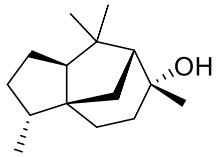

NN-rank 1: ['C[C]1CC[C]2C(C)(C)[C]3C[C@@]12CC=C3C', 'O']

NN-rank 2: ['C=C1CC[C@@]23C[C]1C(C)(C)[C]2CC[C]3C', 'O']

NN-rank 3: ['CC1(C)[C]2CC[C@](C)[C@@]23CC[C@](C)[C]1C3', 'O']

NN-rank 4: ['C[C]1CC[C]2C(C)(C)C3=C(C)CC[C@]12C3', 'O']

NN-rank 5: ['C[C]1CC[C]2C(C)(C)[C]3C[C@@]12C=C=C3C', 'O']

NN-rank 6: ['C=C(C)[C@]1C[C@]2(C)[C](CC[C@@]2C)C1(C)C', 'O']

NN-rank 7: ['C[C]1CC[C]2C(C)(C)C3=C[C@@]12CC[C@@]3(C)O']

NN-rank 8: ['C[C]=CC[C@@]12C[C]C(C)(C)[C]1CC[C]2C', 'O']

NN-rank 9: ['C[C]=CC[C@@]12C=[C]C(C)(C)[C]1CC[C]2C', 'O']

NN-rank 10: ['C', 'CC1(C)[C]2CC[C][C@@]23CC[C@@](C)(O)[C]1C3']

NN-rank 11: ['CC1=CC[C]2C(C)(C)[C]3C[C@]21CC[C@@]3(C)O']

NN-rank 12: ['C[C]1C=C[C]2C(C)(C)[C]3C[C@@]12CC[C@@]3(C)O']

NN-rank 13: ['C[C]1CC[C]2C(C)(C)[C]3C[C@@]12C=C[C@@]3(C)O']

NN-rank 14: ['C=[C]CC[C@@]12C[C]C(C)(C)[C]1CC[C]2C', 'O']

NN-rank 15: ['C=[C]CC[C@@]12C=[C]C(C)(C)[C]1CC[C]2C', 'O']

NN-rank 16: ['C[C]CC[C@@]12C=[C]C(C)(C)[C]1CC[C]2C', 'O']

NN-rank 17: ['C[C]CC[C@@]12C[C]C(C)(C)[C]1CC[C]2C', 'O']

NN-rank 18: ['C[C]1CC[C]2C(C)(C)[C]=C(C)CC[C@]12C', 'O']

NN-rank 19: ['C[C](O)CC[C@@]12C=[C]C(C)(C)[C]1CC[C]2C']

NN-rank 20: ['CC[C](C)[C@@]12[C]C(C)(C)[C](C1)[C@](C)(O)CC2']

NN-rank 21: ['C[C](O)CC[C@@]12C[C]C(C)(C)[C]1CC[C]2C']

NN-rank 22: ['C[C]=C=C[C@@]12C[C]C(C)(C)[C]1CC[C]2C', 'O']

NN-rank 23: ['C[C]1CC[C]2C(C)(C)[C]C[C@@]12CC=C(C)O']

NN-rank 24: ['C=C=CC[C@@]12C[C]C(C)(C)[C]1CC[C]2C', 'O']

NN-rank 25: ['C[C]1CC[C]2C(C)(C)[C]=C[C@@]12CC=C(C)O']

NN/MS matches corresponding with tox GHS classification highlight in Bold font.

67. Cinnamaldehyde: C1=CC=C(C=C1)/C=C/C=O

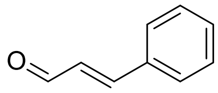

Summary Report of GHS Classification for NN/MS Matches:

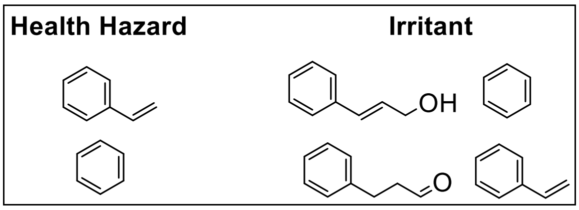

NN-rank 1: ['C=O', 'CCc1ccccc1']

NN-rank 2: ['O=CCCc1ccccc1']

EI-MS matched molecular weight (relative abundance: 5.7%) for O=CCCc1ccccc1

GHS hazard classification: Irritant

NN-rank 3: ['O=C=CCc1ccccc1']

EI-MS matched molecular weight (relative abundance: 99.99%) for O=C=CCc1ccccc1

GHS hazard classification: none of the hazards selected for correlation

NN-rank 4: ['O=C=C=Cc1ccccc1']

NN-rank 5: ['C=Cc1ccccc1', 'C=O']

EI-MS matched molecular weight (relative abundance: 58.49%) for C=Cc1ccccc1

GHS hazard classification: Health Hazard, Irritant

NN-rank 6: ['OC/C=C/c1ccccc1']

EI-MS matched molecular weight (relative abundance: 5.7%) for OC/C=C/c1ccccc1

GHS hazard classification: Irritant

NN-rank 7: ['CC=O', 'Cc1ccccc1']

NN-rank 8: ['C=CC=O', 'c1ccccc1']

EI-MS matched molecular weight (relative abundance: 42.6%) for c1ccccc1

GHS hazard classification: Health Hazard, Irritant

NN-rank 9: ['CCC=O', 'c1ccccc1']

EI-MS matched molecular weight (relative abundance: 42.6%) for c1ccccc1

GHS hazard classification: Health Hazard, Irritant

NN-rank 10: ['OC=CCc1ccccc1']

EI-MS matched molecular weight (relative abundance: 5.7%) for OC=CCc1ccccc1

GHS hazard classification: none of the hazards selected for correlation

NN/MS matches corresponding with tox GHS classification highlight in Bold font.

NN-rank 11: ['C=CCc1ccccc1', 'O']

NN-rank 12: ['CC=C=O', 'c1ccccc1']

EI-MS matched molecular weight (relative abundance: 42.6%) for c1ccccc1

GHS hazard classification: Health Hazard, Irritant

NN-rank 13: ['C=C=O', 'Cc1ccccc1']

NN-rank 14: ['CCc1ccccc1', 'CO']

NN-rank 15: ['c1ccc(C2CCO2)cc1']

EI-MS matched molecular weight (relative abundance: 5.7%) for c1ccc(C2CCO2)cc1

GHS hazard classification: none of the hazards selected for correlation

NN-rank 16: ['C=C=Cc1ccccc1', 'O']

NN-rank 17: ['C=O', 'CC', 'c1ccccc1']

EI-MS matched molecular weight (relative abundance: 42.6%) for c1ccccc1

GHS hazard classification: Health Hazard, Irritant

NN-rank 18: ['OC=C=Cc1ccccc1']

EI-MS matched molecular weight (relative abundance: 99.99%) for OC=C=Cc1ccccc1

GHS hazard classification: none of the hazards selected for correlation

NN-rank 19: ['C/C=C/c1ccccc1', 'O']

NN-rank 20: ['C1=C(c2ccccc2)OC1']

EI-MS matched molecular weight (relative abundance: 99.99%) for C1=C(c2ccccc2)OC1

GHS hazard classification: unknown

NN-rank 21: ['O=C1CC1c1ccccc1']

EI-MS matched molecular weight (relative abundance: 99.99%) for O=C1CC1c1ccccc1

GHS hazard classification: none of the hazards selected for correlation

NN-rank 22: ['O=CCC1C2=CC=C1C=C2']

EI-MS matched molecular weight (relative abundance: 99.99%) for O=CCC1C2=CC=C1C=C2

GHS hazard classification: unknown

NN-rank 23: ['OC1CC1c1ccccc1']

EI-MS matched molecular weight (relative abundance: 5.7%) for OC1CC1c1ccccc1

GHS hazard classification: none of the hazards selected for correlation

NN-rank 24: ['C1=CC(c2ccccc2)O1']

EI-MS matched molecular weight (relative abundance: 99.99%) for C1=CC(c2ccccc2)O1

NN/MS matches corresponding with tox GHS classification highlight in Bold font.

GHS hazard classification: unknown

NN-rank 25: ['O=CC=C1C2=CC=C1C=C2']

NN/MS matches corresponding with tox GHS classification highlight in Bold font.

68. Cinnamyl alcohol: C1=CC=C(C=C1)/C=C/CO

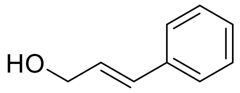

Summary Report of GHS Classification for NN/MS Matches:

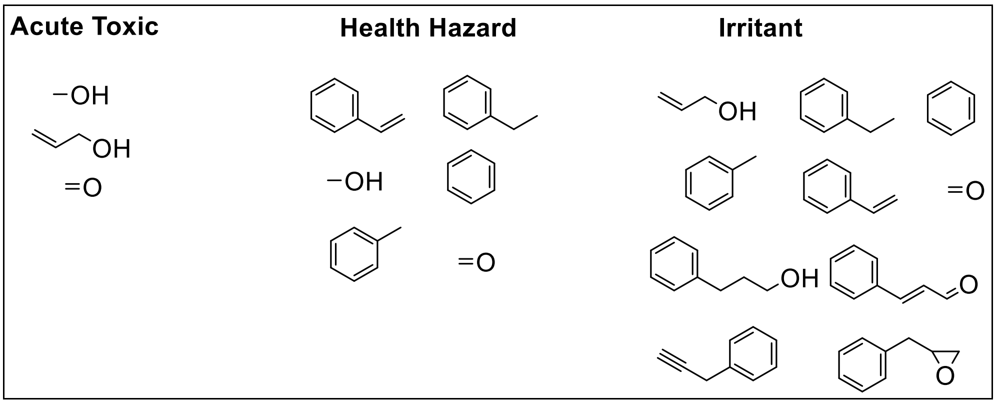

NN-rank 1: ['OCCCc1ccccc1']

EI-MS matched molecular weight (relative abundance: 6.09%) for OCCCc1ccccc1

GHS hazard classification: Irritant

NN-rank 2: ['O=C/C=C/c1ccccc1']

EI-MS matched molecular weight (relative abundance: 14.59%) for O=C/C=C/c1ccccc1

GHS hazard classification: Irritant

NN-rank 3: ['CCc1ccccc1', 'CO']

EI-MS matched molecular weight (relative abundance: 51.19%) for CCc1ccccc1

GHS hazard classification: Health Hazard, Irritant

EI-MS matched molecular weight (relative abundance: 7.69%) for CO

GHS hazard classification: Acute Toxic, Health Hazard

NN-rank 4: ['C=Cc1ccccc1', 'CO']

EI-MS matched molecular weight (relative abundance: 28.59%) for C=Cc1ccccc1

GHS hazard classification: Health Hazard, Irritant

EI-MS matched molecular weight (relative abundance: 7.69%) for CO

GHS hazard classification: Acute Toxic, Health Hazard

NN-rank 5: ['OC=CCc1ccccc1']

EI-MS matched molecular weight (relative abundance: 18.89%) for OC=CCc1ccccc1

GHS hazard classification: none of the hazards selected for correlation

NN/MS matches corresponding with tox GHS classification highlight in Bold font.

NN-rank 6: ['C=CCc1ccccc1', 'O']

EI-MS matched molecular weight (relative abundance: 16.09%) for C=CCc1ccccc1

GHS hazard classification: Health Hazard

NN-rank 7: ['C=CCO', 'c1ccccc1']

EI-MS matched molecular weight (relative abundance: 5.49%) for C=CCO

GHS hazard classification: Acute Toxic, Irritant

EI-MS matched molecular weight (relative abundance: 54.09%) for c1ccccc1

GHS hazard classification: Health Hazard, Irritant

NN-rank 8: ['C1=C(c2ccccc2)OC1']

EI-MS matched molecular weight (relative abundance: 14.59%) for C1=C(c2ccccc2)OC1

GHS hazard classification: unknown

NN-rank 9: ['OC=C=Cc1ccccc1']

EI-MS matched molecular weight (relative abundance: 14.59%) for OC=C=Cc1ccccc1

GHS hazard classification: none of the hazards selected for correlation

NN-rank 10: ['C/C=C/c1ccccc1', 'O']

EI-MS matched molecular weight (relative abundance: 16.09%) for C/C=C/c1ccccc1

GHS hazard classification: none of the hazards selected for correlation

NN-rank 11: ['c1ccc(C2CCO2)cc1']

EI-MS matched molecular weight (relative abundance: 18.89%) for c1ccc(C2CCO2)cc1

GHS hazard classification: none of the hazards selected for correlation

NN-rank 12: ['C=C=Cc1ccccc1', 'O']

EI-MS matched molecular weight (relative abundance: 45.19%) for C=C=Cc1ccccc1

GHS hazard classification: none of the hazards selected for correlation

NN-rank 13: ['CCO', 'Cc1ccccc1']

EI-MS matched molecular weight (relative abundance: 75.69%) for Cc1ccccc1

GHS hazard classification: Health Hazard, Irritant

NN-rank 14: ['c1ccc(CC2CO2)cc1']

EI-MS matched molecular weight (relative abundance: 18.89%) for c1ccc(CC2CO2)cc1

GHS hazard classification: Irritant

NN-rank 15: ['C(=C1/CO1)\\c1ccccc1']

EI-MS matched molecular weight (relative abundance: 14.59%) for C(=C1/CO1)\c1ccccc1

NN/MS matches corresponding with tox GHS classification highlight in Bold font.

GHS hazard classification: none of the hazards selected for correlation

NN-rank 16: ['C=O', 'CCc1ccccc1']

EI-MS matched molecular weight (relative abundance: 9.19%) for C=O

GHS hazard classification: Acute Toxic, Health Hazard, Irritant

EI-MS matched molecular weight (relative abundance: 51.19%) for CCc1ccccc1

GHS hazard classification: Health Hazard, Irritant

NN-rank 17: ['C1=Cc2ccccc21', 'CO']

EI-MS matched molecular weight (relative abundance: 7.69%) for CO

GHS hazard classification: Acute Toxic, Health Hazard

NN-rank 18: ['C1=Cc2ccccc21', 'CO']

EI-MS matched molecular weight (relative abundance: 7.69%) for CO

GHS hazard classification: Acute Toxic, Health Hazard

NN-rank 19: ['C=Cc1ccccc1', 'C=O']

EI-MS matched molecular weight (relative abundance: 28.59%) for C=Cc1ccccc1

GHS hazard classification: Health Hazard, Irritant

EI-MS matched molecular weight (relative abundance: 9.19%) for C=O

GHS hazard classification: Acute Toxic, Health Hazard, Irritant

NN-rank 20: ['C#CCc1ccccc1', 'O']

EI-MS matched molecular weight (relative abundance: 45.19%) for C#CCc1ccccc1

GHS hazard classification: Irritant

NN-rank 21: ['CCCO', 'c1ccccc1']

EI-MS matched molecular weight (relative abundance: 54.09%) for c1ccccc1

GHS hazard classification: Health Hazard, Irritant

NN-rank 22: ['O=C=C=Cc1ccccc1']

NN-rank 23: ['C/C=C(\\O)c1ccccc1']

EI-MS matched molecular weight (relative abundance: 18.89%) for C/C=C(\O)c1ccccc1

GHS hazard classification: none of the hazards selected for correlation

NN-rank 24: ['OCC1=Cc2ccccc21']

EI-MS matched molecular weight (relative abundance: 14.59%) for OCC1=Cc2ccccc21

GHS hazard classification: none of the hazards selected for correlation

NN-rank 25: ['OCC1=Cc2ccccc21']

EI-MS matched molecular weight (relative abundance: 14.59%) for

NN/MS matches corresponding with tox GHS classification highlight in Bold font.

OCC1=Cc2ccccc21

GHS hazard classification: none of the hazards selected for correlation

NN/MS matches corresponding with tox GHS classification highlight in Bold font.

69. Cis-3-hexenol: CC/C=C\CCO

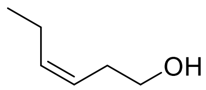

Summary Report of GHS Classification for NN/MS Matches:

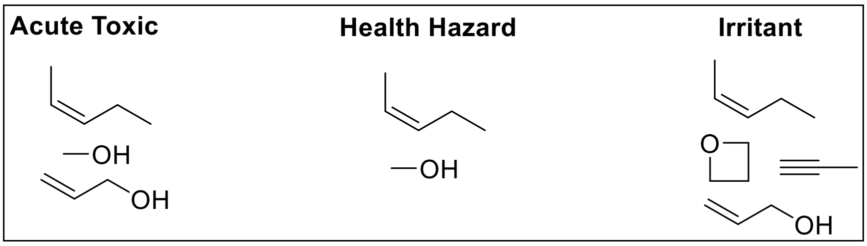

NN-rank 1: ['CC/C=C\\CC=O']

NN-rank 2: ['C', 'C/C=C\\CCO']

NN-rank 3: ['CCC', 'CCCO']

EI-MS matched molecular weight (relative abundance: 9.35%) for CCC

GHS hazard classification: none of the hazards selected for correlation

NN-rank 4: ['C/C=C\\CC', 'CO']

EI-MS matched molecular weight (relative abundance: 18.41%) for C/C=C\CC

GHS hazard classification: Acute Toxic, Health Hazard, Irritant

EI-MS matched molecular weight (relative abundance: 21.85%) for CO

GHS hazard classification: Acute Toxic, Health Hazard

NN-rank 5: ['CCCCCCO']

NN-rank 6: ['CCC1=CCCO1']

NN-rank 7: ['C=C/C=C\\CC', 'O']

EI-MS matched molecular weight (relative abundance: 7.73%) for C=C/C=C\CC

GHS hazard classification: none of the hazards selected for correlation

NN-rank 8: ['C=CC', 'CCCO']

EI-MS matched molecular weight (relative abundance: 75.02%) for C=CC

GHS hazard classification: none of the hazards selected for correlation

NN-rank 9: ['CC=C=CCCO']

NN-rank 10: ['C#CC', 'CCCO']

EI-MS matched molecular weight (relative abundance: 39.05%) for C#CC

GHS hazard classification: Irritant

NN-rank 11: ['CC=CCCCO']

NN-rank 12: ['C=CCCO', 'CC']

NN/MS matches corresponding with tox GHS classification highlight in Bold font.

EI-MS matched molecular weight (relative abundance: 13.84%) for CC

GHS hazard classification: none of the hazards selected for correlation

NN-rank 13: ['C', 'C=CCCCO']

NN-rank 14: ['CC/C=C\\CC', 'O']

NN-rank 15: ['CC/C=C1\\CCO1']

NN-rank 16: ['CCCOCCC']

NN-rank 17: ['CC/C=C\\C1CO1']

NN-rank 18: ['C=CCC', 'CCO']

EI-MS matched molecular weight (relative abundance: 38.21%) for C=CCC

GHS hazard classification: none of the hazards selected for correlation

NN-rank 19: ['CC#CCCCO']

NN-rank 20: ['CC/C=C\\C=CO']

NN-rank 21: ['CCCC=CCO']

NN-rank 22: ['C=CCO', 'CCC']

EI-MS matched molecular weight (relative abundance: 11.98%) for C=CCO

GHS hazard classification: Acute Toxic, Irritant

EI-MS matched molecular weight (relative abundance: 9.35%) for CCC

GHS hazard classification: none of the hazards selected for correlation

NN-rank 23: ['C', 'C=C=CCCO']

NN-rank 24: ['CC/C=C(/O)CC']

NN-rank 25: ['C1COC1', 'CCC']

EI-MS matched molecular weight (relative abundance: 11.98%) for C1COC1

GHS hazard classification: Irritant

EI-MS matched molecular weight (relative abundance: 9.35%) for CCC

GHS hazard classification: none of the hazards selected for correlation

NN/MS matches corresponding with tox GHS classification highlight in Bold font.

70. Cis-3-hexenyl Acetate: CC/C=C\CCOC(=O)C

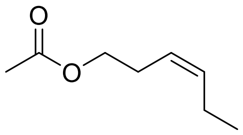

Summary Report of GHS Classification for NN/MS Matches:

Acute Toxic	Health Hazard	Irritant
O=C=	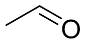	O=C= 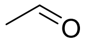

NN-rank 1: ['CC/C=C\\CCO', 'CC=O']

EI-MS matched molecular weight (relative abundance: 99.99%) for CC=O

GHS hazard classification: Health Hazard, Irritant

NN-rank 2: ['CC(=O)O', 'CC/C=C\\CC']

NN-rank 3: ['C', 'C/C=C\\CCOC(C)=O']

EI-MS matched molecular weight (relative abundance: 6.61%) for C

GHS hazard classification: none of the hazards selected for correlation

NN-rank 4: ['C/C=C\\CC', 'COC(C)=O']

NN-rank 5: ['CC/C=C\\CC=O', 'CC=O']

EI-MS matched molecular weight (relative abundance: 99.99%) for CC=O

GHS hazard classification: Health Hazard, Irritant

NN-rank 6: ['CCC1=CCCO1', 'CC=O']

EI-MS matched molecular weight (relative abundance: 99.99%) for CC=O

GHS hazard classification: Health Hazard, Irritant

NN-rank 7: ['CCC', 'CCCOC(C)=O']

EI-MS matched molecular weight (relative abundance: 99.99%) for CCC

GHS hazard classification: none of the hazards selected for correlation

NN-rank 8: ['CC(=O)O', 'C=C/C=C\\CC']

EI-MS matched molecular weight (relative abundance: 6.91%) for C=C/C=C\CC

GHS hazard classification: none of the hazards selected for correlation

NN-rank 9: ['CCCCCCOC(C)=O']

NN-rank 10: ['C', 'C=CCCCOC(C)=O']

EI-MS matched molecular weight (relative abundance: 6.61%) for C

GHS hazard classification: none of the hazards selected for correlation

NN-rank 11: ['CCCC=CCOC(C)=O']

NN/MS matches corresponding with tox GHS classification highlight in Bold font.

NN-rank 12: ['CC=C=CCCOC(C)=O']

NN-rank 13: ['CC=CCCCOC(C)=O']

NN-rank 14: ['CC/C=C\\C=COC(C)=O']

NN-rank 15: ['CCC=C=CCOC(C)=O']

NN-rank 16: ['CC', 'C=CCCOC(C)=O']

EI-MS matched molecular weight (relative abundance: 6.61%) for CC

GHS hazard classification: none of the hazards selected for correlation

NN-rank 17: ['C=CCOC(C)=O', 'CCC']

EI-MS matched molecular weight (relative abundance: 99.99%) for CCC

GHS hazard classification: none of the hazards selected for correlation

NN-rank 18: ['C=C=O', 'CC/C=C\\CCO']

EI-MS matched molecular weight (relative abundance: 21.22%) for C=C=O

GHS hazard classification: Acute Toxic, Irritant

NN-rank 19: ['C=CCC', 'CCOC(C)=O']

EI-MS matched molecular weight (relative abundance: 7.51%) for C=CCC

GHS hazard classification: none of the hazards selected for correlation

NN-rank 20: ['C=CC', 'CCCOC(C)=O']

EI-MS matched molecular weight (relative abundance: 21.22%) for C=CC

GHS hazard classification: none of the hazards selected for correlation

NN-rank 21: ['CC=O', 'CCCOCCC']

EI-MS matched molecular weight (relative abundance: 99.99%) for CC=O

GHS hazard classification: Health Hazard, Irritant

NN-rank 22: ['C=CCCC', 'COC(C)=O']

NN-rank 23: ['CC/C=C(/O)CC', 'CC=O']

EI-MS matched molecular weight (relative abundance: 99.99%) for CC=O

GHS hazard classification: Health Hazard, Irritant

NN-rank 24: ['C', 'C=C=CCCOC(C)=O']

EI-MS matched molecular weight (relative abundance: 6.61%) for C

GHS hazard classification: none of the hazards selected for correlation

NN-rank 25: ['CC=O', 'CCC1CCCO1']

EI-MS matched molecular weight (relative abundance: 99.99%) for CC=O

GHS hazard classification: Health Hazard, Irritant

NN/MS matches corresponding with tox GHS classification highlight in Bold font.

71. Cis-3-Hexenyl Butyrate: CCCC(=O)OCC/C=C\CC

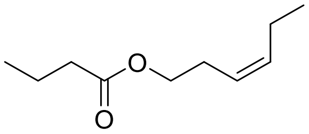

Summary Report of GHS Classification for NN/MS Matches:

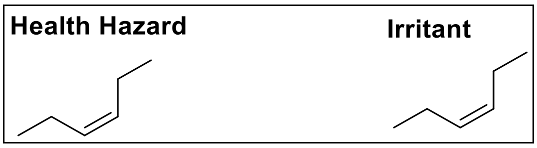

NN-rank 1: ['CC/C=C\\CC', 'CCCC(=O)O']

EI-MS matched molecular weight (relative abundance: 8.79%) for CC/C=C\CC

GHS hazard classification: Health Hazard, Irritant

NN-rank 2: ['CC/C=C\\CC', 'CCCC(=O)O']

EI-MS matched molecular weight (relative abundance: 8.79%) for CC/C=C\CC

GHS hazard classification: Health Hazard, Irritant

NN-rank 3: ['CC/C=C\\CCO', 'CCCCO']

NN-rank 4: ['CC/C=C\\CCO', 'CCCC=O']

EI-MS matched molecular weight (relative abundance: 79.99%) for CCCC=O

GHS hazard classification: none of the hazards selected for correlation

NN-rank 5: ['C/C=C\\CC', 'CCCC(=O)OC']

NN-rank 6: ['C', 'C/C=C\\CCOC(=O)CCC']

NN-rank 7: ['C', 'CC/C=C\\CCOC(=O)CC']

NN-rank 8: ['CCC', 'CCCOC(=O)CCC']

EI-MS matched molecular weight (relative abundance: 69.49%) for CCC

GHS hazard classification: none of the hazards selected for correlation

NN-rank 9: ['CC1CC/C=C\\CCOC(=O)C1']

NN-rank 10: ['CC/C=C\\CC', 'CCCC=O', 'O']

EI-MS matched molecular weight (relative abundance: 8.79%) for CC/C=C\CC

GHS hazard classification: Health Hazard, Irritant

EI-MS matched molecular weight (relative abundance: 79.99%) for CCCC=O

GHS hazard classification: none of the hazards selected for correlation

NN-rank 11: ['C/C=C\\CCOC(=O)CC(C)C']

NN-rank 12: ['CCCCCCOC(=O)CCC']

NN-rank 13: ['CC=C=CCCOC(=O)CCC']

NN/MS matches corresponding with tox GHS classification highlight in Bold font.

NN-rank 14: ['CC/C=C\\CCOCCCC', 'O']

NN-rank 15: ['C=CC', 'CCCOC(=O)CCC']

EI-MS matched molecular weight (relative abundance: 30.19%) for C=CC

GHS hazard classification: none of the hazards selected for correlation

NN-rank 16: ['C', 'C=CCCCOC(=O)CCC']

NN-rank 17: ['CCC1=CCCOC(=O)C1CC']

NN-rank 18: ['CC=CCCCOC(=O)CCC']

NN-rank 19: ['CC', 'C=CCCOC(=O)CCC']

EI-MS matched molecular weight (relative abundance: 6.09%) for CC

GHS hazard classification: none of the hazards selected for correlation

NN-rank 20: ['CCC', 'C=CCOC(=O)CCC']

EI-MS matched molecular weight (relative abundance: 69.49%) for CCC

GHS hazard classification: none of the hazards selected for correlation

NN-rank 21: ['CCCC=CCOC(=O)CCC']

NN-rank 22: ['C', 'C/C=C\\CC', 'CCCC(=O)O']

NN-rank 23: ['C=CCC', 'CCCC(=O)OCC']

EI-MS matched molecular weight (relative abundance: 13.69%) for C=CCC

GHS hazard classification: none of the hazards selected for correlation

NN-rank 24: ['CCC=C=CCOC(=O)CCC']

NN-rank 25: ['C', 'C/C=C\\CC', 'CCCC(=O)O']

NN/MS matches corresponding with tox GHS classification highlight in Bold font.

72. Cis-3-Hexenyl Isovalerate: CC/C=C\CCOC(=O)CC(C)C

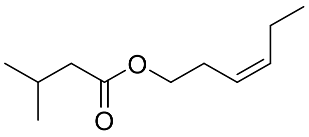

Summary Report of GHS Classification for NN/MS Matches:

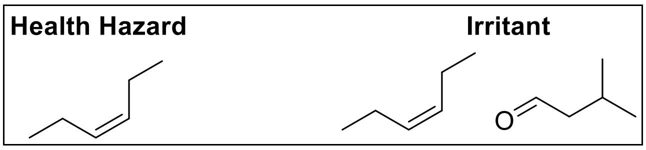

NN-rank 1: ['CC(C)CC(=O)O', 'CC/C=C\\CC']

EI-MS matched molecular weight (relative abundance: 16.19%) for CC/C=C\CC

GHS hazard classification: Health Hazard, Irritant

NN-rank 2: ['CC(C)CCO', 'CC/C=C\\CCO']

NN-rank 3: ['CC(C)CC(=O)O', 'CC/C=C\\CC']

EI-MS matched molecular weight (relative abundance: 16.19%) for CC/C=C\CC

GHS hazard classification: Health Hazard, Irritant

NN-rank 4: ['CC(C)CC=O', 'CC/C=C\\CCO']

EI-MS matched molecular weight (relative abundance: 58.29%) for CC(C)CC=O

GHS hazard classification: Irritant

NN-rank 5: ['C/C=C\\CC', 'COC(=O)CC(C)C']

NN-rank 6: ['C', 'C/C=C\\CCOC(=O)CC(C)C']

NN-rank 7: ['CCC=C(C(=O)OCCC)C(C)C']

NN-rank 8: ['CCC', 'CCCOC(=O)CC(C)C']

EI-MS matched molecular weight (relative abundance: 18.39%) for CCC

GHS hazard classification: none of the hazards selected for correlation

NN-rank 9: ['CC/C=C\\CCOC(=O)C=C(C)C']

NN-rank 10: ['CCC1=CCCOC(=O)C1C(C)C']

NN-rank 11: ['CCCCCCOC(=O)CC(C)C']

NN-rank 12: ['CC=C=CCCOC(=O)CC(C)C']

NN-rank 13: ['CCC1=C(C(C)C)C(=O)OCCC1']

NN-rank 14: ['CC=CCCCOC(=O)CC(C)C']

NN-rank 15: ['C', 'C=CCCCOC(=O)CC(C)C']

NN-rank 16: ['CC/C=C\\CCOCCC(C)C', 'O']

NN-rank 17: ['C=CCC', 'CCOC(=O)CC(C)C']

NN/MS matches corresponding with tox GHS classification highlight in Bold font.

EI-MS matched molecular weight (relative abundance: 25.99%) for C=CCC

GHS hazard classification: none of the hazards selected for correlation

NN-rank 18: ['CC', 'C=CCCOC(=O)CC(C)C']

EI-MS matched molecular weight (relative abundance: 19.69%) for CC

GHS hazard classification: none of the hazards selected for correlation

NN-rank 19: ['C=CC', 'CCCOC(=O)CC(C)C']

EI-MS matched molecular weight (relative abundance: 43.69%) for C=CC

GHS hazard classification: none of the hazards selected for correlation

NN-rank 20: ['CC(C)CC=O', 'CC/C=C\\CC', 'O']

EI-MS matched molecular weight (relative abundance: 58.29%) for CC(C)CC=O

GHS hazard classification: Irritant

EI-MS matched molecular weight (relative abundance: 16.19%) for CC/C=C\CC

GHS hazard classification: Health Hazard, Irritant

NN-rank 21: ['CCCC=CCOC(=O)CC(C)C']

NN-rank 22: ['CCCOC(=O)C(CCC)C(C)C']

NN-rank 23: ['C', 'CC(C)CC(=O)O', 'C/C=C\\CC']

NN-rank 24: ['CCC=C=CCOC(=O)CC(C)C']

NN-rank 25: ['C=CCOC(=O)CC(C)C', 'CCC']

EI-MS matched molecular weight (relative abundance: 18.39%) for CCC

GHS hazard classification: none of the hazards selected for correlation

NN/MS matches corresponding with tox GHS classification highlight in Bold font.

73. cis-6-Nonen-1-ol: CC/C=C\CCCCCO

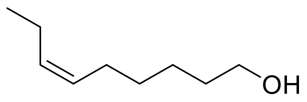

Summary Report of GHS Classification for NN/MS Matches:

Acute Toxic	Health Hazard	Irritant
—OH =O	—OH =O	=O

NN-rank 1: ['CC/C=C\\CCCCC=O']

NN-rank 2: ['C', 'C/C=C\\CCCCCO']

NN-rank 3: ['CCC', 'CCCCCCO']

EI-MS matched molecular weight (relative abundance: 18.03%) for CCC

GHS hazard classification: none of the hazards selected for correlation

NN-rank 4: ['CC/C=C\\CCCC', 'CO']

EI-MS matched molecular weight (relative abundance: 40.02%) for CO

GHS hazard classification: Acute Toxic, Health Hazard

NN-rank 5: ['CCCCCCCCCO']

NN-rank 6: ['CC/C1=C\\CCCCCO1']

NN-rank 7: ['C', 'C=CCCCCCCO']

NN-rank 8: ['CC/C=C1\\CCCCCO1']

NN-rank 9: ['C=CCCC/C=C\\CC', 'O']

NN-rank 10: ['CC=C=CCCCCCO']

NN-rank 11: ['CC=CCCCCCCO']

NN-rank 12: ['CC/C=C\\CCC', 'CCO']

NN-rank 13: ['C=CCCCCCO', 'CC']

EI-MS matched molecular weight (relative abundance: 29.49%) for CC

GHS hazard classification: none of the hazards selected for correlation

NN-rank 14: ['C1CCCOCC1', 'CCC']

EI-MS matched molecular weight (relative abundance: 18.03%) for CCC

GHS hazard classification: none of the hazards selected for correlation

NN-rank 15: ['CCCCCCOCCC']

NN-rank 16: ['C#CC', 'CCCCCCO']

EI-MS matched molecular weight (relative abundance: 39.89%) for C#CC

NN/MS matches corresponding with tox GHS classification highlight in Bold font.

GHS hazard classification: Irritant

NN-rank 17: ['C=CC', 'CCCCCCO']

EI-MS matched molecular weight (relative abundance: 99.99%) for C=CC

GHS hazard classification: none of the hazards selected for correlation

NN-rank 18: ['C', 'C#CCCCCCCO']

NN-rank 19: ['CC/C=C\\CCCCC', 'O']

NN-rank 20: ['CC/C=C\\CCCC=CO']

NN-rank 21: ['CC#CCCCCCCO']

NN-rank 22: ['C=O', 'CC/C=C\\CCCC']

EI-MS matched molecular weight (relative abundance: 29.49%) for C=O

GHS hazard classification: Acute Toxic, Health Hazard, Irritant

NN-rank 23: ['CCCC=CCCCCO']

NN-rank 24: ['CCCC1CCCCCO1']

NN-rank 25: ['CCC1CCCCCCO1']

NN/MS matches corresponding with tox GHS classification highlight in Bold font.

74. Citral: CC(=CCC/C(=C/C=O)/C)C

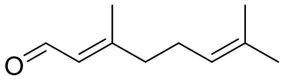

Summary Report of GHS Classification for NN/MS Matches:

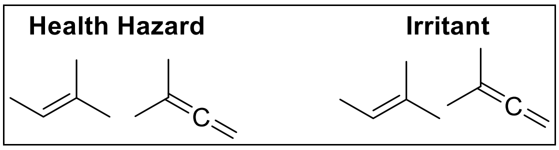

NN-rank 1: ['CC(C)=CCCC(C)CC=O']

NN-rank 2: ['C', 'CC=CCC/C(C)=C/C=O']

NN-rank 3: ['C', 'CC=CCC/C(C)=C/C=O']

NN-rank 4: ['CC(C)=C=CC/C(C)=C/C=O']

NN-rank 5: ['CC(C)=CCC/C(C)=C/CO']

NN-rank 6: ['CC(C)=CC=O', 'CC=C(C)C']

EI-MS matched molecular weight (relative abundance: 96.0%) for CC=C(C)C

GHS hazard classification: Health Hazard, Irritant

NN-rank 7: ['CC(C)=CCC=C(C)CC=O']

NN-rank 8: ['C', 'C', 'C=CCC/C(C)=C/C=O']

NN-rank 9: ['C=C(CC=O)CCC=C(C)C']

NN-rank 10: ['C/C(=C\\C=O)CCCC(C)C']

NN-rank 11: ['CC(C)=CC=C/C(C)=C/C=O']

NN-rank 12: ['C', 'CCCCC/C(C)=C/C=O']

NN-rank 13: ['C', 'CCCCC/C(C)=C/C=O']

NN-rank 14: ['C=C(C)CCC/C(C)=C/C=O']

NN-rank 15: ['C=C(C)CCC/C(C)=C/C=O']

NN-rank 16: ['CCC', 'CCC/C(C)=C/C=O']

NN-rank 17: ['CC(=C=C=O)CCC=C(C)C']

NN-rank 18: ['C', 'C=C=CCC/C(C)=C/C=O']

NN-rank 19: ['C', 'C=C=CCC/C(C)=C/C=O']

NN-rank 20: ['CC(C)=CC=O', 'C=C=C(C)C']

EI-MS matched molecular weight (relative abundance: 11.0%) for C=C=C(C)C

GHS hazard classification: Health Hazard, Irritant

NN-rank 21: ['C/C(=C\\C=O)CC=CC(C)C']

NN-rank 22: ['CC(C)=CCCC(C)C=C=O']

NN/MS matches corresponding with tox GHS classification highlight in Bold font.

NN-rank 23: ['C', 'C', 'CCCC/C(C)=C/C=O']

NN-rank 24: ['C/C=C(\\C)CCC=C(C)C', 'O']

NN-rank 25: ['C=CC/C(C)=C/C=O', 'CCC']

75. Citric Acid: C(C(=O)O)C(CC(=O)O)(C(=O)O)O

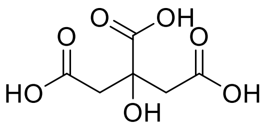

NN-rank 1: ['CC(=O)O', 'O=C(O)CC(=O)C(=O)O']

NN-rank 2: ['CC(=O)O', 'O=C(O)CC(=O)C(=O)O']

NN-rank 3: ['CC(=O)O', 'CC(=O)O', 'O=CC(=O)O']

NN-rank 4: ['CC(=O)O', 'O=C(O)CC(O)C(=O)O']

NN-rank 5: ['CC(=O)O', 'O=C(O)CC(O)C(=O)O']

NN-rank 6: ['O', 'O=C(O)C=C(CC(=O)O)C(=O)O']

NN-rank 7: ['O', 'O=C(O)C=C(CC(=O)O)C(=O)O']

NN-rank 8: ['CC(=O)O', 'O=C(O)C=C(O)C(=O)O']

NN-rank 9: ['CC(=O)O', 'O=C(O)C=C(O)C(=O)O']

NN-rank 10: ['CC(=O)O', 'CC(=O)O', 'O=C(O)CO']

NN-rank 11: ['O', 'O=C(O)CC(CC(=O)O)C(=O)O']

NN-rank 12: ['CC(=O)O', 'O', 'O=C(O)C=CC(=O)O']

NN-rank 13: ['CC(=O)O', 'O', 'O=C(O)C=CC(=O)O']

NN-rank 14: ['O=C(O)CC(O)(CC(=O)O)C(=O)O']

NN-rank 15: ['O=C(O)CC(O)(CC(=O)O)C(=O)O']

NN-rank 16: ['CC(=O)O', 'O', 'O=C(O)CCC(=O)O']

NN-rank 17: ['CC(=O)O', 'O', 'O=C(O)CCC(=O)O']

NN-rank 18: ['O', 'O=C(O)CC(O)(CO)CC(=O)O']

NN-rank 19: ['O=C(O)CC(O)(CC(=O)O)C(=O)O']

NN-rank 20: ['O', 'O=C(O)CC(O)(CCO)C(=O)O']

NN-rank 21: ['O=C(O)CC(O)(CC(=O)O)C(O)O']

NN-rank 22: ['CC(=O)O', 'CC(=O)O', 'CC(=O)O', 'O']

NN-rank 23: ['O', 'O=CC(O)(CC(=O)O)CC(=O)O']

NN-rank 24: ['O', 'O=C(O)CC(O)(CCO)C(=O)O']

NN-rank 25: ['O', 'O=C(O)CC(O)(CCO)C(=O)O']

NN/MS matches corresponding with tox GHS classification highlight in Bold font.

76. Citronellol: CC(CCC=C(C)C)CCO

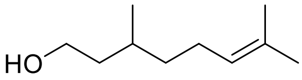

Summary Report of GHS Classification for NN/MS Matches:

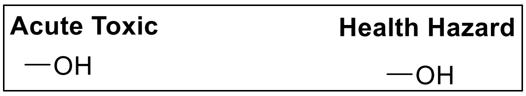

NN-rank 1: ['CC1CCCC(C)(C)OCC1']

NN-rank 2: ['CC(C)CCCC(C)CCO']

NN-rank 3: ['C', 'CCCCCC(C)CCO']

NN-rank 4: ['C', 'CCCCCC(C)CCO']

NN-rank 5: ['C=C(C)CCCC(C)CCO']

NN-rank 6: ['C=C(C)CCCC(C)CCO']

NN-rank 7: ['C=CC(C)CCC=C(C)C', 'O']

NN-rank 8: ['CC(C)=CCCC(C)CC=O']

NN-rank 9: ['C', 'CC=CCCC(C)CCO']

NN-rank 10: ['C', 'CC=CCCC(C)CCO']

NN-rank 11: ['CC(C)=CCCC(C)C', 'CO']

EI-MS matched molecular weight (relative abundance: 28.33%) for CO

GHS hazard classification: Acute Toxic, Health Hazard

NN-rank 12: ['C', 'C', 'CCCCC(C)CCO']

NN-rank 13: ['C', 'CC1=CCCC(C)CCO1']

NN-rank 14: ['C', 'CC1=CCCC(C)CCO1']

NN-rank 15: ['CC1CC=CC(C)(C)OCC1']

NN-rank 16: ['CC(C)C=CCC(C)CCO']

NN-rank 17: ['CCC(C)CCCC(C)(C)O']

NN-rank 18: ['C', 'C', 'C=CCCC(C)CCO']

NN-rank 19: ['C', 'CC1CCCC(C)OCC1']

NN-rank 20: ['C', 'CC1CCCC(C)OCC1']

NN-rank 21: ['CC(C)=CCCC(C)C=CO']

NN-rank 22: ['CC(C)=C=CCC(C)CCO']

NN-rank 23: ['CC1CCCC(C)(C)CC1', 'O']

NN-rank 24: ['CCC(C)CCC=C(C)C', 'O']

NN/MS matches corresponding with tox GHS classification highlight in Bold font.

NN-rank 25: ['CC(C)=CCCC(C)=CCO']

77. Citronellyl Acetate: CC(CCC=C(C)C)CCOC(=O)C

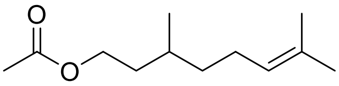

Summary Report of GHS Classification for NN/MS Matches:

Acute Toxic	Health Hazard	Irritant
O=C=	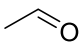	O=C= 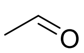

NN-rank 1: ['CC(C)=CCCC(C)CCO', 'CC=O']

EI-MS matched molecular weight (relative abundance: 99.99%) for CC=O

GHS hazard classification: Health Hazard, Irritant

NN-rank 2: ['CC(=O)OCCC(C)CCCC(C)C']

NN-rank 3: ['CC(C)=CCCC(C)CC=O', 'CC=O']

EI-MS matched molecular weight (relative abundance: 99.99%) for CC=O

GHS hazard classification: Health Hazard, Irritant

NN-rank 4: ['C', 'CCCCCC(C)CCOC(C)=O']

NN-rank 5: ['C', 'CCCCCC(C)CCOC(C)=O']

NN-rank 6: ['C=C(C)CCCC(C)CCOC(C)=O']

NN-rank 7: ['C=C(C)CCCC(C)CCOC(C)=O']

NN-rank 8: ['CC1CCOC(=O)C=C(C(C)C)CC1']

NN-rank 9: ['CC(=O)O', 'CCC(C)CCC=C(C)C']

NN-rank 10: ['C', 'CC=CCCC(C)CCOC(C)=O']

NN-rank 11: ['C', 'CC=CCCC(C)CCOC(C)=O']

NN-rank 12: ['CC(=O)OCCC(C)CC=CC(C)C']

NN-rank 13: ['C', 'C', 'CCCCC(C)CCOC(C)=O']

NN-rank 14: ['C=CC(C)CCC=C(C)C', 'CC(=O)O']

NN-rank 15: ['CC(=O)OCCC(C)CC=C=C(C)C']

NN-rank 16: ['C=C=O', 'CC(C)=CCCC(C)CCO']

EI-MS matched molecular weight (relative abundance: 77.07%) for C=C=O

GHS hazard classification: Acute Toxic, Irritant

NN-rank 17: ['CC(C)=CCCC(C)C', 'COC(C)=O']

NN-rank 18: ['CC(=O)OC=CC(C)CCC=C(C)C']

NN-rank 19: ['CC(=O)O', 'CC1CCCC(C)(C)CC1']

NN-rank 20: ['C', 'C', 'C=CCCC(C)CCOC(C)=O']

NN/MS matches corresponding with tox GHS classification highlight in Bold font.

NN-rank 21: ['CC1CCCC(C)(C)C(=O)C1', 'CC=O']

EI-MS matched molecular weight (relative abundance: 99.99%) for CC=O

GHS hazard classification: Health Hazard, Irritant

NN-rank 22: ['CC(=O)OC1CC(C)CCCC1(C)C']

NN-rank 23: ['CC(C)=C1CCC(C)CCOC(=O)C1']

NN-rank 24: ['CC1CCOC(=O)CC(C(C)C)CC1']

NN-rank 25: ['C', 'CCC1=CC(=O)OCCC(C)CC1']

NN/MS matches corresponding with tox GHS classification highlight in Bold font.

78. D-Carvone: CC1=CC[C@@H](CC1=O)C(=C)C

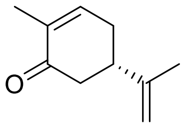

NN-rank 1: ['CC1=CC[C](C(C)C)CC1=O']

EI-MS matched molecular weight (relative abundance: 7.91%) for CC1=CC[C](C(C)C)CC1=O

GHS hazard classification: unknown

NN-rank 2: ['C=C(C)C1=CC(=O)C(C)=CC1']

NN-rank 3: ['CC1=CCC(=C(C)C)CC1=O']

NN-rank 4: ['C=C(C)[C]1CCC(C)C(=O)C1']

EI-MS matched molecular weight (relative abundance: 7.91%) for C=C(C)[C]1CCC(C)C(=O)C1

GHS hazard classification: unknown

NN-rank 5: ['C', 'C=C(C)[C]1CC=CC(=O)C1']

NN-rank 6: ['C', 'C=C(C)[C]1CCCC(=O)C1']

NN-rank 7: ['C=C(C)[C]1CC=C(C)C(O)C1']

EI-MS matched molecular weight (relative abundance: 7.91%) for C=C(C)[C]1CC=C(C)C(O)C1

GHS hazard classification: unknown

NN-rank 8: ['C=C(C)C1=CC=C(C)C(=O)C1']

NN-rank 9: ['C=C1CC2=C(C)C(=O)C[C]1C2']

NN-rank 10: ['C=C(C)[C]1CC=C(C)C(=O)C1']

NN-rank 11: ['C=C(C)[C](CC)CC(=O)CC']

NN-rank 12: ['C=C(C)[C]CC(=O)C(C)=CC']

NN-rank 13: ['C=C1CC2C[C]1CC(=O)C2C']

NN-rank 14: ['CC1=CC2=C(C)C(=O)C[C]1C2']

NN-rank 15: ['CC1=CC[C]CC1=O', 'C=CC']

EI-MS matched molecular weight (relative abundance: 19.08%) for CC1=CC[C]CC1=O

GHS hazard classification: unknown

EI-MS matched molecular weight (relative abundance: 17.5%) for C=CC

GHS hazard classification: none of the hazards selected for correlation

NN-rank 16: ['CC1=C2C[C](CC1=O)C(C)C2']

NN-rank 17: ['CC=C(C)C(=O)C[C]=C(C)C']

NN/MS matches corresponding with tox GHS classification highlight in Bold font.

EI-MS matched molecular weight (relative abundance: 7.91%) for CC=C(C)C(=O)C[C]=C(C)C

GHS hazard classification: unknown

NN-rank 18: ['C=C(C)[C]=CC(=O)C(C)=CC']

NN-rank 19: ['C=C(C)[C]1C=C(O)C(C)=CC1']

NN-rank 20: ['CC1=CC[C]CC1=O', 'CCC']

EI-MS matched molecular weight (relative abundance: 19.08%) for CC1=CC[C]CC1=O

GHS hazard classification: unknown

NN-rank 21: ['CC1=CC[C]=CC1=O', 'C=CC']

EI-MS matched molecular weight (relative abundance: 12.7%) for CC1=CC[C]=CC1=O

GHS hazard classification: unknown

EI-MS matched molecular weight (relative abundance: 17.5%) for C=CC

GHS hazard classification: none of the hazards selected for correlation

NN-rank 22: ['C=C(C)[C]CC=C(C)C(C)=O']

NN-rank 23: ['C=C(C)[C](CC=O)CC=CC']

EI-MS matched molecular weight (relative abundance: 7.91%) for C=C(C)[C](CC=O)CC=CC

GHS hazard classification: unknown

NN-rank 24: ['C', 'C=C1CC2C[C]1CC(=O)C2']

NN-rank 25: ['CC1CC2C[C]1CC(=O)C2C']

EI-MS matched molecular weight (relative abundance: 7.91%) for CC1CC2C[C]1CC(=O)C2C

GHS hazard classification: unknown

NN/MS matches corresponding with tox GHS classification highlight in Bold font.

79. Decanal: CCCCCCCCCC=O

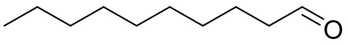

Summary Report of GHS Classification for NN/MS Matches:

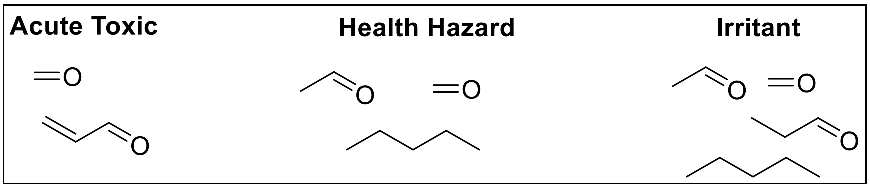

NN-rank 1: ['CCCCCCCC=CC=O']

NN-rank 2: ['CCCCCCCCCCO']

NN-rank 3: ['C', 'CCCCCCCCC=O']

NN-rank 4: ['CC', 'CCCCCCCC=O']

EI-MS matched molecular weight (relative abundance: 50.79%) for CC

GHS hazard classification: none of the hazards selected for correlation

NN-rank 5: ['C=O', 'C=CCCCCCCC']

EI-MS matched molecular weight (relative abundance: 50.79%) for C=O

GHS hazard classification: Acute Toxic, Health Hazard, Irritant

NN-rank 6: ['C=O', 'CCCCCCCCC']

EI-MS matched molecular weight (relative abundance: 50.79%) for C=O

GHS hazard classification: Acute Toxic, Health Hazard, Irritant

NN-rank 7: ['CCC', 'CCCCCCC=O']

EI-MS matched molecular weight (relative abundance: 99.99%) for CCC

GHS hazard classification: none of the hazards selected for correlation

NN-rank 8: ['CC=O', 'CCCCCCCC']

EI-MS matched molecular weight (relative abundance: 99.99%) for CC=O

GHS hazard classification: Health Hazard, Irritant

NN-rank 9: ['CCCCCCC=CCC=O']

NN-rank 10: ['CCCC', 'CCCCCC=O']

EI-MS matched molecular weight (relative abundance: 62.19%) for CCCC

GHS hazard classification: none of the hazards selected for correlation

NN-rank 11: ['CCCCCCCCC=C=O']

NN-rank 12: ['O=CCC1CCCCCCC1']

NN-rank 13: ['CCC=O', 'CCCCCCC']

EI-MS matched molecular weight (relative abundance: 62.19%) for CCC=O

NN/MS matches corresponding with tox GHS classification highlight in Bold font.

GHS hazard classification: Irritant

NN-rank 14: ['CCCC=O', 'CCCCCC']

EI-MS matched molecular weight (relative abundance: 33.79%) for CCCC=O

GHS hazard classification: none of the hazards selected for correlation

NN-rank 15: ['CCCCC', 'CCCCC=O']

EI-MS matched molecular weight (relative abundance: 33.79%) for CCCCC

GHS hazard classification: Health Hazard, Irritant

NN-rank 16: ['CCCCCCCCCC', 'O']

NN-rank 17: ['C=CCCCCCCCC', 'O']

NN-rank 18: ['CCCCCCC(C)CC=O']

NN-rank 19: ['CCCCCCCCC=CO']

NN-rank 20: ['C1CCCCCOCCCC1']

NN-rank 21: ['O=CC=C1CCCCCCC1']

NN-rank 22: ['C=CC=O', 'CCCCCCC']

EI-MS matched molecular weight (relative abundance: 61.89%) for C=CC=O

GHS hazard classification: Acute Toxic

NN-rank 23: ['CCCCCCCC=C=C=O']

NN-rank 24: ['C=CCCCCCCC', 'CO']

NN-rank 25: ['CCCCCCCCCOC']

NN/MS matches corresponding with tox GHS classification highlight in Bold font.

80. Decanoic Acid: CCCCCCCCCC(=O)O

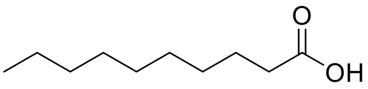

Summary Report of GHS Classification for NN/MS Matches:

Acute Toxic

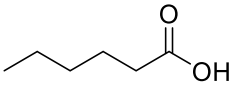

NN-rank 1: ['CCCCCCCCCC(=O)O']

NN-rank 2: ['C', 'CCCCCCCCC(=O)O']

NN-rank 3: ['O=C1CCCCCCCCCO1']

NN-rank 4: ['CCCCCCCCCCO', 'O']

NN-rank 5: ['CCCCCCCCCCO', 'O']

NN-rank 6: ['CCCCCCCCC(=O)OC']

NN-rank 7: ['CC', 'CCCCCCCC(=O)O']

EI-MS matched molecular weight (relative abundance: 25.29%) for CC

GHS hazard classification: none of the hazards selected for correlation

EI-MS matched molecular weight (relative abundance: 5.49%) for CCCCCCCC(=O)O

GHS hazard classification: none of the hazards selected for correlation

NN-rank 8: ['CC(=O)O', 'CCCCCCCC']

NN-rank 9: ['CCCCCCCCC', 'O=CO']

EI-MS matched molecular weight (relative abundance: 6.79%) for O=CO

GHS hazard classification: none of the hazards selected for correlation

NN-rank 10: ['CCC', 'CCCCCCC(=O)O']

EI-MS matched molecular weight (relative abundance: 43.59%) for CCC

GHS hazard classification: none of the hazards selected for correlation

EI-MS matched molecular weight (relative abundance: 33.49%) for CCCCCCC(=O)O

GHS hazard classification: none of the hazards selected for correlation

NN-rank 11: ['CCCCCCCCCC=O', 'O']

NN-rank 12: ['CCCCCCCCCC=O', 'O']

NN-rank 13: ['CCCCCCCC=CC(=O)O']

NN-rank 14: ['CCCC', 'CCCCCC(=O)O']

EI-MS matched molecular weight (relative abundance: 42.09%) for CCCC

GHS hazard classification: none of the hazards selected for correlation

NN/MS matches corresponding with tox GHS classification highlight in Bold font.

EI-MS matched molecular weight (relative abundance: 9.69%) for CCCCCC(=O)O

GHS hazard classification: Acute Toxic

NN-rank 15: ['CCCCCCCCC', 'O=CO']

EI-MS matched molecular weight (relative abundance: 6.79%) for O=CO

GHS hazard classification: none of the hazards selected for correlation

NN-rank 16: ['CCCCCCC=CCC(=O)O']

NN-rank 17: ['CCC(=O)O', 'CCCCCCC']

EI-MS matched molecular weight (relative abundance: 89.19%) for CCC(=O)O

GHS hazard classification: none of the hazards selected for correlation

NN-rank 18: ['CCCC(=O)O', 'CCCCCC']

EI-MS matched molecular weight (relative abundance: 13.59%) for CCCC(=O)O

GHS hazard classification: none of the hazards selected for correlation

NN-rank 19: ['OCCCCCCCCCCO']

NN-rank 20: ['CCCCCCCCC=CO', 'O']

NN-rank 21: ['CCCCCCCCCC', 'O', 'O']

NN-rank 22: ['CCCCCCCCC=C=O', 'O']

NN-rank 23: ['C=CCCCCCCCC', 'O', 'O']

NN-rank 24: ['O=CCCCCCCCCO']

NN-rank 25: ['C1CCCCCOCCCC1', 'O']

NN/MS matches corresponding with tox GHS classification highlight in Bold font.

81. delta-Decalactone: CCCCCC1CCCC(=O)O1

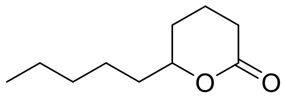

NN-rank 1: ['CCCCCC(O)CCCCO']

NN-rank 2: ['CCCCCCCCCC(=O)O']

NN-rank 3: ['C', 'CCCCC1CCCC(=O)O1']

NN-rank 4: ['CCCCCC(=O)CCCCO']

NN-rank 5: ['CCCCCCCCCC(=O)O']

NN-rank 6: ['CCCCCC1CCCC(=O)O1']

NN-rank 7: ['CCCCCC(O)CCCC=O']

NN-rank 8: ['CCCCCC=CCCC(=O)O']

NN-rank 9: ['CCCCCC1CCCC(O)O1']

NN-rank 10: ['O=C1CCCCCCCCCO1']

NN-rank 11: ['CCCCCC=CCCC(=O)O']

NN-rank 12: ['CCCCCC1=CCCC(=O)O1']

NN-rank 13: ['CC', 'CCCC1CCCC(=O)O1']

EI-MS matched molecular weight (relative abundance: 11.11%) for CC

GHS hazard classification: none of the hazards selected for correlation

NN-rank 14: ['CCCCCCCCC(=O)OC']

NN-rank 15: ['OC1CCCCCOCCCC1']

NN-rank 16: ['CCCCCC(=O)CCCC=O']

NN-rank 17: ['CC1CCCCCCCC(=O)O1']

NN-rank 18: ['CCCCC(O)CCCCOC']

NN-rank 19: ['CCCCC1CCCCC(=O)O1']

NN-rank 20: ['CC1CCCC(O)CCCCO1']

NN-rank 21: ['CC1CCCC(=O)O1', 'CCCC']

EI-MS matched molecular weight (relative abundance: 5.5%) for CCCC

GHS hazard classification: none of the hazards selected for correlation

NN-rank 22: ['CCCCCCCCCCO', 'O']

EI-MS matched molecular weight (relative abundance: 7.91%) for O

GHS hazard classification: none of the hazards selected for correlation

NN-rank 23: ['CCC', 'CCC1CCCC(=O)O1']

EI-MS matched molecular weight (relative abundance: 26.62%) for CCC

NN/MS matches corresponding with tox GHS classification highlight in Bold font.

GHS hazard classification: none of the hazards selected for correlation

NN-rank 24: ['CCCCC1OCCCCC1O']

NN-rank 25: ['CCCCC1CCCC(OC)O1']

NN/MS matches corresponding with tox GHS classification highlight in Bold font.

82. delta-Dodecalactone: CCCCCCCC1CCCC(=O)O1

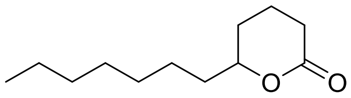

NN-rank 1: ['CCCCCCCC(O)CCCCO']

NN-rank 2: ['CCCCCCCCCCCC(=O)O']

NN-rank 3: ['C', 'CCCCCCC1CCCC(=O)O1']

NN-rank 4: ['CCCCCCCC(=O)CCCCO']

NN-rank 5: ['CCCCCCCC1CCCC(=O)O1']

NN-rank 6: ['CCCCCCCCCCCC(=O)O']

NN-rank 7: ['CCCCCCCC(O)CCCC=O']

NN-rank 8: ['CCCCCCCC=CCCC(=O)O']

NN-rank 9: ['CCCCCCCCCCC(=O)OC']

NN-rank 10: ['CCCCCCCC1CCCC(O)O1']

NN-rank 11: ['O=C1CCCCCCCCCCO1']

NN-rank 12: ['CCCCCCCC=CCCC(=O)O']

NN-rank 13: ['CCCCCCCC1=CCCC(=O)O1']

NN-rank 14: ['OC1CCCCCCCOCCCC1']

NN-rank 15: ['CCCCCCCC(=O)CCCC=O']

NN-rank 16: ['CC', 'CCCCCC1CCCC(=O)O1']

EI-MS matched molecular weight (relative abundance: 12.89%) for CC

GHS hazard classification: none of the hazards selected for correlation

NN-rank 17: ['CCCCCCC(O)CCCCOC']

NN-rank 18: ['CC1CCCCCCCCCC(=O)O1']

NN-rank 19: ['CCCCCCC1CCCCC(=O)O1']

NN-rank 20: ['CC1CCCCCC(O)CCCCO1']

NN-rank 21: ['CCCCCCCCCCCCO', 'O']

NN-rank 22: ['CCCCCCC1OCCCCC1O']

NN-rank 23: ['CCCCCCC1CCCC(OC)O1']

NN-rank 24: ['C1CCCOC2CCCC(CCC1)O2']

NN-rank 25: ['C', 'O=C1CCCCCCCCCO1']

NN/MS matches corresponding with tox GHS classification highlight in Bold font.

83. Dihydrocoumarin: C1CC(=O)OC2=CC=CC=C21

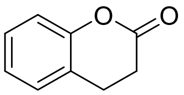

Summary Report of GHS Classification for NN/MS Matches:

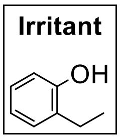

NN-rank 1: ['OCCCc1ccccc1O']

NN-rank 2: ['O=CCCc1ccccc1O']

EI-MS matched molecular weight (relative abundance: 10.21%) for O=CCCc1ccccc1O

GHS hazard classification: none of the hazards selected for correlation

NN-rank 3: ['c1ccc2c(c1)CCCO2', 'O']

NN-rank 4: ['O=C1CCc2cccc1c2O']

NN-rank 5: ['OC1CCc2ccccc2O1']

EI-MS matched molecular weight (relative abundance: 10.21%) for OC1CCc2ccccc2O1

GHS hazard classification: none of the hazards selected for correlation

NN-rank 6: ['O=C(O)CCc1ccccc1']

EI-MS matched molecular weight (relative abundance: 10.21%) for O=C(O)CCc1ccccc1

GHS hazard classification: none of the hazards selected for correlation

NN-rank 7: ['O=C1CCc2c(O)cccc21']

NN-rank 8: ['O=C(O)CCc1ccccc1']

EI-MS matched molecular weight (relative abundance: 10.21%) for O=C(O)CCc1ccccc1

GHS hazard classification: none of the hazards selected for correlation

NN-rank 9: ['O=c1ccc2ccccc2o1']

NN-rank 10: ['Oc1c2cccc1C(O)CC2']

EI-MS matched molecular weight (relative abundance: 10.21%) for Oc1c2cccc1C(O)CC2

GHS hazard classification: unknown

NN-rank 11: ['OCCCc1cccc2c1O2']

NN/MS matches corresponding with tox GHS classification highlight in Bold font.

EI-MS matched molecular weight (relative abundance: 10.21%) for OCCCc1cccc2c1O2

GHS hazard classification: unknown

NN-rank 12: ['O', 'OCCCc1ccccc1']

NN-rank 13: ['Oc1ccccc1CC1CO1']

EI-MS matched molecular weight (relative abundance: 10.21%) for Oc1ccccc1CC1CO1

GHS hazard classification: none of the hazards selected for correlation

NN-rank 14: ['C=O', 'CCc1ccccc1O']

EI-MS matched molecular weight (relative abundance: 7.01%) for CCc1ccccc1O

GHS hazard classification: Irritant

NN-rank 15: ['Oc1cccc2c1CCC2O']

EI-MS matched molecular weight (relative abundance: 10.21%) for Oc1cccc2c1CCC2O

GHS hazard classification: none of the hazards selected for correlation

NN-rank 16: ['OC=CCc1ccccc1O']

EI-MS matched molecular weight (relative abundance: 10.21%) for OC=CCc1ccccc1O

GHS hazard classification: none of the hazards selected for correlation

NN-rank 17: ['OC12CCc3cccc1c3O2']

NN-rank 18: ['O=C1CC2c3cccc(c32)O1']

NN-rank 19: ['O=C1CC2C3=CC=CC2=C3O1']

NN-rank 20: ['O=C=CCc1ccccc1O']

NN-rank 21: ['O=CCCc1cccc2c1O2']

NN-rank 22: ['OC1=CCc2ccccc2O1']

NN-rank 23: ['O', 'Oc1c2cccc1CCC2']

NN-rank 24: ['OC12CCc3c(cccc31)O2']

NN-rank 25: ['c1ccc2c(c1)CC1OC1O2']

NN/MS matches corresponding with tox GHS classification highlight in Bold font.

84. Dimethyl Anthranilate: CNC1=CC=CC=C1C(=O)OC

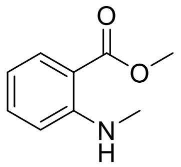

Summary Report of GHS Classification for NN/MS Matches:

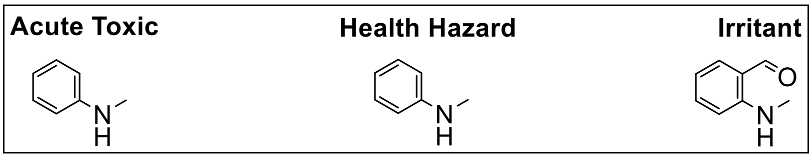

NN-rank 1: ['C', 'CNc1ccccc1C(=O)O']

NN-rank 2: ['C', 'COC(=O)c1ccccc1N']

NN-rank 3: ['C', 'CNc1ccccc1C=O', 'O']

EI-MS matched molecular weight (relative abundance: 23.52%) for CNc1ccccc1C=O

GHS hazard classification: Irritant

NN-rank 4: ['C', 'CNc1ccccc1C(=O)O']

NN-rank 5: ['CNc1ccccc1C=O', 'CO']

EI-MS matched molecular weight (relative abundance: 23.52%) for CNc1ccccc1C=O

GHS hazard classification: Irritant

NN-rank 6: ['CNc1ccccc1COC', 'O']

NN-rank 7: ['CNc1ccccc1CO', 'CO']

NN-rank 8: ['CNc1ccccc1C', 'CO', 'O']

NN-rank 9: ['C', 'CNc1c2cccc1C2=O', 'O']

EI-MS matched molecular weight (relative abundance: 55.55%) for CNc1c2cccc1C2=O

GHS hazard classification: unknown

NN-rank 10: ['CNc1ccccc1', 'COC=O']

EI-MS matched molecular weight (relative abundance: 15.91%) for CNc1ccccc1

GHS hazard classification: Acute Toxic, Health Hazard

NN-rank 11: ['C', 'CN1C(=O)c2ccccc21', 'O']

EI-MS matched molecular weight (relative abundance: 55.55%) for CN1C(=O)c2ccccc21

GHS hazard classification: none of the hazards selected for correlation

NN/MS matches corresponding with tox GHS classification highlight in Bold font.

NN-rank 12: ['CN1C(=O)c2ccccc21', 'CO']

EI-MS matched molecular weight (relative abundance: 55.55%) for CN1C(=O)c2ccccc21

GHS hazard classification: none of the hazards selected for correlation

NN-rank 13: ['C', 'CO', 'O=C1Nc2ccccc21']

NN-rank 14: ['C', 'C', 'O', 'O=C1Nc2ccccc21']

NN-rank 15: ['CNc1c2cccc1C2=O', 'CO']

EI-MS matched molecular weight (relative abundance: 55.55%) for CNc1c2cccc1C2=O

GHS hazard classification: unknown

NN-rank 16: ['C', 'C=O', 'CNc1ccccc1', 'O']

EI-MS matched molecular weight (relative abundance: 15.91%) for CNc1ccccc1

GHS hazard classification: Acute Toxic, Health Hazard

NN-rank 17: ['CNc1c(C)cccc1C(=O)O']

NN-rank 18: ['CN', 'COC(=O)c1ccccc1']

NN-rank 19: ['CNc1ccccc1', 'COC', 'O']

EI-MS matched molecular weight (relative abundance: 15.91%) for CNc1ccccc1

GHS hazard classification: Acute Toxic, Health Hazard

NN-rank 20: ['C', 'CNc1cccc2c(=O)c12', 'O']

EI-MS matched molecular weight (relative abundance: 55.55%) for CNc1cccc2c(=O)c12

GHS hazard classification: unknown

NN-rank 21: ['C', 'CNc1ccccc1', 'O=C=O']

EI-MS matched molecular weight (relative abundance: 15.91%) for CNc1ccccc1

GHS hazard classification: Acute Toxic, Health Hazard

NN-rank 22: ['C', 'COC(=O)c1ccccc1N']

NN-rank 23: ['CNc1cccc(C)c1C(=O)O']

NN-rank 24: ['CNc1c2cccc1C(=O)OC2']

NN-rank 25: ['C=O', 'CNc1ccccc1', 'CO']

EI-MS matched molecular weight (relative abundance: 15.91%) for CNc1ccccc1

GHS hazard classification: Acute Toxic, Health Hazard

NN/MS matches corresponding with tox GHS classification highlight in Bold font.

85. Cassia oil-A: C/C=C/C1=CC=CC=C1

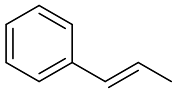

Summary Report of GHS Classification for NN/MS Matches:

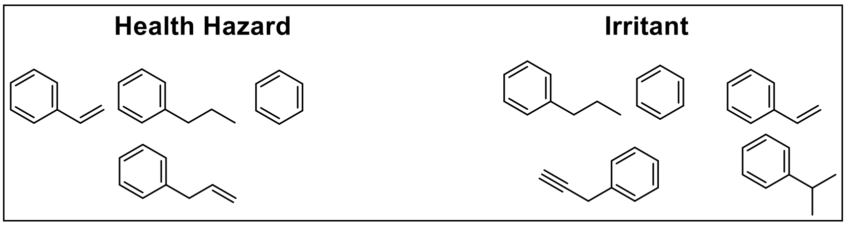

NN-rank 1: ['CCCc1ccccc1']

EI-MS matched molecular weight (relative abundance: 7.03%) for CCCc1ccccc1

GHS hazard classification: Health Hazard, Irritant

NN-rank 2: ['C=CCc1ccccc1']

EI-MS matched molecular weight (relative abundance: 99.99%) for C=CCc1ccccc1

GHS hazard classification: Health Hazard

NN-rank 3: ['C', 'CCc1ccccc1']

NN-rank 4: ['c1ccc(C2CC2)cc1']

EI-MS matched molecular weight (relative abundance: 99.99%) for c1ccc(C2CC2)cc1

GHS hazard classification: none of the hazards selected for correlation

NN-rank 5: ['C#CCc1ccccc1']

EI-MS matched molecular weight (relative abundance: 33.13%) for C#CCc1ccccc1

GHS hazard classification: Irritant

NN-rank 6: ['C1=CC1c1ccccc1']

EI-MS matched molecular weight (relative abundance: 33.13%) for C1=CC1c1ccccc1

GHS hazard classification: none of the hazards selected for correlation

NN-rank 7: ['C1#CC1c1ccccc1']

NN-rank 8: ['CCC', 'c1ccccc1']

EI-MS matched molecular weight (relative abundance: 10.01%) for c1ccccc1

GHS hazard classification: Health Hazard, Irritant

NN-rank 9: ['C=CC', 'c1ccccc1']

EI-MS matched molecular weight (relative abundance: 10.01%) for c1ccccc1

GHS hazard classification: Health Hazard, Irritant

NN-rank 10: ['C1=C(c2ccccc2)C1']

NN/MS matches corresponding with tox GHS classification highlight in Bold font.

EI-MS matched molecular weight (relative abundance: 33.13%) for C1=C(c2ccccc2)C1

GHS hazard classification: none of the hazards selected for correlation

NN-rank 11: ['CC1Cc2ccccc21']

EI-MS matched molecular weight (relative abundance: 99.99%) for CC1Cc2ccccc21

GHS hazard classification: none of the hazards selected for correlation

NN-rank 12: ['CC1Cc2ccccc21']

EI-MS matched molecular weight (relative abundance: 99.99%) for CC1Cc2ccccc21

GHS hazard classification: none of the hazards selected for correlation

NN-rank 13: ['CCC1c2ccccc21']

EI-MS matched molecular weight (relative abundance: 99.99%) for CCC1c2ccccc21

GHS hazard classification: none of the hazards selected for correlation

NN-rank 14: ['CCC1c2ccccc21']

EI-MS matched molecular weight (relative abundance: 99.99%) for CCC1c2ccccc21

GHS hazard classification: none of the hazards selected for correlation

NN-rank 15: ['C1=C(c2ccccc2)C1']

EI-MS matched molecular weight (relative abundance: 33.13%) for C1=C(c2ccccc2)C1

GHS hazard classification: none of the hazards selected for correlation

NN-rank 16: ['C', 'C=Cc1ccccc1']

EI-MS matched molecular weight (relative abundance: 10.01%) for C=Cc1ccccc1

GHS hazard classification: Health Hazard, Irritant

NN-rank 17: ['C=C=Cc1ccccc1']

EI-MS matched molecular weight (relative abundance: 33.13%) for C=C=Cc1ccccc1

GHS hazard classification: none of the hazards selected for correlation

NN-rank 18: ['CC(C)c1ccccc1']

EI-MS matched molecular weight (relative abundance: 7.03%) for CC(C)c1ccccc1

GHS hazard classification: Irritant

NN-rank 19: ['C=CC', 'c1ccccc1']

EI-MS matched molecular weight (relative abundance: 10.01%) for c1ccccc1

GHS hazard classification: Health Hazard, Irritant

NN-rank 20: ['CCC1C2=CC=C1C=C2']

EI-MS matched molecular weight (relative abundance: 99.99%) for CCC1C2=CC=C1C=C2

GHS hazard classification: none of the hazards selected for correlation

NN/MS matches corresponding with tox GHS classification highlight in Bold font.

NN-rank 21: ['C1CC1', 'c1ccccc1']

EI-MS matched molecular weight (relative abundance: 10.01%) for c1ccccc1

GHS hazard classification: Health Hazard, Irritant

NN-rank 22: ['c1ccc2c(c1)C1CC21']

EI-MS matched molecular weight (relative abundance: 33.13%) for c1ccc2c(c1)C1CC21

GHS hazard classification: none of the hazards selected for correlation

NN-rank 23: ['c1ccc2c(c1)C1CC21']

EI-MS matched molecular weight (relative abundance: 33.13%) for c1ccc2c(c1)C1CC21

GHS hazard classification: none of the hazards selected for correlation

NN-rank 24: ['CCC1c2cccc1c2']

EI-MS matched molecular weight (relative abundance: 99.99%) for CCC1c2cccc1c2

GHS hazard classification: none of the hazards selected for correlation

NN-rank 25: ['CCC1c2cccc1c2']

EI-MS matched molecular weight (relative abundance: 99.99%) for CCC1c2cccc1c2

GHS hazard classification: none of the hazards selected for correlation

NN/MS matches corresponding with tox GHS classification highlight in Bold font.

86. Dodecane: CCCCCCCCCCCC

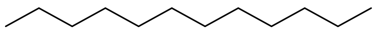

Summary Report of GHS Classification for NN/MS Matches:

Health Hazard	Irritant
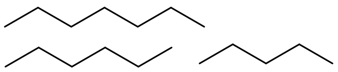	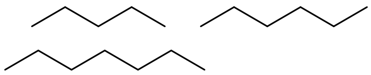

NN-rank 1: ['C', 'CCCCCCCCCCC']

NN-rank 2: ['C', 'CCCCCCCCCCC']

NN-rank 3: ['CCCCCCCCCCCC']

NN-rank 4: ['CCCCCCCCCCCC']

NN-rank 5: ['CCCCCCCCCC(C)C']

NN-rank 6: ['CCCCCCCCCC(C)C']

NN-rank 7: ['CC1CCCCCCCCC1']

NN-rank 8: ['CC1CCCCCCCCC1']

NN-rank 9: ['C1CCCCCCCCCC1']

NN-rank 10: ['CC', 'CCCCCCCCCC']

EI-MS matched molecular weight (relative abundance: 13.7%) for CC

GHS hazard classification: none of the hazards selected for correlation

NN-rank 11: ['CC', 'CCCCCCCCCC']

EI-MS matched molecular weight (relative abundance: 13.7%) for CC

GHS hazard classification: none of the hazards selected for correlation

NN-rank 12: ['C', 'C1CCCCCCCCC1']

NN-rank 13: ['C', 'C1CCCCCCCCC1']

NN-rank 14: ['CCC', 'CCCCCCCCC']

EI-MS matched molecular weight (relative abundance: 68.39%) for CCC

GHS hazard classification: none of the hazards selected for correlation

NN-rank 15: ['CCC', 'CCCCCCCCC']

EI-MS matched molecular weight (relative abundance: 68.39%) for CCC

GHS hazard classification: none of the hazards selected for correlation

NN-rank 16: ['CCCCCCCCCCCC']

NN-rank 17: ['CCCCCCCCCCCC']

NN-rank 18: ['CCCC', 'CCCCCCCC']

EI-MS matched molecular weight (relative abundance: 99.99%) for CCCC

NN/MS matches corresponding with tox GHS classification highlight in Bold font.

GHS hazard classification: none of the hazards selected for correlation

NN-rank 19: ['CCCC', 'CCCCCCCC']

EI-MS matched molecular weight (relative abundance: 99.99%) for CCCC

GHS hazard classification: none of the hazards selected for correlation

NN-rank 20: ['CC1CCCCCCCCC1C']

NN-rank 21: ['CCCCC', 'CCCCCCC']

EI-MS matched molecular weight (relative abundance: 53.69%) for CCCCC

GHS hazard classification: Health Hazard, Irritant

EI-MS matched molecular weight (relative abundance: 5.9%) for CCCCCCC

GHS hazard classification: Health Hazard, Irritant

NN-rank 22: ['CCCCC', 'CCCCCCC']

EI-MS matched molecular weight (relative abundance: 53.69%) for CCCCC

GHS hazard classification: Health Hazard, Irritant

EI-MS matched molecular weight (relative abundance: 5.9%) for CCCCCCC

GHS hazard classification: Health Hazard, Irritant

NN-rank 23: ['CCCCCC', 'CCCCCC']

EI-MS matched molecular weight (relative abundance: 32.9%) for CCCCCC

GHS hazard classification: Health Hazard, Irritant

EI-MS matched molecular weight (relative abundance: 32.9%) for CCCCCC

GHS hazard classification: Health Hazard, Irritant

NN-rank 24: ['C', 'CC1CCCCCCCCC1']

NN-rank 25: ['C', 'CC1CCCCCCCCC1']

NN/MS matches corresponding with tox GHS classification highlight in Bold font.

87. Triethyl Citrate: CCOC(=O)CC(CC(=O)OCC)(C(=O)OCC)O

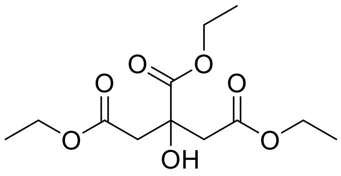

NN-rank 1: ['CC', 'CCOC(=O)CC(O)(CC(=O)OCC)C(=O)O']

EI-MS matched molecular weight (relative abundance: 44.99%) for CC

GHS hazard classification: none of the hazards selected for correlation

NN-rank 2: ['CC', 'CCOC(=O)CC(O)(CC(=O)O)C(=O)OCC']

EI-MS matched molecular weight (relative abundance: 44.99%) for CC

GHS hazard classification: none of the hazards selected for correlation

NN-rank 3: ['CC', 'CCOC(=O)CC(O)(CC(=O)O)C(=O)OCC']

EI-MS matched molecular weight (relative abundance: 44.99%) for CC

GHS hazard classification: none of the hazards selected for correlation

NN-rank 4: ['CCO', 'CCOC(=O)CC(O)(CO)CC(=O)OCC']

NN-rank 5: ['CCO', 'CCOC(=O)C1C(=O)CC1(O)C(=O)OCC']

NN-rank 6: ['CCO', 'CCOC(=O)C1C(=O)CC1(O)C(=O)OCC']

NN-rank 7: ['CCO', 'CCOC(=O)CC(O)(C=O)CC(=O)OCC']

NN-rank 8: ['CCO', 'CCOC(=O)CC(O)(CCO)C(=O)OCC']

NN-rank 9: ['CCO', 'CCOC(=O)CC(O)(CCO)C(=O)OCC']

NN-rank 10: ['CCO', 'CCOC(=O)CC1(O)C(=O)C1C(=O)OCC']

NN-rank 11: ['CCO', 'CCOC(=O)CC1(O)C(=O)C1C(=O)OCC']

NN-rank 12: ['CCO', 'CCOC(=O)CC(O)(CC=O)C(=O)OCC']

NN-rank 13: ['CCO', 'CCOC(=O)CC(O)(CC=O)C(=O)OCC']

NN-rank 14: ['CC', 'CCOC(=O)CC(O)(CC(=O)O)C(=O)OCC']

EI-MS matched molecular weight (relative abundance: 44.99%) for CC

GHS hazard classification: none of the hazards selected for correlation

NN-rank 15: ['CC', 'CCOC(=O)CC(O)(CC(=O)O)C(=O)OCC']

EI-MS matched molecular weight (relative abundance: 44.99%) for CC

GHS hazard classification: none of the hazards selected for correlation

NN-rank 16: ['CCOC(=O)CC(CC(=O)OCC)C(=O)OCC', 'O']

NN-rank 17: ['CCOC(=O)CC(O)(CC(=O)OCC)C(O)OCC']

NN-rank 18: ['CCOC(=O)CC(O)(CC(O)OCC)C(=O)OCC']

NN-rank 19: ['CCOC(=O)CC(O)(CC(O)OCC)C(=O)OCC']

NN/MS matches corresponding with tox GHS classification highlight in Bold font.

NN-rank 20: ['CCO', 'CCOC(=O)C1C(O)CC1(O)C(=O)OCC']

NN-rank 21: ['CCO', 'CCOC(=O)C1C(O)CC1(O)C(=O)OCC']

NN-rank 22: ['CCOC(=O)C1C(O)(OCC)CC1(O)C(=O)OCC']

NN-rank 23: ['CCOC(=O)C1C(O)(OCC)CC1(O)C(=O)OCC']

NN-rank 24: ['CCO', 'CCOC(=O)CC1(O)C(O)C1C(=O)OCC']

NN-rank 25: ['CCO', 'CCOC(=O)CC1(O)C(O)C1C(=O)OCC']

NN/MS matches corresponding with tox GHS classification highlight in Bold font.

88. Ethyl-3-Hydroxy Butyrate: CC(O)CC(OCC)=O

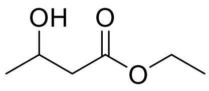

Summary Report of GHS Classification for NN/MS Matches:

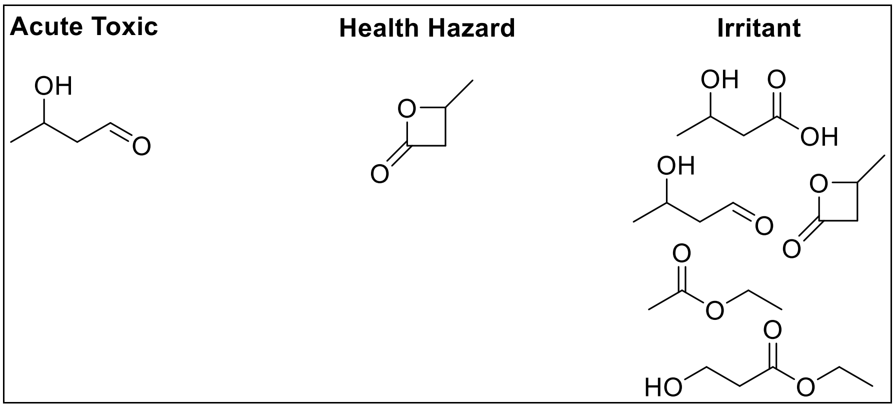

NN-rank 1: ['CC', 'CC(O)CC(=O)O']

EI-MS matched molecular weight (relative abundance: 29.6%) for CC

GHS hazard classification: none of the hazards selected for correlation

EI-MS matched molecular weight (relative abundance: 5.2%) for CC(O)CC(=O)O

GHS hazard classification: Irritant

NN-rank 2: ['CC', 'CC(O)CC(=O)O']

EI-MS matched molecular weight (relative abundance: 29.6%) for CC

GHS hazard classification: none of the hazards selected for correlation

EI-MS matched molecular weight (relative abundance: 5.2%) for CC(O)CC(=O)O

GHS hazard classification: Irritant

NN-rank 3: ['CC(O)CCO', 'CCO']

EI-MS matched molecular weight (relative abundance: 18.4%) for CC(O)CCO

GHS hazard classification: none of the hazards selected for correlation

EI-MS matched molecular weight (relative abundance: 96.19%) for CCO

GHS hazard classification: none of the hazards selected for correlation

NN-rank 4: ['CC=CC(=O)OCC', 'O']

NN-rank 5: ['CCOC(=O)C=C(C)O']

NN-rank 6: ['CCOC(=O)CC(C)=O']

NN-rank 7: ['CC', 'CC(O)CC=O', 'O']

EI-MS matched molecular weight (relative abundance: 29.6%) for CC

NN/MS matches corresponding with tox GHS classification highlight in Bold font.

GHS hazard classification: none of the hazards selected for correlation

EI-MS matched molecular weight (relative abundance: 49.8%) for CC(O)CC=O

GHS hazard classification: Acute Toxic, Irritant

NN-rank 8: ['CC(O)CC=O', 'CCO']

EI-MS matched molecular weight (relative abundance: 49.8%) for CC(O)CC=O

GHS hazard classification: Acute Toxic, Irritant

EI-MS matched molecular weight (relative abundance: 96.19%) for CCO

GHS hazard classification: none of the hazards selected for correlation

NN-rank 9: ['CCCC(=O)OCC', 'O']

NN-rank 10: ['C', 'CCOC(=O)CCO']

EI-MS matched molecular weight (relative abundance: 38.1%) for CCOC(=O)CCO

GHS hazard classification: Irritant

NN-rank 11: ['C', 'COC(=O)CC(C)O']

EI-MS matched molecular weight (relative abundance: 38.1%) for COC(=O)CC(C)O

GHS hazard classification: none of the hazards selected for correlation

NN-rank 12: ['CC(C)O', 'CCOC=O']

NN-rank 13: ['CCOCCC(C)O', 'O']

EI-MS matched molecular weight (relative abundance: 38.1%) for CCOCCC(C)O

GHS hazard classification: none of the hazards selected for correlation

NN-rank 14: ['CCO', 'O=C1CC(O)C1']

EI-MS matched molecular weight (relative abundance: 96.19%) for CCO

GHS hazard classification: none of the hazards selected for correlation

EI-MS matched molecular weight (relative abundance: 12.3%) for O=C1CC(O)C1

GHS hazard classification: none of the hazards selected for correlation

NN-rank 15: ['CC', 'O', 'O=C1CC(O)C1']

EI-MS matched molecular weight (relative abundance: 29.6%) for CC

GHS hazard classification: none of the hazards selected for correlation

EI-MS matched molecular weight (relative abundance: 12.3%) for O=C1CC(O)C1

GHS hazard classification: none of the hazards selected for correlation

NN-rank 16: ['CC1CC(=O)O1', 'CCO']

EI-MS matched molecular weight (relative abundance: 12.3%) for CC1CC(=O)O1

GHS hazard classification: Health Hazard, Irritant

EI-MS matched molecular weight (relative abundance: 96.19%) for CCO

GHS hazard classification: none of the hazards selected for correlation

NN-rank 17: ['CC', 'CC(C)O', 'O=C=O']

NN/MS matches corresponding with tox GHS classification highlight in Bold font.

EI-MS matched molecular weight (relative abundance: 29.6%) for CC

GHS hazard classification: none of the hazards selected for correlation

EI-MS matched molecular weight (relative abundance: 37.4%) for O=C=O

GHS hazard classification: none of the hazards selected for correlation

NN-rank 18: ['CCO', 'CCOC(C)=O']

EI-MS matched molecular weight (relative abundance: 96.19%) for CCO

GHS hazard classification: none of the hazards selected for correlation

EI-MS matched molecular weight (relative abundance: 49.8%) for CCOC(C)=O

GHS hazard classification: Irritant

NN-rank 19: ['CCOC(=O)C1OC1C']

NN-rank 20: ['C', 'CCOC(=O)C=CO']

NN-rank 21: ['CCOC(=O)C1=C(C)O1']

NN-rank 22: ['CC', 'CC(O)CCO', 'O']

EI-MS matched molecular weight (relative abundance: 29.6%) for CC

GHS hazard classification: none of the hazards selected for correlation

EI-MS matched molecular weight (relative abundance: 18.4%) for CC(O)CCO

GHS hazard classification: none of the hazards selected for correlation

NN-rank 23: ['CC', 'CC(C)O', 'O=CO']

EI-MS matched molecular weight (relative abundance: 29.6%) for CC

GHS hazard classification: none of the hazards selected for correlation

EI-MS matched molecular weight (relative abundance: 96.19%) for O=CO

GHS hazard classification: none of the hazards selected for correlation

NN-rank 24: ['CC(O)C=C=O', 'CCO']

EI-MS matched molecular weight (relative abundance: 12.3%) for CC(O)C=C=O

GHS hazard classification: none of the hazards selected for correlation

EI-MS matched molecular weight (relative abundance: 96.19%) for CCO

GHS hazard classification: none of the hazards selected for correlation

NN-rank 25: ['CC', 'CC1CC(=O)O1', 'O']

EI-MS matched molecular weight (relative abundance: 29.6%) for CC

GHS hazard classification: none of the hazards selected for correlation

EI-MS matched molecular weight (relative abundance: 12.3%) for CC1CC(=O)O1

GHS hazard classification: Health Hazard, Irritant

NN/MS matches corresponding with tox GHS classification highlight in Bold font.

89. Ethyl 2-Methyl Butyrate: CCC(C)C(=O)OCC

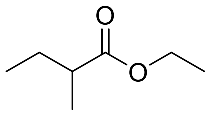

Summary Report of GHS Classification for NN/MS Matches:

Acute Toxic	Health Hazard	Irritant
=O	=O	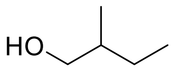 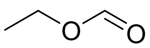 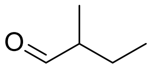 =O

NN-rank 1: ['CC', 'CCC(C)C(=O)O']

EI-MS matched molecular weight (relative abundance: 35.04%) for CC

GHS hazard classification: none of the hazards selected for correlation

NN-rank 2: ['CC', 'CCC(C)C(=O)O']

EI-MS matched molecular weight (relative abundance: 35.04%) for CC

GHS hazard classification: none of the hazards selected for correlation

NN-rank 3: ['CC', 'CCC(C)C=O', 'O']

EI-MS matched molecular weight (relative abundance: 35.04%) for CC

GHS hazard classification: none of the hazards selected for correlation

EI-MS matched molecular weight (relative abundance: 37.1%) for CCC(C)C=O

GHS hazard classification: Irritant

NN-rank 4: ['CC', 'CCC1(C)OC1=O']

EI-MS matched molecular weight (relative abundance: 35.04%) for CC

GHS hazard classification: none of the hazards selected for correlation

NN-rank 5: ['CCC(C)CO', 'CCO']

EI-MS matched molecular weight (relative abundance: 14.16%) for CCC(C)CO

GHS hazard classification: Irritant

EI-MS matched molecular weight (relative abundance: 8.4%) for CCO

GHS hazard classification: none of the hazards selected for correlation

NN-rank 6: ['CC', 'CCC(C)(O)C=O']

EI-MS matched molecular weight (relative abundance: 35.04%) for CC

GHS hazard classification: none of the hazards selected for correlation

NN-rank 7: ['C=C(CC)C(=O)OCC']

NN/MS matches corresponding with tox GHS classification highlight in Bold font.

NN-rank 8: ['C', 'CC', 'CCC1OC1=O']

EI-MS matched molecular weight (relative abundance: 35.04%) for CC

GHS hazard classification: none of the hazards selected for correlation

EI-MS matched molecular weight (relative abundance: 37.1%) for CCC1OC1=O

GHS hazard classification: none of the hazards selected for correlation

NN-rank 9: ['C', 'CCCC(=O)OCC']

EI-MS matched molecular weight (relative abundance: 8.7%) for CCCC(=O)OCC

GHS hazard classification: none of the hazards selected for correlation

NN-rank 10: ['CC', 'CCC(C)=C=O', 'O']

EI-MS matched molecular weight (relative abundance: 35.04%) for CC

GHS hazard classification: none of the hazards selected for correlation

NN-rank 11: ['CCC(C)C=O', 'CCO']

EI-MS matched molecular weight (relative abundance: 37.1%) for CCC(C)C=O

GHS hazard classification: Irritant

EI-MS matched molecular weight (relative abundance: 8.4%) for CCO

GHS hazard classification: none of the hazards selected for correlation

NN-rank 12: ['CC=C(C)C(=O)OCC']

NN-rank 13: ['CC', 'CCCC', 'O=CO']

EI-MS matched molecular weight (relative abundance: 35.04%) for CC

GHS hazard classification: none of the hazards selected for correlation

EI-MS matched molecular weight (relative abundance: 99.99%) for CCCC

GHS hazard classification: none of the hazards selected for correlation

EI-MS matched molecular weight (relative abundance: 8.4%) for O=CO

GHS hazard classification: none of the hazards selected for correlation

NN-rank 14: ['CC', 'CC', 'CC1OC1=O']

EI-MS matched molecular weight (relative abundance: 35.04%) for CC

GHS hazard classification: none of the hazards selected for correlation

EI-MS matched molecular weight (relative abundance: 35.04%) for CC

GHS hazard classification: none of the hazards selected for correlation

NN-rank 15: ['C=O', 'CC', 'CCCC', 'O']

EI-MS matched molecular weight (relative abundance: 35.04%) for C=O

GHS hazard classification: Acute Toxic, Health Hazard, Irritant

EI-MS matched molecular weight (relative abundance: 35.04%) for CC

GHS hazard classification: none of the hazards selected for correlation

EI-MS matched molecular weight (relative abundance: 99.99%) for CCCC

NN/MS matches corresponding with tox GHS classification highlight in Bold font.

GHS hazard classification: none of the hazards selected for correlation

NN-rank 16: ['C=CC(C)C(=O)OCC']

NN-rank 17: ['CCOCC(C)CC', 'O']

EI-MS matched molecular weight (relative abundance: 8.7%) for CCOCC(C)CC

GHS hazard classification: none of the hazards selected for correlation

NN-rank 18: ['CC', 'CCCC', 'O=C=O']

EI-MS matched molecular weight (relative abundance: 35.04%) for CC

GHS hazard classification: none of the hazards selected for correlation

EI-MS matched molecular weight (relative abundance: 99.99%) for CCCC

GHS hazard classification: none of the hazards selected for correlation

NN-rank 19: ['C', 'CC', 'CCC(O)C=O']

EI-MS matched molecular weight (relative abundance: 35.04%) for CC

GHS hazard classification: none of the hazards selected for correlation

EI-MS matched molecular weight (relative abundance: 14.16%) for CCC(O)C=O

GHS hazard classification: none of the hazards selected for correlation

NN-rank 20: ['CC1CCCCOC1=O']

NN-rank 21: ['C', 'CCOC(=O)C(C)C']

EI-MS matched molecular weight (relative abundance: 8.7%) for CCOC(=O)C(C)C

GHS hazard classification: none of the hazards selected for correlation

NN-rank 22: ['CCCC', 'CCOC=O']

EI-MS matched molecular weight (relative abundance: 99.99%) for CCCC

GHS hazard classification: none of the hazards selected for correlation

EI-MS matched molecular weight (relative abundance: 8.61%) for CCOC=O

GHS hazard classification: Irritant

NN-rank 23: ['CC1CCOC(=O)C1C']

NN-rank 24: ['CC', 'CCOC(=O)CC']

EI-MS matched molecular weight (relative abundance: 35.04%) for CC

GHS hazard classification: none of the hazards selected for correlation

NN-rank 25: ['C', 'CC=CC(=O)OCC']

NN/MS matches corresponding with tox GHS classification highlight in Bold font.

90. Ethyl 2-Phenyl Acetate: O=C(OCC)CC1=CC=CC=C1

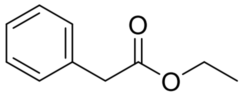

Summary Report of GHS Classification for NN/MS Matches:

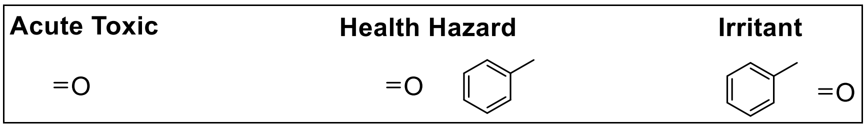

NN-rank 1: ['CC', 'O=C(O)Cc1ccccc1']

EI-MS matched molecular weight (relative abundance: 11.41%) for CC

GHS hazard classification: none of the hazards selected for correlation

NN-rank 2: ['CC', 'O=C(O)Cc1ccccc1']

EI-MS matched molecular weight (relative abundance: 11.41%) for CC

GHS hazard classification: none of the hazards selected for correlation

NN-rank 3: ['CCO', 'OCCc1ccccc1']

NN-rank 4: ['CC', 'O', 'O=CCc1ccccc1']

EI-MS matched molecular weight (relative abundance: 11.41%) for CC

GHS hazard classification: none of the hazards selected for correlation

NN-rank 5: ['CCc1ccccc1CC(=O)O']

NN-rank 6: ['CCc1ccccc1CC(=O)O']

NN-rank 7: ['CC', 'O', 'O=C1Cc2ccccc21']

EI-MS matched molecular weight (relative abundance: 11.41%) for CC

GHS hazard classification: none of the hazards selected for correlation

NN-rank 8: ['CC', 'O', 'O=C1Cc2ccccc21']

EI-MS matched molecular weight (relative abundance: 11.41%) for CC

GHS hazard classification: none of the hazards selected for correlation

NN-rank 9: ['CCO', 'O=CCc1ccccc1']

NN-rank 10: ['CCc1ccccc1CC(=O)O']

NN-rank 11: ['CCc1ccccc1CC(=O)O']

NN-rank 12: ['CCOC(C)=O', 'c1ccccc1']

NN-rank 13: ['CC', 'Cc1ccccc1', 'O=CO']

EI-MS matched molecular weight (relative abundance: 11.41%) for CC

GHS hazard classification: none of the hazards selected for correlation

NN/MS matches corresponding with tox GHS classification highlight in Bold font.

EI-MS matched molecular weight (relative abundance: 99.99%) for Cc1ccccc1

GHS hazard classification: Health Hazard, Irritant

NN-rank 14: ['CCOC=O', 'Cc1ccccc1']

EI-MS matched molecular weight (relative abundance: 99.99%) for Cc1ccccc1

GHS hazard classification: Health Hazard, Irritant

NN-rank 15: ['CCOCCc1ccccc1', 'O']

NN-rank 16: ['CCOC(=O)Cc1ccccc1']

NN-rank 17: ['CC', 'Cc1ccccc1', 'O=C=O']

EI-MS matched molecular weight (relative abundance: 11.41%) for CC

GHS hazard classification: none of the hazards selected for correlation

EI-MS matched molecular weight (relative abundance: 99.99%) for Cc1ccccc1

GHS hazard classification: Health Hazard, Irritant

NN-rank 18: ['O=C1OCC=C1c1ccccc1']

NN-rank 19: ['CCO', 'O=C1Cc2ccccc21']

NN-rank 20: ['CCO', 'O=C1Cc2ccccc21']

NN-rank 21: ['C=O', 'CC', 'Cc1ccccc1', 'O']

EI-MS matched molecular weight (relative abundance: 11.41%) for C=O

GHS hazard classification: Acute Toxic, Health Hazard, Irritant

EI-MS matched molecular weight (relative abundance: 11.41%) for CC

GHS hazard classification: none of the hazards selected for correlation

EI-MS matched molecular weight (relative abundance: 99.99%) for Cc1ccccc1

GHS hazard classification: Health Hazard, Irritant

NN-rank 22: ['C', 'COC(=O)Cc1ccccc1']

NN-rank 23: ['CC1OC(=O)Cc2ccccc21']

NN-rank 24: ['CC1OC(=O)Cc2ccccc21']

NN-rank 25: ['CC', 'O', 'OCCc1ccccc1']

EI-MS matched molecular weight (relative abundance: 11.41%) for CC

GHS hazard classification: none of the hazards selected for correlation

NN/MS matches corresponding with tox GHS classification highlight in Bold font.

91. Ethyl Acetate: CCOC(=O)C

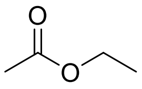

Summary Report of GHS Classification for NN/MS Matches:

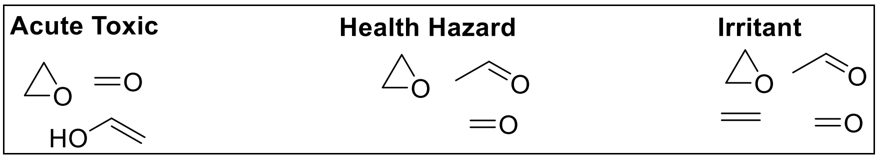

NN-rank 1: ['CC=O', 'CCO']

EI-MS matched molecular weight (relative abundance: 99.99%) for CC=O

GHS hazard classification: Health Hazard, Irritant

EI-MS matched molecular weight (relative abundance: 14.61%) for CCO

GHS hazard classification: none of the hazards selected for correlation

NN-rank 2: ['C=C=O', 'CCO']

EI-MS matched molecular weight (relative abundance: 14.61%) for CCO

GHS hazard classification: none of the hazards selected for correlation

NN-rank 3: ['CC', 'CC(=O)O']

EI-MS matched molecular weight (relative abundance: 12.41%) for CC

GHS hazard classification: none of the hazards selected for correlation

NN-rank 4: ['CCO', 'CCO']

EI-MS matched molecular weight (relative abundance: 14.61%) for CCO

GHS hazard classification: none of the hazards selected for correlation

EI-MS matched molecular weight (relative abundance: 14.61%) for CCO

GHS hazard classification: none of the hazards selected for correlation

NN-rank 5: ['C', 'CCOC=O']

EI-MS matched molecular weight (relative abundance: 5.2%) for C

GHS hazard classification: none of the hazards selected for correlation

NN-rank 6: ['C1CO1', 'CC=O']

EI-MS matched molecular weight (relative abundance: 99.99%) for C1CO1

GHS hazard classification: Acute Toxic, Health Hazard, Irritant

EI-MS matched molecular weight (relative abundance: 99.99%) for CC=O

GHS hazard classification: Health Hazard, Irritant

NN-rank 7: ['CCOCC', 'O']

NN/MS matches corresponding with tox GHS classification highlight in Bold font.

NN-rank 8: ['C=C', 'CC(=O)O']

EI-MS matched molecular weight (relative abundance: 5.4%) for C=C

GHS hazard classification: Irritant

NN-rank 9: ['CC=O', 'CCO']

EI-MS matched molecular weight (relative abundance: 99.99%) for CC=O

GHS hazard classification: Health Hazard, Irritant

EI-MS matched molecular weight (relative abundance: 14.61%) for CCO

GHS hazard classification: none of the hazards selected for correlation

NN-rank 10: ['C', 'COC(C)=O']

EI-MS matched molecular weight (relative abundance: 5.2%) for C

GHS hazard classification: none of the hazards selected for correlation

NN-rank 11: ['C', 'C=O', 'CCO']

EI-MS matched molecular weight (relative abundance: 5.2%) for C

GHS hazard classification: none of the hazards selected for correlation

EI-MS matched molecular weight (relative abundance: 12.41%) for C=O

GHS hazard classification: Acute Toxic, Health Hazard, Irritant

EI-MS matched molecular weight (relative abundance: 14.61%) for CCO

GHS hazard classification: none of the hazards selected for correlation

NN-rank 12: ['CC(=O)CCO']

NN-rank 13: ['C=COC(C)=O']

NN-rank 14: ['C=C', 'CCO', 'O']

EI-MS matched molecular weight (relative abundance: 5.4%) for C=C

GHS hazard classification: Irritant

EI-MS matched molecular weight (relative abundance: 14.61%) for CCO

GHS hazard classification: none of the hazards selected for correlation

NN-rank 15: ['CCOCC=O']

NN-rank 16: ['CC=O', 'CC=O']

EI-MS matched molecular weight (relative abundance: 99.99%) for CC=O

GHS hazard classification: Health Hazard, Irritant

EI-MS matched molecular weight (relative abundance: 99.99%) for CC=O

GHS hazard classification: Health Hazard, Irritant

NN-rank 17: ['CCOC(C)=O']

NN-rank 18: ['C=COCC', 'O']

NN-rank 19: ['C=CO', 'CCO']

EI-MS matched molecular weight (relative abundance: 99.99%) for C=CO

NN/MS matches corresponding with tox GHS classification highlight in Bold font.

GHS hazard classification: Acute Toxic

EI-MS matched molecular weight (relative abundance: 14.61%) for CCO

GHS hazard classification: none of the hazards selected for correlation

NN-rank 20: ['CC', 'O=CCO']

EI-MS matched molecular weight (relative abundance: 12.41%) for CC

GHS hazard classification: none of the hazards selected for correlation

NN-rank 21: ['CC', 'O=C1CO1']

EI-MS matched molecular weight (relative abundance: 12.41%) for CC

GHS hazard classification: none of the hazards selected for correlation

NN-rank 22: ['CCOC(C)O']

NN-rank 23: ['CC', 'CC(=O)O']

EI-MS matched molecular weight (relative abundance: 12.41%) for CC

GHS hazard classification: none of the hazards selected for correlation

NN-rank 24: ['O=C1C=CCO1']

NN-rank 25: ['C1CO1', 'C=C=O']

EI-MS matched molecular weight (relative abundance: 99.99%) for C1CO1

GHS hazard classification: Acute Toxic, Health Hazard, Irritant

NN/MS matches corresponding with tox GHS classification highlight in Bold font.

92. Ethyl Acetoacetate: CCOC(=O)CC(=O)C

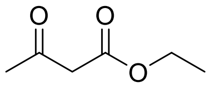

Summary Report of GHS Classification for NN/MS Matches:

**Health Hazard**	**Irritant**
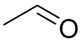	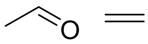

NN-rank 1: ['CC=CC(=O)OCC', 'O']

NN-rank 2: ['CCCC(=O)OCC', 'O']

NN-rank 3: ['CC', 'CC(=O)CC(=O)O']

EI-MS matched molecular weight (relative abundance: 23.59%) for CC

GHS hazard classification: none of the hazards selected for correlation

NN-rank 4: ['CC=O', 'CCOC(C)=O']

EI-MS matched molecular weight (relative abundance: 99.99%) for CC=O

GHS hazard classification: Health Hazard, Irritant

NN-rank 5: ['C', 'C=CC(=O)OCC', 'O']

EI-MS matched molecular weight (relative abundance: 8.19%) for C

GHS hazard classification: none of the hazards selected for correlation

NN-rank 6: ['CCOC(=O)C=CCO']

NN-rank 7: ['C=CC', 'CCOC=O', 'O']

NN-rank 8: ['CC(C)=O', 'CCOC=O']

NN-rank 9: ['CCOC(=O)CCCO']

NN-rank 10: ['CCOC(=O)C=C(C)O']

NN-rank 11: ['C', 'CCOC(=O)CC', 'O']

EI-MS matched molecular weight (relative abundance: 8.19%) for C

GHS hazard classification: none of the hazards selected for correlation

NN-rank 12: ['CCOC(=O)CC(C)O']

NN-rank 13: ['CCCC(=O)OCCO']

NN-rank 14: ['C', 'CCOC(=O)C=C=O']

EI-MS matched molecular weight (relative abundance: 8.19%) for C

GHS hazard classification: none of the hazards selected for correlation

NN-rank 15: ['C', 'COC(=O)CC(C)=O']

EI-MS matched molecular weight (relative abundance: 8.19%) for C

NN/MS matches corresponding with tox GHS classification highlight in Bold font.

GHS hazard classification: none of the hazards selected for correlation

NN-rank 16: ['CC=CC(=O)OCCO']

NN-rank 17: ['CC(=O)CC=O', 'CCO']

EI-MS matched molecular weight (relative abundance: 14.59%) for CC(=O)CC=O

GHS hazard classification: none of the hazards selected for correlation

EI-MS matched molecular weight (relative abundance: 5.39%) for CCO

GHS hazard classification: none of the hazards selected for correlation

NN-rank 18: ['CC', 'CCOC(C)=O', 'O']

EI-MS matched molecular weight (relative abundance: 23.59%) for CC

GHS hazard classification: none of the hazards selected for correlation

NN-rank 19: ['CCO', 'CCOC(C)=O']

EI-MS matched molecular weight (relative abundance: 5.39%) for CCO

GHS hazard classification: none of the hazards selected for correlation

NN-rank 20: ['CC(=O)CCO', 'CCO']

EI-MS matched molecular weight (relative abundance: 5.39%) for CCO

GHS hazard classification: none of the hazards selected for correlation

NN-rank 21: ['C=CCO', 'CCOC=O']

NN-rank 22: ['CCOC(=O)C=C1CO1']

NN-rank 23: ['C', 'C=C', 'CCOC=O', 'O']

EI-MS matched molecular weight (relative abundance: 8.19%) for C

GHS hazard classification: none of the hazards selected for correlation

EI-MS matched molecular weight (relative abundance: 10.69%) for C=C

GHS hazard classification: Irritant

NN-rank 24: ['C', 'CCOC(=O)CC=O']

EI-MS matched molecular weight (relative abundance: 8.19%) for C

GHS hazard classification: none of the hazards selected for correlation

NN-rank 25: ['CCO', 'O=C1CC(=O)C1']

EI-MS matched molecular weight (relative abundance: 5.39%) for CCO

GHS hazard classification: none of the hazards selected for correlation

NN/MS matches corresponding with tox GHS classification highlight in Bold font.

93. Ethyl Butyrate: CCCC(=O)OCC

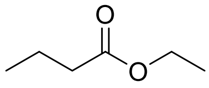

Summary Report of GHS Classification for NN/MS Matches:

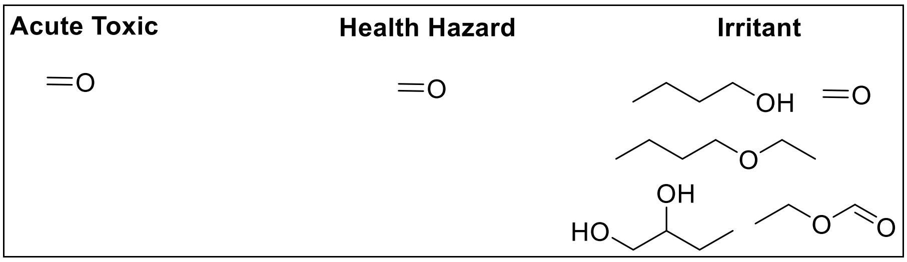

NN-rank 1: ['CC', 'CCCC(=O)O']

EI-MS matched molecular weight (relative abundance: 58.82%) for CC

GHS hazard classification: none of the hazards selected for correlation

NN-rank 2: ['CC', 'CCCC(=O)O']

EI-MS matched molecular weight (relative abundance: 58.82%) for CC

GHS hazard classification: none of the hazards selected for correlation

NN-rank 3: ['CC', 'CCCC=O', 'O']

EI-MS matched molecular weight (relative abundance: 58.82%) for CC

GHS hazard classification: none of the hazards selected for correlation

EI-MS matched molecular weight (relative abundance: 99.99%) for CCCC=O

GHS hazard classification: none of the hazards selected for correlation

NN-rank 4: ['CC', 'CCC1OC1=O']

EI-MS matched molecular weight (relative abundance: 58.82%) for CC

GHS hazard classification: none of the hazards selected for correlation

NN-rank 5: ['CCCCO', 'CCO']

EI-MS matched molecular weight (relative abundance: 21.08%) for CCCCO

GHS hazard classification: Irritant

EI-MS matched molecular weight (relative abundance: 18.5%) for CCO

GHS hazard classification: none of the hazards selected for correlation

NN-rank 6: ['CC', 'CC1CC1=O', 'O']

EI-MS matched molecular weight (relative abundance: 58.82%) for CC

GHS hazard classification: none of the hazards selected for correlation

NN-rank 7: ['CC', 'CCC', 'O=CO']

NN/MS matches corresponding with tox GHS classification highlight in Bold font.

EI-MS matched molecular weight (relative abundance: 58.82%) for CC

GHS hazard classification: none of the hazards selected for correlation

EI-MS matched molecular weight (relative abundance: 93.73%) for CCC

GHS hazard classification: none of the hazards selected for correlation

EI-MS matched molecular weight (relative abundance: 18.5%) for O=CO

GHS hazard classification: none of the hazards selected for correlation

NN-rank 8: ['C', 'CCOC(=O)CC']

EI-MS matched molecular weight (relative abundance: 7.16%) for C

GHS hazard classification: none of the hazards selected for correlation

EI-MS matched molecular weight (relative abundance: 9.05%) for CCOC(=O)CC

GHS hazard classification: none of the hazards selected for correlation

NN-rank 9: ['CC', 'CCC1OC1=O']

EI-MS matched molecular weight (relative abundance: 58.82%) for CC

GHS hazard classification: none of the hazards selected for correlation

NN-rank 10: ['CCCC=O', 'CCO']

EI-MS matched molecular weight (relative abundance: 99.99%) for CCCC=O

GHS hazard classification: none of the hazards selected for correlation

EI-MS matched molecular weight (relative abundance: 18.5%) for CCO

GHS hazard classification: none of the hazards selected for correlation

NN-rank 11: ['CC', 'CC', 'O=C1CO1']

EI-MS matched molecular weight (relative abundance: 58.82%) for CC

GHS hazard classification: none of the hazards selected for correlation

EI-MS matched molecular weight (relative abundance: 58.82%) for CC

GHS hazard classification: none of the hazards selected for correlation

NN-rank 12: ['CC', 'CCC', 'O=C=O']

EI-MS matched molecular weight (relative abundance: 58.82%) for CC

GHS hazard classification: none of the hazards selected for correlation

EI-MS matched molecular weight (relative abundance: 93.73%) for CCC

GHS hazard classification: none of the hazards selected for correlation

EI-MS matched molecular weight (relative abundance: 23.31%) for O=C=O

GHS hazard classification: none of the hazards selected for correlation

NN-rank 13: ['CC', 'CCC(O)C=O']

EI-MS matched molecular weight (relative abundance: 58.82%) for CC

GHS hazard classification: none of the hazards selected for correlation

NN-rank 14: ['C', 'CC', 'O', 'O=C1CC1']

NN/MS matches corresponding with tox GHS classification highlight in Bold font.

EI-MS matched molecular weight (relative abundance: 7.16%) for C

GHS hazard classification: none of the hazards selected for correlation

EI-MS matched molecular weight (relative abundance: 58.82%) for CC

GHS hazard classification: none of the hazards selected for correlation

NN-rank 15: ['CC1CCOC(=O)C1']

NN-rank 16: ['CCCCOCC', 'O']

EI-MS matched molecular weight (relative abundance: 9.05%) for CCCCOCC

GHS hazard classification: Irritant

NN-rank 17: ['CC', 'CCC(O)CO']

EI-MS matched molecular weight (relative abundance: 58.82%) for CC

GHS hazard classification: none of the hazards selected for correlation

EI-MS matched molecular weight (relative abundance: 15.93%) for CCC(O)CO

GHS hazard classification: Irritant

NN-rank 18: ['CC', 'CCCCO', 'O']

EI-MS matched molecular weight (relative abundance: 58.82%) for CC

GHS hazard classification: none of the hazards selected for correlation

EI-MS matched molecular weight (relative abundance: 21.08%) for CCCCO

GHS hazard classification: Irritant

NN-rank 19: ['C=O', 'CC', 'CCC', 'O']

EI-MS matched molecular weight (relative abundance: 58.82%) for C=O

GHS hazard classification: Acute Toxic, Health Hazard, Irritant

EI-MS matched molecular weight (relative abundance: 58.82%) for CC

GHS hazard classification: none of the hazards selected for correlation

EI-MS matched molecular weight (relative abundance: 93.73%) for CCC

GHS hazard classification: none of the hazards selected for correlation

NN-rank 20: ['CC', 'CCOC(C)=O']

EI-MS matched molecular weight (relative abundance: 58.82%) for CC

GHS hazard classification: none of the hazards selected for correlation

NN-rank 21: ['O=C1CCCCCO1']

NN-rank 22: ['CC', 'CCC(O)C=O']

EI-MS matched molecular weight (relative abundance: 58.82%) for CC

GHS hazard classification: none of the hazards selected for correlation

NN-rank 23: ['C', 'C', 'CCCC(=O)O']

EI-MS matched molecular weight (relative abundance: 7.16%) for C

GHS hazard classification: none of the hazards selected for correlation

NN/MS matches corresponding with tox GHS classification highlight in Bold font.

EI-MS matched molecular weight (relative abundance: 7.16%) for C

GHS hazard classification: none of the hazards selected for correlation

NN-rank 24: ['C', 'CCCC(=O)OC']

EI-MS matched molecular weight (relative abundance: 7.16%) for C

GHS hazard classification: none of the hazards selected for correlation

EI-MS matched molecular weight (relative abundance: 9.05%) for CCCC(=O)OC

GHS hazard classification: none of the hazards selected for correlation

NN-rank 25: ['CCC', 'CCOC=O']

EI-MS matched molecular weight (relative abundance: 93.73%) for CCC

GHS hazard classification: none of the hazards selected for correlation

EI-MS matched molecular weight (relative abundance: 21.08%) for CCOC=O

GHS hazard classification: Irritant

NN/MS matches corresponding with tox GHS classification highlight in Bold font.

94. Ethyl Cinnamate: CCOC(=O)/C=C/C1=CC=CC=C1

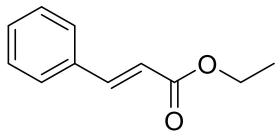

Summary Report of GHS Classification for NN/MS Matches:

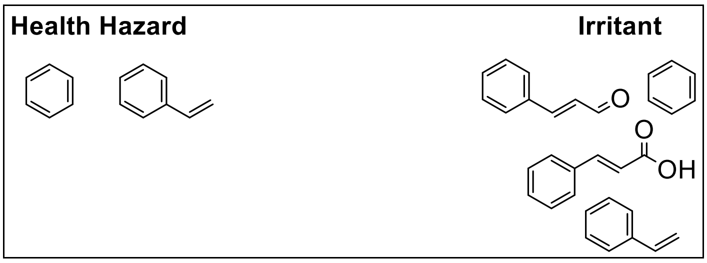

NN-rank 1: ['CC', 'O=C(O)/C=C/c1ccccc1']

EI-MS matched molecular weight (relative abundance: 5.79%) for CC

GHS hazard classification: none of the hazards selected for correlation

EI-MS matched molecular weight (relative abundance: 11.49%) for O=C(O)/C=C/c1ccccc1

GHS hazard classification: Irritant

NN-rank 2: ['CCO', 'OC/C=C/c1ccccc1']

NN-rank 3: ['CC', 'O=C(O)/C=C/c1ccccc1']

EI-MS matched molecular weight (relative abundance: 5.79%) for CC

GHS hazard classification: none of the hazards selected for correlation

EI-MS matched molecular weight (relative abundance: 11.49%) for O=C(O)/C=C/c1ccccc1

GHS hazard classification: Irritant

NN-rank 4: ['CCOC(=O)CCc1ccccc1']

NN-rank 5: ['CCO', 'O=C/C=C/c1ccccc1']

EI-MS matched molecular weight (relative abundance: 99.99%) for O=C/C=C/c1ccccc1

GHS hazard classification: Irritant

NN-rank 6: ['CC', 'O', 'O=C/C=C/c1ccccc1']

EI-MS matched molecular weight (relative abundance: 5.79%) for CC

GHS hazard classification: none of the hazards selected for correlation

EI-MS matched molecular weight (relative abundance: 99.99%) for

NN/MS matches corresponding with tox GHS classification highlight in Bold font.

O=C/C=C/c1ccccc1

GHS hazard classification: Irritant

NN-rank 7: ['CC', 'O=C1C=C(c2ccccc2)O1']

EI-MS matched molecular weight (relative abundance: 5.79%) for CC

GHS hazard classification: none of the hazards selected for correlation

NN-rank 8: ['CC', 'O=C/C=C(\\O)c1ccccc1']

EI-MS matched molecular weight (relative abundance: 5.79%) for CC

GHS hazard classification: none of the hazards selected for correlation

EI-MS matched molecular weight (relative abundance: 11.49%) for O=C/C=C(\O)c1ccccc1

GHS hazard classification: unknown

NN-rank 9: ['CCOC/C=C/c1ccccc1', 'O']

NN-rank 10: ['CC', 'O=C1CC(c2ccccc2)O1']

EI-MS matched molecular weight (relative abundance: 5.79%) for CC

GHS hazard classification: none of the hazards selected for correlation

EI-MS matched molecular weight (relative abundance: 11.49%) for O=C1CC(c2ccccc2)O1

GHS hazard classification: none of the hazards selected for correlation

NN-rank 11: ['CCO', 'O=c1cc1-c1ccccc1']

NN-rank 12: ['CCO/C(=C\\C=O)c1ccccc1']

NN-rank 13: ['C', 'COC(=O)/C=C/c1ccccc1']

NN-rank 14: ['O=C1OCC/C1=C\\c1ccccc1']

NN-rank 15: ['C=Cc1ccccc1', 'CCOC=O']

EI-MS matched molecular weight (relative abundance: 44.39%) for C=Cc1ccccc1

GHS hazard classification: Health Hazard, Irritant

NN-rank 16: ['O=C1C=C(c2ccccc2)CCO1']

NN-rank 17: ['CC', 'OC/C=C(\\O)c1ccccc1']

EI-MS matched molecular weight (relative abundance: 5.79%) for CC

GHS hazard classification: none of the hazards selected for correlation

NN-rank 18: ['C/C=C/c1ccccc1', 'CCO', 'O']

NN-rank 19: ['C=CC(=O)OCC', 'c1ccccc1']

EI-MS matched molecular weight (relative abundance: 29.99%) for c1ccccc1

GHS hazard classification: Health Hazard, Irritant

NN-rank 20: ['CC', 'O', 'O=c1cc1-c1ccccc1']

EI-MS matched molecular weight (relative abundance: 5.79%) for CC

NN/MS matches corresponding with tox GHS classification highlight in Bold font.

GHS hazard classification: none of the hazards selected for correlation

NN-rank 21: ['CCOC=O', 'CCc1ccccc1']

NN-rank 22: ['O=C1OCCC1Cc1ccccc1']

NN-rank 23: ['CC', 'O=CCC(O)c1ccccc1']

EI-MS matched molecular weight (relative abundance: 5.79%) for CC

GHS hazard classification: none of the hazards selected for correlation

NN-rank 24: ['CCO/C(=C\\CO)c1ccccc1']

NN-rank 25: ['CCOC(C)=O', 'Cc1ccccc1']

NN/MS matches corresponding with tox GHS classification highlight in Bold font.

95. Ethyl Decanoate: CCCCCCCCCC(=O)OCC

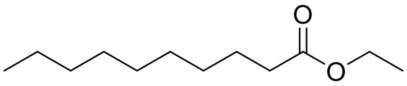

Summary Report of GHS Classification for NN/MS Matches:

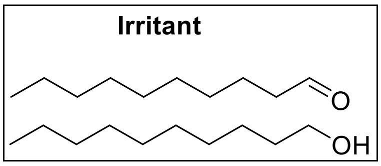

NN-rank 1: ['CC', 'CCCCCCCCCC(=O)O']

EI-MS matched molecular weight (relative abundance: 22.59%) for CC

GHS hazard classification: none of the hazards selected for correlation

NN-rank 2: ['CC', 'CCCCCCCCCC(=O)O']

EI-MS matched molecular weight (relative abundance: 22.59%) for CC

GHS hazard classification: none of the hazards selected for correlation

NN-rank 3: ['CC', 'CCCCCCCCCC=O', 'O']

EI-MS matched molecular weight (relative abundance: 22.59%) for CC

GHS hazard classification: none of the hazards selected for correlation

EI-MS matched molecular weight (relative abundance: 14.39%) for CCCCCCCCCC=O

GHS hazard classification: Irritant

NN-rank 4: ['CC', 'O=C1CCCCCCCCCO1']

EI-MS matched molecular weight (relative abundance: 22.59%) for CC

GHS hazard classification: none of the hazards selected for correlation

NN-rank 5: ['CC', 'CCCCCCCCC(=O)OC']

EI-MS matched molecular weight (relative abundance: 22.59%) for CC

GHS hazard classification: none of the hazards selected for correlation

NN-rank 6: ['CCCCCCCCCCO', 'CCO']

EI-MS matched molecular weight (relative abundance: 9.89%) for CCCCCCCCCCO

GHS hazard classification: Irritant

EI-MS matched molecular weight (relative abundance: 8.29%) for CCO

GHS hazard classification: none of the hazards selected for correlation

NN-rank 7: ['CC', 'O=C1CCCCCCCCCO1']

EI-MS matched molecular weight (relative abundance: 22.59%) for CC

GHS hazard classification: none of the hazards selected for correlation

NN/MS matches corresponding with tox GHS classification highlight in Bold font.

NN-rank 8: ['CC', 'CCCCCCCCC1OC1=O']

EI-MS matched molecular weight (relative abundance: 22.59%) for CC

GHS hazard classification: none of the hazards selected for correlation

NN-rank 9: ['CC', 'OCCCCCCCCCCO']

EI-MS matched molecular weight (relative abundance: 22.59%) for CC

GHS hazard classification: none of the hazards selected for correlation

NN-rank 10: ['CC', 'CCCCCCCCC(=O)OC']

EI-MS matched molecular weight (relative abundance: 22.59%) for CC

GHS hazard classification: none of the hazards selected for correlation

NN-rank 11: ['CC', 'O=CCCCCCCCCCO']

EI-MS matched molecular weight (relative abundance: 22.59%) for CC

GHS hazard classification: none of the hazards selected for correlation

NN-rank 12: ['CC', 'CCCCCCCCCO', 'CO']

EI-MS matched molecular weight (relative abundance: 22.59%) for CC

GHS hazard classification: none of the hazards selected for correlation

NN-rank 13: ['C', 'CCCCCCCCC(=O)OCC']

NN-rank 14: ['CCCCCCCCCC=O', 'CCO']

EI-MS matched molecular weight (relative abundance: 14.39%) for CCCCCCCCCC=O

GHS hazard classification: Irritant

EI-MS matched molecular weight (relative abundance: 8.29%) for CCO

GHS hazard classification: none of the hazards selected for correlation

NN-rank 15: ['CC', 'CCCCCCCCC', 'O=CO']

EI-MS matched molecular weight (relative abundance: 22.59%) for CC

GHS hazard classification: none of the hazards selected for correlation

EI-MS matched molecular weight (relative abundance: 8.29%) for O=CO

GHS hazard classification: none of the hazards selected for correlation

NN-rank 16: ['CCC1CCCCCCCCC(=O)O1']

NN-rank 17: ['CCCCCCCCCCOCC', 'O']

NN-rank 18: ['CC', 'CCCCCCCCCCO', 'O']

EI-MS matched molecular weight (relative abundance: 22.59%) for CC

GHS hazard classification: none of the hazards selected for correlation

EI-MS matched molecular weight (relative abundance: 9.89%) for CCCCCCCCCCO

GHS hazard classification: Irritant

NN-rank 19: ['CCCCCCCC(C)C(=O)OCC']

NN-rank 20: ['O=C1CCCCCCCCCCCO1']

NN/MS matches corresponding with tox GHS classification highlight in Bold font.

NN-rank 21: ['CC', 'CCCCCCCC', 'O=C1CO1']

EI-MS matched molecular weight (relative abundance: 22.59%) for CC

GHS hazard classification: none of the hazards selected for correlation

EI-MS matched molecular weight (relative abundance: 11.39%) for O=C1CO1

GHS hazard classification: none of the hazards selected for correlation

NN-rank 22: ['CC1CCCCCCCC(=O)OCC1']

NN-rank 23: ['CC', 'CCCCCCCC(=O)OCC']

EI-MS matched molecular weight (relative abundance: 22.59%) for CC

GHS hazard classification: none of the hazards selected for correlation

NN-rank 24: ['CC', 'O=C1OC2CCCCCCCC12']

EI-MS matched molecular weight (relative abundance: 22.59%) for CC

GHS hazard classification: none of the hazards selected for correlation

NN-rank 25: ['CCCCCCCCC(=O)OCCC']

NN/MS matches corresponding with tox GHS classification highlight in Bold font.

96. Ethyl Dodecanoate: CCCCCCCCCCCC(=O)OCC

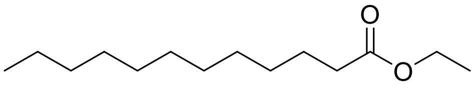

Summary Report of GHS Classification for NN/MS Matches:

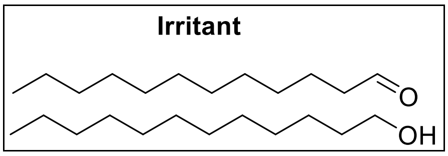

NN-rank 1: ['CC', 'CCCCCCCCCCCC(=O)O']

EI-MS matched molecular weight (relative abundance: 19.99%) for CC

GHS hazard classification: none of the hazards selected for correlation

NN-rank 2: ['CC', 'CCCCCCCCCCCC(=O)O']

EI-MS matched molecular weight (relative abundance: 19.99%) for CC

GHS hazard classification: none of the hazards selected for correlation

NN-rank 3: ['CC', 'CCCCCCCCCCC(=O)OC']

EI-MS matched molecular weight (relative abundance: 19.99%) for CC

GHS hazard classification: none of the hazards selected for correlation

NN-rank 4: ['CC', 'CCCCCCCCCCCC=O', 'O']

EI-MS matched molecular weight (relative abundance: 19.99%) for CC

GHS hazard classification: none of the hazards selected for correlation

EI-MS matched molecular weight (relative abundance: 13.19%) for CCCCCCCCCCCC=O

GHS hazard classification: Irritant

NN-rank 5: ['CC', 'O=C1CCCCCCCCCCCO1']

EI-MS matched molecular weight (relative abundance: 19.99%) for CC

GHS hazard classification: none of the hazards selected for correlation

NN-rank 6: ['CCCCCCCCCCCCO', 'CCO']

EI-MS matched molecular weight (relative abundance: 7.29%) for CCCCCCCCCCCCO

GHS hazard classification: Irritant

EI-MS matched molecular weight (relative abundance: 6.39%) for CCO

GHS hazard classification: none of the hazards selected for correlation

NN-rank 7: ['CC', 'O=C1CCCCCCCCCCCO1']

EI-MS matched molecular weight (relative abundance: 19.99%) for CC

NN/MS matches corresponding with tox GHS classification highlight in Bold font.

GHS hazard classification: none of the hazards selected for correlation

NN-rank 8: ['CC', 'CCCCCCCCCCC1OC1=O']

EI-MS matched molecular weight (relative abundance: 19.99%) for CC

GHS hazard classification: none of the hazards selected for correlation

NN-rank 9: ['CC', 'OCCCCCCCCCCCCO']

EI-MS matched molecular weight (relative abundance: 19.99%) for CC

GHS hazard classification: none of the hazards selected for correlation

NN-rank 10: ['CC', 'CCCCCCCCCC(=O)OC']

EI-MS matched molecular weight (relative abundance: 19.99%) for CC

GHS hazard classification: none of the hazards selected for correlation

NN-rank 11: ['CC', 'CCCCCCCCCCCO', 'CO']

EI-MS matched molecular weight (relative abundance: 19.99%) for CC

GHS hazard classification: none of the hazards selected for correlation

NN-rank 12: ['CC', 'O=CCCCCCCCCCCCO']

EI-MS matched molecular weight (relative abundance: 19.99%) for CC

GHS hazard classification: none of the hazards selected for correlation

NN-rank 13: ['C', 'CCCCCCCCCCC(=O)OCC']

NN-rank 14: ['CCCCCCCCCCCC=O', 'CCO']

EI-MS matched molecular weight (relative abundance: 13.19%) for CCCCCCCCCCCC=O

GHS hazard classification: Irritant

EI-MS matched molecular weight (relative abundance: 6.39%) for CCO

GHS hazard classification: none of the hazards selected for correlation

NN-rank 15: ['CC', 'CCCCCCCCCCC', 'O=CO']

EI-MS matched molecular weight (relative abundance: 19.99%) for CC

GHS hazard classification: none of the hazards selected for correlation

EI-MS matched molecular weight (relative abundance: 6.39%) for O=CO

GHS hazard classification: none of the hazards selected for correlation

NN-rank 16: ['CCC1CCCCCCCCCCC(=O)O1']

NN-rank 17: ['CCCCCCCCCCCCCOCC', 'O']

NN-rank 18: ['CCCCCCCCCC(C)C(=O)OCC']

NN-rank 19: ['CC', 'CCCCCCCCCCCCO', 'O']

EI-MS matched molecular weight (relative abundance: 19.99%) for CC

GHS hazard classification: none of the hazards selected for correlation

EI-MS matched molecular weight (relative abundance: 7.29%) for CCCCCCCCCCCCO

NN/MS matches corresponding with tox GHS classification highlight in Bold font.

GHS hazard classification: Irritant

NN-rank 20: ['O=C1CCCCCCCCCCCCCO1']

NN-rank 21: ['CC', 'CCCCCCCCCC', 'O=C1CO1']

EI-MS matched molecular weight (relative abundance: 19.99%) for CC

GHS hazard classification: none of the hazards selected for correlation

EI-MS matched molecular weight (relative abundance: 13.09%) for O=C1CO1

GHS hazard classification: none of the hazards selected for correlation

NN-rank 22: ['CC1CCCCCCCCCC(=O)OCC1']

NN-rank 23: ['CC', 'O=C1OC2CCCCCCCCCC12']

EI-MS matched molecular weight (relative abundance: 19.99%) for CC

GHS hazard classification: none of the hazards selected for correlation

NN-rank 24: ['CC', 'CCCCCCCCCC(=O)OCC']

EI-MS matched molecular weight (relative abundance: 19.99%) for CC

GHS hazard classification: none of the hazards selected for correlation

NN-rank 25: ['CCCCCCCCCCC(=O)OCCC']

NN/MS matches corresponding with tox GHS classification highlight in Bold font.

97. Ethyl Heptanoate: CCCCCCC(=O)OCC

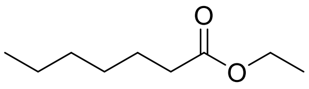

Summary Report of GHS Classification for NN/MS Matches:

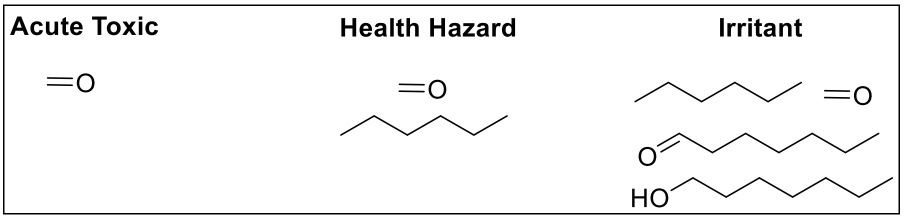

NN-rank 1: ['CC', 'CCCCCCC(=O)O']

EI-MS matched molecular weight (relative abundance: 13.91%) for CC

GHS hazard classification: none of the hazards selected for correlation

NN-rank 2: ['CC', 'CCCCCCC(=O)O']

EI-MS matched molecular weight (relative abundance: 13.91%) for CC

GHS hazard classification: none of the hazards selected for correlation

NN-rank 3: ['CC', 'CCCCCCC=O', 'O']

EI-MS matched molecular weight (relative abundance: 13.91%) for CC

GHS hazard classification: none of the hazards selected for correlation

EI-MS matched molecular weight (relative abundance: 45.64%) for CCCCCCC=O

GHS hazard classification: Irritant

NN-rank 4: ['CC', 'O=C1CCCCCCO1']

EI-MS matched molecular weight (relative abundance: 13.91%) for CC

GHS hazard classification: none of the hazards selected for correlation

NN-rank 5: ['CCCCCCCO', 'CCO']

EI-MS matched molecular weight (relative abundance: 19.02%) for CCCCCCCO

GHS hazard classification: Irritant

EI-MS matched molecular weight (relative abundance: 8.11%) for CCO

GHS hazard classification: none of the hazards selected for correlation

NN-rank 6: ['CC', 'CCCCCC(=O)OC']

EI-MS matched molecular weight (relative abundance: 13.91%) for CC

GHS hazard classification: none of the hazards selected for correlation

NN-rank 7: ['CC', 'CCCCCC1OC1=O']

EI-MS matched molecular weight (relative abundance: 13.91%) for CC

NN/MS matches corresponding with tox GHS classification highlight in Bold font.

GHS hazard classification: none of the hazards selected for correlation

NN-rank 8: ['C', 'CCCCCC(=O)OCC']

NN-rank 9: ['CCCCCCC=O', 'CCO']

EI-MS matched molecular weight (relative abundance: 45.64%) for CCCCCCC=O

GHS hazard classification: Irritant

EI-MS matched molecular weight (relative abundance: 8.11%) for CCO

GHS hazard classification: none of the hazards selected for correlation

NN-rank 10: ['CC', 'CCCCCC', 'O=CO']

EI-MS matched molecular weight (relative abundance: 13.91%) for CC

GHS hazard classification: none of the hazards selected for correlation

EI-MS matched molecular weight (relative abundance: 13.11%) for CCCCCC

GHS hazard classification: Health Hazard, Irritant

EI-MS matched molecular weight (relative abundance: 8.11%) for O=CO

GHS hazard classification: none of the hazards selected for correlation

NN-rank 11: ['CCC1CCCCCC(=O)O1']

NN-rank 12: ['CC', 'CCCCCCCO', 'O']

EI-MS matched molecular weight (relative abundance: 13.91%) for CC

GHS hazard classification: none of the hazards selected for correlation

EI-MS matched molecular weight (relative abundance: 19.02%) for CCCCCCCO

GHS hazard classification: Irritant

NN-rank 13: ['CCCCCCCOCC', 'O']

NN-rank 14: ['CC', 'CCCCCC', 'O=C=O']

EI-MS matched molecular weight (relative abundance: 13.91%) for CC

GHS hazard classification: none of the hazards selected for correlation

EI-MS matched molecular weight (relative abundance: 13.11%) for CCCCCC

GHS hazard classification: Health Hazard, Irritant

EI-MS matched molecular weight (relative abundance: 8.01%) for O=C=O

GHS hazard classification: none of the hazards selected for correlation

NN-rank 15: ['O=C1CCCCCCCCO1']

NN-rank 16: ['CC', 'CCCCC', 'O=C1CO1']

EI-MS matched molecular weight (relative abundance: 13.91%) for CC

GHS hazard classification: none of the hazards selected for correlation

EI-MS matched molecular weight (relative abundance: 9.21%) for O=C1CO1

GHS hazard classification: none of the hazards selected for correlation

NN-rank 17: ['CC1CCCCC(=O)OCC1']

NN/MS matches corresponding with tox GHS classification highlight in Bold font.

NN-rank 18: ['CC', 'CCCCC(=O)OCC']

EI-MS matched molecular weight (relative abundance: 13.91%) for CC

GHS hazard classification: none of the hazards selected for correlation

NN-rank 19: ['CCCCCC(=O)OCCC']

NN-rank 20: ['CCCCC(C)C(=O)OCC']

NN-rank 21: ['CC', 'O=CCCCCCCO']

EI-MS matched molecular weight (relative abundance: 13.91%) for CC

GHS hazard classification: none of the hazards selected for correlation

NN-rank 22: ['CC', 'O=C1OC2CCCCC12']

EI-MS matched molecular weight (relative abundance: 13.91%) for CC

GHS hazard classification: none of the hazards selected for correlation

NN-rank 23: ['C=O', 'CC', 'CCCCCC', 'O']

EI-MS matched molecular weight (relative abundance: 13.91%) for C=O

GHS hazard classification: Acute Toxic, Health Hazard, Irritant

EI-MS matched molecular weight (relative abundance: 13.91%) for CC

GHS hazard classification: none of the hazards selected for correlation

EI-MS matched molecular weight (relative abundance: 13.11%) for CCCCCC

GHS hazard classification: Health Hazard, Irritant

NN-rank 24: ['CC', 'CCCCCC(O)C=O']

EI-MS matched molecular weight (relative abundance: 13.91%) for CC

GHS hazard classification: none of the hazards selected for correlation

NN-rank 25: ['C1CCCOCCC1', 'CCO']

EI-MS matched molecular weight (relative abundance: 45.64%) for C1CCCOCCC1

GHS hazard classification: none of the hazards selected for correlation

EI-MS matched molecular weight (relative abundance: 8.11%) for CCO

GHS hazard classification: none of the hazards selected for correlation

NN/MS matches corresponding with tox GHS classification highlight in Bold font.

98. Ethyl Hexanoate: CCCCCC(=O)OCC

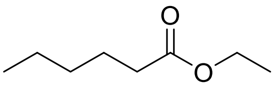

Summary Report of GHS Classification for NN/MS Matches:

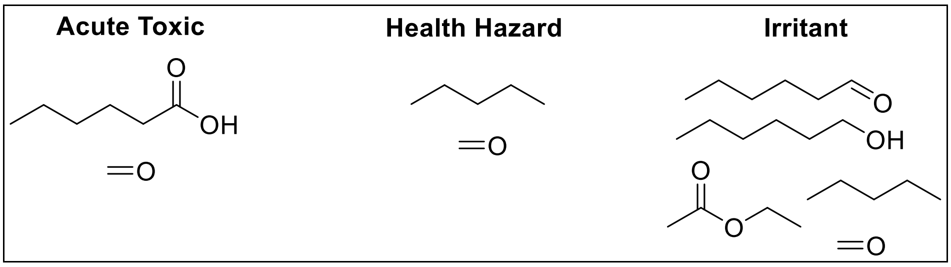

NN-rank 1: ['CC', 'CCCCCC(=O)O']

EI-MS matched molecular weight (relative abundance: 98.49%) for CC

GHS hazard classification: none of the hazards selected for correlation

EI-MS matched molecular weight (relative abundance: 8.41%) for CCCCCC(=O)O

GHS hazard classification: Acute Toxic

NN-rank 2: ['CC', 'CCCCCC(=O)O']

EI-MS matched molecular weight (relative abundance: 98.49%) for CC

GHS hazard classification: none of the hazards selected for correlation

EI-MS matched molecular weight (relative abundance: 8.41%) for CCCCCC(=O)O

GHS hazard classification: Acute Toxic

NN-rank 3: ['CC', 'CCCCCC=O', 'O']

EI-MS matched molecular weight (relative abundance: 98.49%) for CC

GHS hazard classification: none of the hazards selected for correlation

EI-MS matched molecular weight (relative abundance: 53.95%) for CCCCCC=O

GHS hazard classification: Irritant

NN-rank 4: ['CC', 'O=C1CCCCCO1']

EI-MS matched molecular weight (relative abundance: 98.49%) for CC

GHS hazard classification: none of the hazards selected for correlation

NN-rank 5: ['CCCCCCO', 'CCO']

EI-MS matched molecular weight (relative abundance: 26.22%) for CCCCCCO

GHS hazard classification: Irritant

EI-MS matched molecular weight (relative abundance: 23.02%) for CCO

GHS hazard classification: none of the hazards selected for correlation

NN/MS matches corresponding with tox GHS classification highlight in Bold font.

NN-rank 6: ['CC', 'CCCCC(=O)OC']

EI-MS matched molecular weight (relative abundance: 98.49%) for CC

GHS hazard classification: none of the hazards selected for correlation

EI-MS matched molecular weight (relative abundance: 8.41%) for CCCCC(=O)OC

GHS hazard classification: none of the hazards selected for correlation

NN-rank 7: ['CC', 'CCCCC1OC1=O']

EI-MS matched molecular weight (relative abundance: 98.49%) for CC

GHS hazard classification: none of the hazards selected for correlation

NN-rank 8: ['C', 'CCCCC(=O)OCC']

EI-MS matched molecular weight (relative abundance: 12.01%) for C

GHS hazard classification: none of the hazards selected for correlation

NN-rank 9: ['CCCCCC=O', 'CCO']

EI-MS matched molecular weight (relative abundance: 53.95%) for CCCCCC=O

GHS hazard classification: Irritant

EI-MS matched molecular weight (relative abundance: 23.02%) for CCO

GHS hazard classification: none of the hazards selected for correlation

NN-rank 10: ['CC', 'CCCCC', 'O=CO']

EI-MS matched molecular weight (relative abundance: 98.49%) for CC

GHS hazard classification: none of the hazards selected for correlation

EI-MS matched molecular weight (relative abundance: 26.82%) for CCCCC

GHS hazard classification: Health Hazard, Irritant

EI-MS matched molecular weight (relative abundance: 23.02%) for O=CO

GHS hazard classification: none of the hazards selected for correlation

NN-rank 11: ['CC', 'CCCCCCO', 'O']

EI-MS matched molecular weight (relative abundance: 98.49%) for CC

GHS hazard classification: none of the hazards selected for correlation

EI-MS matched molecular weight (relative abundance: 26.22%) for CCCCCCO

GHS hazard classification: Irritant

NN-rank 12: ['CC', 'CCCCC', 'O=C=O']

EI-MS matched molecular weight (relative abundance: 98.49%) for CC

GHS hazard classification: none of the hazards selected for correlation

EI-MS matched molecular weight (relative abundance: 26.82%) for CCCCC

GHS hazard classification: Health Hazard, Irritant

EI-MS matched molecular weight (relative abundance: 35.03%) for O=C=O

GHS hazard classification: none of the hazards selected for correlation

NN/MS matches corresponding with tox GHS classification highlight in Bold font.

NN-rank 13: ['CCC1CCCCC(=O)O1']

NN-rank 14: ['CCCCCCOCC', 'O']

NN-rank 15: ['O=C1CCCCCCCO1']

NN-rank 16: ['CC', 'CCCC(=O)OCC']

EI-MS matched molecular weight (relative abundance: 98.49%) for CC

GHS hazard classification: none of the hazards selected for correlation

EI-MS matched molecular weight (relative abundance: 8.41%) for CCCC(=O)OCC

GHS hazard classification: none of the hazards selected for correlation

NN-rank 17: ['CC', 'CCCC', 'O=C1CO1']

EI-MS matched molecular weight (relative abundance: 98.49%) for CC

GHS hazard classification: none of the hazards selected for correlation

EI-MS matched molecular weight (relative abundance: 5.2%) for CCCC

GHS hazard classification: none of the hazards selected for correlation

EI-MS matched molecular weight (relative abundance: 5.2%) for O=C1CO1

GHS hazard classification: none of the hazards selected for correlation

NN-rank 18: ['CC1CCCC(=O)OCC1']

NN-rank 19: ['CCCCC(=O)OCCC']

NN-rank 20: ['CCC', 'CCOC(=O)CC']

EI-MS matched molecular weight (relative abundance: 85.98%) for CCC

GHS hazard classification: none of the hazards selected for correlation

EI-MS matched molecular weight (relative abundance: 26.22%) for CCOC(=O)CC

GHS hazard classification: none of the hazards selected for correlation

NN-rank 21: ['CC', 'O=CCCCCCO']

EI-MS matched molecular weight (relative abundance: 98.49%) for CC

GHS hazard classification: none of the hazards selected for correlation

EI-MS matched molecular weight (relative abundance: 8.41%) for O=CCCCCCO

GHS hazard classification: Irritant

NN-rank 22: ['C=O', 'CC', 'CCCCC', 'O']

EI-MS matched molecular weight (relative abundance: 98.49%) for C=O

GHS hazard classification: Acute Toxic, Health Hazard, Irritant

EI-MS matched molecular weight (relative abundance: 98.49%) for CC

GHS hazard classification: none of the hazards selected for correlation

EI-MS matched molecular weight (relative abundance: 26.82%) for CCCCC

GHS hazard classification: Health Hazard, Irritant

NN-rank 23: ['CC', 'CCCCC(O)C=O']

NN/MS matches corresponding with tox GHS classification highlight in Bold font.

EI-MS matched molecular weight (relative abundance: 98.49%) for CC

GHS hazard classification: none of the hazards selected for correlation

EI-MS matched molecular weight (relative abundance: 8.41%) for CCCCC(O)C=O

GHS hazard classification: none of the hazards selected for correlation

NN-rank 24: ['CCCC(C)C(=O)OCC']

NN-rank 25: ['CCCC', 'CCOC(C)=O']

EI-MS matched molecular weight (relative abundance: 5.2%) for CCCC

GHS hazard classification: none of the hazards selected for correlation

EI-MS matched molecular weight (relative abundance: 7.31%) for CCOC(C)=O

GHS hazard classification: Irritant

NN/MS matches corresponding with tox GHS classification highlight in Bold font.

99. Ethyl Isovalerate: CCOC(=O)CC(C)C

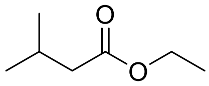

Summary Report of GHS Classification for NN/MS Matches:

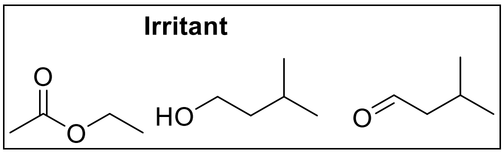

NN-rank 1: ['CC', 'CC(C)CC(=O)O']

EI-MS matched molecular weight (relative abundance: 88.59%) for CC

GHS hazard classification: none of the hazards selected for correlation

NN-rank 2: ['CC', 'CC(C)CC(=O)O']

EI-MS matched molecular weight (relative abundance: 88.59%) for CC

GHS hazard classification: none of the hazards selected for correlation

NN-rank 3: ['CC', 'CC(C)CC=O', 'O']

EI-MS matched molecular weight (relative abundance: 88.59%) for CC

GHS hazard classification: none of the hazards selected for correlation

EI-MS matched molecular weight (relative abundance: 81.89%) for CC(C)CC=O

GHS hazard classification: Irritant

NN-rank 4: ['CC(C)CCO', 'CCO']

EI-MS matched molecular weight (relative abundance: 15.59%) for CC(C)CCO

GHS hazard classification: Irritant

EI-MS matched molecular weight (relative abundance: 26.39%) for CCO

GHS hazard classification: none of the hazards selected for correlation

NN-rank 5: ['CCC(C(=O)O)C(C)C']

NN-rank 6: ['C', 'CCCC(=O)OCC']

EI-MS matched molecular weight (relative abundance: 10.29%) for CCCC(=O)OCC

GHS hazard classification: none of the hazards selected for correlation

NN-rank 7: ['C', 'CCCC(=O)OCC']

EI-MS matched molecular weight (relative abundance: 10.29%) for CCCC(=O)OCC

GHS hazard classification: none of the hazards selected for correlation

NN-rank 8: ['CCOC(=O)C=C(C)C']

NN-rank 9: ['CC(C)CC=O', 'CCO']

NN/MS matches corresponding with tox GHS classification highlight in Bold font.

EI-MS matched molecular weight (relative abundance: 81.89%) for CC(C)CC=O

GHS hazard classification: Irritant

EI-MS matched molecular weight (relative abundance: 26.39%) for CCO

GHS hazard classification: none of the hazards selected for correlation

NN-rank 10: ['CC', 'CC(C)C=C=O', 'O']

EI-MS matched molecular weight (relative abundance: 88.59%) for CC

GHS hazard classification: none of the hazards selected for correlation

NN-rank 11: ['CC(C)C1CCOC1=O']

NN-rank 12: ['CCC(C(=O)O)C(C)C']

NN-rank 13: ['C=C(C)CC(=O)OCC']

NN-rank 14: ['C=C(C)CC(=O)OCC']

NN-rank 15: ['CCOCCC(C)C', 'O']

EI-MS matched molecular weight (relative abundance: 10.29%) for CCOCCC(C)C

GHS hazard classification: none of the hazards selected for correlation

NN-rank 16: ['CC', 'CC(C)CCO', 'O']

EI-MS matched molecular weight (relative abundance: 88.59%) for CC

GHS hazard classification: none of the hazards selected for correlation

EI-MS matched molecular weight (relative abundance: 15.59%) for CC(C)CCO

GHS hazard classification: Irritant

NN-rank 17: ['CC1(C)CCOC(=O)C1']

NN-rank 18: ['CCC(C(=O)O)=C(C)C']

NN-rank 19: ['CCC', 'CCOC(C)=O']

EI-MS matched molecular weight (relative abundance: 32.19%) for CCC

GHS hazard classification: none of the hazards selected for correlation

EI-MS matched molecular weight (relative abundance: 15.59%) for CCOC(C)=O

GHS hazard classification: Irritant

NN-rank 20: ['C', 'COC(=O)CC(C)C']

EI-MS matched molecular weight (relative abundance: 10.29%) for COC(=O)CC(C)C

GHS hazard classification: none of the hazards selected for correlation

NN-rank 21: ['CC(C)C=CO', 'CCO']

EI-MS matched molecular weight (relative abundance: 81.89%) for CC(C)C=CO

GHS hazard classification: none of the hazards selected for correlation

EI-MS matched molecular weight (relative abundance: 26.39%) for CCO

GHS hazard classification: none of the hazards selected for correlation

NN-rank 22: ['C', 'C', 'CC(C)CC(=O)O']

NN/MS matches corresponding with tox GHS classification highlight in Bold font.

NN-rank 23: ['CC(C)=C1CCOC1=O']

NN-rank 24: ['CC(C)C1(C(=O)O)CC1']

NN-rank 25: ['CCC(C=O)C(C)C', 'O']

NN/MS matches corresponding with tox GHS classification highlight in Bold font.

100. Ethyl Lactate: CCOC(=O)C(C)O

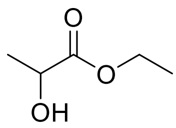

NN-rank 1: ['CC', 'CC(O)C(=O)O']

EI-MS matched molecular weight (relative abundance: 15.0%) for CC

GHS hazard classification: none of the hazards selected for correlation

NN-rank 2: ['CC', 'CC(O)C(=O)O']

EI-MS matched molecular weight (relative abundance: 15.0%) for CC

GHS hazard classification: none of the hazards selected for correlation

NN-rank 3: ['CC', 'CC(O)C=O', 'O']

EI-MS matched molecular weight (relative abundance: 15.0%) for CC

GHS hazard classification: none of the hazards selected for correlation

NN-rank 4: ['CCOC(=O)C(C)O']

NN-rank 5: ['CCOC(=O)CC', 'O']

NN-rank 6: ['C', 'COC(=O)C(C)O']

NN-rank 7: ['CC(O)C=O', 'CCO']

EI-MS matched molecular weight (relative abundance: 99.9%) for CCO

GHS hazard classification: none of the hazards selected for correlation

NN-rank 8: ['CC(O)CO', 'CCO']

EI-MS matched molecular weight (relative abundance: 99.9%) for CCO

GHS hazard classification: none of the hazards selected for correlation

NN-rank 9: ['CC', 'CC1OC1=O', 'O']

EI-MS matched molecular weight (relative abundance: 15.0%) for CC

GHS hazard classification: none of the hazards selected for correlation

NN-rank 10: ['CCO', 'CCOC=O']

EI-MS matched molecular weight (relative abundance: 99.9%) for CCO

GHS hazard classification: none of the hazards selected for correlation

NN-rank 11: ['CCOC(=O)C(C)=O']

NN-rank 12: ['CC(O)C=O', 'CCO']

EI-MS matched molecular weight (relative abundance: 99.9%) for CCO

GHS hazard classification: none of the hazards selected for correlation

NN-rank 13: ['CCOCC(C)O', 'O']

NN-rank 14: ['C#CC(=O)OCC', 'O']

NN/MS matches corresponding with tox GHS classification highlight in Bold font.

NN-rank 15: ['CC', 'CC1OC1=O', 'O']

EI-MS matched molecular weight (relative abundance: 15.0%) for CC

GHS hazard classification: none of the hazards selected for correlation

NN-rank 16: ['CC', 'CC1(O)OC1=O']

EI-MS matched molecular weight (relative abundance: 15.0%) for CC

GHS hazard classification: none of the hazards selected for correlation

NN-rank 17: ['CC', 'O', 'O=C1CC1O']

EI-MS matched molecular weight (relative abundance: 15.0%) for CC

GHS hazard classification: none of the hazards selected for correlation

NN-rank 18: ['C', 'CCOC(=O)CO']

NN-rank 19: ['CC1OC1=O', 'CCO']

EI-MS matched molecular weight (relative abundance: 99.9%) for CCO

GHS hazard classification: none of the hazards selected for correlation

NN-rank 20: ['CC', 'CCO', 'O=C=O']

EI-MS matched molecular weight (relative abundance: 15.0%) for CC

GHS hazard classification: none of the hazards selected for correlation

EI-MS matched molecular weight (relative abundance: 99.9%) for CCO

GHS hazard classification: none of the hazards selected for correlation

NN-rank 21: ['CCO', 'O=C1CC1O']

EI-MS matched molecular weight (relative abundance: 99.9%) for CCO

GHS hazard classification: none of the hazards selected for correlation

NN-rank 22: ['CC', 'CCO', 'O=CO']

EI-MS matched molecular weight (relative abundance: 15.0%) for CC

GHS hazard classification: none of the hazards selected for correlation

EI-MS matched molecular weight (relative abundance: 99.9%) for CCO

GHS hazard classification: none of the hazards selected for correlation

EI-MS matched molecular weight (relative abundance: 99.9%) for O=CO

GHS hazard classification: none of the hazards selected for correlation

NN-rank 23: ['C1CO1', 'CC(O)C=O']

NN-rank 24: ['CC(O)CO', 'CCO']

EI-MS matched molecular weight (relative abundance: 99.9%) for CCO

GHS hazard classification: none of the hazards selected for correlation

NN-rank 25: ['CC(O)C=O', 'CC=O']

NN/MS matches corresponding with tox GHS classification highlight in Bold font.

101. Ethyl maltol: CCC1=C(C(=O)C=CO1)O

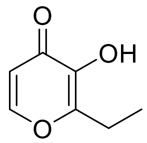

Summary Report of GHS Classification for NN/MS Matches:

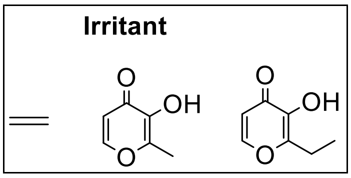

NN-rank 1: ['C', 'Cc1occc(=O)c1O']

EI-MS matched molecular weight (relative abundance: 21.39%) for Cc1occc(=O)c1O

GHS hazard classification: Irritant

NN-rank 2: ['CCc1cc(=O)cco1', 'O']

NN-rank 3: ['CC', 'O=c1ccocc1O']

EI-MS matched molecular weight (relative abundance: 9.19%) for CC

GHS hazard classification: none of the hazards selected for correlation

NN-rank 4: ['Cc1occ(C)c(=O)c1O']

EI-MS matched molecular weight (relative abundance: 56.99%) for Cc1occ(C)c(=O)c1O

GHS hazard classification: none of the hazards selected for correlation

NN-rank 5: ['O=c1c2coc(c1O)CC2']

NN-rank 6: [No prediction]

NN-rank 7: [No prediction]

NN-rank 8: ['C=Cc1occc(=O)c1O']

NN-rank 9: ['CC1C2=COC1=C(O)C2=O']

NN-rank 10: ['O=c1cc2oc(c1O)CC2']

NN-rank 11: ['Cc1cc(=O)c(O)c(C)o1']

EI-MS matched molecular weight (relative abundance: 56.99%) for Cc1cc(=O)c(O)c(C)o1

GHS hazard classification: none of the hazards selected for correlation

NN-rank 12: [No prediction]

NN-rank 13: ['C', 'O=C1C2=COC(=C1O)C2']

NN/MS matches corresponding with tox GHS classification highlight in Bold font.

NN-rank 14: [No prediction]

NN-rank 15: [No prediction]

NN-rank 16: ['OC1=C2CCC1(O)C=CO2']

EI-MS matched molecular weight (relative abundance: 56.99%) for OC1=C2CCC1(O)C=CO2

GHS hazard classification: unknown

NN-rank 17: ['C', 'C', 'O=c1ccocc1O']

NN-rank 18: ['CCc1cocc(O)c1=O']

EI-MS matched molecular weight (relative abundance: 56.99%) for CCc1cocc(O)c1=O

GHS hazard classification: none of the hazards selected for correlation

NN-rank 19: ['CCc1occc(=O)c1O']

EI-MS matched molecular weight (relative abundance: 56.99%) for CCc1occc(=O)c1O

GHS hazard classification: Irritant

NN-rank 20: [No prediction]

NN-rank 21: ['O=c1c2coc(c1O)C=C2']

NN-rank 22: [No prediction]

NN-rank 23: ['CC1=C(O)C(C)(O)C=CO1']

EI-MS matched molecular weight (relative abundance: 8.19%) for CC1=C(O)C(C)(O)C=CO1

GHS hazard classification: unknown

NN-rank 24: ['C=C', 'O=c1ccocc1O']

EI-MS matched molecular weight (relative abundance: 9.49%) for C=C

GHS hazard classification: Irritant

NN-rank 25: ['C', 'Cc1cocc(O)c1=O']

EI-MS matched molecular weight (relative abundance: 21.39%) for Cc1cocc(O)c1=O

GHS hazard classification: none of the hazards selected for correlation

NN/MS matches corresponding with tox GHS classification highlight in Bold font.

102. trans-2-Hexenylacetate: CCC/C=C/COC(C)=O

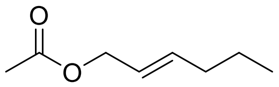

Summary Report of GHS Classification for NN/MS Matches:

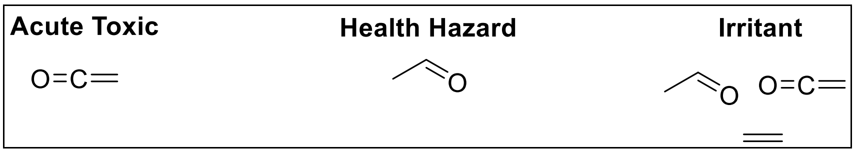

NN-rank 1: ['CC=O', 'CCC/C=C/CO']

EI-MS matched molecular weight (relative abundance: 99.9%) for CC=O

GHS hazard classification: Health Hazard, Irritant

NN-rank 2: ['CC(=O)O', 'C=C=CCCC']

NN-rank 3: ['CCCCC=COC(C)=O']

NN-rank 4: ['CCCC=C=COC(C)=O']

NN-rank 5: ['CC(=O)O', 'C=CCCCC']

NN-rank 6: ['CC(=O)O', 'C=CC=CCC']

NN-rank 7: ['CC(=O)O', 'C/C=C/CCC']

NN-rank 8: ['C', 'CC/C=C/COC(C)=O']

NN-rank 9: ['C=COC(C)=O', 'CCCC']

EI-MS matched molecular weight (relative abundance: 12.0%) for CCCC

GHS hazard classification: none of the hazards selected for correlation

NN-rank 10: ['CC=O', 'CCC/C=C/C=O']

EI-MS matched molecular weight (relative abundance: 99.9%) for CC=O

GHS hazard classification: Health Hazard, Irritant

NN-rank 11: ['CC=O', 'CCCC1C=CO1']

EI-MS matched molecular weight (relative abundance: 99.9%) for CC=O

GHS hazard classification: Health Hazard, Irritant

NN-rank 12: ['CCC=CC=COC(C)=O']

NN-rank 13: ['CC=O', 'CCCC1=CCO1']

EI-MS matched molecular weight (relative abundance: 99.9%) for CC=O

GHS hazard classification: Health Hazard, Irritant

NN-rank 14: ['CCC=CCCOC(C)=O']

NN-rank 15: ['CCCCCCOC(C)=O']

NN-rank 16: ['C/C=C/COC(C)=O', 'CC']

NN/MS matches corresponding with tox GHS classification highlight in Bold font.

NN-rank 17: ['CC(=O)O', 'C#CCCCC']

NN-rank 18: ['CC=O', 'CCCC=C=C=O']

EI-MS matched molecular weight (relative abundance: 99.9%) for CC=O

GHS hazard classification: Health Hazard, Irritant

NN-rank 19: ['C=C', 'CC(=O)O', 'CCCC']

EI-MS matched molecular weight (relative abundance: 13.0%) for C=C

GHS hazard classification: Irritant

EI-MS matched molecular weight (relative abundance: 12.0%) for CCCC

GHS hazard classification: none of the hazards selected for correlation

NN-rank 20: ['CC=O', 'C/C=C(\\O)CCC']

EI-MS matched molecular weight (relative abundance: 99.9%) for CC=O

GHS hazard classification: Health Hazard, Irritant

NN-rank 21: ['CCC=C=CCOC(C)=O']

NN-rank 22: ['C=C=O', 'CCC/C=C/CO']

EI-MS matched molecular weight (relative abundance: 20.0%) for C=C=O

GHS hazard classification: Acute Toxic, Irritant

NN-rank 23: ['CC/C=C(\\C)COC(C)=O']

NN-rank 24: ['CC=O', 'CCCCC=C=O']

EI-MS matched molecular weight (relative abundance: 99.9%) for CC=O

GHS hazard classification: Health Hazard, Irritant

NN-rank 25: ['C=C', 'CC(=O)O', 'C=CCC']

EI-MS matched molecular weight (relative abundance: 13.0%) for C=C

GHS hazard classification: Irritant

EI-MS matched molecular weight (relative abundance: 13.0%) for C=CCC

GHS hazard classification: none of the hazards selected for correlation

NN/MS matches corresponding with tox GHS classification highlight in Bold font.

103. Ethyl methyl phenylglycidate: O=C(C1OC1(C)C2=CC=CC=C2)OCC

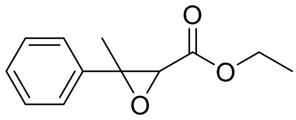

Summary Report of GHS Classification for NN/MS Matches:

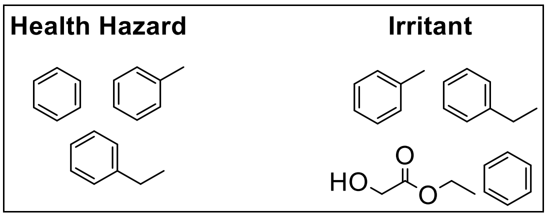

NN-rank 1: ['C', 'CCOC(=O)C(=O)Cc1ccccc1']

NN-rank 2: ['CCOC(=O)C(=O)C(C)c1ccccc1']

EI-MS matched molecular weight (relative abundance: 9.0%) for CCOC(=O)C(=O)C(C)c1ccccc1

GHS hazard classification: none of the hazards selected for correlation

NN-rank 3: ['C', 'CCOC(=O)C(O)Cc1ccccc1']

NN-rank 4: ['CCOC(=O)C(O)C(C)c1ccccc1']

NN-rank 5: ['C=C(c1ccccc1)C(O)C(=O)OCC']

EI-MS matched molecular weight (relative abundance: 9.0%) for C=C(c1ccccc1)C(O)C(=O)OCC

GHS hazard classification: none of the hazards selected for correlation

NN-rank 6: ['C=C(C(=O)C(=O)OCC)c1ccccc1']

NN-rank 7: ['CCOC(=O)C(=O)CC', 'c1ccccc1']

EI-MS matched molecular weight (relative abundance: 45.6%) for c1ccccc1

GHS hazard classification: Health Hazard, Irritant

NN-rank 8: ['CCOC(=O)C(O)CC', 'c1ccccc1']

EI-MS matched molecular weight (relative abundance: 45.6%) for c1ccccc1

GHS hazard classification: Health Hazard, Irritant

NN-rank 9: ['CCOC(=O)C(O)=C(C)c1ccccc1']

EI-MS matched molecular weight (relative abundance: 9.0%) for CCOC(=O)C(O)=C(C)c1ccccc1

GHS hazard classification: unknown

NN-rank 10: ['C', 'CCOC(=O)C(C)=O', 'c1ccccc1']

NN/MS matches corresponding with tox GHS classification highlight in Bold font.

EI-MS matched molecular weight (relative abundance: 45.6%) for c1ccccc1

GHS hazard classification: Health Hazard, Irritant

NN-rank 11: ['CC', 'CC1(c2ccccc2)OC(=O)C1O']

EI-MS matched molecular weight (relative abundance: 24.4%) for CC

GHS hazard classification: none of the hazards selected for correlation

NN-rank 12: ['C', 'CCOC(=O)C1OC1c1ccccc1']

NN-rank 13: ['C', 'CCOC(=O)CO', 'Cc1ccccc1']

EI-MS matched molecular weight (relative abundance: 62.1%) for CCOC(=O)CO

GHS hazard classification: Irritant

EI-MS matched molecular weight (relative abundance: 5.8%) for Cc1ccccc1

GHS hazard classification: Health Hazard, Irritant

NN-rank 14: ['C', 'CCOC(=O)C(O)=Cc1ccccc1']

NN-rank 15: ['C=C(CC(=O)OCC)c1ccccc1', 'O']

NN-rank 16: ['CCOC(=O)CO', 'CCc1ccccc1']

EI-MS matched molecular weight (relative abundance: 62.1%) for CCOC(=O)CO

GHS hazard classification: Irritant

EI-MS matched molecular weight (relative abundance: 63.7%) for CCc1ccccc1

GHS hazard classification: Health Hazard, Irritant

NN-rank 17: ['C', 'CC', 'O=C1OC(c2ccccc2)C1O']

EI-MS matched molecular weight (relative abundance: 24.4%) for CC

GHS hazard classification: none of the hazards selected for correlation

NN-rank 18: ['CCOC(=O)CC(C)(O)c1ccccc1']

NN-rank 19: ['C', 'CCOC(=O)CCc1ccccc1', 'O']

NN-rank 20: ['CCOC(=O)CC(C)c1ccccc1', 'O']

NN-rank 21: ['CCOC(=O)CC(CO)c1ccccc1']

NN-rank 22: ['CC', 'CC1(c2ccccc2)OC1C(=O)O']

EI-MS matched molecular weight (relative abundance: 24.4%) for CC

GHS hazard classification: none of the hazards selected for correlation

NN-rank 23: ['C=CC(O)C(=O)OCC', 'c1ccccc1']

EI-MS matched molecular weight (relative abundance: 45.6%) for c1ccccc1

GHS hazard classification: Health Hazard, Irritant

NN-rank 24: ['C', 'CC', 'O=C1OC(c2ccccc2)C1=O']

EI-MS matched molecular weight (relative abundance: 24.4%) for CC

GHS hazard classification: none of the hazards selected for correlation

EI-MS matched molecular weight (relative abundance: 5.8%) for

NN/MS matches corresponding with tox GHS classification highlight in Bold font.

O=C1OC(c2ccccc2)C1=O

GHS hazard classification: unknown

NN-rank 25: ['CCOC(=O)C1OCC1c1ccccc1']

EI-MS matched molecular weight (relative abundance: 9.0%) for CCOC(=O)C1OCC1c1ccccc1

GHS hazard classification: unknown

NN/MS matches corresponding with tox GHS classification highlight in Bold font.

104. Ethyl Nonanoate: CCCCCCCCC(=O)OCC

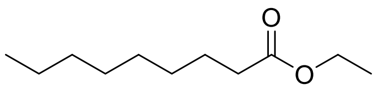

Summary Report of GHS Classification for NN/MS Matches:

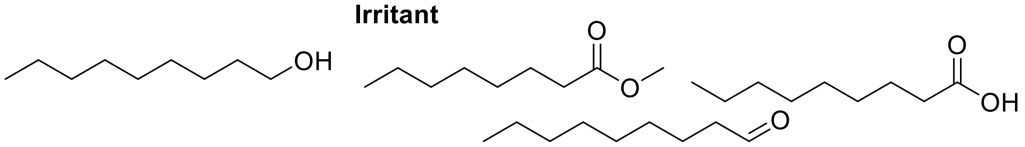

NN-rank 1: ['CC', 'CCCCCCCCC(=O)O']

EI-MS matched molecular weight (relative abundance: 19.79%) for CC

GHS hazard classification: none of the hazards selected for correlation

EI-MS matched molecular weight (relative abundance: 5.89%) for CCCCCCCCC(=O)O

GHS hazard classification: Irritant

NN-rank 2: ['CC', 'CCCCCCCCC(=O)O']

EI-MS matched molecular weight (relative abundance: 19.79%) for CC

GHS hazard classification: none of the hazards selected for correlation

EI-MS matched molecular weight (relative abundance: 5.89%) for CCCCCCCCC(=O)O

GHS hazard classification: Irritant

NN-rank 3: ['CC', 'CCCCCCCCC=O', 'O']

EI-MS matched molecular weight (relative abundance: 19.79%) for CC

GHS hazard classification: none of the hazards selected for correlation

EI-MS matched molecular weight (relative abundance: 18.49%) for CCCCCCCCC=O

GHS hazard classification: Irritant

NN-rank 4: ['CC', 'O=C1CCCCCCCCO1']

EI-MS matched molecular weight (relative abundance: 19.79%) for CC

GHS hazard classification: none of the hazards selected for correlation

NN-rank 5: ['CC', 'CCCCCCCC(=O)OC']

EI-MS matched molecular weight (relative abundance: 19.79%) for CC

GHS hazard classification: none of the hazards selected for correlation

EI-MS matched molecular weight (relative abundance: 5.89%) for CCCCCCCC(=O)OC

GHS hazard classification: Irritant

NN-rank 6: ['CCCCCCCCCO', 'CCO']

EI-MS matched molecular weight (relative abundance: 6.39%) for CCCCCCCCCO

NN/MS matches corresponding with tox GHS classification highlight in Bold font.

GHS hazard classification: Irritant

EI-MS matched molecular weight (relative abundance: 7.89%) for CCO

GHS hazard classification: none of the hazards selected for correlation

NN-rank 7: ['CC', 'O=C1CCCCCCCCO1']

EI-MS matched molecular weight (relative abundance: 19.79%) for CC

GHS hazard classification: none of the hazards selected for correlation

NN-rank 8: ['CC', 'CCCCCCCC1OC1=O']

EI-MS matched molecular weight (relative abundance: 19.79%) for CC

GHS hazard classification: none of the hazards selected for correlation

NN-rank 9: ['CC', 'OCCCCCCCCCO']

EI-MS matched molecular weight (relative abundance: 19.79%) for CC

GHS hazard classification: none of the hazards selected for correlation

NN-rank 10: ['CC', 'CCCCCCCC(=O)OC']

EI-MS matched molecular weight (relative abundance: 19.79%) for CC

GHS hazard classification: none of the hazards selected for correlation

EI-MS matched molecular weight (relative abundance: 5.89%) for CCCCCCCC(=O)OC

GHS hazard classification: Irritant

NN-rank 11: ['CC', 'O=CCCCCCCCCO']

EI-MS matched molecular weight (relative abundance: 19.79%) for CC

GHS hazard classification: none of the hazards selected for correlation

EI-MS matched molecular weight (relative abundance: 5.89%) for O=CCCCCCCCCO

GHS hazard classification: none of the hazards selected for correlation

NN-rank 12: ['CC', 'CCCCCCCCO', 'CO']

EI-MS matched molecular weight (relative abundance: 19.79%) for CC

GHS hazard classification: none of the hazards selected for correlation

NN-rank 13: ['C', 'CCCCCCCC(=O)OCC']

NN-rank 14: ['CCCCCCCCC=O', 'CCO']

EI-MS matched molecular weight (relative abundance: 18.49%) for CCCCCCCCC=O

GHS hazard classification: Irritant

EI-MS matched molecular weight (relative abundance: 7.89%) for CCO

GHS hazard classification: none of the hazards selected for correlation

NN-rank 15: ['CC', 'CCCCCCCC', 'O=CO']

EI-MS matched molecular weight (relative abundance: 19.79%) for CC

GHS hazard classification: none of the hazards selected for correlation

EI-MS matched molecular weight (relative abundance: 7.89%) for O=CO

NN/MS matches corresponding with tox GHS classification highlight in Bold font.

GHS hazard classification: none of the hazards selected for correlation

NN-rank 16: ['CCC1CCCCCCCC(=O)O1']

NN-rank 17: ['CCCCCCCCCOCC', 'O']

NN-rank 18: ['CC', 'CCCCCCCCCO', 'O']

EI-MS matched molecular weight (relative abundance: 19.79%) for CC

GHS hazard classification: none of the hazards selected for correlation

EI-MS matched molecular weight (relative abundance: 6.39%) for CCCCCCCCCO

GHS hazard classification: Irritant

NN-rank 19: ['CCCCCCC(C)C(=O)OCC']

NN-rank 20: ['O=C1CCCCCCCCCCO1']

NN-rank 21: ['CC', 'CCCCCCC', 'O=C1CO1']

EI-MS matched molecular weight (relative abundance: 19.79%) for CC

GHS hazard classification: none of the hazards selected for correlation

EI-MS matched molecular weight (relative abundance: 16.09%) for O=C1CO1

GHS hazard classification: none of the hazards selected for correlation

NN-rank 22: ['CC1CCCCCCC(=O)OCC1']

NN-rank 23: ['CC', 'CCCCCCC(=O)OCC']

EI-MS matched molecular weight (relative abundance: 19.79%) for CC

GHS hazard classification: none of the hazards selected for correlation

EI-MS matched molecular weight (relative abundance: 5.89%) for CCCCCCC(=O)OCC

GHS hazard classification: none of the hazards selected for correlation

NN-rank 24: ['CC', 'O=C1OC2CCCCCCC12']

EI-MS matched molecular weight (relative abundance: 19.79%) for CC

GHS hazard classification: none of the hazards selected for correlation

NN-rank 25: ['CCCCCCCC(=O)OCCC']

NN/MS matches corresponding with tox GHS classification highlight in Bold font.

105. Ethyl Octanoate: CCCCCCCC(=O)OCC

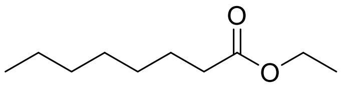

Summary Report of GHS Classification for NN/MS Matches:

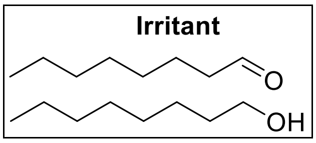

NN-rank 1: ['CC', 'CCCCCCCC(=O)O']

EI-MS matched molecular weight (relative abundance: 22.69%) for CC

GHS hazard classification: none of the hazards selected for correlation

NN-rank 2: ['CC', 'CCCCCCCC(=O)O']

EI-MS matched molecular weight (relative abundance: 22.69%) for CC

GHS hazard classification: none of the hazards selected for correlation

NN-rank 3: ['CC', 'CCCCCCCC=O', 'O']

EI-MS matched molecular weight (relative abundance: 22.69%) for CC

GHS hazard classification: none of the hazards selected for correlation

EI-MS matched molecular weight (relative abundance: 26.59%) for CCCCCCCC=O

GHS hazard classification: Irritant

NN-rank 4: ['CC', 'O=C1CCCCCCCO1']

EI-MS matched molecular weight (relative abundance: 22.69%) for CC

GHS hazard classification: none of the hazards selected for correlation

NN-rank 5: ['CCCCCCCCO', 'CCO']

EI-MS matched molecular weight (relative abundance: 6.89%) for CCCCCCCCO

GHS hazard classification: Irritant

EI-MS matched molecular weight (relative abundance: 9.19%) for CCO

GHS hazard classification: none of the hazards selected for correlation

NN-rank 6: ['CC', 'CCCCCCC(=O)OC']

EI-MS matched molecular weight (relative abundance: 22.69%) for CC

GHS hazard classification: none of the hazards selected for correlation

NN-rank 7: ['CC', 'O=C1CCCCCCCO1']

EI-MS matched molecular weight (relative abundance: 22.69%) for CC

GHS hazard classification: none of the hazards selected for correlation

NN-rank 8: ['CC', 'CCCCCCC1OC1=O']

NN/MS matches corresponding with tox GHS classification highlight in Bold font.

EI-MS matched molecular weight (relative abundance: 22.69%) for CC

GHS hazard classification: none of the hazards selected for correlation

NN-rank 9: ['CC', 'OCCCCCCCCCO']

EI-MS matched molecular weight (relative abundance: 22.69%) for CC

GHS hazard classification: none of the hazards selected for correlation

NN-rank 10: ['CC', 'CCCCCCC(=O)OC']

EI-MS matched molecular weight (relative abundance: 22.69%) for CC

GHS hazard classification: none of the hazards selected for correlation

NN-rank 11: ['CC', 'O=CCCCCCCCO']

EI-MS matched molecular weight (relative abundance: 22.69%) for CC

GHS hazard classification: none of the hazards selected for correlation

NN-rank 12: ['C', 'CCCCCCC(=O)OCC']

NN-rank 13: ['CCCCCCCC=O', 'CCO']

EI-MS matched molecular weight (relative abundance: 26.59%) for CCCCCCCC=O

GHS hazard classification: Irritant

EI-MS matched molecular weight (relative abundance: 9.19%) for CCO

GHS hazard classification: none of the hazards selected for correlation

NN-rank 14: ['CC', 'CCCCCCC', 'O=CO']

EI-MS matched molecular weight (relative abundance: 22.69%) for CC

GHS hazard classification: none of the hazards selected for correlation

EI-MS matched molecular weight (relative abundance: 9.19%) for O=CO

GHS hazard classification: none of the hazards selected for correlation

NN-rank 15: ['CC', 'CCCCCCCO', 'CO']

EI-MS matched molecular weight (relative abundance: 22.69%) for CC

GHS hazard classification: none of the hazards selected for correlation

EI-MS matched molecular weight (relative abundance: 7.19%) for CCCCCCCO

GHS hazard classification: Irritant

NN-rank 16: ['CCC1CCCCCCC(=O)O1']

NN-rank 17: ['CC', 'CCCCCCCCO', 'O']

EI-MS matched molecular weight (relative abundance: 22.69%) for CC

GHS hazard classification: none of the hazards selected for correlation

EI-MS matched molecular weight (relative abundance: 6.89%) for CCCCCCCCO

GHS hazard classification: Irritant

NN-rank 18: ['CCCCCCCCCOCC', 'O']

NN-rank 19: ['CCCCCC(C)C(=O)OCC']

NN/MS matches corresponding with tox GHS classification highlight in Bold font.

NN-rank 20: ['O=C1CCCCCCCCCO1']

NN-rank 21: ['CC', 'CCCCCCC', 'O=C=O']

EI-MS matched molecular weight (relative abundance: 22.69%) for CC

GHS hazard classification: none of the hazards selected for correlation

EI-MS matched molecular weight (relative abundance: 5.69%) for O=C=O

GHS hazard classification: none of the hazards selected for correlation

NN-rank 22: ['CC', 'CCCCCC', 'O=C1CO1']

EI-MS matched molecular weight (relative abundance: 22.69%) for CC

GHS hazard classification: none of the hazards selected for correlation

EI-MS matched molecular weight (relative abundance: 28.59%) for O=C1CO1

GHS hazard classification: none of the hazards selected for correlation

NN-rank 23: ['CC1CCCCCC(=O)OCC1']

NN-rank 24: ['CC', 'CCCCCC(=O)OCC']

EI-MS matched molecular weight (relative abundance: 22.69%) for CC

GHS hazard classification: none of the hazards selected for correlation

NN-rank 25: ['CC', 'O=C1OC2CCCCCC12']

EI-MS matched molecular weight (relative abundance: 22.69%) for CC

GHS hazard classification: none of the hazards selected for correlation

NN/MS matches corresponding with tox GHS classification highlight in Bold font.

106. Ethyl Propionate: CCC(=O)OCC

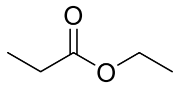

Summary Report of GHS Classification for NN/MS Matches:

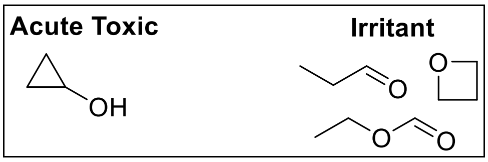

NN-rank 1: ['CC', 'CCC(=O)O']

EI-MS matched molecular weight (relative abundance: 99.99%) for CC

GHS hazard classification: none of the hazards selected for correlation

EI-MS matched molecular weight (relative abundance: 5.24%) for CCC(=O)O

GHS hazard classification: none of the hazards selected for correlation

NN-rank 2: ['CC', 'CCC(=O)O']

EI-MS matched molecular weight (relative abundance: 99.99%) for CC

GHS hazard classification: none of the hazards selected for correlation

EI-MS matched molecular weight (relative abundance: 5.24%) for CCC(=O)O

GHS hazard classification: none of the hazards selected for correlation

NN-rank 3: ['CCCO', 'CCO']

EI-MS matched molecular weight (relative abundance: 6.92%) for CCO

GHS hazard classification: none of the hazards selected for correlation

NN-rank 4: ['CCC=O', 'CCO']

EI-MS matched molecular weight (relative abundance: 59.67%) for CCC=O

GHS hazard classification: Irritant

EI-MS matched molecular weight (relative abundance: 6.92%) for CCO

GHS hazard classification: none of the hazards selected for correlation

NN-rank 5: ['CCO', 'O=C1CC1']

EI-MS matched molecular weight (relative abundance: 6.92%) for CCO

GHS hazard classification: none of the hazards selected for correlation

NN-rank 6: ['CC', 'O=C1CCO1']

EI-MS matched molecular weight (relative abundance: 99.99%) for CC

GHS hazard classification: none of the hazards selected for correlation

NN-rank 7: ['O=C1CCCCO1']

NN-rank 8: ['C', 'CCOC(C)=O']

NN-rank 9: ['CC', 'CCC=O', 'O']

EI-MS matched molecular weight (relative abundance: 99.99%) for CC

GHS hazard classification: none of the hazards selected for correlation

EI-MS matched molecular weight (relative abundance: 59.67%) for CCC=O

GHS hazard classification: Irritant

NN-rank 10: ['CC', 'CC1OC1=O']

EI-MS matched molecular weight (relative abundance: 99.99%) for CC

GHS hazard classification: none of the hazards selected for correlation

NN-rank 11: ['C', 'CCC(=O)OC']

NN-rank 12: ['CC', 'CC1OC1=O']

EI-MS matched molecular weight (relative abundance: 99.99%) for CC

GHS hazard classification: none of the hazards selected for correlation

NN-rank 13: ['CCO', 'OC1CC1']

EI-MS matched molecular weight (relative abundance: 6.92%) for CCO

GHS hazard classification: none of the hazards selected for correlation

EI-MS matched molecular weight (relative abundance: 59.67%) for OC1CC1

GHS hazard classification: Acute Toxic

NN-rank 14: ['C1COC1', 'CCO']

EI-MS matched molecular weight (relative abundance: 59.67%) for C1COC1

GHS hazard classification: Irritant

EI-MS matched molecular weight (relative abundance: 6.92%) for CCO

GHS hazard classification: none of the hazards selected for correlation

NN-rank 15: ['CC', 'O', 'O=C1CC1']

EI-MS matched molecular weight (relative abundance: 99.99%) for CC

GHS hazard classification: none of the hazards selected for correlation

NN-rank 16: ['CCC(=O)CCO']

NN-rank 17: ['CCCOCC', 'O']

NN-rank 18: ['C=CC(=O)OCC']

NN-rank 19: ['CCCOC(C)=O']

NN-rank 20: ['CCCCC(=O)O']

NN-rank 21: ['CCCCC(=O)O']

NN-rank 22: ['CC1CCC(=O)O1']

NN-rank 23: ['CC', 'CCOC=O']

EI-MS matched molecular weight (relative abundance: 99.99%) for CC

NN/MS matches corresponding with tox GHS classification highlight in Bold font.

GHS hazard classification: none of the hazards selected for correlation

EI-MS matched molecular weight (relative abundance: 5.24%) for CCOC=O

GHS hazard classification: Irritant

NN-rank 24: ['CCOC1(O)CC1']

NN-rank 25: ['CC', 'CC(O)CO']

EI-MS matched molecular weight (relative abundance: 99.99%) for CC

GHS hazard classification: none of the hazards selected for correlation

EI-MS matched molecular weight (relative abundance: 9.94%) for CC(O)CO

GHS hazard classification: none of the hazards selected for correlation

NN/MS matches corresponding with tox GHS classification highlight in Bold font.

107. Ethyl Vanillin: CCOC1=C(C=CC(=C1)C=O)O

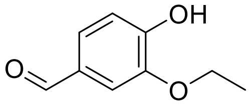

Summary Report of GHS Classification for NN/MS Matches:

Acute Toxic	Health Hazard	Irritant
=O	=O	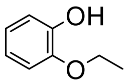 =O
		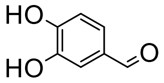 =

NN-rank 1: ['CC', 'O=Cc1ccc(O)c(O)c1']

EI-MS matched molecular weight (relative abundance: 11.8%) for CC

GHS hazard classification: none of the hazards selected for correlation

EI-MS matched molecular weight (relative abundance: 99.99%) for O=Cc1ccc(O)c(O)c1

GHS hazard classification: Irritant

NN-rank 2: ['CCOc1cc(C=O)ccc1O']

NN-rank 3: ['C', 'COc1cc(C=O)ccc1O']

NN-rank 4: ['C=O', 'CCOc1ccccc1O']

EI-MS matched molecular weight (relative abundance: 11.8%) for C=O

GHS hazard classification: Acute Toxic, Health Hazard, Irritant

EI-MS matched molecular weight (relative abundance: 99.99%) for CCOc1ccccc1O

GHS hazard classification: Irritant

NN-rank 5: ['CCOc1cc(CO)ccc1O']

NN-rank 6: ['CCO', 'O=Cc1ccc(O)cc1']

NN-rank 7: ['CCO', 'O=Cc1ccc(O)cc1']

NN-rank 8: ['C=C', 'O=Cc1ccc(O)c(O)c1']

EI-MS matched molecular weight (relative abundance: 11.85%) for C=C

GHS hazard classification: Irritant

EI-MS matched molecular weight (relative abundance: 99.99%) for O=Cc1ccc(O)c(O)c1

GHS hazard classification: Irritant

NN-rank 9: ['CCc1cc(C=O)cc(O)c1O']

NN/MS matches corresponding with tox GHS classification highlight in Bold font.

NN-rank 10: ['C=COc1cc(C=O)ccc1O']

NN-rank 11: ['CCOCc1ccc(O)c(O)c1']

NN-rank 12: ['C1CO1', 'O=Cc1ccc(O)cc1']

NN-rank 13: ['C', 'Cc1cc(C=O)cc(O)c1O']

NN-rank 14: ['CCOc1cc(C)ccc1O', 'O']

NN-rank 15: ['C', 'COCc1ccc(O)c(O)c1']

NN-rank 16: ['C', 'C', 'O=Cc1ccc(O)c(O)c1']

EI-MS matched molecular weight (relative abundance: 99.99%) for O=Cc1ccc(O)c(O)c1

GHS hazard classification: Irritant

NN-rank 17: ['CCOc1cc2cc(c1O)C2=O']

NN-rank 18: ['C=COc1cc(C=O)ccc1O']

NN-rank 19: ['CCOc1cccc(C=O)c1', 'O']

NN-rank 20: ['CCOc1cc2cc(c1O)C2O']

NN-rank 21: ['CC1Oc2cc(C=O)cc1c2O']

NN-rank 22: ['CCOc1ccccc1O', 'CO']

EI-MS matched molecular weight (relative abundance: 99.99%) for CCOc1ccccc1O

GHS hazard classification: Irritant

NN-rank 23: ['C=Cc1cc(C=O)cc(O)c1O']

NN-rank 24: ['C', 'O=Cc1cc2c(O)c(c1)OC2']

NN-rank 25: ['CC=O', 'O=Cc1ccc(O)cc1']

NN/MS matches corresponding with tox GHS classification highlight in Bold font.

108. Ethyl Vanillin Propylene Glycol Acetal: CCOC1=C(C=CC(=C1)C2OCC(O2)C)O

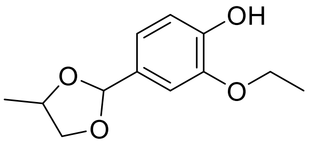

Summary Report of GHS Classification for NN/MS Matches:

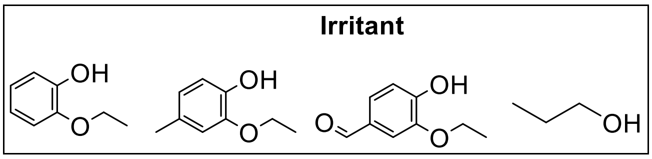

NN-rank 1: ['CC(O)CO', 'CCOc1cc(C)ccc1O']

EI-MS matched molecular weight (relative abundance: 23.0%) for CCOc1cc(C)ccc1O

GHS hazard classification: Irritant

NN-rank 2: ['CCOc1cc(COC(C)CO)ccc1O']

NN-rank 3: ['CCOc1cc(COCC(C)O)ccc1O']

NN-rank 4: ['CC(C)O', 'CCOc1cc(C=O)ccc1O']

EI-MS matched molecular weight (relative abundance: 39.0%) for CC(C)O

GHS hazard classification: none of the hazards selected for correlation

EI-MS matched molecular weight (relative abundance: 99.9%) for CCOc1cc(C=O)ccc1O

GHS hazard classification: Irritant

NN-rank 5: ['CCCO', 'CCOc1cc(C=O)ccc1O']

EI-MS matched molecular weight (relative abundance: 39.0%) for CCCO

GHS hazard classification: Irritant

EI-MS matched molecular weight (relative abundance: 99.9%) for CCOc1cc(C=O)ccc1O

GHS hazard classification: Irritant

NN-rank 6: ['CC', 'CC1COC(c2ccc(O)c(O)c2)O1']

NN-rank 7: ['C', 'COc1cc(C2OCC(C)O2)ccc1O']

NN-rank 8: ['CCOc1cc(COC(C)C=O)ccc1O']

EI-MS matched molecular weight (relative abundance: 73.0%) for CCOc1cc(COC(C)C=O)ccc1O

GHS hazard classification: unknown

NN/MS matches corresponding with tox GHS classification highlight in Bold font.

NN-rank 9: ['CCCOC(O)c1ccc(O)c(OCC)c1']

NN-rank 10: ['CC(O)C=O', 'CCOc1cc(C)ccc1O']

EI-MS matched molecular weight (relative abundance: 23.0%) for CCOc1cc(C)ccc1O

GHS hazard classification: Irritant

NN-rank 11: ['CCOc1cc(C(O)OC(C)C)ccc1O']

NN-rank 12: ['CC(=O)CO', 'CCOc1cc(C)ccc1O']

EI-MS matched molecular weight (relative abundance: 23.0%) for CCOc1cc(C)ccc1O

GHS hazard classification: Irritant

NN-rank 13: ['CCOc1cc(COC2(C)CO2)ccc1O']

EI-MS matched molecular weight (relative abundance: 73.0%) for CCOc1cc(COC2(C)CO2)ccc1O

GHS hazard classification: unknown

NN-rank 14: ['CCCOC(=O)c1ccc(O)c(OCC)c1']

EI-MS matched molecular weight (relative abundance: 73.0%) for CCCOC(=O)c1ccc(O)c(OCC)c1

GHS hazard classification: none of the hazards selected for correlation

NN-rank 15: ['CCOc1cc(C(=O)OC(C)C)ccc1O']

EI-MS matched molecular weight (relative abundance: 73.0%) for CCOc1cc(C(=O)OC(C)C)ccc1O

GHS hazard classification: none of the hazards selected for correlation

NN-rank 16: ['CC1COC(c2ccc(O)cc2)O1', 'CCO']

NN-rank 17: ['CC1COC(C)Oc2cc(ccc2O)CO1']

EI-MS matched molecular weight (relative abundance: 73.0%) for CC1COC(C)Oc2cc(ccc2O)CO1

GHS hazard classification: unknown

NN-rank 18: ['CCOc1cc(COCC(C)=O)ccc1O']

EI-MS matched molecular weight (relative abundance: 73.0%) for CCOc1cc(COCC(C)=O)ccc1O

GHS hazard classification: unknown

NN-rank 19: ['CCC=O', 'CCOc1cc(C=O)ccc1O']

EI-MS matched molecular weight (relative abundance: 99.9%) for CCOc1cc(C=O)ccc1O

GHS hazard classification: Irritant

NN-rank 20: ['CCCO', 'CCOc1cc(C)ccc1O', 'O']

EI-MS matched molecular weight (relative abundance: 39.0%) for CCCO

NN/MS matches corresponding with tox GHS classification highlight in Bold font.

GHS hazard classification: Irritant

EI-MS matched molecular weight (relative abundance: 23.0%) for CCOc1cc(C)ccc1O

GHS hazard classification: Irritant

NN-rank 21: ['Cc1ccc(O)c(OC(C)OCC(C)O)c1']

NN-rank 22: ['CC1CO1', 'CCOc1cc(CO)ccc1O']

NN-rank 23: ['C', 'CCOc1cc(C2OCCO2)ccc1O']

NN-rank 24: ['CC1(O)CO1', 'CCOc1cc(C)ccc1O']

EI-MS matched molecular weight (relative abundance: 23.0%) for CCOc1cc(C)ccc1O

GHS hazard classification: Irritant

NN-rank 25: ['CCOc1ccccc1O', 'COC(C)CO']

EI-MS matched molecular weight (relative abundance: 59.0%) for CCOc1ccccc1O

GHS hazard classification: Irritant

NN/MS matches corresponding with tox GHS classification highlight in Bold font.

109. Eucalyptol: CC1(C2CCC(O1)(CC2)C)C

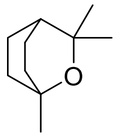

Summary Report of GHS Classification for NN/MS Matches:

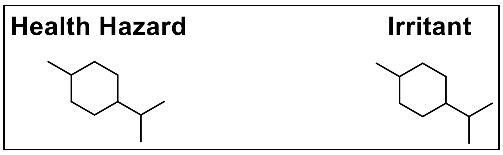

NN-rank 1: ['CC(C)C1CCC(C)(O)CC1']

NN-rank 2: ['CC1(O)CCC2C(C1)C2(C)C']

NN-rank 3: ['CC1(O)CCC2C(C1)C2(C)C']

NN-rank 4: ['CC12C=CC(CC1)C(C)(C)O2']

NN-rank 5: ['CC12C=CC(CC1)C(C)(C)O2']

NN-rank 6: ['CC1CCC(C(C)(C)O)CC1']

NN-rank 7: ['C', 'CC1(C)OC2CCC1CC2']

EI-MS matched molecular weight (relative abundance: 35.53%) for CC1(C)OC2CCC1CC2

GHS hazard classification: none of the hazards selected for correlation

NN-rank 8: ['CC1(O)CCCCC1', 'CCC']

EI-MS matched molecular weight (relative abundance: 99.99%) for CCC

GHS hazard classification: none of the hazards selected for correlation

NN-rank 9: ['CC12CC=C(CC1)C(C)(C)O2']

NN-rank 10: ['CC12CC=C(CC1)C(C)(C)O2']

NN-rank 11: ['CC1(O)C=C2C(CC1)C2(C)C']

NN-rank 12: ['CC1(O)C=C2C(CC1)C2(C)C']

NN-rank 13: ['CC1=CCC(C(C)(C)O)CC1']

NN-rank 14: ['CC1=CCC(C(C)(C)O)CC1']

NN-rank 15: ['CC(C)OC1(C)CCCCC1']

NN-rank 16: ['CC(C)(O)CCC1CC1(C)C']

NN-rank 17: ['CC(C)(O)CCC1CC1(C)C']

NN-rank 18: ['CC1CCC(C)(C)OC1(C)C']

NN-rank 19: ['CC1CCC(C)(C)OC1(C)C']

NN/MS matches corresponding with tox GHS classification highlight in Bold font.

NN-rank 20: ['CCC1(C)CCCC(C)(C)O1']

NN-rank 21: ['CCC1(C)CCCC(C)(C)O1']

NN-rank 22: ['CC1(C)CCCC(C)(O)CC1']

NN-rank 23: ['CC1(C)CCCC(C)(O)CC1']

NN-rank 24: ['C', 'CC(C)(O)C1CCCCC1']

NN-rank 25: ['CC1CCC(C(C)C)CC1', 'O']

EI-MS matched molecular weight (relative abundance: 35.53%) for CC1CCC(C(C)C)CC1

GHS hazard classification: Health Hazard, Irritant

NN/MS matches corresponding with tox GHS classification highlight in Bold font.

110. trans-2-Hexenol: CCC/C=C/CO

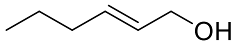

Summary Report of GHS Classification for NN/MS Matches:

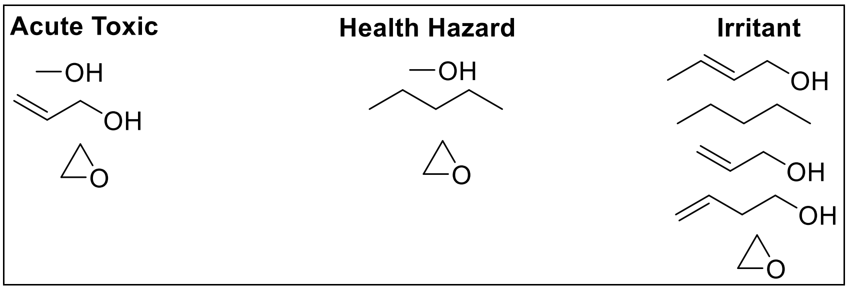

NN-rank 1: ['C', 'CC/C=C/CO']

NN-rank 2: ['CCC/C=C/C=O']

NN-rank 3: ['CCC=CCCO']

NN-rank 4: ['CCCCCCO']

NN-rank 5: ['CCCC1=CCO1']

NN-rank 6: ['CCC/C=C1/CO1']

NN-rank 7: ['C/C=C/CO', 'CC']

EI-MS matched molecular weight (relative abundance: 10.19%) for C/C=C/CO

GHS hazard classification: Irritant

EI-MS matched molecular weight (relative abundance: 15.99%) for CC

GHS hazard classification: none of the hazards selected for correlation

NN-rank 8: ['CCCC1CCO1']

NN-rank 9: ['CCC=C=CCO']

NN-rank 10: ['C=CCCC', 'CO']

EI-MS matched molecular weight (relative abundance: 9.69%) for CO

GHS hazard classification: Acute Toxic, Health Hazard

NN-rank 11: ['C=CCCCC', 'O']

NN-rank 12: ['CCCCC=CO']

NN-rank 13: ['C=C=CCCC', 'O']

NN-rank 14: ['C/C=C(\\O)CCC']

NN-rank 15: ['CCCCC', 'CO']

EI-MS matched molecular weight (relative abundance: 10.19%) for CCCCC

GHS hazard classification: Health Hazard, Irritant

EI-MS matched molecular weight (relative abundance: 9.69%) for CO

NN/MS matches corresponding with tox GHS classification highlight in Bold font.

GHS hazard classification: Acute Toxic, Health Hazard

NN-rank 16: ['C/C=C/CCC', 'O']

NN-rank 17: ['CCCC=C=CO']

NN-rank 18: ['CCCCC1CO1']

NN-rank 19: ['CCCC', 'CCO']

EI-MS matched molecular weight (relative abundance: 99.99%) for CCCC

GHS hazard classification: none of the hazards selected for correlation

NN-rank 20: ['C=CCCO', 'CC']

EI-MS matched molecular weight (relative abundance: 10.19%) for C=CCCO

GHS hazard classification: Irritant

EI-MS matched molecular weight (relative abundance: 15.99%) for CC

GHS hazard classification: none of the hazards selected for correlation

NN-rank 21: ['C=CCO', 'CCC']

EI-MS matched molecular weight (relative abundance: 99.99%) for C=CCO

GHS hazard classification: Acute Toxic, Irritant

EI-MS matched molecular weight (relative abundance: 14.99%) for CCC

GHS hazard classification: none of the hazards selected for correlation

NN-rank 22: ['CCC/C=C(/C)O']

NN-rank 23: ['CC=CCC', 'CO']

EI-MS matched molecular weight (relative abundance: 9.69%) for CO

GHS hazard classification: Acute Toxic, Health Hazard

NN-rank 24: ['CCC=CC1CO1']

NN-rank 25: ['C1CO1', 'CCCC']

EI-MS matched molecular weight (relative abundance: 14.99%) for C1CO1

GHS hazard classification: Acute Toxic, Health Hazard, Irritant

EI-MS matched molecular weight (relative abundance: 99.99%) for CCCC

GHS hazard classification: none of the hazards selected for correlation

NN/MS matches corresponding with tox GHS classification highlight in Bold font.

111. Furaneol: CC1C(=O)C(=C(O1)C)O

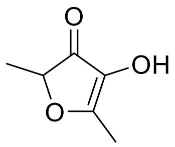

NN-rank 1: ['CC1=C(O)C(O)C(C)O1']

NN-rank 2: ['CC1=C(O)CC(C)O1', 'O']

NN-rank 3: ['CCOC(C)=C(O)CO']

NN-rank 4: ['CCC(=O)C(O)=C(C)O']

NN-rank 5: ['CCOC(C)=C(O)C=O']

NN-rank 6: ['CC1OC(C)C(O)C1=O']

NN-rank 7: ['C', 'CC1=C(O)C(=O)CO1']

EI-MS matched molecular weight (relative abundance: 7.79%) for C

GHS hazard classification: none of the hazards selected for correlation

NN-rank 8: ['Cc1oc(C)c(O)c1O']

NN-rank 9: ['CC=C(O)C(=O)C(C)O']

NN-rank 10: ['CC1=C(O)C2OCC2O1']

NN-rank 11: ['CC', 'CC(O)=C(O)CO']

EI-MS matched molecular weight (relative abundance: 15.69%) for CC

GHS hazard classification: none of the hazards selected for correlation

NN-rank 12: ['CC1OC2=C(O)C1OC2']

NN-rank 13: ['CC(=CO)OC(C)CO']

NN-rank 14: ['CC=C(O)C(O)=C(C)O']

NN-rank 15: ['CC1OC2=C(O)C1(O)C2']

NN-rank 16: ['Cc1cc(O)c(C)o1', 'O']

NN-rank 17: ['CC1=C2OC2(O)C(C)O1']

NN-rank 18: ['CCC(O)C(=O)C(C)O']

NN-rank 19: ['CC1=C(O)COCCO1']

NN-rank 20: ['CC(=CO)OC(C)C=O']

NN-rank 21: ['CC1=C(O)CC(CO)O1']

NN-rank 22: ['CC1=C2OC2C(C)O1', 'O']

NN-rank 23: ['C', 'COC(C)=C(O)CO']

EI-MS matched molecular weight (relative abundance: 7.79%) for C

GHS hazard classification: none of the hazards selected for correlation

NN/MS matches corresponding with tox GHS classification highlight in Bold font.

NN-rank 24: ['CC', 'CC(O)=C(O)C=O']

EI-MS matched molecular weight (relative abundance: 15.69%) for CC

GHS hazard classification: none of the hazards selected for correlation

NN-rank 25: ['CCOC(C)=C(C)O', 'O']

NN/MS matches corresponding with tox GHS classification highlight in Bold font.

112. Furfural: C1=COC(=C1)C=O

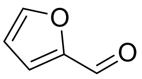

Summary Report of GHS Classification for NN/MS Matches:

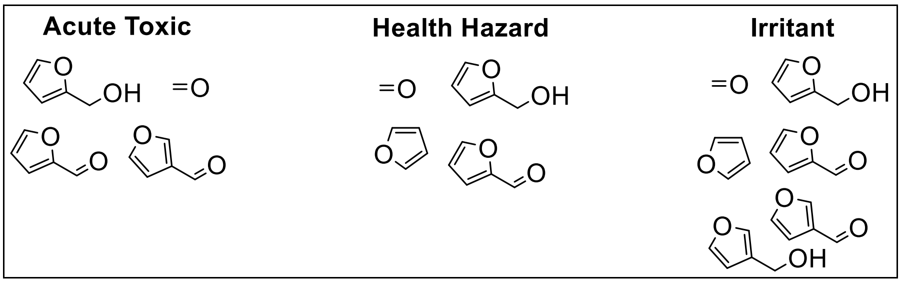

NN-rank 1: ['OCc1ccco1']

EI-MS matched molecular weight (relative abundance: 5.77%) for OCc1ccco1

GHS hazard classification: Acute Toxic, Health Hazard, Irritant

NN-rank 2: ['C=O', 'c1ccoc1']

EI-MS matched molecular weight (relative abundance: 20.18%) for C=O

GHS hazard classification: Acute Toxic, Health Hazard, Irritant

EI-MS matched molecular weight (relative abundance: 11.4%) for c1ccoc1

GHS hazard classification: Health Hazard, Irritant

NN-rank 3: ['CO', 'c1ccoc1']

EI-MS matched molecular weight (relative abundance: 11.4%) for c1ccoc1

GHS hazard classification: Health Hazard, Irritant

NN-rank 4: ['O=C1c2ccc1o2']

NN-rank 5: ['Cc1ccco1', 'O']

NN-rank 6: ['OC1c2ccc1o2']

EI-MS matched molecular weight (relative abundance: 99.15%) for OC1c2ccc1o2

GHS hazard classification: unknown

NN-rank 7: ['c1cc2oc1CO2']

EI-MS matched molecular weight (relative abundance: 99.15%) for c1cc2oc1CO2

GHS hazard classification: unknown

NN-rank 8: ['O=c1c2ccoc12']

NN-rank 9: ['O=Cc1ccco1']

EI-MS matched molecular weight (relative abundance: 99.15%) for O=Cc1ccco1

GHS hazard classification: Acute Toxic, Health Hazard, Irritant

NN/MS matches corresponding with tox GHS classification highlight in Bold font.

NN-rank 10: ['OC1c2ccoc21']

EI-MS matched molecular weight (relative abundance: 99.15%) for OC1c2ccoc21

GHS hazard classification: none of the hazards selected for correlation

NN-rank 11: ['c1cc2c(o1)CO2']

EI-MS matched molecular weight (relative abundance: 99.15%) for c1cc2c(o1)CO2

GHS hazard classification: none of the hazards selected for correlation

NN-rank 12: ['O=Cc1oc2cc1-2']

NN-rank 13: ['O=Cc1ccco1']

EI-MS matched molecular weight (relative abundance: 99.15%) for O=Cc1ccco1

GHS hazard classification: Acute Toxic, Health Hazard, Irritant

NN-rank 14: ['OCc1ccco1']

EI-MS matched molecular weight (relative abundance: 5.77%) for OCc1ccco1

GHS hazard classification: Acute Toxic, Health Hazard, Irritant

NN-rank 15: ['c1cc2oc1C2', 'O']

NN-rank 16: ['C=O', 'c1ccoc1']

EI-MS matched molecular weight (relative abundance: 20.18%) for C=O

GHS hazard classification: Acute Toxic, Health Hazard, Irritant

EI-MS matched molecular weight (relative abundance: 11.4%) for c1ccoc1

GHS hazard classification: Health Hazard, Irritant

NN-rank 17: ['O=Cc1ccoc1']

EI-MS matched molecular weight (relative abundance: 99.15%) for O=Cc1ccoc1

GHS hazard classification: Acute Toxic, Irritant

NN-rank 18: ['OCc1ccco1']

EI-MS matched molecular weight (relative abundance: 5.77%) for OCc1ccco1

GHS hazard classification: Acute Toxic, Health Hazard, Irritant

NN-rank 19: ['Cc1ccc(O)o1']

EI-MS matched molecular weight (relative abundance: 5.77%) for Cc1ccc(O)o1

GHS hazard classification: none of the hazards selected for correlation

NN-rank 20: ['c1cc2c(o1)C2', 'O']

NN-rank 21: ['COc1ccco1']

EI-MS matched molecular weight (relative abundance: 5.77%) for COc1ccco1

GHS hazard classification: none of the hazards selected for correlation

NN-rank 22: ['C', 'O', 'c1ccoc1']

EI-MS matched molecular weight (relative abundance: 11.4%) for c1ccoc1

GHS hazard classification: Health Hazard, Irritant

NN/MS matches corresponding with tox GHS classification highlight in Bold font.

NN-rank 23: ['OCc1ccoc1']

EI-MS matched molecular weight (relative abundance: 5.77%) for OCc1ccoc1

GHS hazard classification: Irritant

NN-rank 24: ['COc1ccco1']

EI-MS matched molecular weight (relative abundance: 5.77%) for COc1ccco1

GHS hazard classification: none of the hazards selected for correlation

NN-rank 25: ['Cc1occc1O']

EI-MS matched molecular weight (relative abundance: 5.77%) for Cc1occc1O

GHS hazard classification: none of the hazards selected for correlation

NN/MS matches corresponding with tox GHS classification highlight in Bold font.

113. Furfuryl Alcohol: C1=COC(=C1)CO

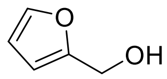

Summary Report of GHS Classification for NN/MS Matches:

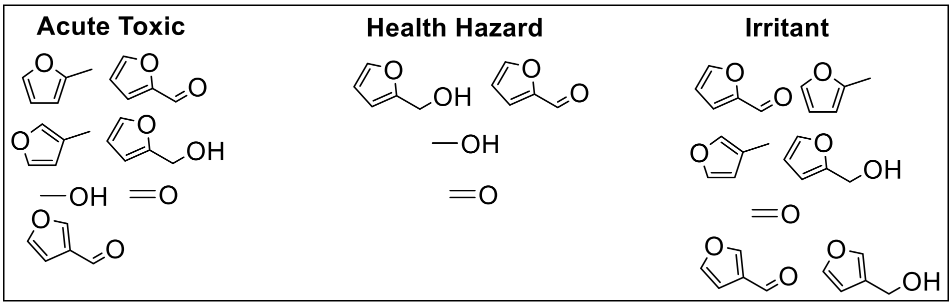

NN-rank 1: ['O=Cc1ccco1']

EI-MS matched molecular weight (relative abundance: 7.01%) for O=Cc1ccco1

GHS hazard classification: Acute Toxic, Health Hazard, Irritant

NN-rank 2: ['Cc1ccco1', 'O']

EI-MS matched molecular weight (relative abundance: 56.25%) for Cc1ccco1

GHS hazard classification: Acute Toxic, Irritant

NN-rank 3: ['CO', 'c1ccoc1']

EI-MS matched molecular weight (relative abundance: 9.31%) for CO

GHS hazard classification: Acute Toxic, Health Hazard

NN-rank 4: ['C=O', 'c1ccoc1']

EI-MS matched molecular weight (relative abundance: 16.82%) for C=O

GHS hazard classification: Acute Toxic, Health Hazard, Irritant

NN-rank 5: ['c1cc2c(o1)C2', 'O']

NN-rank 6: ['c1cc2oc1C2', 'O']

NN-rank 7: ['C', 'O', 'c1ccoc1']

NN-rank 8: ['O=Cc1ccco1']

EI-MS matched molecular weight (relative abundance: 7.01%) for O=Cc1ccco1

GHS hazard classification: Acute Toxic, Health Hazard, Irritant

NN-rank 9: ['O=c1c2ccoc12']

NN-rank 10: ['OC1c2ccoc21']

EI-MS matched molecular weight (relative abundance: 7.01%) for OC1c2ccoc21

GHS hazard classification: none of the hazards selected for correlation

NN/MS matches corresponding with tox GHS classification highlight in Bold font.

NN-rank 11: ['O=C1c2ccc1o2']

NN-rank 12: ['OC1c2ccc1o2']

EI-MS matched molecular weight (relative abundance: 7.01%) for OC1c2ccc1o2

GHS hazard classification: unknown

NN-rank 13: ['O=Cc1ccco1']

EI-MS matched molecular weight (relative abundance: 7.01%) for O=Cc1ccco1

GHS hazard classification: Acute Toxic, Health Hazard, Irritant

NN-rank 14: ['OCc1ccco1']

EI-MS matched molecular weight (relative abundance: 51.35%) for OCc1ccco1

GHS hazard classification: Acute Toxic, Health Hazard, Irritant

NN-rank 15: ['c1oc2cc1OC2']

EI-MS matched molecular weight (relative abundance: 7.01%) for c1oc2cc1OC2

GHS hazard classification: unknown

NN-rank 16: ['O=Cc1ccco1']

EI-MS matched molecular weight (relative abundance: 7.01%) for O=Cc1ccco1

GHS hazard classification: Acute Toxic, Health Hazard, Irritant

NN-rank 17: ['OCc1ccco1']

EI-MS matched molecular weight (relative abundance: 51.35%) for OCc1ccco1

GHS hazard classification: Acute Toxic, Health Hazard, Irritant

NN-rank 18: ['Cc1ccco1', 'O']

EI-MS matched molecular weight (relative abundance: 56.25%) for Cc1ccco1

GHS hazard classification: Acute Toxic, Irritant

NN-rank 19: ['Cc1cc(O)co1']

EI-MS matched molecular weight (relative abundance: 51.35%) for Cc1cc(O)co1

GHS hazard classification: none of the hazards selected for correlation

NN-rank 20: ['OCc1ccco1']

EI-MS matched molecular weight (relative abundance: 51.35%) for OCc1ccco1

GHS hazard classification: Acute Toxic, Health Hazard, Irritant

NN-rank 21: ['Cc1ccco1', 'O']

EI-MS matched molecular weight (relative abundance: 56.25%) for Cc1ccco1

GHS hazard classification: Acute Toxic, Irritant

NN-rank 22: ['Cc1ccco1', 'O']

EI-MS matched molecular weight (relative abundance: 56.25%) for Cc1ccco1

GHS hazard classification: Acute Toxic, Irritant

NN-rank 23: ['Cc1ccoc1', 'O']

NN/MS matches corresponding with tox GHS classification highlight in Bold font.

EI-MS matched molecular weight (relative abundance: 56.25%) for Cc1ccoc1

GHS hazard classification: Acute Toxic, Irritant

NN-rank 24: ['O=Cc1ccoc1']

EI-MS matched molecular weight (relative abundance: 7.01%) for O=Cc1ccoc1

GHS hazard classification: Acute Toxic, Irritant

NN-rank 25: ['OCc1ccoc1']

EI-MS matched molecular weight (relative abundance: 51.35%) for OCc1ccoc1

GHS hazard classification: Irritant

NN/MS matches corresponding with tox GHS classification highlight in Bold font.

114. gamma-Decalactone: CCCCCCC1CCC(=O)O1

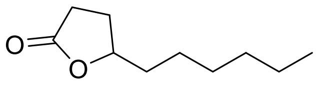

NN-rank 1: ['CCCCCCC(O)CCCO']

NN-rank 2: ['C', 'CCCCCC1CCC(=O)O1']

NN-rank 3: ['CCCCCCCCCC(=O)O']

NN-rank 4: ['CCCCCCC(O)CCC=O']

NN-rank 5: ['CCCCCCC1CCC(O)O1']

NN-rank 6: ['CCCCCCCCCC(=O)O']

NN-rank 7: ['CCCCCCC(=O)CCCO']

NN-rank 8: ['OC1CCCCCCOCCC1']

NN-rank 9: ['CC', 'CCCCC1CCC(=O)O1']

EI-MS matched molecular weight (relative abundance: 13.81%) for CC

GHS hazard classification: none of the hazards selected for correlation

NN-rank 10: ['CCCCCC(O)CCCOC']

NN-rank 11: ['CCCCCCC1C=CC(=O)O1']

NN-rank 12: ['CCCCCCC1=CCC(=O)O1']

NN-rank 13: ['CC1CCCCC(O)CCCO1']

NN-rank 14: ['CCCCCCC=CCC(=O)O']

NN-rank 15: ['CCCCCC1CCC(OC)O1']

NN-rank 16: ['O=C1CCCCCCCCCO1']

NN-rank 17: ['C1CCCC2CCC(OCC1)O2']

NN-rank 18: ['CCCCCCC1CCCO1', 'O']

NN-rank 19: ['CCC', 'CCCC1CCC(=O)O1']

EI-MS matched molecular weight (relative abundance: 8.71%) for CCC

GHS hazard classification: none of the hazards selected for correlation

NN-rank 20: ['CCCCCCCCC(=O)OC']

NN-rank 21: ['CCCCCCC(=O)CCC=O']

NN-rank 22: ['CCCCCCC=CCC(=O)O']

NN-rank 23: ['CCCCCC1OCCCC1O']

NN-rank 24: ['CCCCCCCCCCO', 'O']

NN-rank 25: ['CC1CCCCCCCC(=O)O1']

NN/MS matches corresponding with tox GHS classification highlight in Bold font.

115. gamma-Dodecalactone: CCCCCCCCC1CCC(=O)O1

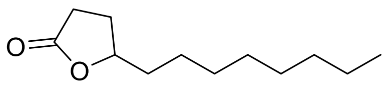

NN-rank 1: ['CCCCCCCCC(O)CCCO']

NN-rank 2: ['C', 'CCCCCCCC1CCC(=O)O1']

NN-rank 3: ['CCCCCCCCCCCC(=O)O']

NN-rank 4: ['CCCCCCCCC(O)CCC=O']

NN-rank 5: ['CCCCCCCCCCCC(=O)O']

NN-rank 6: ['CCCCCCCCC1CCC(O)O1']

NN-rank 7: ['CCCCCCCCC(=O)CCCO']

NN-rank 8: ['OC1CCCCCCCCOCCC1']

NN-rank 9: ['CC', 'CCCCCCC1CCC(=O)O1']

EI-MS matched molecular weight (relative abundance: 43.0%) for CC

GHS hazard classification: none of the hazards selected for correlation

NN-rank 10: ['CCCCCCCC(O)CCCOC']

NN-rank 11: ['CCCCCCCCCCC(=O)OC']

NN-rank 12: ['CCCCCCCCC1C=CC(=O)O1']

NN-rank 13: ['CCCCCCCCC1=CCC(=O)O1']

NN-rank 14: ['O=C1CCCCCCCCCCCO1']

NN-rank 15: ['CCCCCCCCC=CCC(=O)O']

NN-rank 16: ['CC1CCCCCCC(O)CCCO1']

NN-rank 17: ['CCCCCCCC1CCC(OC)O1']

NN-rank 18: ['C1CCCCC2CCC(OCCC1)O2']

NN-rank 19: ['CCCCCCCCC=CCC(=O)O']

NN-rank 20: ['CCCCCCCCC1CCC(=O)O1']

NN-rank 21: ['CCCCCCCCC1CCCO1', 'O']

NN-rank 22: ['CCCCCCCCC(=O)CCC=O']

NN-rank 23: ['CCCCCCCC1OCCCC1O']

NN-rank 24: ['CCCCCCCCCCCCO', 'O']

NN-rank 25: ['CC1CCCCCCCCCC(=O)O1']

NN/MS matches corresponding with tox GHS classification highlight in Bold font.

116. gamma-Hexalactone: CCC1CCC(=O)O1

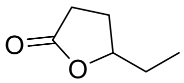

NN-rank 1: ['CCC(O)CCCO']

NN-rank 2: ['CCC(=O)CCCO']

NN-rank 3: ['CCC(O)CCC=O']

NN-rank 4: ['OC1CCCOCC1']

NN-rank 5: ['CCCCCC(=O)O']

NN-rank 6: ['CCC(=O)CCC=O']

NN-rank 7: ['CCC1CCC(O)O1']

NN-rank 8: ['CCCCCCO', 'O']

NN-rank 9: ['CC1OCCCC1O']

NN-rank 10: ['CCC(O)CC1CO1']

NN-rank 11: ['C', 'CC1CCC(=O)O1']

NN-rank 12: ['CCC1C=CC(=O)O1']

NN-rank 13: ['CCCCCC(=O)O']

NN-rank 14: ['O=C1CCCOCC1']

NN-rank 15: ['COCCCC(C)O']

NN-rank 16: ['C1CC2CCC(O1)O2']

NN-rank 17: ['CC1OCCCC1=O']

NN-rank 18: ['O=C1OC2CCC1C2']

NN-rank 19: ['CCC1CCCO1', 'O']

NN-rank 20: ['CCC(=O)CC1CO1']

NN-rank 21: ['C', 'OC1CCCOC1']

NN-rank 22: ['CC1OC2CCC1O2']

NN-rank 23: ['C1CCCOCC1', 'O']

NN-rank 24: ['CCCCCC=O', 'O']

NN-rank 25: ['CCC1CC2OC2O1']

117. gamma-Nonalactone: CCCCCC1CCC(=O)O1

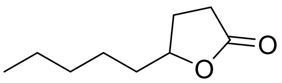

NN-rank 1: ['CCCCCC(O)CCCO']

NN-rank 2: ['C', 'CCCCC1CCC(=O)O1']

NN-rank 3: ['CCCCCCCCC(=O)O']

NN-rank 4: ['CCCCCC(O)CCC=O']

NN-rank 5: ['CCCCCC1CCC(O)O1']

NN-rank 6: ['CCCCCCCCC(=O)O']

NN-rank 7: ['CCCCCC(=O)CCCO']

NN-rank 8: ['CC', 'CCCC1CCC(=O)O1']

EI-MS matched molecular weight (relative abundance: 19.69%) for CC

GHS hazard classification: none of the hazards selected for correlation

NN-rank 9: ['OC1CCCCCOCCC1']

NN-rank 10: ['CCCCCC1C=CC(=O)O1']

NN-rank 11: ['CCCCC(O)CCCOC']

NN-rank 12: ['CCCCCC1=CCC(=O)O1']

NN-rank 13: ['CC1CCCC(O)CCCO1']

NN-rank 14: ['CCCCCC=CCC(=O)O']

NN-rank 15: ['CCCCC1CCC(OC)O1']

NN-rank 16: ['CCCCCC1CCCO1', 'O']

NN-rank 17: ['C1CCOC2CCC(CC1)O2']

NN-rank 18: ['CC1CCC(=O)O1', 'CCCC']

EI-MS matched molecular weight (relative abundance: 8.49%) for CC1CCC(=O)O1

GHS hazard classification: none of the hazards selected for correlation

EI-MS matched molecular weight (relative abundance: 5.89%) for CCCC

GHS hazard classification: none of the hazards selected for correlation

NN-rank 19: ['O=C1CCCCCCCCO1']

NN-rank 20: ['CCCCCC(=O)CCC=O']

NN-rank 21: ['CCCCC1OCCCC1O']

NN-rank 22: ['CCCCCC=CCC(=O)O']

NN-rank 23: ['CCCCCC1CCC(=O)O1']

NN-rank 24: ['CCCCCCCC(=O)OC']

NN/MS matches corresponding with tox GHS classification highlight in Bold font.

NN-rank 25: ['CCCCCCCCCCO', 'O']

NN/MS matches corresponding with tox GHS classification highlight in Bold font.

118. gamma-Octalactone: CCCCC1CCC(=O)O1

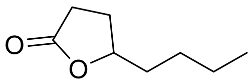

Summary Report of GHS Classification for NN/MS Matches:

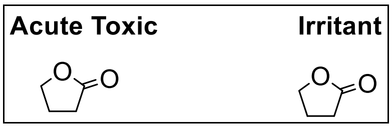

NN-rank 1: ['CCCCC(O)CCCO']

NN-rank 2: ['C', 'CCCC1CCC(=O)O1']

NN-rank 3: ['CCCCCCCC(=O)O']

NN-rank 4: ['CCCCC(O)CCC=O']

NN-rank 5: ['CCCCC1CCC(O)O1']

NN-rank 6: ['CCCCCCCC(=O)O']

NN-rank 7: ['CCCCC(=O)CCCO']

NN-rank 8: ['OC1CCCCOCCC1']

NN-rank 9: ['CC', 'CCC1CCC(=O)O1']

EI-MS matched molecular weight (relative abundance: 22.0%) for CC

GHS hazard classification: none of the hazards selected for correlation

NN-rank 10: ['CCCCC1C=CC(=O)O1']

NN-rank 11: ['CC1CCC(O)CCCO1']

NN-rank 12: ['CCCCC1=CCC(=O)O1']

NN-rank 13: ['CCCC1OCCCC1O']

NN-rank 14: ['CC1CCC(=O)O1', 'CCC']

NN-rank 15: ['CCCCC=CCC(=O)O']

NN-rank 16: ['CCCCC(=O)CCC=O']

NN-rank 17: ['CCCCC1CCCO1', 'O']

NN-rank 18: ['C1CCC2CCC(OC1)O2']

NN-rank 19: ['CCCC(O)CCCOC']

NN-rank 20: ['CCCC1CCC(OC)O1']

NN-rank 21: ['O=C1CCCCCCCO1']

NN-rank 22: ['CCCCC=CCC(=O)O']

NN-rank 23: ['CC1CCC2CCC(O1)O2']

NN-rank 24: ['CCCCCCCCO', 'O']

NN/MS matches corresponding with tox GHS classification highlight in Bold font.

NN-rank 25: ['CCCC', 'O=C1CCCO1']

EI-MS matched molecular weight (relative abundance: 12.0%) for CCCC

GHS hazard classification: none of the hazards selected for correlation

EI-MS matched molecular weight (relative abundance: 99.9%) for O=C1CCCO1

GHS hazard classification: Acute Toxic, Irritant

NN/MS matches corresponding with tox GHS classification highlight in Bold font.

119. gamma-Terpinene: CC1=CCC(=CC1)C(C)C

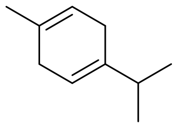

NN-rank 1: ['CC1C=C=C(C(C)C)CC1']

NN-rank 2: ['CC1=CCC(C(C)C)=C=C1']

NN-rank 3: ['C', 'CC(C)C1=C=CCCC1']

EI-MS matched molecular weight (relative abundance: 30.39%) for CC(C)C1=C=CCCC1

GHS hazard classification: none of the hazards selected for correlation

NN-rank 4: ['CC1C=C=C(C(C)C)C=C1']

NN-rank 5: ['CC(C)C1=CC2C(C)CC12']

NN-rank 6: ['CC1CC2C1=CC2C(C)C']

NN-rank 7: ['CC(C)C1CC2C(C)CC12']

NN-rank 8: ['CC1C=CC(C(C)C)=CC1']

NN-rank 9: ['C=C1C=C=C(C(C)C)CC1']

NN-rank 10: ['CC(C)C1C=CC2(C)CC12']

NN-rank 11: ['CC1=CC2C(C(C)C)=CC12']

NN-rank 12: ['CC1=CC2C1CC2C(C)C']

NN-rank 13: ['CC1C=C=CCC1', 'CCC']

EI-MS matched molecular weight (relative abundance: 99.99%) for CC1C=C=CCC1

GHS hazard classification: none of the hazards selected for correlation

EI-MS matched molecular weight (relative abundance: 17.59%) for CCC

GHS hazard classification: none of the hazards selected for correlation

NN-rank 14: ['CC1=C=CC(C(C)C)=CC1']

NN-rank 15: ['CC1=CC2C1=CC2C(C)C']

NN-rank 16: ['CC1=C=CC(C(C)C)C=C1']

NN-rank 17: ['CC(C)C1=C=C2C(C)CC12']

NN-rank 18: ['CC1CC2C=CC12', 'CCC']

EI-MS matched molecular weight (relative abundance: 99.99%) for CC1CC2C=CC12

GHS hazard classification: none of the hazards selected for correlation

EI-MS matched molecular weight (relative abundance: 17.59%) for CCC

GHS hazard classification: none of the hazards selected for correlation

NN-rank 19: ['CC1=C=CC(C(C)C)CC1']

NN/MS matches corresponding with tox GHS classification highlight in Bold font.

NN-rank 20: ['CC(C)C1=CCC2(C)CC12']

NN-rank 21: ['Cc1ccc(C(C)C)cc1']

NN-rank 22: ['CC1CC2CCC12', 'CCC']

EI-MS matched molecular weight (relative abundance: 17.59%) for CCC

GHS hazard classification: none of the hazards selected for correlation

NN-rank 23: ['CC(C)C1=CC2C1=CC2C']

NN-rank 24: ['CC1CC=C(C(C)C)CC1']

NN-rank 25: ['CC(C)C1=C=CC2(C)CC12']

NN/MS matches corresponding with tox GHS classification highlight in Bold font.

120. gamma-Undecalactone: CCCCCCCC1CCC(=O)O1

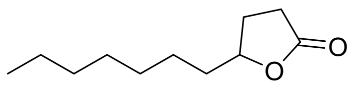

NN-rank 1: ['CCCCCCCC(O)CCCO']

NN-rank 2: ['C', 'CCCCCCC1CCC(=O)O1']

NN-rank 3: ['CCCCCCCCCCC(=O)O']

NN-rank 4: ['CCCCCCCC(O)CCC=O']

NN-rank 5: ['CCCCCCCC1CCC(O)O1']

NN-rank 6: ['CCCCCCCCCCC(=O)O']

NN-rank 7: ['CCCCCCCC(=O)CCCO']

NN-rank 8: ['OC1CCCCCCCOCCC1']

NN-rank 9: ['CC', 'CCCCCC1CCC(=O)O1']

EI-MS matched molecular weight (relative abundance: 16.51%) for CC

GHS hazard classification: none of the hazards selected for correlation

NN-rank 10: ['CCCCCCC(O)CCCOC']

NN-rank 11: ['CCCCCCCC1C=CC(=O)O1']

NN-rank 12: ['CCCCCCCC1=CCC(=O)O1']

NN-rank 13: ['CCCCCCCCCC(=O)OC']

NN-rank 14: ['CC1CCCCCC(O)CCCO1']

NN-rank 15: ['CCCCCCCC=CCC(=O)O']

NN-rank 16: ['O=C1CCCCCCCCCCO1']

NN-rank 17: ['CCCCCCC1CCC(OC)O1']

NN-rank 18: ['C1CCCOC2CCC(CCC1)O2']

NN-rank 19: ['CCCCCCCC1CCCO1', 'O']

NN-rank 20: ['CCCCCCCC=CCC(=O)O']

NN-rank 21: ['CCCCCCCC(=O)CCC=O']

NN-rank 22: ['CCCCCCCC1CCC(=O)O1']

NN-rank 23: ['CCCCCCC1OCCCC1O']

NN-rank 24: ['CCCCCCCCCCCO', 'O']

NN-rank 25: ['CC1CCCCCCCCC(=O)O1']

121. gamma-Valerolactone: CC1CCC(=O)O1

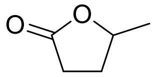

Summary Report of GHS Classification for NN/MS Matches:

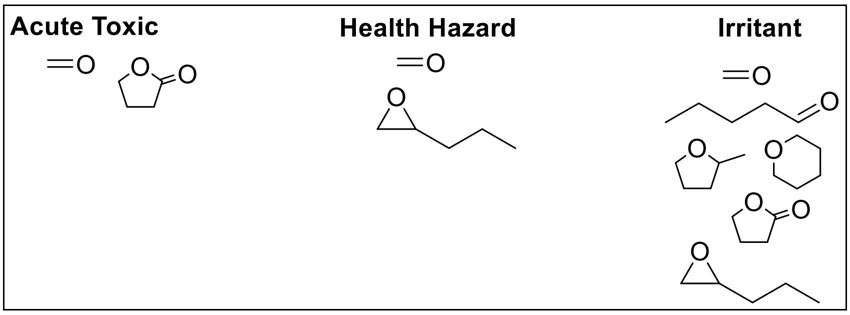

NN-rank 1: ['CC(O)CCCO']

NN-rank 2: ['CC(O)CCC=O']

NN-rank 3: ['CCCCC(=O)O']

NN-rank 4: ['CCCCCO', 'O']

NN-rank 5: ['CC1CCC(O)O1']

NN-rank 6: ['CC(O)CC1CO1']

NN-rank 7: ['CC(=O)CCCO']

NN-rank 8: ['CC1C=CC(=O)O1']

NN-rank 9: ['OC1CCCOC1']

NN-rank 10: ['CC(=O)CCC=O']

NN-rank 11: ['CCCCC(=O)O']

NN-rank 12: ['CC1(O)CCCO1']

NN-rank 13: ['CC1CCCO1', 'O']

EI-MS matched molecular weight (relative abundance: 46.8%) for CC1CCCO1

GHS hazard classification: Irritant

NN-rank 14: ['CC(O)CC=CO']

NN-rank 15: ['C', 'O=C1CCCO1']

EI-MS matched molecular weight (relative abundance: 46.8%) for O=C1CCCO1

GHS hazard classification: Acute Toxic, Irritant

NN-rank 16: ['CC(O)CC=C=O']

NN-rank 17: ['CCCCC=O', 'O']

EI-MS matched molecular weight (relative abundance: 46.8%) for CCCCC=O

NN/MS matches corresponding with tox GHS classification highlight in Bold font.

GHS hazard classification: Irritant

NN-rank 18: ['C=O', 'CCC(C)O']

EI-MS matched molecular weight (relative abundance: 25.8%) for C=O

GHS hazard classification: Acute Toxic, Health Hazard, Irritant

NN-rank 19: ['CC(O)C1CCO1']

NN-rank 20: ['CC1CC2OC2O1']

NN-rank 21: ['CC1CCC(O)O1']

NN-rank 22: ['CC1CCCO1', 'O']

EI-MS matched molecular weight (relative abundance: 46.8%) for CC1CCCO1

GHS hazard classification: Irritant

NN-rank 23: ['CC1CC1C(=O)O']

NN-rank 24: ['CCCC1CO1', 'O']

EI-MS matched molecular weight (relative abundance: 46.8%) for CCCC1CO1

GHS hazard classification: Health Hazard, Irritant

NN-rank 25: ['C1CC2OCC1O2']

NN/MS matches corresponding with tox GHS classification highlight in Bold font.

122. trans-2-Hexenoic acid: CCC/C=C/C(=O)O

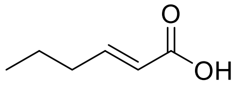

Summary Report of GHS Classification for NN/MS Matches:

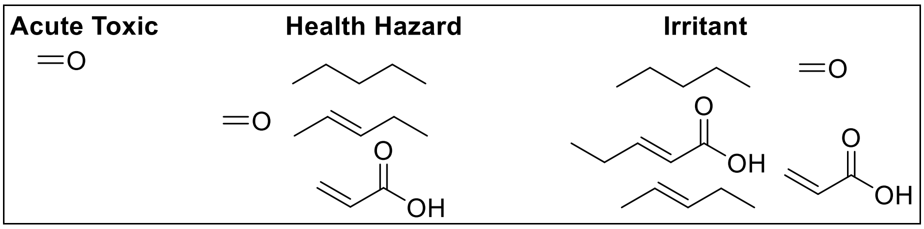

NN-rank 1: ['CCCCCC(=O)O']

NN-rank 2: ['CCC=CCC(=O)O']

NN-rank 3: ['CCCCC', 'O=CO']

> **EI-MS matched molecular weight (relative abundance: 7.4%) for CCCCC**
>
> **GHS hazard classification: Health Hazard, Irritant**
>
> **EI-MS matched molecular weight (relative abundance: 16.7%) for O=CO**
>
> **GHS hazard classification: none of the hazards selected for correlation**

NN-rank 4: ['CCC', 'CCC(=O)O']

> **EI-MS matched molecular weight (relative abundance: 18.7%) for CCC**
>
> **GHS hazard classification: none of the hazards selected for correlation**
>
> **EI-MS matched molecular weight (relative abundance: 99.99%) for CCC(=O)O**
>
> **GHS hazard classification: none of the hazards selected for correlation**

NN-rank 5: ['O=C(O)C1CCCC1']

NN-rank 6: ['O=C(O)CC1CCC1']

NN-rank 7: ['C=CCC(=O)O', 'CC']

> **EI-MS matched molecular weight (relative abundance: 31.0%) for CC**
>
> **GHS hazard classification: none of the hazards selected for correlation**

NN-rank 8: ['CCCC(C)C(=O)O']

NN-rank 9: ['C', 'CC/C=C/C(=O)O']

> **EI-MS matched molecular weight (relative abundance: 30.2%) for CC/C=C/C(=O)O**
>
> **GHS hazard classification: Irritant**

NN-rank 10: ['CCC(C)CC(=O)O']

NN-rank 11: ['CCCC1CC1=O', 'O']

NN-rank 12: ['C/C=C/C(=O)O', 'CC']

NN/MS matches corresponding with tox GHS classification highlight in Bold font.

EI-MS matched molecular weight (relative abundance: 31.0%) for CC

GHS hazard classification: none of the hazards selected for correlation

NN-rank 13: ['CC=CCC', 'O=CO']

EI-MS matched molecular weight (relative abundance: 17.4%) for CC=CCC

GHS hazard classification: Health Hazard, Irritant

EI-MS matched molecular weight (relative abundance: 16.7%) for O=CO

GHS hazard classification: none of the hazards selected for correlation

NN-rank 14: ['CC1CCC1', 'O=CO']

EI-MS matched molecular weight (relative abundance: 17.4%) for CC1CCC1

GHS hazard classification: none of the hazards selected for correlation

EI-MS matched molecular weight (relative abundance: 16.7%) for O=CO

GHS hazard classification: none of the hazards selected for correlation

NN-rank 15: ['O=C(O)C1C2CCC21']

NN-rank 16: ['C1CCCC1', 'O=CO']

EI-MS matched molecular weight (relative abundance: 17.4%) for C1CCCC1

GHS hazard classification: none of the hazards selected for correlation

EI-MS matched molecular weight (relative abundance: 16.7%) for O=CO

GHS hazard classification: none of the hazards selected for correlation

NN-rank 17: ['CC/C=C(\\C)C(=O)O']

NN-rank 18: ['C=CCCC', 'O=CO']

EI-MS matched molecular weight (relative abundance: 17.4%) for C=CCCC

GHS hazard classification: none of the hazards selected for correlation

EI-MS matched molecular weight (relative abundance: 16.7%) for O=CO

GHS hazard classification: none of the hazards selected for correlation

NN-rank 19: ['O=C(O)C1=CCCC1']

NN-rank 20: ['O=C(O)C=C1CCC1']

NN-rank 21: ['O=C(O)C1C=CCC1']

NN-rank 22: ['CCC/C=C/C=O', 'O']

NN-rank 23: ['C=O', 'CCCCC', 'O']

EI-MS matched molecular weight (relative abundance: 31.0%) for C=O

GHS hazard classification: Acute Toxic, Health Hazard, Irritant

EI-MS matched molecular weight (relative abundance: 7.4%) for CCCCC

GHS hazard classification: Health Hazard, Irritant

NN-rank 24: ['C=CC(=O)O', 'CCC']

EI-MS matched molecular weight (relative abundance: 7.4%) for C=CC(=O)O

NN/MS matches corresponding with tox GHS classification highlight in Bold font.

GHS hazard classification: Health Hazard, Irritant

EI-MS matched molecular weight (relative abundance: 18.7%) for CCC

GHS hazard classification: none of the hazards selected for correlation

NN-rank 25: ['CCCC(C)C(=O)O']

NN/MS matches corresponding with tox GHS classification highlight in Bold font.

123. Geraniol: CC(=CCC/C(=C/CO)/C)C

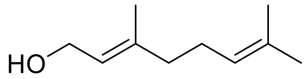

Summary Report of GHS Classification for NN/MS Matches:

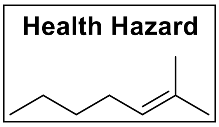

NN-rank 1: ['CC(C)=CCC/C(C)=C/C=O']

NN-rank 2: ['CC(C)=CCCC(C)CCO']

NN-rank 3: ['C', 'CC=CCC/C(C)=C/CO']

NN-rank 4: ['C', 'CC=CCC/C(C)=C/CO']

NN-rank 5: ['C=C(CCO)CCC=C(C)C']

NN-rank 6: ['C/C(=C\\CO)CCCC(C)C']

NN-rank 7: ['CC(C)=CCC=C(C)CCO']

NN-rank 8: ['C=C(C)CCC/C(C)=C/CO']

NN-rank 9: ['C=C(C)CCC/C(C)=C/CO']

NN-rank 10: ['C', 'CCCCC/C(C)=C/CO']

NN-rank 11: ['C', 'CCCCC/C(C)=C/CO']

NN-rank 12: ['C', 'C', 'C=CCC/C(C)=C/CO']

NN-rank 13: ['CC(C)=C=CC/C(C)=C/CO']

NN-rank 14: ['CCCCC=C(C)C', 'CCO']

EI-MS matched molecular weight (relative abundance: 7.81%) for CCCCC=C(C)C

GHS hazard classification: Health Hazard

NN-rank 15: ['C', 'CC(C)=CCCC=CCO']

NN-rank 16: ['C/C1=C\\COC(C)(C)CCC1']

NN-rank 17: ['CCC', 'CCC/C(C)=C/CO']

EI-MS matched molecular weight (relative abundance: 6.01%) for CCC

GHS hazard classification: none of the hazards selected for correlation

NN-rank 18: ['C/C=C(\\C)CCC=C(C)C', 'O']

NN-rank 19: ['C', 'CC1=CCC/C(C)=C/CO1']

NN-rank 20: ['C', 'CC1=CCC/C(C)=C/CO1']

NN-rank 21: ['C', 'CC(C)=CCCCCCO']

NN-rank 22: ['C', 'C', 'CCCC/C(C)=C/CO']

NN/MS matches corresponding with tox GHS classification highlight in Bold font.

NN-rank 23: ['CC=CCC=C(C)C', 'CCO']

NN-rank 24: ['C/C(=C\\CO)CC=CC(C)C']

NN-rank 25: ['CC1(C)CCCC1(C)CCO']

NN/MS matches corresponding with tox GHS classification highlight in Bold font.

124. Geranyl acetate: CC(=CCC/C(=C/COC(=O)C)/C)C

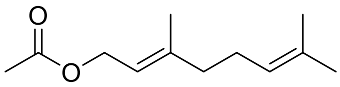

Summary Report of GHS Classification for NN/MS Matches:

Health Hazard	Irritant
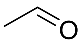	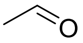

NN-rank 1: ['CC(C)=CCC/C(C)=C/CO', 'CC=O']

EI-MS matched molecular weight (relative abundance: 44.7%) for CC=O

GHS hazard classification: Health Hazard, Irritant

NN-rank 2: ['CC(=O)OC/C=C(\\C)CCCC(C)C']

NN-rank 3: ['C=C=C(C)CCC=C(C)C', 'CC(=O)O']

NN-rank 4: ['CC(=O)OCCC(C)CCC=C(C)C']

NN-rank 5: ['CC(C)=CCC/C(C)=C/C=O', 'CC=O']

EI-MS matched molecular weight (relative abundance: 44.7%) for CC=O

GHS hazard classification: Health Hazard, Irritant

NN-rank 6: ['CC1=CCC(C)(C)CCC1', 'CC(=O)O']

NN-rank 7: ['C=CC(C)CCC=C(C)C', 'CC(=O)O']

NN-rank 8: ['C/C=C(\\C)CCC=C(C)C', 'CC(=O)O']

NN-rank 9: ['CC(=O)OC=C=C(C)CCC=C(C)C']

NN-rank 10: ['CC(=O)OC=CC(C)CCC=C(C)C']

NN-rank 11: ['CC(=O)OCCC(C)=CCC=C(C)C']

NN-rank 12: ['C', 'CCCCC/C(C)=C/COC(C)=O']

NN-rank 13: ['C', 'CCCCC/C(C)=C/COC(C)=O']

NN-rank 14: ['CC(=O)OC1C=C(C)CCCC1(C)C']

NN-rank 15: ['CC1=CC(=O)C(C)(C)CCC1', 'CC=O']

EI-MS matched molecular weight (relative abundance: 44.7%) for CC=O

GHS hazard classification: Health Hazard, Irritant

NN-rank 16: ['CC(=C=C=O)CCC=C(C)C', 'CC=O']

EI-MS matched molecular weight (relative abundance: 44.7%) for CC=O

GHS hazard classification: Health Hazard, Irritant

NN-rank 17: ['C/C1=C\\COC(=O)C=C(C(C)C)CC1']

NN-rank 18: ['C=CC(C)=CCC=C(C)C', 'CC(=O)O']

NN-rank 19: ['CC(=O)O', 'CC1=C=CC(C)(C)CCC1']

NN/MS matches corresponding with tox GHS classification highlight in Bold font.

NN-rank 20: ['C', 'CC=CCC/C(C)=C/COC(C)=O']

NN-rank 21: ['C', 'CC=CCC/C(C)=C/COC(C)=O']

NN-rank 22: ['CC(C)=CCCC(C)C=C=O', 'CC=O']

EI-MS matched molecular weight (relative abundance: 44.7%) for CC=O

GHS hazard classification: Health Hazard, Irritant

NN-rank 23: ['C=C(C)CCC/C(C)=C/COC(C)=O']

NN-rank 24: ['C=C(C)CCC/C(C)=C/COC(C)=O']

NN-rank 25: ['CC(=O)OC/C=C(\\C)CC=C=C(C)C']

NN/MS matches corresponding with tox GHS classification highlight in Bold font.

125. trans-2-Hexenal: CCC/C=C/C=O

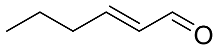

Summary Report of GHS Classification for NN/MS Matches:

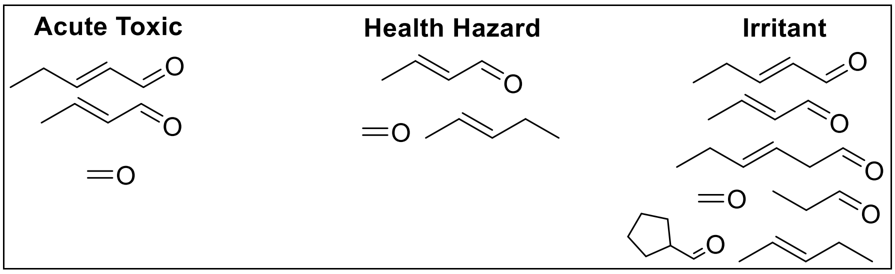

NN-rank 1: ['CCCCCC=O']

NN-rank 2: ['CCC=CCC=O']

EI-MS matched molecular weight (relative abundance: 10.89%) for CCC=CCC=O

GHS hazard classification: Irritant

NN-rank 3: ['CCCCC=C=O']

EI-MS matched molecular weight (relative abundance: 10.89%) for CCCCC=C=O

GHS hazard classification: none of the hazards selected for correlation

NN-rank 4: ['C=O', 'CCCCC']

EI-MS matched molecular weight (relative abundance: 39.79%) for C=O

GHS hazard classification: Acute Toxic, Health Hazard, Irritant

NN-rank 5: ['C=CCC=O', 'CC']

EI-MS matched molecular weight (relative abundance: 46.89%) for C=CCC=O

GHS hazard classification: none of the hazards selected for correlation

EI-MS matched molecular weight (relative abundance: 39.79%) for CC

GHS hazard classification: none of the hazards selected for correlation

NN-rank 6: ['CCC#CCC=O']

NN-rank 7: ['C=O', 'CC=CCC']

EI-MS matched molecular weight (relative abundance: 39.79%) for C=O

GHS hazard classification: Acute Toxic, Health Hazard, Irritant

EI-MS matched molecular weight (relative abundance: 46.89%) for CC=CCC

GHS hazard classification: Health Hazard, Irritant

NN-rank 8: ['CCCCC=CO']

NN-rank 9: ['CCC/C=C/CO']

NN/MS matches corresponding with tox GHS classification highlight in Bold font.

NN-rank 10: ['C', 'CC/C=C/C=O']

EI-MS matched molecular weight (relative abundance: 50.49%) for CC/C=C/C=O

GHS hazard classification: Acute Toxic, Irritant

NN-rank 11: ['O=CC1CCCC1']

EI-MS matched molecular weight (relative abundance: 10.89%) for O=CC1CCCC1

GHS hazard classification: Irritant

NN-rank 12: ['CCC', 'CCC=O']

EI-MS matched molecular weight (relative abundance: 17.29%) for CCC

GHS hazard classification: none of the hazards selected for correlation

EI-MS matched molecular weight (relative abundance: 40.49%) for CCC=O

GHS hazard classification: Irritant

NN-rank 13: ['CCC=CC=C=O']

NN-rank 14: ['O=CCC1CCC1']

EI-MS matched molecular weight (relative abundance: 10.89%) for O=CCC1CCC1

GHS hazard classification: none of the hazards selected for correlation

NN-rank 15: ['C/C=C/C=O', 'CC']

EI-MS matched molecular weight (relative abundance: 46.89%) for C/C=C/C=O

GHS hazard classification: Acute Toxic, Health Hazard, Irritant

EI-MS matched molecular weight (relative abundance: 39.79%) for CC

GHS hazard classification: none of the hazards selected for correlation

NN-rank 16: ['CCCC=C=C=O']

NN-rank 17: ['CCCC1CCO1']

NN-rank 18: ['C=O', 'CC#CCC']

EI-MS matched molecular weight (relative abundance: 39.79%) for C=O

GHS hazard classification: Acute Toxic, Health Hazard, Irritant

NN-rank 19: ['C=O', 'C=CCCC']

EI-MS matched molecular weight (relative abundance: 39.79%) for C=O

GHS hazard classification: Acute Toxic, Health Hazard, Irritant

EI-MS matched molecular weight (relative abundance: 46.89%) for C=CCCC

GHS hazard classification: none of the hazards selected for correlation

NN-rank 20: ['C#CCC=O', 'CC']

EI-MS matched molecular weight (relative abundance: 39.79%) for CC

GHS hazard classification: none of the hazards selected for correlation

NN-rank 21: ['CCCC(C)C=O']

NN-rank 22: ['C1CCCC1', 'C=O']

NN/MS matches corresponding with tox GHS classification highlight in Bold font.

EI-MS matched molecular weight (relative abundance: 46.89%) for C1CCCC1

GHS hazard classification: none of the hazards selected for correlation

EI-MS matched molecular weight (relative abundance: 39.79%) for C=O

GHS hazard classification: Acute Toxic, Health Hazard, Irritant

NN-rank 23: ['CCC=C=CC=O']

NN-rank 24: ['CC1CCC1C=O']

EI-MS matched molecular weight (relative abundance: 10.89%) for CC1CCC1C=O

GHS hazard classification: none of the hazards selected for correlation

NN-rank 25: ['CCCCC', 'CO']

NN/MS matches corresponding with tox GHS classification highlight in Bold font.

126. Thio Menthone: CC(C1)CCC(C(C)(S)C)C1=O

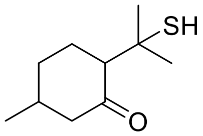

Summary Report of GHS Classification for NN/MS Matches:

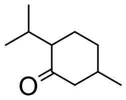

NN-rank 1: ['CC1CCC(C(C)(C)S)CC1', 'O']

NN-rank 2: ['CC1CCC(C(C)C)C(=O)C1', 'S']

EI-MS matched molecular weight (relative abundance: 14.0%) for CC1CCC(C(C)C)C(=O)C1

GHS hazard classification: Irritant

NN-rank 3: ['CC1CCC(C(C)(C)S)C(O)C1']

NN-rank 4: ['CC(CC=O)CCCC(C)(C)S']

NN-rank 5: ['C', 'CC(C)(S)C1CCCCC1=O']

NN-rank 6: ['CC1CC=C(C(C)(C)S)C(=O)C1']

NN-rank 7: ['CC1CC=C(C(C)(C)S)CC1', 'O']

NN-rank 8: ['CC1=CC(=O)C(C(C)(C)S)CC1']

NN-rank 9: ['CC(CCO)CCCC(C)(C)S']

NN-rank 10: ['CC1CCC2C(=O)C1SC2(C)C']

NN-rank 11: ['CC1C=CC(C(C)(C)S)CC1', 'O']

NN-rank 12: ['CC(C)(S)C1C(=O)CC2CC1C2']

NN-rank 13: ['CC(CC=O)CC=CC(C)(C)S']

NN-rank 14: ['CC1CCC(C(C)(C)S)=C(O)C1']

NN-rank 15: ['CC1CCCC(=O)C1C(C)(C)S']

NN-rank 16: ['CCC(C)CC(=O)CC(C)(C)S']

NN-rank 17: ['CC(C)CC(=O)C(C)C(C)(C)S']

NN-rank 18: ['CC(C=C=O)CCCC(C)(C)S']

NN-rank 19: ['CCCC(=O)C(CC)C(C)(C)S']

NN-rank 20: ['CC1CCC(C(C)(C)S)C(=O)C1']

NN-rank 21: ['CC1C=C(O)C(C(C)(C)S)CC1']

NN/MS matches corresponding with tox GHS classification highlight in Bold font.

NN-rank 22: ['CCCCCC(C(C)=O)C(C)(C)S']

NN-rank 23: ['C', 'CC(C)(S)C1CCC=CC1=O']

NN-rank 24: ['C=C(C(=O)CC(C)C)C(C)(C)S']

NN-rank 25: ['C=CC(C)CCCC(C)(C)S', 'O']

NN/MS matches corresponding with tox GHS classification highlight in Bold font.

127. Guaiacol: COC1=CC=CC=C1O

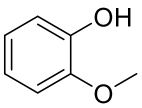

Summary Report of GHS Classification for NN/MS Matches:

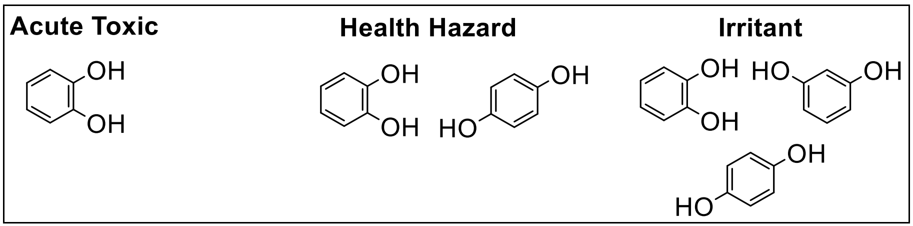

NN-rank 1: ['C', 'Oc1ccccc1O']

EI-MS matched molecular weight (relative abundance: 99.99%) for Oc1ccccc1O

GHS hazard classification: Acute Toxic, Health Hazard, Irritant

NN-rank 2: ['Cc1cccc(O)c1O']

NN-rank 3: ['C', 'Oc1c2cccc1O2']

NN-rank 4: ['C', 'Oc1cccc2c1O2']

NN-rank 5: ['C', 'Oc1cc2ccc1o2']

NN-rank 6: ['Oc1c2cccc1OC2']

NN-rank 7: ['CO', 'Oc1ccccc1']

NN-rank 8: ['C', 'Oc1ccc2cc1O2']

NN-rank 9: ['Cc1cccc(O)c1O']

NN-rank 10: ['COc1ccccc1', 'O']

NN-rank 11: ['Oc1cccc2c1OC2']

NN-rank 12: ['COc1c2ccc-2c1O']

NN-rank 13: ['C', 'Oc1cccc(O)c1']

EI-MS matched molecular weight (relative abundance: 99.99%) for Oc1cccc(O)c1

GHS hazard classification: Irritant

NN-rank 14: ['Cc1ccc(O)c(O)c1']

NN-rank 15: ['Oc1cc2ccc1OC2']

NN-rank 16: ['COc1cccc(O)c1']

NN-rank 17: ['C', 'Oc1ccccc1O']

EI-MS matched molecular weight (relative abundance: 99.99%) for Oc1ccccc1O

GHS hazard classification: Acute Toxic, Health Hazard, Irritant

NN/MS matches corresponding with tox GHS classification highlight in Bold font.

NN-rank 18: ['C', 'Oc1cccc(O)c1']

EI-MS matched molecular weight (relative abundance: 99.99%) for Oc1cccc(O)c1

GHS hazard classification: Irritant

NN-rank 19: ['Cc1ccc(O)c(O)c1']

NN-rank 20: ['Oc1ccc2cc1OC2']

NN-rank 21: ['COc1ccccc1O']

NN-rank 22: ['COc1cccc(O)c1']

NN-rank 23: ['C', 'Oc1ccc(O)cc1']

EI-MS matched molecular weight (relative abundance: 99.99%) for Oc1ccc(O)cc1

GHS hazard classification: Health Hazard, Irritant

NN-rank 24: ['Cc1ccc2c(c1O)O2']

NN-rank 25: ['C=O', 'Oc1ccccc1']

NN/MS matches corresponding with tox GHS classification highlight in Bold font.

128. Hexanal: CCCCCC=O

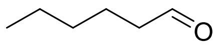

Summary Report of GHS Classification for NN/MS Matches:

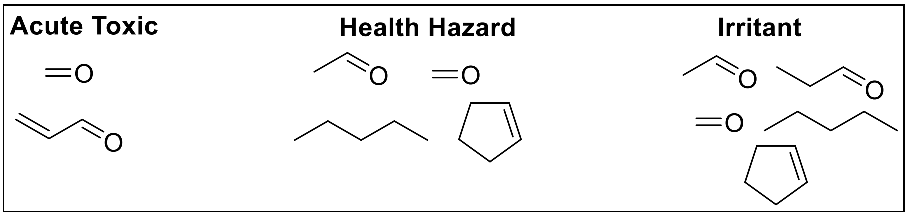

NN-rank 1: ['CCCC=CC=O']

NN-rank 2: ['CCCCCCO']

NN-rank 3: ['C', 'CCCCC=O']

NN-rank 4: ['C=O', 'C=CCCC']

EI-MS matched molecular weight (relative abundance: 32.99%) for C=O

GHS hazard classification: Acute Toxic, Health Hazard, Irritant

NN-rank 5: ['CC', 'CCCC=O']

EI-MS matched molecular weight (relative abundance: 32.99%) for CC

GHS hazard classification: none of the hazards selected for correlation

EI-MS matched molecular weight (relative abundance: 6.69%) for CCCC=O

GHS hazard classification: none of the hazards selected for correlation

NN-rank 6: ['C=O', 'CCCCC']

EI-MS matched molecular weight (relative abundance: 32.99%) for C=O

GHS hazard classification: Acute Toxic, Health Hazard, Irritant

EI-MS matched molecular weight (relative abundance: 6.69%) for CCCCC

GHS hazard classification: Health Hazard, Irritant

NN-rank 7: ['CC=O', 'CCCC']

EI-MS matched molecular weight (relative abundance: 55.09%) for CC=O

GHS hazard classification: Health Hazard, Irritant

EI-MS matched molecular weight (relative abundance: 38.09%) for CCCC

GHS hazard classification: none of the hazards selected for correlation

NN-rank 8: ['CCC', 'CCC=O']

EI-MS matched molecular weight (relative abundance: 55.09%) for CCC

GHS hazard classification: none of the hazards selected for correlation

EI-MS matched molecular weight (relative abundance: 38.09%) for CCC=O

NN/MS matches corresponding with tox GHS classification highlight in Bold font.

GHS hazard classification: Irritant

NN-rank 9: ['CCCCC=C=O']

NN-rank 10: ['C=CCCCC=O']

NN-rank 11: ['CCC=CCC=O']

NN-rank 12: ['O=CC1=CCCC1']

NN-rank 13: ['CCCCCC', 'O']

NN-rank 14: ['C=CCCCC', 'O']

NN-rank 15: ['C=CC=O', 'CCC']

EI-MS matched molecular weight (relative abundance: 15.29%) for C=CC=O

GHS hazard classification: Acute Toxic

EI-MS matched molecular weight (relative abundance: 55.09%) for CCC

GHS hazard classification: none of the hazards selected for correlation

NN-rank 16: ['CCCCC=CO']

NN-rank 17: ['O=CCC1CCC1']

NN-rank 18: ['C1CCCOCC1']

NN-rank 19: ['O=C1CCCCC1']

NN-rank 20: ['C=C(C=O)CCC']

NN-rank 21: ['OC1CCCCC1']

NN-rank 22: ['C1=CCCC1', 'C=O']

EI-MS matched molecular weight (relative abundance: 8.09%) for C1=CCCC1

GHS hazard classification: Health Hazard, Irritant

EI-MS matched molecular weight (relative abundance: 32.99%) for C=O

GHS hazard classification: Acute Toxic, Health Hazard, Irritant

NN-rank 23: ['CCCC=C=C=O']

NN-rank 24: ['CC1CCC1C=O']

NN-rank 25: ['CCC(C)CC=O']

NN/MS matches corresponding with tox GHS classification highlight in Bold font.

129. Hexanoic Acid: CCCCCC(=O)O

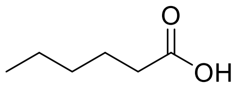

NN-rank 1: ['CCCCCC(=O)O']

NN-rank 2: ['C', 'CCCCC(=O)O']

NN-rank 3: ['O=C1CCCCCO1']

NN-rank 4: ['CCCCCCO', 'O']

NN-rank 5: ['CCCCCCO', 'O']

NN-rank 6: ['CC', 'CCCC(=O)O']

EI-MS matched molecular weight (relative abundance: 10.51%) for CC

GHS hazard classification: none of the hazards selected for correlation

EI-MS matched molecular weight (relative abundance: 13.41%) for CCCC(=O)O

GHS hazard classification: none of the hazards selected for correlation

NN-rank 7: ['CC(=O)O', 'CCCC']

EI-MS matched molecular weight (relative abundance: 9.91%) for CCCC

GHS hazard classification: none of the hazards selected for correlation

NN-rank 8: ['CCCCC(=O)OC']

NN-rank 9: ['CCCCC', 'O=CO']

EI-MS matched molecular weight (relative abundance: 10.91%) for O=CO

GHS hazard classification: none of the hazards selected for correlation

NN-rank 10: ['CCC', 'CCC(=O)O']

EI-MS matched molecular weight (relative abundance: 15.51%) for CCC

GHS hazard classification: none of the hazards selected for correlation

EI-MS matched molecular weight (relative abundance: 44.54%) for CCC(=O)O

GHS hazard classification: none of the hazards selected for correlation

NN-rank 11: ['CCCC=CC(=O)O']

NN-rank 12: ['CCCCCC=O', 'O']

NN-rank 13: ['C=CCCCC(=O)O']

NN-rank 14: ['CCCCCC=O', 'O']

NN-rank 15: ['CCCCC', 'O=CO']

EI-MS matched molecular weight (relative abundance: 10.91%) for O=CO

GHS hazard classification: none of the hazards selected for correlation

NN-rank 16: ['CCCC(C)C(=O)O']

NN-rank 17: ['OCCCCCCO']

NN/MS matches corresponding with tox GHS classification highlight in Bold font.

NN-rank 18: ['CCCCC=CO', 'O']

NN-rank 19: ['O=C(O)C1CCCC1']

NN-rank 20: ['CCCCC1OC1=O']

NN-rank 21: ['CCCCCC', 'O', 'O']

NN-rank 22: ['CCCCC=C=O', 'O']

NN-rank 23: ['CCCCC(=O)C=O']

NN-rank 24: ['O=CCCCCCO']

NN-rank 25: ['C=CCCCC', 'O', 'O']

NN/MS matches corresponding with tox GHS classification highlight in Bold font.

130. Hexyl Acetate: CCCCCCOC(=O)C

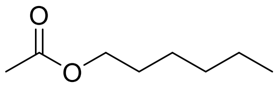

Summary Report of GHS Classification for NN/MS Matches:

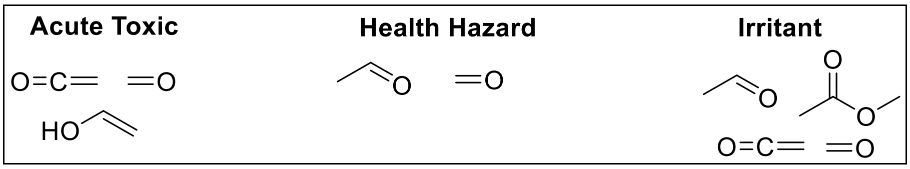

NN-rank 1: ['CC=O', 'CCCCCCO']

EI-MS matched molecular weight (relative abundance: 99.99%) for CC=O

GHS hazard classification: Health Hazard, Irritant

NN-rank 2: ['CC(=O)O', 'CCCCCC']

NN-rank 3: ['CCCCCCO', 'CCO']

NN-rank 4: ['CC=O', 'CCCCCC=O']

EI-MS matched molecular weight (relative abundance: 99.99%) for CC=O

GHS hazard classification: Health Hazard, Irritant

NN-rank 5: ['CCCCC', 'COC(C)=O']

EI-MS matched molecular weight (relative abundance: 11.39%) for COC(C)=O

GHS hazard classification: Irritant

NN-rank 6: ['C', 'CCCCCOC(C)=O']

NN-rank 7: ['CC(=O)CCCCCCO']

NN-rank 8: ['C=C=O', 'CCCCCCO']

EI-MS matched molecular weight (relative abundance: 20.19%) for C=C=O

GHS hazard classification: Acute Toxic, Irritant

NN-rank 9: ['CC(C)=O', 'CCCCCO']

NN-rank 10: ['CC(=O)O', 'C=CCCCC']

NN-rank 11: ['CC', 'CCCCOC(C)=O']

EI-MS matched molecular weight (relative abundance: 9.29%) for CC

GHS hazard classification: none of the hazards selected for correlation

NN-rank 12: ['CCCC', 'CCOC(C)=O']

NN-rank 13: ['CCCCC=COC(C)=O']

NN-rank 14: ['CCCCCCOC(C)O']

NN-rank 15: ['C', 'C=O', 'CCCCCCO']

NN/MS matches corresponding with tox GHS classification highlight in Bold font.

EI-MS matched molecular weight (relative abundance: 9.29%) for C=O

GHS hazard classification: Acute Toxic, Health Hazard, Irritant

NN-rank 16: ['C', 'CCCCCCOC=O']

NN-rank 17: ['CCC', 'CCCOC(C)=O']

EI-MS matched molecular weight (relative abundance: 99.99%) for CCC

GHS hazard classification: none of the hazards selected for correlation

NN-rank 18: ['CCOCCCCCCO']

NN-rank 19: ['C=CO', 'CCCCCCO']

EI-MS matched molecular weight (relative abundance: 99.99%) for C=CO

GHS hazard classification: Acute Toxic

NN-rank 20: ['CCCCCC=O', 'CCO']

NN-rank 21: ['CC(O)CCCCCCO']

NN-rank 22: ['C=O', 'CC=O', 'CCCCC']

EI-MS matched molecular weight (relative abundance: 9.29%) for C=O

GHS hazard classification: Acute Toxic, Health Hazard, Irritant

EI-MS matched molecular weight (relative abundance: 99.99%) for CC=O

GHS hazard classification: Health Hazard, Irritant

NN-rank 23: ['CC(=O)CCCCCC=O']

NN-rank 24: ['C=C=O', 'CCCCCC=O']

EI-MS matched molecular weight (relative abundance: 20.19%) for C=C=O

GHS hazard classification: Acute Toxic, Irritant

NN-rank 25: ['C=COC(C)=O', 'CCCC']

NN/MS matches corresponding with tox GHS classification highlight in Bold font.

131. Hexyl Butyrate: CCCCCCOC(=O)CCC

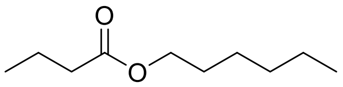

Summary Report of GHS Classification for NN/MS Matches:

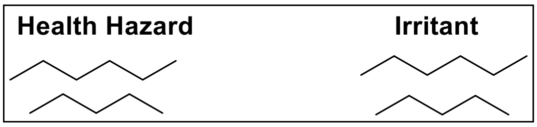

NN-rank 1: ['CCCC(=O)O', 'CCCCCC']

EI-MS matched molecular weight (relative abundance: 5.81%) for CCCCCC

GHS hazard classification: Health Hazard, Irritant

NN-rank 2: ['CCCC(=O)O', 'CCCCCC']

EI-MS matched molecular weight (relative abundance: 5.81%) for CCCCCC

GHS hazard classification: Health Hazard, Irritant

NN-rank 3: ['CCCCCCO', 'CCCCO']

NN-rank 4: ['CCCC=O', 'CCCCCCO']

EI-MS matched molecular weight (relative abundance: 90.98%) for CCCC=O

GHS hazard classification: none of the hazards selected for correlation

NN-rank 5: ['CCCC=O', 'CCCCCC', 'O']

EI-MS matched molecular weight (relative abundance: 90.98%) for CCCC=O

GHS hazard classification: none of the hazards selected for correlation

EI-MS matched molecular weight (relative abundance: 5.81%) for CCCCCC

GHS hazard classification: Health Hazard, Irritant

NN-rank 6: ['CCCC(=O)OC', 'CCCCC']

EI-MS matched molecular weight (relative abundance: 90.98%) for CCCCC

GHS hazard classification: Health Hazard, Irritant

NN-rank 7: ['C', 'CCCCCOC(=O)CCC']

NN-rank 8: ['C', 'CCCCCCOC(=O)CC']

NN-rank 9: ['C', 'CCCC(=O)O', 'CCCCC']

EI-MS matched molecular weight (relative abundance: 90.98%) for CCCCC

GHS hazard classification: Health Hazard, Irritant

NN-rank 10: ['CC1CCCCCCOC(=O)C1']

NN-rank 11: ['CCCCCOC(=O)CC(C)C']

NN-rank 12: ['CCCCCCOCCCC', 'O']

NN/MS matches corresponding with tox GHS classification highlight in Bold font.

NN-rank 13: ['CCCC(=O)O', 'C=CCCCC']

NN-rank 14: ['C', 'CCCC(=O)O', 'CCCCC']

EI-MS matched molecular weight (relative abundance: 90.98%) for CCCCC

GHS hazard classification: Health Hazard, Irritant

NN-rank 15: ['CCCCCOC(=O)CCCC']

NN-rank 16: ['CCCC(=O)O', 'C=CCCCC']

NN-rank 17: ['CCCCCOC(=O)C(C)CC']

NN-rank 18: ['O=C1CCCCCCCCCO1']

NN-rank 19: ['CCCCCCCOC(=O)CC']

NN-rank 20: ['CCCC', 'CCCC(=O)OCC']

EI-MS matched molecular weight (relative abundance: 7.61%) for CCCC

GHS hazard classification: none of the hazards selected for correlation

NN-rank 21: ['CCCC', 'CCCCCCO', 'O']

EI-MS matched molecular weight (relative abundance: 7.61%) for CCCC

GHS hazard classification: none of the hazards selected for correlation

NN-rank 22: ['CC', 'CCCCOC(=O)CCC']

EI-MS matched molecular weight (relative abundance: 14.31%) for CC

GHS hazard classification: none of the hazards selected for correlation

NN-rank 23: ['CC1CCCCOC(=O)CCC1']

NN-rank 24: ['CCC(=O)OCCCCC(C)C']

NN-rank 25: ['CCC1CCCCCCOC1=O']

NN/MS matches corresponding with tox GHS classification highlight in Bold font.

132. Ionone (mixed isomers): O=C(/C=C/C1C(C)=CCCC1(C)C)C

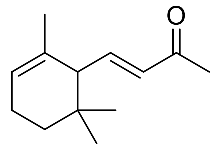

NN-rank 1: ['CC1=CCCC(C)(C)C1/C=C/C(C)O']

NN-rank 2: ['C=C1CCCC(C)(C)C1/C=C/C(C)=O']

NN-rank 3: ['CC(=O)/C=C/C1=C(C)CCCC1(C)C']

NN-rank 4: ['C=C1C=CCC(C)(C)C1/C=C/C(C)=O']

NN-rank 5: ['CC(=O)/C=C/C1C(C)CCCC1(C)C']

NN-rank 6: ['CC(=O)CCC1C(C)=CCCC1(C)C']

NN-rank 7: ['CC(=O)C=C=C1C(C)=CCCC1(C)C']

NN-rank 8: ['CC(=O)/C=C/C1C(C)=C=CCC1(C)C']

NN-rank 9: ['CC(=O)/C=C/C1C(C)C=CCC1(C)C']

NN-rank 10: ['CC(=O)CC=C1C(C)=CCCC1(C)C']

NN-rank 11: ['CC(=O)/C=C/C1=C(C)C=CCC1(C)C']

NN-rank 12: ['CC(=O)/C=C/C1C(C)=CC=CC1(C)C']

NN-rank 13: ['C=C(O)/C=C/C1C(C)=CCCC1(C)C']

NN-rank 14: ['C', 'CC1=CCCC(C)(C)C1/C=C/CO']

NN-rank 15: ['CC1=CCCC(C)(C)C1C1=CC(=O)C1']

NN-rank 16: ['CC(=O)/C=C/C=C(C)CCCC(C)C']

NN-rank 17: ['CC(=O)C=C=CC(C)=CCCC(C)C']

NN-rank 18: ['CC(=O)/C=C/CC(C)=CCCC(C)C']

NN-rank 19: ['C', 'CC(=O)/C=C/C1=C=CCCC1(C)C']

NN-rank 20: ['CC1=CCCC(C)(C)C1C1CC(=O)C1']

NN-rank 21: ['C=C(C)C(/C=C/C(C)=O)C(C)(C)CC']

NN-rank 22: ['C', 'CC(=O)/C=C/C1CCCCC1(C)C']

NN-rank 23: ['CCC(C)(C)C(/C=C/C(C)=O)C(C)C']

NN-rank 24: ['CCC=C(C)C(/C=C/C(C)=O)C(C)C']

NN-rank 25: ['CCC(C)(C)C(/C=C/C(C)=O)=C(C)C']

NN/MS matches corresponding with tox GHS classification highlight in Bold font.

133. Isoamyl Acetate: CC(C)CCOC(=O)C

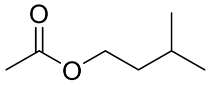

Summary Report of GHS Classification for NN/MS Matches:

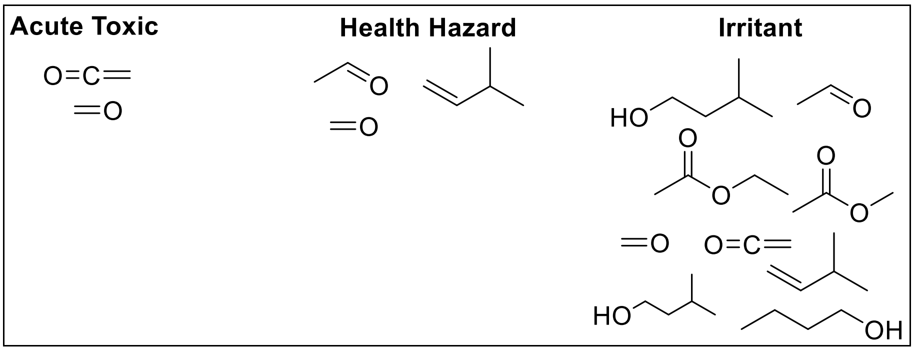

NN-rank 1: ['CC(C)CCO', 'CC=O']

EI-MS matched molecular weight (relative abundance: 13.4%) for CC(C)CCO

GHS hazard classification: Irritant

EI-MS matched molecular weight (relative abundance: 99.99%) for CC=O

GHS hazard classification: Health Hazard, Irritant

NN-rank 2: ['CC(C)CC=O', 'CC=O']

EI-MS matched molecular weight (relative abundance: 99.99%) for CC=O

GHS hazard classification: Health Hazard, Irritant

NN-rank 3: ['CC(C)CCO', 'CCO']

EI-MS matched molecular weight (relative abundance: 13.4%) for CC(C)CCO

GHS hazard classification: Irritant

NN-rank 4: ['C=C=O', 'CC(C)CCO']

EI-MS matched molecular weight (relative abundance: 18.0%) for C=C=O

GHS hazard classification: Acute Toxic, Irritant

EI-MS matched molecular weight (relative abundance: 13.4%) for CC(C)CCO

GHS hazard classification: Irritant

NN-rank 5: ['C', 'CCCCOC(C)=O']

NN-rank 6: ['C', 'CCCCOC(C)=O']

NN-rank 7: ['CC(=O)O', 'CCC(C)C']

NN-rank 8: ['CC(C)C', 'COC(C)=O']

EI-MS matched molecular weight (relative abundance: 9.6%) for COC(C)=O

NN/MS matches corresponding with tox GHS classification highlight in Bold font.

GHS hazard classification: Irritant

NN-rank 9: ['CC(=O)CC(C)CCO']

NN-rank 10: ['CC(=O)CC(C)CCO']

NN-rank 11: ['C=CC(C)C', 'CC(=O)O']

EI-MS matched molecular weight (relative abundance: 7.1%) for C=CC(C)C

GHS hazard classification: Health Hazard, Irritant

NN-rank 12: ['CC(=O)OC=CC(C)C']

NN-rank 13: ['C', 'C=O', 'CC(C)CCO']

EI-MS matched molecular weight (relative abundance: 5.6%) for C=O

GHS hazard classification: Acute Toxic, Health Hazard, Irritant

EI-MS matched molecular weight (relative abundance: 13.4%) for CC(C)CCO

GHS hazard classification: Irritant

NN-rank 14: ['CC(C)CCOC(C)O']

NN-rank 15: ['CCC', 'CCOC(C)=O']

EI-MS matched molecular weight (relative abundance: 99.99%) for CCC

GHS hazard classification: none of the hazards selected for correlation

EI-MS matched molecular weight (relative abundance: 13.4%) for CCOC(C)=O

GHS hazard classification: Irritant

NN-rank 16: ['CC(C)CC=O', 'CCO']

NN-rank 17: ['CC(C)=O', 'CCCCO']

EI-MS matched molecular weight (relative abundance: 9.6%) for CCCCO

GHS hazard classification: Irritant

NN-rank 18: ['CC(C)=O', 'CCCCO']

EI-MS matched molecular weight (relative abundance: 9.6%) for CCCCO

GHS hazard classification: Irritant

NN-rank 19: ['C', 'CC(C)CCOC=O']

NN-rank 20: ['C=C=O', 'CC(C)CC=O']

EI-MS matched molecular weight (relative abundance: 18.0%) for C=C=O

GHS hazard classification: Acute Toxic, Irritant

NN-rank 21: ['C=CO', 'CC(C)CCO']

EI-MS matched molecular weight (relative abundance: 99.99%) for C=CO

GHS hazard classification: Acute Toxic

EI-MS matched molecular weight (relative abundance: 13.4%) for CC(C)CCO

GHS hazard classification: Irritant

NN-rank 22: ['C', 'C', 'CCCOC(C)=O']

NN/MS matches corresponding with tox GHS classification highlight in Bold font.

NN-rank 23: ['CC(C)C=C=O', 'CC=O']

EI-MS matched molecular weight (relative abundance: 99.99%) for CC=O

GHS hazard classification: Health Hazard, Irritant

NN-rank 24: ['C=O', 'CC(C)C', 'CC=O']

EI-MS matched molecular weight (relative abundance: 5.6%) for C=O

GHS hazard classification: Acute Toxic, Health Hazard, Irritant

EI-MS matched molecular weight (relative abundance: 99.99%) for CC=O

GHS hazard classification: Health Hazard, Irritant

NN-rank 25: ['CC(C)CC1=CC(=O)O1']

NN/MS matches corresponding with tox GHS classification highlight in Bold font.

134. Isoamyl Alcohol: CC(C)CCO

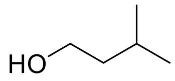

Summary Report of GHS Classification for NN/MS Matches:

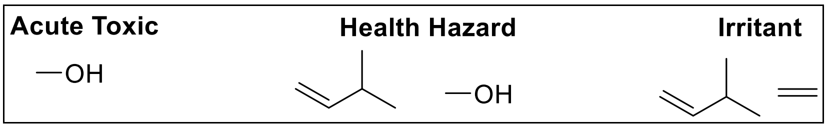

NN-rank 1: ['C=CC(C)C', 'O']

EI-MS matched molecular weight (relative abundance: 5.3%) for C=CC(C)C

GHS hazard classification: Health Hazard, Irritant

NN-rank 2: ['C#CC(C)C', 'O']

NN-rank 3: ['CCC(C)C', 'O']

NN-rank 4: ['C', 'CCCCO']

EI-MS matched molecular weight (relative abundance: 20.12%) for C

GHS hazard classification: none of the hazards selected for correlation

NN-rank 5: ['C', 'CCCCO']

EI-MS matched molecular weight (relative abundance: 20.12%) for C

GHS hazard classification: none of the hazards selected for correlation

NN-rank 6: ['CC(C)CC=O']

NN-rank 7: ['CCC(C)(C)O']

NN-rank 8: ['CCC', 'CCO']

EI-MS matched molecular weight (relative abundance: 82.37%) for CCC

GHS hazard classification: none of the hazards selected for correlation

EI-MS matched molecular weight (relative abundance: 19.92%) for CCO

GHS hazard classification: none of the hazards selected for correlation

NN-rank 9: ['CC(C)C', 'CO']

EI-MS matched molecular weight (relative abundance: 30.73%) for CC(C)C

GHS hazard classification: none of the hazards selected for correlation

EI-MS matched molecular weight (relative abundance: 61.26%) for CO

GHS hazard classification: Acute Toxic, Health Hazard

NN-rank 10: ['CC1(C)CC1', 'O']

EI-MS matched molecular weight (relative abundance: 5.3%) for CC1(C)CC1

GHS hazard classification: none of the hazards selected for correlation

NN/MS matches corresponding with tox GHS classification highlight in Bold font.

NN-rank 11: ['C=C', 'CCC', 'O']

EI-MS matched molecular weight (relative abundance: 59.45%) for C=C

GHS hazard classification: Irritant

EI-MS matched molecular weight (relative abundance: 82.37%) for CCC

GHS hazard classification: none of the hazards selected for correlation

NN-rank 12: ['C#C', 'CCC', 'O']

EI-MS matched molecular weight (relative abundance: 82.37%) for CCC

GHS hazard classification: none of the hazards selected for correlation

NN-rank 13: ['C', 'CC(C)C', 'O']

EI-MS matched molecular weight (relative abundance: 20.12%) for C

GHS hazard classification: none of the hazards selected for correlation

EI-MS matched molecular weight (relative abundance: 30.73%) for CC(C)C

GHS hazard classification: none of the hazards selected for correlation

NN-rank 14: ['CC(C)C(C)O']

NN-rank 15: ['CCC(C)CO']

NN-rank 16: ['CCC(C)CO']

NN-rank 17: ['CC(C)C#CO']

NN-rank 18: ['C', 'C', 'CCCO']

EI-MS matched molecular weight (relative abundance: 20.12%) for C

GHS hazard classification: none of the hazards selected for correlation

EI-MS matched molecular weight (relative abundance: 20.12%) for C

GHS hazard classification: none of the hazards selected for correlation

NN-rank 19: ['CC(C)C=CO']

NN-rank 20: ['C', 'CCC(C)O']

EI-MS matched molecular weight (relative abundance: 20.12%) for C

GHS hazard classification: none of the hazards selected for correlation

NN-rank 21: ['C', 'CCC(C)O']

EI-MS matched molecular weight (relative abundance: 20.12%) for C

GHS hazard classification: none of the hazards selected for correlation

NN-rank 22: ['C=C(C)CCO']

NN-rank 23: ['CC1(C)CCO1']

NN-rank 24: ['CC(C)C1CO1']

NN-rank 25: ['C', 'CC1CC1', 'O']

EI-MS matched molecular weight (relative abundance: 20.12%) for C

GHS hazard classification: none of the hazards selected for correlation

NN/MS matches corresponding with tox GHS classification highlight in Bold font.

EI-MS matched molecular weight (relative abundance: 99.99%) for CC1CC1

GHS hazard classification: none of the hazards selected for correlation

NN/MS matches corresponding with tox GHS classification highlight in Bold font.

135. Isoamyl Butyrate: CCCC(=O)OCCC(C)C

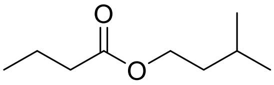

Summary Report of GHS Classification for NN/MS Matches:

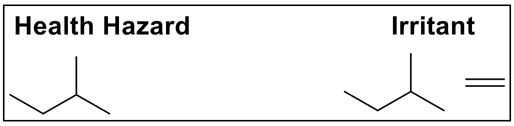

NN-rank 1: ['CCC(C)C', 'CCCC(=O)O']

EI-MS matched molecular weight (relative abundance: 99.99%) for CCC(C)C

GHS hazard classification: Health Hazard, Irritant

NN-rank 2: ['CCC(C)C', 'CCCC(=O)O']

EI-MS matched molecular weight (relative abundance: 99.99%) for CCC(C)C

GHS hazard classification: Health Hazard, Irritant

NN-rank 3: ['CC(C)CCO', 'CCCCO']

NN-rank 4: ['CC(C)CCO', 'CCCC=O']

EI-MS matched molecular weight (relative abundance: 99.99%) for CCCC=O

GHS hazard classification: none of the hazards selected for correlation

NN-rank 5: ['CCC(C)C', 'CCCC=O', 'O']

EI-MS matched molecular weight (relative abundance: 99.99%) for CCC(C)C

GHS hazard classification: Health Hazard, Irritant

EI-MS matched molecular weight (relative abundance: 99.99%) for CCCC=O

GHS hazard classification: none of the hazards selected for correlation

NN-rank 6: ['CC(C)C', 'CCCC(=O)OC']

NN-rank 7: ['C=CC(C)C', 'CCCC(=O)O']

NN-rank 8: ['C', 'CCC(=O)OCCC(C)C']

NN-rank 9: ['C', 'CCCCOC(=O)CCC']

NN-rank 10: ['C', 'CCCCOC(=O)CCC']

NN-rank 11: ['CCCCOCCC(C)C', 'O']

NN-rank 12: ['C=CC(C)C', 'CCCC(=O)O']

NN-rank 13: ['C', 'CC(C)C', 'CCCC(=O)O']

NN-rank 14: ['CC(C)CCO', 'CCCC', 'O']

NN-rank 15: ['C', 'CC(C)C', 'CCCC(=O)O']

NN-rank 16: ['CCC(C)C', 'CCC1OC1=O']

NN/MS matches corresponding with tox GHS classification highlight in Bold font.

EI-MS matched molecular weight (relative abundance: 99.99%) for CCC(C)C

GHS hazard classification: Health Hazard, Irritant

NN-rank 17: ['CCC', 'CCCC(=O)OCC']

EI-MS matched molecular weight (relative abundance: 81.79%) for CCC

GHS hazard classification: none of the hazards selected for correlation

NN-rank 18: ['CC1CCOC(=O)CC(C)C1']

NN-rank 19: ['CC1CCOC(=O)CC(C)C1']

NN-rank 20: ['CCCC(=O)OC=CC(C)C']

NN-rank 21: ['CC', 'CC(=O)OCCC(C)C']

EI-MS matched molecular weight (relative abundance: 8.39%) for CC

GHS hazard classification: none of the hazards selected for correlation

NN-rank 22: ['C=CC(C)C', 'CCCC=O', 'O']

EI-MS matched molecular weight (relative abundance: 99.99%) for CCCC=O

GHS hazard classification: none of the hazards selected for correlation

NN-rank 23: ['C=C', 'CCC', 'CCCC(=O)O']

EI-MS matched molecular weight (relative abundance: 12.09%) for C=C

GHS hazard classification: Irritant

EI-MS matched molecular weight (relative abundance: 81.79%) for CCC

GHS hazard classification: none of the hazards selected for correlation

NN-rank 24: ['CC(C)CCOC=O', 'CCC']

EI-MS matched molecular weight (relative abundance: 81.79%) for CCC

GHS hazard classification: none of the hazards selected for correlation

NN-rank 25: ['CCC(C)C', 'CCCCO', 'O']

EI-MS matched molecular weight (relative abundance: 99.99%) for CCC(C)C

GHS hazard classification: Health Hazard, Irritant

NN/MS matches corresponding with tox GHS classification highlight in Bold font.

136. Isoamyl Isovalerate: CC(C)CCOC(=O)CC(C)C

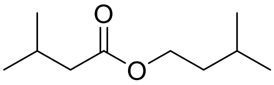

Summary Report of GHS Classification for NN/MS Matches:

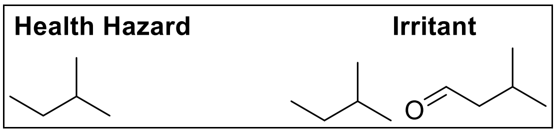

NN-rank 1: ['CC(C)CC(=O)O', 'CCC(C)C']

EI-MS matched molecular weight (relative abundance: 31.39%) for CCC(C)C

GHS hazard classification: Health Hazard, Irritant

NN-rank 2: ['CC(C)CC(=O)O', 'CCC(C)C']

EI-MS matched molecular weight (relative abundance: 31.39%) for CCC(C)C

GHS hazard classification: Health Hazard, Irritant

NN-rank 3: ['CC(C)CCO', 'CC(C)CCO']

NN-rank 4: ['CC(C)CC=O', 'CC(C)CCO']

EI-MS matched molecular weight (relative abundance: 56.69%) for CC(C)CC=O

GHS hazard classification: Irritant

NN-rank 5: ['CC(C)C', 'COC(=O)CC(C)C']

EI-MS matched molecular weight (relative abundance: 37.69%) for CC(C)C

GHS hazard classification: none of the hazards selected for correlation

NN-rank 6: ['CC(C)CC=O', 'CCC(C)C', 'O']

EI-MS matched molecular weight (relative abundance: 56.69%) for CC(C)CC=O

GHS hazard classification: Irritant

EI-MS matched molecular weight (relative abundance: 31.39%) for CCC(C)C

GHS hazard classification: Health Hazard, Irritant

NN-rank 7: ['C=CC(C)C', 'CC(C)CC(=O)O']

NN-rank 8: ['C', 'CCCCOC(=O)CC(C)C']

NN-rank 9: ['C', 'CCCCOC(=O)CC(C)C']

NN-rank 10: ['C', 'CCCC(=O)OCCC(C)C']

NN-rank 11: ['C', 'CCCC(=O)OCCC(C)C']

NN-rank 12: ['CC(C)=CC(=O)OCCC(C)C']

NN-rank 13: ['C=CC(C)C', 'CC(C)CC(=O)O']

NN-rank 14: ['CC(C)CCOCCC(C)C', 'O']

NN/MS matches corresponding with tox GHS classification highlight in Bold font.

NN-rank 15: ['C', 'CC(C)C', 'CC(C)CC(=O)O']

EI-MS matched molecular weight (relative abundance: 37.69%) for CC(C)C

GHS hazard classification: none of the hazards selected for correlation

NN-rank 16: ['CC(C)CCC(C(=O)O)C(C)C']

NN-rank 17: ['C', 'CC(C)C', 'CC(C)CC(=O)O']

EI-MS matched molecular weight (relative abundance: 37.69%) for CC(C)C

GHS hazard classification: none of the hazards selected for correlation

NN-rank 18: ['CC(C)CCC(C(=O)O)C(C)C']

NN-rank 19: ['CCC', 'CCOC(=O)CC(C)C']

EI-MS matched molecular weight (relative abundance: 45.19%) for CCC

GHS hazard classification: none of the hazards selected for correlation

NN-rank 20: ['CC(C)CCO', 'CCC(C)C', 'O']

EI-MS matched molecular weight (relative abundance: 31.39%) for CCC(C)C

GHS hazard classification: Health Hazard, Irritant

NN-rank 21: ['CC(C)C=COC(=O)CC(C)C']

NN-rank 22: ['CC(C)C=CO', 'CC(C)CCO']

EI-MS matched molecular weight (relative abundance: 56.69%) for CC(C)C=CO

GHS hazard classification: none of the hazards selected for correlation

NN-rank 23: ['CC(C)C', 'CC(C)C(C)C(=O)O']

EI-MS matched molecular weight (relative abundance: 37.69%) for CC(C)C

GHS hazard classification: none of the hazards selected for correlation

NN-rank 24: ['CC(=O)OCCC(C)C', 'CCC']

EI-MS matched molecular weight (relative abundance: 45.19%) for CCC

GHS hazard classification: none of the hazards selected for correlation

NN-rank 25: ['CC(C)C=C=O', 'CC(C)CCO']

NN/MS matches corresponding with tox GHS classification highlight in Bold font.

137. Isoamyl Phenyl Acetate: CC(C)CCOC(=O)CC1=CC=CC=C1

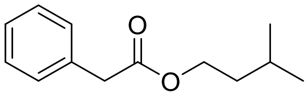

Summary Report of GHS Classification for NN/MS Matches:

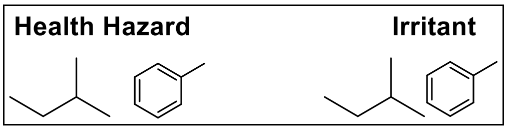

NN-rank 1: ['CCC(C)C', 'O=C(O)Cc1ccccc1']

EI-MS matched molecular weight (relative abundance: 15.01%) for CCC(C)C

GHS hazard classification: Health Hazard, Irritant

NN-rank 2: ['CCC(C)C', 'O=C(O)Cc1ccccc1']

EI-MS matched molecular weight (relative abundance: 15.01%) for CCC(C)C

GHS hazard classification: Health Hazard, Irritant

NN-rank 3: ['CC(C)CCO', 'OCCc1ccccc1']

NN-rank 4: ['CC(C)C', 'COC(=O)Cc1ccccc1']

NN-rank 5: ['CC(C)CCO', 'O=CCc1ccccc1']

NN-rank 6: ['CCC(C)C', 'O', 'O=CCc1ccccc1']

EI-MS matched molecular weight (relative abundance: 15.01%) for CCC(C)C

GHS hazard classification: Health Hazard, Irritant

NN-rank 7: ['CC(C)CCOCCc1ccccc1', 'O']

NN-rank 8: ['C', 'CCCCOC(=O)Cc1ccccc1']

NN-rank 9: ['C', 'CCCCOC(=O)Cc1ccccc1']

NN-rank 10: ['C=CC(C)C', 'O=C(O)Cc1ccccc1']

NN-rank 11: ['CC(C)CCc1ccccc1CC(=O)O']

NN-rank 12: ['CC(C)CCc1ccccc1CC(=O)O']

NN-rank 13: ['CC(C)CCO', 'CCc1ccccc1', 'O']

NN-rank 14: ['CC(C)CCOC=O', 'Cc1ccccc1']

EI-MS matched molecular weight (relative abundance: 99.99%) for Cc1ccccc1

GHS hazard classification: Health Hazard, Irritant

NN-rank 15: ['CC(=O)OCCC(C)C', 'c1ccccc1']

NN-rank 16: ['CCC(C)C', 'O', 'O=C1Cc2ccccc21']

EI-MS matched molecular weight (relative abundance: 15.01%) for CCC(C)C

NN/MS matches corresponding with tox GHS classification highlight in Bold font.

GHS hazard classification: Health Hazard, Irritant

NN-rank 17: ['C=CC(C)C', 'O=C(O)Cc1ccccc1']

NN-rank 18: ['CCC', 'CCOC(=O)Cc1ccccc1']

EI-MS matched molecular weight (relative abundance: 47.34%) for CCC

GHS hazard classification: none of the hazards selected for correlation

NN-rank 19: ['C', 'CC(C)C', 'O=C(O)Cc1ccccc1']

NN-rank 20: ['C', 'CC(C)C', 'O=C(O)Cc1ccccc1']

NN-rank 21: ['CC(C)C=COC(=O)Cc1ccccc1']

NN-rank 22: ['CC(C)CCc1ccccc1CC(=O)O']

NN-rank 23: ['CC(C)CCc1ccccc1CC(=O)O']

NN-rank 24: ['C', 'C', 'CCCOC(=O)Cc1ccccc1']

NN-rank 25: ['CC(C)C', 'O=C1Cc2ccccc2CO1']

NN/MS matches corresponding with tox GHS classification highlight in Bold font.

138. Isobutyl Acetate: CC(C)COC(=O)C

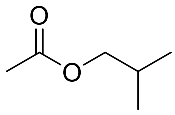

Summary Report of GHS Classification for NN/MS Matches:

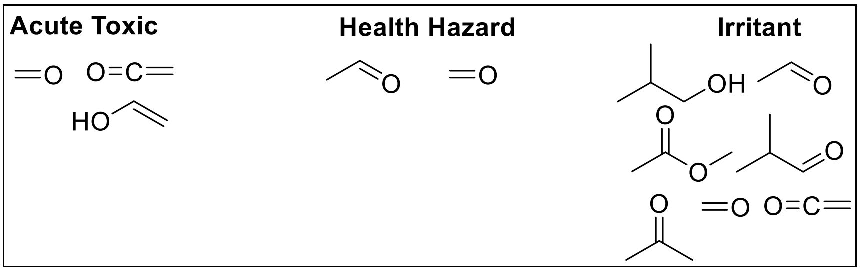

NN-rank 1: ['CC(C)CO', 'CC=O']

EI-MS matched molecular weight (relative abundance: 33.23%) for CC(C)CO

GHS hazard classification: Irritant

EI-MS matched molecular weight (relative abundance: 99.99%) for CC=O

GHS hazard classification: Health Hazard, Irritant

NN-rank 2: ['CCC', 'COC(C)=O']

EI-MS matched molecular weight (relative abundance: 99.99%) for CCC

GHS hazard classification: none of the hazards selected for correlation

EI-MS matched molecular weight (relative abundance: 33.23%) for COC(C)=O

GHS hazard classification: Irritant

NN-rank 3: ['CC(C)C', 'CC(=O)O']

EI-MS matched molecular weight (relative abundance: 6.41%) for CC(C)C

GHS hazard classification: none of the hazards selected for correlation

NN-rank 4: ['C', 'CCCOC(C)=O']

EI-MS matched molecular weight (relative abundance: 7.21%) for C

GHS hazard classification: none of the hazards selected for correlation

NN-rank 5: ['C', 'CCCOC(C)=O']

EI-MS matched molecular weight (relative abundance: 7.21%) for C

GHS hazard classification: none of the hazards selected for correlation

NN-rank 6: ['CC(C)CO', 'CCO']

EI-MS matched molecular weight (relative abundance: 33.23%) for CC(C)CO

GHS hazard classification: Irritant

NN/MS matches corresponding with tox GHS classification highlight in Bold font.

NN-rank 7: ['C=C=O', 'CC(C)CO']

EI-MS matched molecular weight (relative abundance: 22.52%) for C=C=O

GHS hazard classification: Acute Toxic, Irritant

EI-MS matched molecular weight (relative abundance: 33.23%) for CC(C)CO

GHS hazard classification: Irritant

NN-rank 8: ['CC(C)C=O', 'CC=O']

EI-MS matched molecular weight (relative abundance: 6.41%) for CC(C)C=O

GHS hazard classification: Irritant

EI-MS matched molecular weight (relative abundance: 99.99%) for CC=O

GHS hazard classification: Health Hazard, Irritant

NN-rank 9: ['C=C(C)COC(C)=O']

NN-rank 10: ['C=C(C)COC(C)=O']

NN-rank 11: ['CC(C)COC(C)O']

NN-rank 12: ['C', 'CC(C)COC=O']

EI-MS matched molecular weight (relative abundance: 7.21%) for C

GHS hazard classification: none of the hazards selected for correlation

NN-rank 13: ['CC(=O)CC(C)CO']

NN-rank 14: ['CC(=O)CC(C)CO']

NN-rank 15: ['C', 'C=O', 'CC(C)CO']

EI-MS matched molecular weight (relative abundance: 7.21%) for C

GHS hazard classification: none of the hazards selected for correlation

EI-MS matched molecular weight (relative abundance: 10.41%) for C=O

GHS hazard classification: Acute Toxic, Health Hazard, Irritant

EI-MS matched molecular weight (relative abundance: 33.23%) for CC(C)CO

GHS hazard classification: Irritant

NN-rank 16: ['C', 'C', 'CCOC(C)=O']

EI-MS matched molecular weight (relative abundance: 7.21%) for C

GHS hazard classification: none of the hazards selected for correlation

EI-MS matched molecular weight (relative abundance: 7.21%) for C

GHS hazard classification: none of the hazards selected for correlation

NN-rank 17: ['CC(=O)OCC1CC1']

NN-rank 18: ['C', 'C=CCOC(C)=O']

EI-MS matched molecular weight (relative abundance: 7.21%) for C

GHS hazard classification: none of the hazards selected for correlation

NN-rank 19: ['C', 'C=CCOC(C)=O']

NN/MS matches corresponding with tox GHS classification highlight in Bold font.

EI-MS matched molecular weight (relative abundance: 7.21%) for C

GHS hazard classification: none of the hazards selected for correlation

NN-rank 20: ['CC(C)=O', 'CCCO']

EI-MS matched molecular weight (relative abundance: 6.41%) for CC(C)=O

GHS hazard classification: Irritant

NN-rank 21: ['CC(C)=O', 'CCCO']

EI-MS matched molecular weight (relative abundance: 6.41%) for CC(C)=O

GHS hazard classification: Irritant

NN-rank 22: ['C=CO', 'CC(C)CO']

EI-MS matched molecular weight (relative abundance: 99.99%) for C=CO

GHS hazard classification: Acute Toxic

EI-MS matched molecular weight (relative abundance: 33.23%) for CC(C)CO

GHS hazard classification: Irritant

NN-rank 23: ['C=O', 'CC=O', 'CCC']

EI-MS matched molecular weight (relative abundance: 10.41%) for C=O

GHS hazard classification: Acute Toxic, Health Hazard, Irritant

EI-MS matched molecular weight (relative abundance: 99.99%) for CC=O

GHS hazard classification: Health Hazard, Irritant

EI-MS matched molecular weight (relative abundance: 99.99%) for CCC

GHS hazard classification: none of the hazards selected for correlation

NN-rank 24: ['C', 'CC(=O)O', 'CCC']

EI-MS matched molecular weight (relative abundance: 7.21%) for C

GHS hazard classification: none of the hazards selected for correlation

EI-MS matched molecular weight (relative abundance: 99.99%) for CCC

GHS hazard classification: none of the hazards selected for correlation

NN-rank 25: ['C', 'CC(C)CO', 'CO']

EI-MS matched molecular weight (relative abundance: 7.21%) for C

GHS hazard classification: none of the hazards selected for correlation

EI-MS matched molecular weight (relative abundance: 33.23%) for CC(C)CO

GHS hazard classification: Irritant

NN/MS matches corresponding with tox GHS classification highlight in Bold font.

139. Isobutyl Alcohol: CC(C)CO

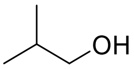

Summary Report of GHS Classification for NN/MS Matches:

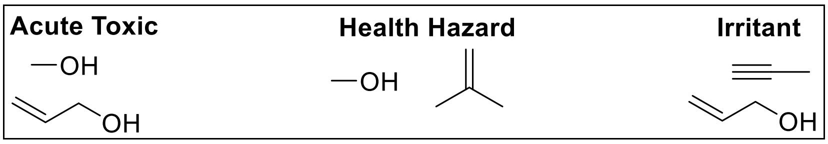

NN-rank 1: ['CC(C)C', 'O']

EI-MS matched molecular weight (relative abundance: 7.9%) for CC(C)C

GHS hazard classification: none of the hazards selected for correlation

NN-rank 2: ['C', 'CCCO']

NN-rank 3: ['C', 'CCCO']

NN-rank 4: ['CC(C)C=O']

NN-rank 5: ['C=C(C)C', 'O']

EI-MS matched molecular weight (relative abundance: 9.9%) for C=C(C)C

GHS hazard classification: Health Hazard

NN-rank 6: ['OCC1CC1']

NN-rank 7: ['CCC', 'CO']

EI-MS matched molecular weight (relative abundance: 99.99%) for CCC

GHS hazard classification: none of the hazards selected for correlation

EI-MS matched molecular weight (relative abundance: 37.6%) for CO

GHS hazard classification: Acute Toxic, Health Hazard

NN-rank 8: ['CC1CC1', 'O']

EI-MS matched molecular weight (relative abundance: 9.9%) for CC1CC1

GHS hazard classification: none of the hazards selected for correlation

NN-rank 9: ['CC1CC1', 'O']

EI-MS matched molecular weight (relative abundance: 9.9%) for CC1CC1

GHS hazard classification: none of the hazards selected for correlation

NN-rank 10: ['C=C(C)CO']

NN-rank 11: ['C=C(C)CO']

NN-rank 12: ['C', 'C', 'CCO']

NN-rank 13: ['CC(C)=CO']

NN-rank 14: ['CC1CC1O']

NN/MS matches corresponding with tox GHS classification highlight in Bold font.

NN-rank 15: ['CC1CC1O']

NN-rank 16: ['C', 'CCC', 'O']

EI-MS matched molecular weight (relative abundance: 99.99%) for CCC

GHS hazard classification: none of the hazards selected for correlation

NN-rank 17: ['C', 'C=CC', 'O']

EI-MS matched molecular weight (relative abundance: 76.89%) for C=CC

GHS hazard classification: none of the hazards selected for correlation

NN-rank 18: ['C', 'C=CC', 'O']

EI-MS matched molecular weight (relative abundance: 76.89%) for C=CC

GHS hazard classification: none of the hazards selected for correlation

NN-rank 19: ['C', 'C#CC', 'O']

EI-MS matched molecular weight (relative abundance: 28.0%) for C#CC

GHS hazard classification: Irritant

NN-rank 20: ['C', 'C#CC', 'O']

EI-MS matched molecular weight (relative abundance: 28.0%) for C#CC

GHS hazard classification: Irritant

NN-rank 21: ['CCCCO']

NN-rank 22: ['CCCCO']

NN-rank 23: ['C', 'CC=CO']

EI-MS matched molecular weight (relative abundance: 7.9%) for CC=CO

GHS hazard classification: none of the hazards selected for correlation

NN-rank 24: ['C', 'CC=CO']

EI-MS matched molecular weight (relative abundance: 7.9%) for CC=CO

GHS hazard classification: none of the hazards selected for correlation

NN-rank 25: ['C', 'C=CCO']

EI-MS matched molecular weight (relative abundance: 7.9%) for C=CCO

GHS hazard classification: Acute Toxic, Irritant

NN/MS matches corresponding with tox GHS classification highlight in Bold font.

140. Isobutyl Butyrate: CCCC(=O)OCC(C)C

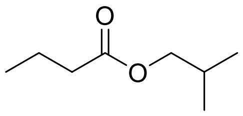

Summary Report of GHS Classification for NN/MS Matches:

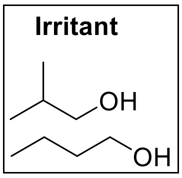

NN-rank 1: ['CC(C)C', 'CCCC(=O)O']

EI-MS matched molecular weight (relative abundance: 14.81%) for CC(C)C

GHS hazard classification: none of the hazards selected for correlation

NN-rank 2: ['CC(C)C', 'CCCC(=O)O']

EI-MS matched molecular weight (relative abundance: 14.81%) for CC(C)C

GHS hazard classification: none of the hazards selected for correlation

NN-rank 3: ['CC(C)C', 'CCCC=O', 'O']

EI-MS matched molecular weight (relative abundance: 14.81%) for CC(C)C

GHS hazard classification: none of the hazards selected for correlation

EI-MS matched molecular weight (relative abundance: 99.99%) for CCCC=O

GHS hazard classification: none of the hazards selected for correlation

NN-rank 4: ['C', 'CCC', 'CCCC(=O)O']

EI-MS matched molecular weight (relative abundance: 27.83%) for CCC

GHS hazard classification: none of the hazards selected for correlation

NN-rank 5: ['CCC', 'CCCC(=O)OC']

EI-MS matched molecular weight (relative abundance: 27.83%) for CCC

GHS hazard classification: none of the hazards selected for correlation

EI-MS matched molecular weight (relative abundance: 7.21%) for CCCC(=O)OC

GHS hazard classification: none of the hazards selected for correlation

NN-rank 6: ['CC(C)CO', 'CCCC=O']

EI-MS matched molecular weight (relative abundance: 5.1%) for CC(C)CO

GHS hazard classification: Irritant

EI-MS matched molecular weight (relative abundance: 99.99%) for CCCC=O

GHS hazard classification: none of the hazards selected for correlation

NN/MS matches corresponding with tox GHS classification highlight in Bold font.

NN-rank 7: ['CC(C)C', 'CCC1OC1=O']

EI-MS matched molecular weight (relative abundance: 14.81%) for CC(C)C

GHS hazard classification: none of the hazards selected for correlation

NN-rank 8: ['CC(C)CO', 'CCCCO']

EI-MS matched molecular weight (relative abundance: 5.1%) for CC(C)CO

GHS hazard classification: Irritant

EI-MS matched molecular weight (relative abundance: 5.1%) for CCCCO

GHS hazard classification: Irritant

NN-rank 9: ['C', 'CCC', 'CCCC(=O)O']

EI-MS matched molecular weight (relative abundance: 27.83%) for CCC

GHS hazard classification: none of the hazards selected for correlation

NN-rank 10: ['C', 'CCCOC(=O)CCC']

NN-rank 11: ['C', 'CCCOC(=O)CCC']

NN-rank 12: ['C', 'CCC(=O)OCC(C)C']

NN-rank 13: ['CCCCOCC(C)C', 'O']

NN-rank 14: ['C', 'CCC', 'CCCC=O', 'O']

EI-MS matched molecular weight (relative abundance: 27.83%) for CCC

GHS hazard classification: none of the hazards selected for correlation

EI-MS matched molecular weight (relative abundance: 99.99%) for CCCC=O

GHS hazard classification: none of the hazards selected for correlation

NN-rank 15: ['CC(C)C', 'CCC(O)C=O']

EI-MS matched molecular weight (relative abundance: 14.81%) for CC(C)C

GHS hazard classification: none of the hazards selected for correlation

NN-rank 16: ['CC1COC(=O)CC(C)C1']

NN-rank 17: ['CC1COC(=O)CC(C)C1']

NN-rank 18: ['CC(C)C', 'CCC', 'O=CO']

EI-MS matched molecular weight (relative abundance: 14.81%) for CC(C)C

GHS hazard classification: none of the hazards selected for correlation

EI-MS matched molecular weight (relative abundance: 27.83%) for CCC

GHS hazard classification: none of the hazards selected for correlation

NN-rank 19: ['CCC(=O)OCC(C)CC']

NN-rank 20: ['CCC(=O)OCC(C)CC']

NN-rank 21: ['CC(C)CO', 'CCCC', 'O']

EI-MS matched molecular weight (relative abundance: 5.1%) for CC(C)CO

GHS hazard classification: Irritant

NN/MS matches corresponding with tox GHS classification highlight in Bold font.

EI-MS matched molecular weight (relative abundance: 14.81%) for CCCC

GHS hazard classification: none of the hazards selected for correlation

NN-rank 22: ['CC1CCCCC(=O)OC1']

NN-rank 23: ['CC1CCCCC(=O)OC1']

NN-rank 24: ['CC', 'CC(C)C', 'O=C1CO1']

EI-MS matched molecular weight (relative abundance: 14.81%) for CC(C)C

GHS hazard classification: none of the hazards selected for correlation

EI-MS matched molecular weight (relative abundance: 14.81%) for O=C1CO1

GHS hazard classification: none of the hazards selected for correlation

NN-rank 25: ['CCCOC(=O)CC(C)C']

NN/MS matches corresponding with tox GHS classification highlight in Bold font.

141. Isobutyric Acid: CC(C)C(=O)O

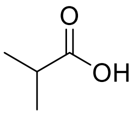

Summary Report of GHS Classification for NN/MS Matches:

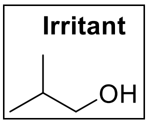

NN-rank 1: ['CC(C)C(=O)O']

NN-rank 2: ['CC1COC1=O']

NN-rank 3: ['CC1COC1=O']

NN-rank 4: ['CC1(C)OC1=O']

NN-rank 5: ['CC(C)CO', 'O']

EI-MS matched molecular weight (relative abundance: 25.89%) for CC(C)CO

GHS hazard classification: Irritant

NN-rank 6: ['C', 'CCC(=O)O']

EI-MS matched molecular weight (relative abundance: 25.89%) for CCC(=O)O

GHS hazard classification: none of the hazards selected for correlation

NN-rank 7: ['C', 'CCC(=O)O']

EI-MS matched molecular weight (relative abundance: 25.89%) for CCC(=O)O

GHS hazard classification: none of the hazards selected for correlation

NN-rank 8: ['CC(C)C=O', 'O']

NN-rank 9: ['CC(C)CO', 'O']

EI-MS matched molecular weight (relative abundance: 25.89%) for CC(C)CO

GHS hazard classification: Irritant

NN-rank 10: ['CC(C)C=O', 'O']

NN-rank 11: ['CC1(C)OC1=O']

NN-rank 12: ['CCC', 'O=CO']

EI-MS matched molecular weight (relative abundance: 99.99%) for CCC

GHS hazard classification: none of the hazards selected for correlation

EI-MS matched molecular weight (relative abundance: 7.69%) for O=CO

GHS hazard classification: none of the hazards selected for correlation

NN-rank 13: ['CCC', 'O=CO']

NN/MS matches corresponding with tox GHS classification highlight in Bold font.

EI-MS matched molecular weight (relative abundance: 99.99%) for CCC

GHS hazard classification: none of the hazards selected for correlation

EI-MS matched molecular weight (relative abundance: 7.69%) for O=CO

GHS hazard classification: none of the hazards selected for correlation

NN-rank 14: ['CC1COC1=O']

NN-rank 15: ['CC1COC1=O']

NN-rank 16: ['C=C(C)C(=O)O']

NN-rank 17: ['CC(C)C(=O)O']

NN-rank 18: ['CC(C=O)CO']

NN-rank 19: ['CC(C=O)CO']

NN-rank 20: ['CC(C)(O)C=O']

NN-rank 21: ['CC1COC1', 'O']

NN-rank 22: ['CC1COC1', 'O']

NN-rank 23: ['CC(CO)CO']

NN-rank 24: ['CC(CO)CO']

NN-rank 25: ['CC(C)C(=O)O']

NN/MS matches corresponding with tox GHS classification highlight in Bold font.

142. Isovaleraldehyde: CC(C)CC=O

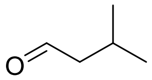

Summary Report of GHS Classification for NN/MS Matches:

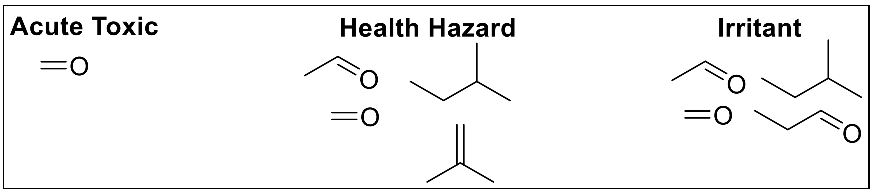

NN-rank 1: ['CC(C)CCO']

NN-rank 2: ['C', 'CCCC=O']

EI-MS matched molecular weight (relative abundance: 35.99%) for CCCC=O

GHS hazard classification: none of the hazards selected for correlation

NN-rank 3: ['C', 'CCCC=O']

EI-MS matched molecular weight (relative abundance: 35.99%) for CCCC=O

GHS hazard classification: none of the hazards selected for correlation

NN-rank 4: ['C=O', 'CC(C)C']

EI-MS matched molecular weight (relative abundance: 46.29%) for C=O

GHS hazard classification: Acute Toxic, Health Hazard, Irritant

EI-MS matched molecular weight (relative abundance: 36.99%) for CC(C)C

GHS hazard classification: none of the hazards selected for correlation

NN-rank 5: ['CC(C)C=C=O']

NN-rank 6: ['CC(C)=CC=O']

NN-rank 7: ['C=C(C)CC=O']

NN-rank 8: ['C=C(C)CC=O']

NN-rank 9: ['C', 'C', 'CCC=O']

EI-MS matched molecular weight (relative abundance: 36.99%) for CCC=O

GHS hazard classification: Irritant

NN-rank 10: ['C=CC(C)C', 'O']

NN-rank 11: ['CC=O', 'CCC']

EI-MS matched molecular weight (relative abundance: 93.39%) for CC=O

GHS hazard classification: Health Hazard, Irritant

EI-MS matched molecular weight (relative abundance: 93.39%) for CCC

GHS hazard classification: none of the hazards selected for correlation

NN/MS matches corresponding with tox GHS classification highlight in Bold font.

NN-rank 12: ['CC(C)C=CO']

NN-rank 13: ['CCC(C)C', 'O']

EI-MS matched molecular weight (relative abundance: 35.99%) for CCC(C)C

GHS hazard classification: Health Hazard, Irritant

NN-rank 14: ['C=O', 'C=C(C)C']

EI-MS matched molecular weight (relative abundance: 46.29%) for C=O

GHS hazard classification: Acute Toxic, Health Hazard, Irritant

EI-MS matched molecular weight (relative abundance: 6.19%) for C=C(C)C

GHS hazard classification: Health Hazard

NN-rank 15: ['CC(C)C', 'CO']

EI-MS matched molecular weight (relative abundance: 36.99%) for CC(C)C

GHS hazard classification: none of the hazards selected for correlation

NN-rank 16: ['O=CCC1CC1']

NN-rank 17: ['C', 'CC=CC=O']

NN-rank 18: ['C', 'CC=CC=O']

NN-rank 19: ['CC1(C)CCO1']

NN-rank 20: ['C', 'C=CCC=O']

NN-rank 21: ['C', 'C=CCC=O']

NN-rank 22: ['CC1CC1C=O']

NN-rank 23: ['C=O', 'CC1CC1']

EI-MS matched molecular weight (relative abundance: 46.29%) for C=O

GHS hazard classification: Acute Toxic, Health Hazard, Irritant

EI-MS matched molecular weight (relative abundance: 6.19%) for CC1CC1

GHS hazard classification: none of the hazards selected for correlation

NN-rank 24: ['CC1CCOC1']

NN-rank 25: ['CC1CCOC1']

NN/MS matches corresponding with tox GHS classification highlight in Bold font.

143. L-Carvone: CC1=CC[C@H](CC1=O)C(=C)C

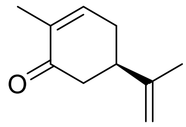

NN-rank 1: ['CC1=CC[C](C(C)C)CC1=O']

EI-MS matched molecular weight (relative abundance: 8.69%) for CC1=CC[C](C(C)C)CC1=O

GHS hazard classification: unknown

NN-rank 2: ['C=C(C)C1=CC(=O)C(C)=CC1']

NN-rank 3: ['CC1=CCC(=C(C)C)CC1=O']

NN-rank 4: ['C=C(C)[C]1CCC(C)C(=O)C1']

EI-MS matched molecular weight (relative abundance: 8.69%) for C=C(C)[C]1CCC(C)C(=O)C1

GHS hazard classification: unknown

NN-rank 5: ['C', 'C=C(C)[C]1CC=CC(=O)C1']

NN-rank 6: ['C', 'C=C(C)[C]1CCCC(=O)C1']

NN-rank 7: ['C=C(C)[C]1CC=C(C)C(O)C1']

EI-MS matched molecular weight (relative abundance: 8.69%) for C=C(C)[C]1CC=C(C)C(O)C1

GHS hazard classification: unknown

NN-rank 8: ['C=C(C)C1=CC=C(C)C(=O)C1']

NN-rank 9: ['C=C1CC2=C(C)C(=O)C[C]1C2']

NN-rank 10: ['C=C(C)[C]1CC=C(C)C(=O)C1']

NN-rank 11: ['C=C(C)[C](CC)CC(=O)CC']

NN-rank 12: ['C=C(C)[C]CC(=O)C(C)=CC']

NN-rank 13: ['C=C1CC2C[C]1CC(=O)C2C']

NN-rank 14: ['CC1=CC2=C(C)C(=O)C[C]1C2']

NN-rank 15: ['CC1=CC[C]CC1=O', 'C=CC']

EI-MS matched molecular weight (relative abundance: 17.19%) for CC1=CC[C]CC1=O

GHS hazard classification: unknown

EI-MS matched molecular weight (relative abundance: 17.79%) for C=CC

GHS hazard classification: none of the hazards selected for correlation

NN-rank 16: ['CC1=C2C[C](CC1=O)C(C)C2']

NN-rank 17: ['CC=C(C)C(=O)C[C]=C(C)C']

NN/MS matches corresponding with tox GHS classification highlight in Bold font.

EI-MS matched molecular weight (relative abundance: 8.69%) for CC=C(C)C(=O)C[C]=C(C)C

GHS hazard classification: unknown

NN-rank 18: ['C=C(C)[C]=CC(=O)C(C)=CC']

NN-rank 19: ['C=C(C)[C]1C=C(O)C(C)=CC1']

NN-rank 20: ['CC1=CC[C]CC1=O', 'CCC']

EI-MS matched molecular weight (relative abundance: 17.19%) for CC1=CC[C]CC1=O

GHS hazard classification: unknown

NN-rank 21: ['CC1=CC[C]=CC1=O', 'C=CC']

EI-MS matched molecular weight (relative abundance: 12.79%) for CC1=CC[C]=CC1=O

GHS hazard classification: unknown

EI-MS matched molecular weight (relative abundance: 17.79%) for C=CC

GHS hazard classification: none of the hazards selected for correlation

NN-rank 22: ['C=C(C)[C]CC=C(C)C(C)=O']

NN-rank 23: ['C=C(C)[C](CC=O)CC=CC']

EI-MS matched molecular weight (relative abundance: 8.69%) for C=C(C)[C](CC=O)CC=CC

GHS hazard classification: unknown

NN-rank 24: ['C', 'C=C1CC2C[C]1CC(=O)C2']

NN-rank 25: ['CC1CC2C[C]1CC(=O)C2C']

EI-MS matched molecular weight (relative abundance: 8.69%) for CC1CC2C[C]1CC(=O)C2C

GHS hazard classification: unknown

NN/MS matches corresponding with tox GHS classification highlight in Bold font.

144. Lactic Acid: CC(C(=O)O)O

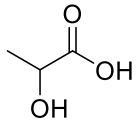

NN-rank 1: ['CCC(=O)O', 'O']

NN-rank 2: ['CC(O)C(=O)O']

NN-rank 3: ['C', 'O=C(O)CO']

NN-rank 4: ['C#CC(=O)O', 'O']

NN-rank 5: ['CCO', 'O=CO']

> **EI-MS matched molecular weight (relative abundance: 99.99%) for CCO**
>
> **GHS hazard classification: none of the hazards selected for correlation**
>
> **EI-MS matched molecular weight (relative abundance: 99.99%) for O=CO**
>
> **GHS hazard classification: none of the hazards selected for correlation**

NN-rank 6: ['C', 'CC(=O)O', 'O']

NN-rank 7: ['O=C1OCC1O']

NN-rank 8: ['CC(O)CO', 'O']

NN-rank 9: ['CC1OC1=O', 'O']

NN-rank 10: ['C=CC(=O)O', 'O']

NN-rank 11: ['C=C(O)C(=O)O']

NN-rank 12: ['CC(=O)C(=O)O']

NN-rank 13: ['O=C(O)C1CO1']

NN-rank 14: ['CC(O)CO', 'O']

NN-rank 15: ['CC(O)C=O', 'O']

NN-rank 16: ['CC(O)C=O', 'O']

NN-rank 17: ['CCO', 'O=CO']

> **EI-MS matched molecular weight (relative abundance: 99.99%) for CCO**
>
> **GHS hazard classification: none of the hazards selected for correlation**
>
> **EI-MS matched molecular weight (relative abundance: 99.99%) for O=CO**
>
> **GHS hazard classification: none of the hazards selected for correlation**

NN-rank 18: ['CC1(O)OC1=O']

NN-rank 19: ['O=C(O)CCO']

NN-rank 20: ['CC1OC1=O', 'O']

NN-rank 21: ['CC1OC1=O', 'O']

NN-rank 22: ['CC(O)C(O)O']

NN/MS matches corresponding with tox GHS classification highlight in Bold font.

NN-rank 23: ['C#CO', 'O=CO']

EI-MS matched molecular weight (relative abundance: 99.99%) for O=CO

GHS hazard classification: none of the hazards selected for correlation

NN-rank 24: ['C', 'O=CC(=O)O']

NN-rank 25: ['OCC(O)CO']

NN/MS matches corresponding with tox GHS classification highlight in Bold font.

145. Levulinic Acid: CC(=O)CCC(=O)O

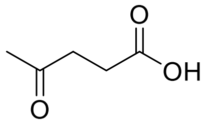

Summary Report of GHS Classification for NN/MS Matches:

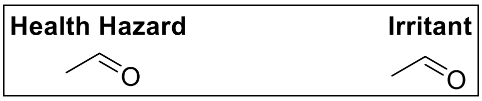

NN-rank 1: ['CCCCC(=O)O', 'O']

NN-rank 2: ['CC(C)=O', 'CC(=O)O']

NN-rank 3: ['CC(O)CCC(=O)O']

NN-rank 4: ['O', 'O=C1CCC(=O)C1']

NN-rank 5: ['CC=CCC(=O)O', 'O']

NN-rank 6: ['CC(=O)CCCO', 'O']

NN-rank 7: ['C', 'CCCC(=O)O', 'O']

EI-MS matched molecular weight (relative abundance: 5.0%) for C

GHS hazard classification: none of the hazards selected for correlation

NN-rank 8: ['CC=O', 'CCC(=O)O']

EI-MS matched molecular weight (relative abundance: 99.99%) for CC=O

GHS hazard classification: Health Hazard, Irritant

EI-MS matched molecular weight (relative abundance: 6.91%) for CCC(=O)O

GHS hazard classification: none of the hazards selected for correlation

NN-rank 9: ['C=CCCC(=O)O', 'O']

NN-rank 10: ['O', 'O=C1CCC(=O)C1']

NN-rank 11: ['CCCCC(=O)O', 'O']

NN-rank 12: ['C', 'O=C(O)CCCO']

EI-MS matched molecular weight (relative abundance: 5.0%) for C

GHS hazard classification: none of the hazards selected for correlation

NN-rank 13: ['CCC(C)=O', 'O=CO']

EI-MS matched molecular weight (relative abundance: 8.31%) for O=CO

GHS hazard classification: none of the hazards selected for correlation

NN-rank 14: ['O=C(O)CCCCO']

NN-rank 15: ['C', 'O=CCCC(=O)O']

EI-MS matched molecular weight (relative abundance: 5.0%) for C

NN/MS matches corresponding with tox GHS classification highlight in Bold font.

GHS hazard classification: none of the hazards selected for correlation

NN-rank 16: ['CC(=O)CCC(=O)O']

NN-rank 17: ['CC(=O)C1CC1=O', 'O']

NN-rank 18: ['C=C(O)CCC(=O)O']

NN-rank 19: ['CC1CCC(=O)O1', 'O']

NN-rank 20: ['C', 'C=CCC(=O)O', 'O']

EI-MS matched molecular weight (relative abundance: 5.0%) for C

GHS hazard classification: none of the hazards selected for correlation

NN-rank 21: ['CCCCC(=O)O', 'O']

NN-rank 22: ['CC(=O)CCCO', 'O']

NN-rank 23: ['CCCC(C)=O', 'O', 'O']

NN-rank 24: ['CC(=O)O', 'C=CC', 'O']

NN-rank 25: ['CC(=O)CCC=O', 'O']

NN/MS matches corresponding with tox GHS classification highlight in Bold font.

146. Limonene: CC1=CCC(CC1)C(=C)C

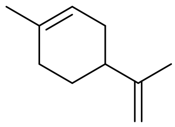

NN-rank 1: ['C', 'C=C(C)C1C=CCCC1']

EI-MS matched molecular weight (relative abundance: 12.53%) for C=C(C)C1C=CCCC1

GHS hazard classification: none of the hazards selected for correlation

NN-rank 2: ['C', 'C=C(C)C1CCCCC1']

NN-rank 3: ['C=C(C)C1C=CC(C)CC1']

NN-rank 4: ['C=C(C)C1CCC(C)CC1']

NN-rank 5: ['C=C(C)C1CC=C(C)CC1']

NN-rank 6: ['C=C1CCC(C(=C)C)CC1']

NN-rank 7: ['C', 'C=C(C)C1CC=CCC1']

EI-MS matched molecular weight (relative abundance: 12.53%) for C=C(C)C1CC=CCC1

GHS hazard classification: none of the hazards selected for correlation

NN-rank 8: ['C', 'C=C(C)C1CC=CCC1']

EI-MS matched molecular weight (relative abundance: 12.53%) for C=C(C)C1CC=CCC1

GHS hazard classification: none of the hazards selected for correlation

NN-rank 9: ['C', 'C=C(C)C1=C=CCCC1']

NN-rank 10: ['C=C1C=CC(C(=C)C)CC1']

NN-rank 11: ['C=C(C)C1C=CC(C)=CC1']

NN-rank 12: ['C=C(C)C1=CC=C(C)CC1']

NN-rank 13: ['C=C(C)C(CC)CCCC']

NN-rank 14: ['C', 'C=C(C)C12CCCC1C2']

EI-MS matched molecular weight (relative abundance: 12.53%) for C=C(C)C12CCCC1C2

GHS hazard classification: none of the hazards selected for correlation

NN-rank 15: ['C=C(C)C1=C=CC(C)CC1']

NN-rank 16: ['C=C(C)C1C=C=C(C)CC1']

NN-rank 17: ['C', 'C=C(C)C1C=CC=CC1']

NN-rank 18: ['C=C(C)C1=CCC(C)=CC1']

NN/MS matches corresponding with tox GHS classification highlight in Bold font.

NN-rank 19: ['C=C(C)C(CC)CCCC']
NN-rank 20: ['C=C(C)C12CCC(C)C1C2']
NN-rank 21: ['C=C(C)C(C=CCC)CC']
NN-rank 22: ['CC1=CCC(C(C)C)CC1']
NN-rank 23: ['C', 'C=C(C)C12C=C1CCC2']
NN-rank 24: ['C', 'C=C(C)C1CC=C=CC1']
NN-rank 25: ['C=C(C)C12CCC(C)=C1C2']

NN/MS matches corresponding with tox GHS classification highlight in Bold font.

147. Linalool: CC(=CCCC(C)(C=C)O)C

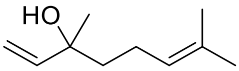

Summary Report of GHS Classification for NN/MS Matches:

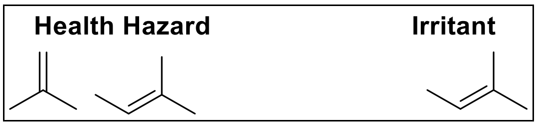

NN-rank 1: ['C=CC1(C)CCCC(C)(C)O1']

NN-rank 2: ['C=CC(C)(O)CCCC(C)C']

NN-rank 3: ['C', 'C=CC1(C)CCC=C(C)O1']

NN-rank 4: ['C', 'C=CC1(C)CCC=C(C)O1']

NN-rank 5: ['C=CC(C)(O)CCCC(=C)C']

NN-rank 6: ['C=CC(C)(O)CCCC(=C)C']

NN-rank 7: ['C=CC1(C)CC=CC(C)(C)O1']

NN-rank 8: ['C', 'C=CC(C)(O)CCCCC']

NN-rank 9: ['C', 'C=CC(C)(O)CCCCC']

NN-rank 10: ['C', 'C', 'C=CC1(C)CCC=CO1']

NN-rank 11: ['C=CC(C)(O)CC=CC(C)C']

NN-rank 12: ['C', 'C=CC1(C)CCCC(C)O1']

NN-rank 13: ['C', 'C=CC1(C)CCCC(C)O1']

NN-rank 14: ['C', 'C=CC(C)(O)CCC=CC']

NN-rank 15: ['C', 'C=CC(C)(O)CCC=CC']

NN-rank 16: ['C', 'C', 'C=CC1(C)CCCCO1']

NN-rank 17: ['C', 'C', 'C=CC(C)(O)CCCC']

NN-rank 18: ['C=CC(C)(C)O', 'CC=C(C)C']

EI-MS matched molecular weight (relative abundance: 42.64%) for CC=C(C)C

GHS hazard classification: Health Hazard, Irritant

NN-rank 19: ['C=C(C)C', 'C=CC(C)(O)CC']

EI-MS matched molecular weight (relative abundance: 63.76%) for C=C(C)C

GHS hazard classification: Health Hazard

NN-rank 20: ['C=CC(C)(CC)OC(C)(C)C']

NN-rank 21: ['C', 'C=CC(C)(O)CC=CCC']

NN-rank 22: ['C', 'C=CC(C)(O)CC=CCC']

NN/MS matches corresponding with tox GHS classification highlight in Bold font.

NN-rank 23: ['C', 'C', 'C=CCCC(C)(O)C=C']

NN-rank 24: ['C=CC(C)(CCC)OC(C)C']

NN-rank 25: ['CCC(C)(O)CCC=C(C)C']

NN/MS matches corresponding with tox GHS classification highlight in Bold font.

148. Linalyl Acetate: CC(=CCCC(C)(C=C)OC(=O)C)C

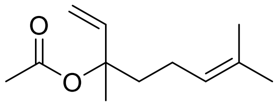

Summary Report of GHS Classification for NN/MS Matches:

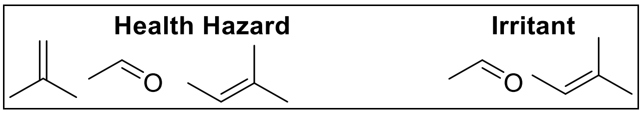

NN-rank 1: ['C=CC(C)(O)CCC=C(C)C', 'CC=O']

EI-MS matched molecular weight (relative abundance: 56.65%) for CC=O

GHS hazard classification: Health Hazard, Irritant

NN-rank 2: ['C=CC(C)CCC=C(C)C', 'CC(=O)O']

NN-rank 3: ['C', 'C=CC(C)(CCC=CC)OC(C)=O']

NN-rank 4: ['C', 'C=CC(C)(CCC=CC)OC(C)=O']

NN-rank 5: ['C=C(C)C', 'C=CC(C)(CC)OC(C)=O']

EI-MS matched molecular weight (relative abundance: 18.22%) for C=C(C)C

GHS hazard classification: Health Hazard

NN-rank 6: ['C=C=C(C)CCC=C(C)C', 'CC(=O)O']

NN-rank 7: ['C=CC(C)(CCCC(C)C)OC(C)=O']

NN-rank 8: ['C', 'C=CC1(C)CCC=C(C)O1', 'CC=O']

EI-MS matched molecular weight (relative abundance: 56.65%) for CC=O

GHS hazard classification: Health Hazard, Irritant

NN-rank 9: ['C', 'C=CC1(C)CCC=C(C)O1', 'CC=O']

EI-MS matched molecular weight (relative abundance: 56.65%) for CC=O

GHS hazard classification: Health Hazard, Irritant

NN-rank 10: ['C', 'C', 'C=CCCC(C)(C=C)OC(C)=O']

NN-rank 11: ['C', 'CC(=O)OC(C)(C)CCC=C(C)C']

NN-rank 12: ['C=CC(C)(C)OC(C)=O', 'CC=C(C)C']

EI-MS matched molecular weight (relative abundance: 26.32%) for CC=C(C)C

GHS hazard classification: Health Hazard, Irritant

NN-rank 13: ['C', 'C=CC(CCC=C(C)C)OC(C)=O']

NN-rank 14: ['C', 'C=CC(C)(CCCCC)OC(C)=O']

NN-rank 15: ['C', 'C=CC(C)(CCCCC)OC(C)=O']

NN/MS matches corresponding with tox GHS classification highlight in Bold font.

NN-rank 16: ['CCC', 'C=CC(C)(CCC)OC(C)=O']

EI-MS matched molecular weight (relative abundance: 56.65%) for CCC

GHS hazard classification: none of the hazards selected for correlation

NN-rank 17: ['C=C', 'CC(=O)OC(C)CCC=C(C)C']

NN-rank 18: ['C=CC(C)(CCCC(=C)C)OC(C)=O']

NN-rank 19: ['C=CC1(C)CCCC(C)(C)O1', 'CC=O']

EI-MS matched molecular weight (relative abundance: 56.65%) for CC=O

GHS hazard classification: Health Hazard, Irritant

NN-rank 20: ['C', 'CC(C)=CCCC1(C)C=CC(=O)O1']

NN-rank 21: ['C', 'C=CCCCC=C(C)C', 'CC(=O)O']

NN-rank 22: ['C', 'C', 'C=CC(C)(CCCC)OC(C)=O']

NN-rank 23: ['C', 'C=CC(C)CCC=C(C)OC(C)=O']

NN-rank 24: ['C', 'C=CC(C)CCC=C(C)OC(C)=O']

NN-rank 25: ['CCC(C)(CCC=C(C)C)OC(C)=O']

NN/MS matches corresponding with tox GHS classification highlight in Bold font.

149. Maltol: CC1=C(C(=O)C=CO1)O

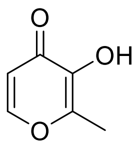

NN-rank 1: ['Cc1cc(=O)cco1', 'O']

NN-rank 2: ['C', 'O=c1ccocc1O']

EI-MS matched molecular weight (relative abundance: 8.29%) for C

GHS hazard classification: none of the hazards selected for correlation

NN-rank 3: [No prediction]

NN-rank 4: [No prediction]

NN-rank 5: ['O=C1C2=COC(=C1O)C2']

NN-rank 6: ['O=c1cc2oc(c1O)C2']

NN-rank 7: ['CC1=C2OC(=CC2=O)O1']

NN-rank 8: ['Cc1cc(=O)cc(O)o1']

NN-rank 9: ['C', 'O=c1ccoc2c1O2']

EI-MS matched molecular weight (relative abundance: 8.29%) for C

GHS hazard classification: none of the hazards selected for correlation

NN-rank 10: ['Cc1cocc(O)c1=O']

NN-rank 11: ['C', 'O=c1ccoc(O)c1']

EI-MS matched molecular weight (relative abundance: 8.29%) for C

GHS hazard classification: none of the hazards selected for correlation

NN-rank 12: ['Cc1cc(=O)c(O)co1']

NN-rank 13: [No prediction]

NN-rank 14: ['Cc1cc(=O)cco1', 'O']

NN-rank 15: [No prediction]

NN-rank 16: [No prediction]

NN-rank 17: [No prediction**]**

EI-MS matched molecular weight (relative abundance: 8.29%) for C

GHS hazard classification: none of the hazards selected for correlation

NN-rank 18: ['CC1=C(O)C2(O)C=C2O1']

NN-rank 19: [No prediction**]**

EI-MS matched molecular weight (relative abundance: 8.29%) for C

GHS hazard classification: none of the hazards selected for correlation

NN/MS matches corresponding with tox GHS classification highlight in Bold font.

NN-rank 20: ['O=C1C(O)=C2OC3=C1C23']

NN-rank 21: ['Cc1coc2c(c1=O)O2']

NN-rank 22: ['Cc1cc(=O)c2c(o1)O2']

NN-rank 23: ['Cc1coc(O)cc1=O']

NN-rank 24: ['O=c1c(O)coc2c1C2']

NN-rank 25: ['C', 'O=c1cc2oc(c1)o2']

EI-MS matched molecular weight (relative abundance: 8.29%) for C

GHS hazard classification: none of the hazards selected for correlation

NN/MS matches corresponding with tox GHS classification highlight in Bold font.

150. Menthol: CC1CCC(C(C1)O)C(C)C

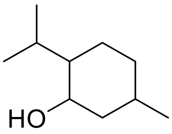

Summary Report of GHS Classification for NN/MS Matches:

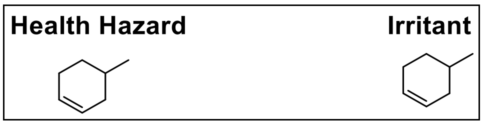

NN-rank 1: ['CC1CCC(C(C)C)C(=O)C1']

NN-rank 2: ['CC1CCC(C(C)C)CC1', 'O']

NN-rank 3: ['CC1CC=C(C(C)C)CC1', 'O']

NN-rank 4: ['C', 'CCC1CCC(C)CC1O']

NN-rank 5: ['C', 'CCC1CCC(C)CC1O']

NN-rank 6: ['CC1C=CC(C(C)C)CC1', 'O']

NN-rank 7: ['C', 'CC(C)C1CCCCC1O']

NN-rank 8: ['CC1C=C(O)C(C(C)C)CC1']

NN-rank 9: ['CC1CCC(C(C)C)=C(O)C1']

NN-rank 10: ['CC1CCCC(O)C1', 'CCC']

EI-MS matched molecular weight (relative abundance: 30.0%) for CCC

GHS hazard classification: none of the hazards selected for correlation

NN-rank 11: ['CCC(C)CC(O)CC(C)C']

NN-rank 12: ['CC(C)C1CCC(C)C2OC12']

NN-rank 13: ['CC(C)C1C(O)CC2CC1C2']

NN-rank 14: ['CC(C)CC(O)C(C)C(C)C']

NN-rank 15: ['CC(C)CCCC(C)CCO']

NN-rank 16: ['CC1CCC2(C(C)C)OC2C1']

NN-rank 17: ['CC1CCC(C(C)C)C(O)C1']

NN-rank 18: ['CC1CC=CCC1', 'CCC', 'O']

EI-MS matched molecular weight (relative abundance: 54.0%) for CC1CC=CCC1

GHS hazard classification: Health Hazard, Irritant

EI-MS matched molecular weight (relative abundance: 30.0%) for CCC

GHS hazard classification: none of the hazards selected for correlation

NN-rank 19: ['CC(C)C1C(C)CCCC1O']

NN-rank 20: ['CCC(C)CC=CC(C)C', 'O']

NN-rank 21: ['CC1C=C=C(C(C)C)CC1', 'O']

NN-rank 22: ['CCCC(O)C(CC)C(C)C']

NN-rank 23: ['CC(C)CC(O)CC1CCC1']

NN-rank 24: ['CC(C)CCCC(C)CC=O']

NN-rank 25: ['CC(C)CCCC(C)C=C=O']

NN/MS matches corresponding with tox GHS classification highlight in Bold font.

151. Menthone: CC1CCC(C(=O)C1)C(C)C

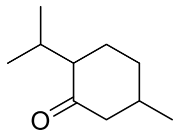

Summary Report of GHS Classification for NN/MS Matches:

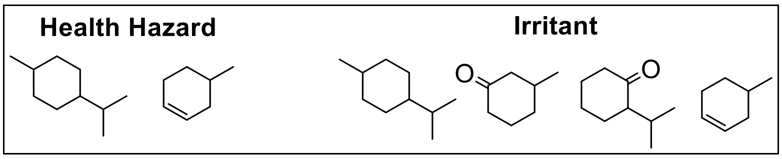

NN-rank 1: ['CC1CCC(C(C)C)CC1', 'O']

EI-MS matched molecular weight (relative abundance: 43.0%) for CC1CCC(C(C)C)CC1

GHS hazard classification: Health Hazard, Irritant

NN-rank 2: ['CC1CCCC(=O)C1', 'CCC']

EI-MS matched molecular weight (relative abundance: 22.0%) for CC1CCCC(=O)C1

GHS hazard classification: Irritant

EI-MS matched molecular weight (relative abundance: 51.0%) for CCC

GHS hazard classification: none of the hazards selected for correlation

NN-rank 3: ['CC1CC=C(C(C)C)C(=O)C1']

NN-rank 4: ['C', 'CCC1CCC(C)CC1=O']

EI-MS matched molecular weight (relative abundance: 43.0%) for CCC1CCC(C)CC1=O

GHS hazard classification: none of the hazards selected for correlation

NN-rank 5: ['C', 'CCC1CCC(C)CC1=O']

EI-MS matched molecular weight (relative abundance: 43.0%) for CCC1CCC(C)CC1=O

GHS hazard classification: none of the hazards selected for correlation

NN-rank 6: ['CC1CCC(C(C)C)C(O)C1']

NN-rank 7: ['CCC(C)CC(=O)CC(C)C']

NN-rank 8: ['CC(C)CCCC(C)CC=O']

NN-rank 9: ['CC(C)=C1CCC(C)CC1=O']

NN-rank 10: ['CC1=CC(=O)C(C(C)C)CC1']

NN-rank 11: ['CC1CC=CC(=O)C1', 'CCC']

EI-MS matched molecular weight (relative abundance: 51.0%) for CCC

GHS hazard classification: none of the hazards selected for correlation

NN-rank 12: ['C', 'CC(C)C1CCCCC1=O']

NN/MS matches corresponding with tox GHS classification highlight in Bold font.

EI-MS matched molecular weight (relative abundance: 43.0%) for CC(C)C1CCCCC1=O

GHS hazard classification: Irritant

NN-rank 13: ['CC1CC=C(C(C)C)CC1', 'O']

NN-rank 14: ['CC(C)CCCC(C)CCO']

NN-rank 15: ['CC1C=CC(C(C)C)CC1', 'O']

NN-rank 16: ['CC(C)C=CCC(C)CC=O']

NN-rank 17: ['CC(C)=CCCC(C)CC=O']

NN-rank 18: ['CCC(C)CC(=O)C=C(C)C']

NN-rank 19: ['CC1CC=CCC1', 'CCC', 'O']

EI-MS matched molecular weight (relative abundance: 15.0%) for CC1CC=CCC1

GHS hazard classification: Health Hazard, Irritant

EI-MS matched molecular weight (relative abundance: 51.0%) for CCC

GHS hazard classification: none of the hazards selected for correlation

NN-rank 20: ['CC1CCC(C(C)C)=C(O)C1']

NN-rank 21: ['CC(C)C', 'CCC(C)CC=O']

NN-rank 22: ['CC1C=C(O)C(C(C)C)CC1']

NN-rank 23: ['CC(C)CCCC(C)C=C=O']

NN-rank 24: ['CCC', 'CCC(C)CC(C)=O']

EI-MS matched molecular weight (relative abundance: 51.0%) for CCC

GHS hazard classification: none of the hazards selected for correlation

NN-rank 25: ['C', 'CC(C)C1CCC=CC1=O']

NN/MS matches corresponding with tox GHS classification highlight in Bold font.

152. Menthyl acetate: CC1CCC(C(C1)OC(=O)C)C(C)C

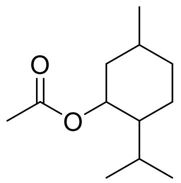

Summary Report of GHS Classification for NN/MS Matches:

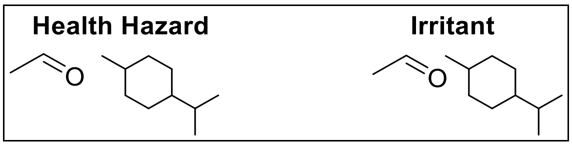

NN-rank 1: ['CC1CCC(C(C)C)C(O)C1', 'CC=O']

EI-MS matched molecular weight (relative abundance: 65.89%) for CC=O

GHS hazard classification: Health Hazard, Irritant

NN-rank 2: ['C', 'CCC1CCC(C)CC1OC(C)=O']

NN-rank 3: ['C', 'CCC1CCC(C)CC1OC(C)=O']

NN-rank 4: ['CC(=O)OC1CC(C)C=CC1C(C)C']

NN-rank 5: ['CC(=O)O', 'CC1C=CC(C(C)C)CC1']

NN-rank 6: ['CC(=O)OC1CC(C)CC=C1C(C)C']

NN-rank 7: ['CC(=O)O', 'CC1CC=C(C(C)C)CC1']

NN-rank 8: ['CC(=O)OC1=CC(C)CCC1C(C)C']

NN-rank 9: ['CC(=O)O', 'CC1CCC(C(C)C)CC1']

EI-MS matched molecular weight (relative abundance: 7.19%) for CC1CCC(C(C)C)CC1

GHS hazard classification: Health Hazard, Irritant

NN-rank 10: ['C', 'CC(=O)OC1CCCCC1C(C)C']

NN-rank 11: ['CC(=O)OC1CC(C)CCC1=C(C)C']

NN-rank 12: ['CC(=O)OC1=C(C(C)C)CCC(C)C1']

NN-rank 13: ['CCC(C)CC(CC(C)C)OC(C)=O']

NN-rank 14: ['CC(=O)OC1CC2CC(C2)C1C(C)C']

NN-rank 15: ['CC(=O)OC(CC(C)C)C(C)C(C)C']

NN-rank 16: ['CC(=O)OC1CC(C)CCC1C(C)C']

NN-rank 17: ['CC(=O)OC1CCCC(C)C1', 'CCC']

EI-MS matched molecular weight (relative abundance: 65.89%) for CCC

GHS hazard classification: none of the hazards selected for correlation

NN-rank 18: ['CC(=O)OC1CCCC(C)C1C(C)C']

NN/MS matches corresponding with tox GHS classification highlight in Bold font.

NN-rank 19: ['C=C(C(C)C)C(CC(C)C)OC(C)=O']

NN-rank 20: ['CC(=O)OC(CC(C)C)CC1CCC1']

NN-rank 21: ['C=CC(C)CC(CC(C)C)OC(C)=O']

NN-rank 22: ['CCCC(OC(C)=O)C(CC)C(C)C']

NN-rank 23: ['C', 'C', 'CC(=O)OC1CC(C)CCC1C']

NN-rank 24: ['CC(=O)OC1CC2CC(=C1C(C)C)C2']

NN-rank 25: ['CC(=O)O', 'CCC(C)CC=CC(C)C']

EI-MS matched molecular weight (relative abundance: 7.19%) for CCC(C)CC=CC(C)C

GHS hazard classification: none of the hazards selected for correlation

NN/MS matches corresponding with tox GHS classification highlight in Bold font.

153. Methyl-alpha-ionone of the hazards selected for correlation:

CCC(=O)/C=C/C1C(=CCCC1(C)C)C

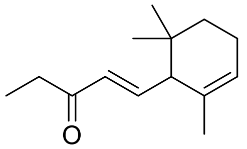

NN-rank 1: ['CCC(=O)/C=C/C1=C(C)CCCC1(C)C']

NN-rank 2: ['CCC(=O)CCC1C(C)=CCCC1(C)C']

NN-rank 3: ['CCC(=O)CC=C1C(C)=CCCC1(C)C']

NN-rank 4: ['C=C1CCCC(C)(C)C1/C=C/C(=O)CC']

NN-rank 5: ['CCC(=O)/C=C/C1C(C)CCCC1(C)C']

NN-rank 6: ['C=C1C=CCC(C)(C)C1/C=C/C(=O)CC']

NN-rank 7: ['CCC(=O)/C=C/C1=C(C)C=CCC1(C)C']

NN-rank 8: ['CCC(=O)/C=C/C1C(C)C=CCC1(C)C']

NN-rank 9: ['C=C1C2CCC(C)(C)C1C2CC(=O)CC']

NN-rank 10: ['CCC(=O)CC1C2CCC(C)(C)C1C2C']

NN-rank 11: ['CCC(=O)CC1C2=C(C)C1CCC2(C)C']

NN-rank 12: ['CCC(=O)C=C=C1C(C)=CCCC1(C)C']

NN-rank 13: ['CCC(=O)CC1C2=C(C)C1C(C)(C)CC2']

NN-rank 14: ['CCC(=O)/C=C/C=C(C)CCCC(C)C']

NN-rank 15: ['CCC(=O)CC=CC(C)=CCCC(C)C']

NN-rank 16: ['CCC(=O)CC1C2=CCC(C)(C)C1C2C']

NN-rank 17: ['C=C1C2=CCC(C)(C)C1C2CC(=O)CC']

NN-rank 18: ['CCC(=O)/C=C/C1C(C)=C=CCC1(C)C']

NN-rank 19: ['C=C1C2=C(CC(=O)CC)C1CCC2(C)C']

NN-rank 20: ['CCC(O)/C=C/C1C(C)=CCCC1(C)C']

NN-rank 21: ['CCC(=O)CC1=C2C(C)C1CCC2(C)C']

NN-rank 22: ['CCC(=O)CC=C=C(C)CCCC(C)C']

NN-rank 23: ['CCC(=O)/C=C1/C2=C(C)C1CCC2(C)C']

NN-rank 24: ['CCC(=O)CC1C2=CCC(C)(C)C1=C2C']

NN-rank 25: ['CCC(=O)/C=C/C=C(C)C=CCC(C)C']

NN/MS matches corresponding with tox GHS classification highlight in Bold font.

154. Methyl Anthranilate: COC(=O)C1=CC=CC=C1N

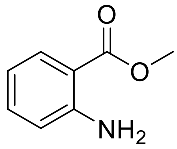

Summary Report of GHS Classification for NN/MS Matches:

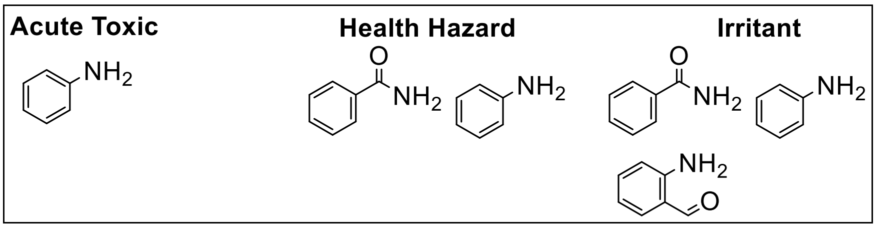

NN-rank 1: ['C', 'Nc1ccccc1C(=O)O']

NN-rank 2: ['C', 'Nc1ccccc1C(=O)O']

NN-rank 3: ['C', 'Nc1ccccc1C=O', 'O']

EI-MS matched molecular weight (relative abundance: 29.93%) for Nc1ccccc1C=O

GHS hazard classification: Irritant

NN-rank 4: ['C', 'Nc1c2cccc1C2=O', 'O']

NN-rank 5: ['C', 'O', 'O=C1Nc2ccccc21']

NN-rank 6: ['COCc1ccccc1N', 'O']

NN-rank 7: ['CO', 'Nc1ccccc1CO']

NN-rank 8: ['CO', 'Nc1ccccc1C=O']

EI-MS matched molecular weight (relative abundance: 29.93%) for Nc1ccccc1C=O

GHS hazard classification: Irritant

NN-rank 9: ['CO', 'O=C1Nc2ccccc21']

NN-rank 10: ['COC=O', 'Nc1ccccc1']

EI-MS matched molecular weight (relative abundance: 56.15%) for Nc1ccccc1

GHS hazard classification: Acute Toxic, Health Hazard, Irritant

NN-rank 11: ['COC(=O)c1ccccc1', 'N']

NN-rank 12: ['CO', 'Cc1ccccc1N', 'O']

NN-rank 13: ['C', 'NC(=O)c1ccccc1', 'O']

EI-MS matched molecular weight (relative abundance: 29.93%) for NC(=O)c1ccccc1

GHS hazard classification: Health Hazard, Irritant

NN-rank 14: ['C', 'C=O', 'Nc1ccccc1', 'O']

NN/MS matches corresponding with tox GHS classification highlight in Bold font.

EI-MS matched molecular weight (relative abundance: 56.15%) for Nc1ccccc1

GHS hazard classification: Acute Toxic, Health Hazard, Irritant

NN-rank 15: ['C', 'Nc1cccc2c(=O)c12', 'O']

NN-rank 16: ['C', 'N', 'O=C1Oc2ccccc21']

EI-MS matched molecular weight (relative abundance: 99.99%) for O=C1Oc2ccccc21

GHS hazard classification: none of the hazards selected for correlation

NN-rank 17: ['CO', 'Nc1c2cccc1C2=O']

NN-rank 18: ['C', 'Nc1ccccc1', 'O=C=O']

EI-MS matched molecular weight (relative abundance: 56.15%) for Nc1ccccc1

GHS hazard classification: Acute Toxic, Health Hazard, Irritant

NN-rank 19: ['CO', 'NC(=O)c1ccccc1']

EI-MS matched molecular weight (relative abundance: 29.93%) for NC(=O)c1ccccc1

GHS hazard classification: Health Hazard, Irritant

NN-rank 20: ['C', 'Nc1ccccc1', 'O=CO']

EI-MS matched molecular weight (relative abundance: 56.15%) for Nc1ccccc1

GHS hazard classification: Acute Toxic, Health Hazard, Irritant

NN-rank 21: ['C', 'N', 'O', 'O=c1c2ccccc12']

NN-rank 22: ['COC', 'Nc1ccccc1', 'O']

EI-MS matched molecular weight (relative abundance: 56.15%) for Nc1ccccc1

GHS hazard classification: Acute Toxic, Health Hazard, Irritant

NN-rank 23: ['Cc1cccc(N)c1C(=O)O']

NN-rank 24: ['COC1=Nc2ccccc21', 'O']

NN-rank 25: ['c1ccc2c(c1)CN2', 'CO', 'O']

NN/MS matches corresponding with tox GHS classification highlight in Bold font.

155. Methyl Cinnamate: COC(=O)/C=C/C1=CC=CC=C1

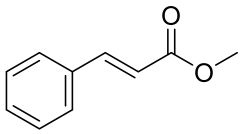

Summary Report of GHS Classification for NN/MS Matches:

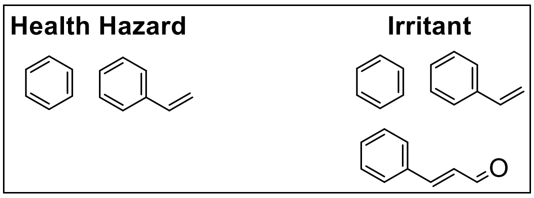

NN-rank 1: ['C', 'O=C(O)/C=C/c1ccccc1']

NN-rank 2: ['C', 'O=C(O)/C=C/c1ccccc1']

NN-rank 3: ['COC(=O)CCc1ccccc1']

NN-rank 4: ['CO', 'OC/C=C/c1ccccc1']

NN-rank 5: ['C', 'O', 'O=C/C=C/c1ccccc1']

EI-MS matched molecular weight (relative abundance: 99.99%) for O=C/C=C/c1ccccc1

GHS hazard classification: Irritant

NN-rank 6: ['CO', 'O=C/C=C/c1ccccc1']

EI-MS matched molecular weight (relative abundance: 99.99%) for O=C/C=C/c1ccccc1

GHS hazard classification: Irritant

NN-rank 7: ['C', 'O=C1C=C(c2ccccc2)O1']

NN-rank 8: ['C', 'O=C1CC(c2ccccc2)O1']

NN-rank 9: ['COC/C=C/c1ccccc1', 'O']

NN-rank 10: ['C', 'O=C/C=C(\\O)c1ccccc1']

NN-rank 11: ['C', 'O', 'O=c1cc1-c1ccccc1']

NN-rank 12: ['CO', 'O=c1cc1-c1ccccc1']

NN-rank 13: ['C/C=C/c1ccccc1', 'CO', 'O']

NN-rank 14: ['CO/C(=C\\C=O)c1ccccc1']

EI-MS matched molecular weight (relative abundance: 13.51%) for CO/C(=C\C=O)c1ccccc1

GHS hazard classification: none of the hazards selected for correlation

NN-rank 15: ['C=Cc1ccccc1', 'COC=O']

NN/MS matches corresponding with tox GHS classification highlight in Bold font.

EI-MS matched molecular weight (relative abundance: 38.03%) for C=Cc1ccccc1

GHS hazard classification: Health Hazard, Irritant

NN-rank 16: ['C', 'O=C1O/C1=C\\c1ccccc1']

NN-rank 17: ['C', 'O', 'O=C1C=Cc2ccccc21']

NN-rank 18: ['C', 'O', 'O=C1C=Cc2ccccc21']

NN-rank 19: ['CCc1ccccc1', 'COC=O']

NN-rank 20: ['C=CC(=O)OC', 'c1ccccc1']

EI-MS matched molecular weight (relative abundance: 19.72%) for c1ccccc1

GHS hazard classification: Health Hazard, Irritant

NN-rank 21: ['CO', 'O=C1C=Cc2ccccc21']

NN-rank 22: ['CO', 'O=C1C=Cc2ccccc21']

NN-rank 23: ['C', 'OC/C=C(\\O)c1ccccc1']

NN-rank 24: ['C', 'O=CCC(O)c1ccccc1']

NN-rank 25: ['C', 'O=C1OC1Cc1ccccc1']

NN/MS matches corresponding with tox GHS classification highlight in Bold font.

156. Methyl cyclopentenolone: CC1=C(C(=O)CC1)O

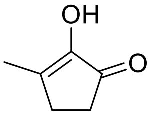

Summary Report of GHS Classification for NN/MS Matches:

Acute Toxic	Health Hazard	Irritant
=O	=O	=O

NN-rank 1: ['CC1CCC(=O)C1O']

EI-MS matched molecular weight (relative abundance: 7.41%) for CC1CCC(=O)C1O

GHS hazard classification: none of the hazards selected for correlation

NN-rank 2: ['CC1=C(O)C(O)CC1']

EI-MS matched molecular weight (relative abundance: 7.41%) for CC1=C(O)C(O)CC1

GHS hazard classification: none of the hazards selected for correlation

NN-rank 3: ['CCC(C)=C(O)C=O']

EI-MS matched molecular weight (relative abundance: 7.41%) for CCC(C)=C(O)C=O

GHS hazard classification: none of the hazards selected for correlation

NN-rank 4: ['CC1=C(O)C(=O)C=C1']

NN-rank 5: ['CCC(C)=C(O)CO']

NN-rank 6: ['C', 'O=C1CCCC1O']

NN-rank 7: ['C', 'O=C1CCC=C1O']

EI-MS matched molecular weight (relative abundance: 6.11%) for O=C1CCC=C1O

GHS hazard classification: none of the hazards selected for correlation

NN-rank 8: ['CC(CO)CCC=O']

NN-rank 9: ['CC(=CO)CCC=O']

EI-MS matched molecular weight (relative abundance: 7.41%) for CC(=CO)CCC=O

GHS hazard classification: unknown

NN-rank 10: ['CC(=O)C(O)=C(C)C']

EI-MS matched molecular weight (relative abundance: 7.41%) for CC(=O)C(O)=C(C)C

GHS hazard classification: none of the hazards selected for correlation

NN-rank 11: ['CCC(=O)C(O)CC']

NN-rank 12: ['CC=C(O)C(=O)CC']

EI-MS matched molecular weight (relative abundance: 7.41%) for CC=C(O)C(=O)CC

GHS hazard classification: none of the hazards selected for correlation

NN/MS matches corresponding with tox GHS classification highlight in Bold font.

NN-rank 13: ['CC1=C(O)CCC1', 'O']

EI-MS matched molecular weight (relative abundance: 6.11%) for CC1=C(O)CCC1

GHS hazard classification: none of the hazards selected for correlation

NN-rank 14: ['C=O', 'CCC(C)CO']

EI-MS matched molecular weight (relative abundance: 7.21%) for C=O

GHS hazard classification: Acute Toxic, Health Hazard, Irritant

NN-rank 15: ['CCCCC(=O)CO']

NN-rank 16: ['CC(=CO)CCCO']

NN-rank 17: ['CC1=C(O)C(O)=CC1']

NN-rank 18: ['C=O', 'CCC(C)=CO']

EI-MS matched molecular weight (relative abundance: 7.21%) for C=O

GHS hazard classification: Acute Toxic, Health Hazard, Irritant

NN-rank 19: ['O=C1CC2CC2=C1O']

NN-rank 20: ['C=CC(C)=C(O)C=O']

NN-rank 21: ['CC(CO)CCCO']

NN-rank 22: ['CC1=C(O)C2CC1O2']

NN-rank 23: ['CC12CC1C(=O)C2O']

NN-rank 24: ['C', 'CC(C)=C(O)C=O']

NN-rank 25: ['CC1=CC(=O)CC1', 'O']

NN/MS matches corresponding with tox GHS classification highlight in Bold font.

157. Methyl dihydrojasmonate: CCCCCC1C(CCC1=O)CC(=O)OC

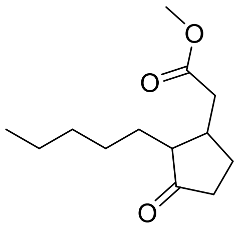

Summary Report of GHS Classification for NN/MS Matches:

Irritant

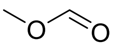

NN-rank 1: ['C', 'CCCCCC1C(=O)CCC1CC(=O)O']

NN-rank 2: ['C', 'CCCCCC1C(=O)CCC1CC(=O)O']

NN-rank 3: ['CCCCCC1C(=O)CCC1=CC(=O)OC']

NN-rank 4: ['CCCCCC1C(O)CCC1CC(=O)OC']

NN-rank 5: ['C', 'CCCCC1C(=O)CCC1CC(=O)OC']

NN-rank 6: ['CCCCCC1C(=O)CCC1CCO', 'CO']

NN-rank 7: ['C', 'CCCCCC1C(=O)CCC1CC=O', 'O']

NN-rank 8: ['CCCCCC1C(=O)C=CC1CC(=O)OC']

NN-rank 9: ['CCCCCC1CCCC1CC(=O)OC', 'O']

NN-rank 10: ['CCCCCC1C(=O)CCC1CC=O', 'CO']

NN-rank 11: ['CCCCCC1C(=O)CCC1CCOC', 'O']

NN-rank 12: ['CCCCCCC(CCC=O)CC(=O)OC']

NN-rank 13: ['CCCCCC1C(=O)CCC1C', 'COC=O']

EI-MS matched molecular weight (relative abundance: 7.1%) for COC=O

GHS hazard classification: Irritant

NN-rank 14: ['CCCCC', 'COC(=O)CC1CCC(=O)C1']

NN-rank 15: ['CCCCCC(CCC(=O)OC)C(=O)CC']

NN-rank 16: ['CCCCCC(C=O)C(CC)CC(=O)OC']

NN-rank 17: ['CCCCCC(CO)C(CC)CC(=O)OC']

NN-rank 18: ['CCCCCCC(CCCO)CC(=O)OC']

NN-rank 19: ['C=O', 'CCCCCCC(CC)CC(=O)OC']

NN-rank 20: ['CCCCCC(C=CC(=O)OC)C(=O)CC']

NN-rank 21: ['C=C1CCC(=O)C1CCCCC', 'COC=O']

NN/MS matches corresponding with tox GHS classification highlight in Bold font.

EI-MS matched molecular weight (relative abundance: 7.1%) for COC=O

GHS hazard classification: Irritant

NN-rank 22: ['CCCCCC(C(C)=O)C(C)CC(=O)OC']

NN-rank 23: ['C=CC(=O)C(CCCCC)CCC(=O)OC']

NN-rank 24: ['C=CC(CC(=O)OC)C(C=O)CCCCC']

NN-rank 25: ['CCCCCC1C(=O)CCC1CC', 'CO', 'O']

NN/MS matches corresponding with tox GHS classification highlight in Bold font.

158. Methyl Salicylate: COC(=O)C1=CC=CC=C1O

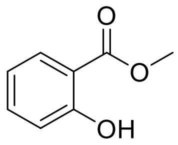

Summary Report of GHS Classification for NN/MS Matches:

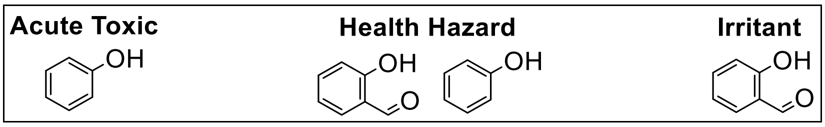

NN-rank 1: ['C', 'O=C(O)c1ccccc1O']

NN-rank 2: ['C', 'O', 'O=Cc1ccccc1O']

EI-MS matched molecular weight (relative abundance: 31.1%) for O=Cc1ccccc1O

GHS hazard classification: Health Hazard, Irritant

NN-rank 3: ['C', 'O=C(O)c1ccccc1O']

NN-rank 4: ['CO', 'OCc1ccccc1O']

NN-rank 5: ['CO', 'O=Cc1ccccc1O']

EI-MS matched molecular weight (relative abundance: 31.1%) for O=Cc1ccccc1O

GHS hazard classification: Health Hazard, Irritant

NN-rank 6: ['COCc1ccccc1O', 'O']

NN-rank 7: ['C', 'O', 'O=C1c2cccc1c2O']

NN-rank 8: ['CO', 'Cc1ccccc1O', 'O']

NN-rank 9: ['COC=O', 'Oc1ccccc1']

EI-MS matched molecular weight (relative abundance: 14.5%) for Oc1ccccc1

GHS hazard classification: Acute Toxic, Health Hazard

NN-rank 10: ['C', 'C=O', 'O', 'Oc1ccccc1']

EI-MS matched molecular weight (relative abundance: 14.5%) for Oc1ccccc1

GHS hazard classification: Acute Toxic, Health Hazard

NN-rank 11: ['CO', 'O=C1c2cccc1c2O']

NN-rank 12: ['C', 'O', 'O=c1c2cccc(O)c12']

NN-rank 13: ['COC', 'O', 'Oc1ccccc1']

EI-MS matched molecular weight (relative abundance: 14.5%) for Oc1ccccc1

GHS hazard classification: Acute Toxic, Health Hazard

NN-rank 14: ['C', 'O=C=O', 'Oc1ccccc1']

NN/MS matches corresponding with tox GHS classification highlight in Bold font.

EI-MS matched molecular weight (relative abundance: 14.5%) for Oc1ccccc1

GHS hazard classification: Acute Toxic, Health Hazard

NN-rank 15: ['C', 'O=CO', 'Oc1ccccc1']

EI-MS matched molecular weight (relative abundance: 14.5%) for Oc1ccccc1

GHS hazard classification: Acute Toxic, Health Hazard

NN-rank 16: ['C', 'O=C1Oc2cccc(O)c21']

NN-rank 17: ['CO', 'O', 'Oc1c2cccc1C2']

NN-rank 18: ['Cc1cccc(C(=O)O)c1O']

NN-rank 19: ['C', 'O=C1Oc2cccc1c2O']

NN-rank 20: ['C=O', 'CO', 'Oc1ccccc1']

EI-MS matched molecular weight (relative abundance: 14.5%) for Oc1ccccc1

GHS hazard classification: Acute Toxic, Health Hazard

NN-rank 21: ['Cc1cccc(O)c1C(=O)O']

NN-rank 22: ['C', 'O', 'O=C1Oc2ccccc21']

NN-rank 23: ['C', 'O', 'OCc1ccccc1O']

NN-rank 24: ['COCc1cccc(O)c1O']

NN-rank 25: ['C', 'O=Cc1cccc(O)c1O']

NN/MS matches corresponding with tox GHS classification highlight in Bold font.

159. Methyl Thiobutyrate: CCCC(OC)=S

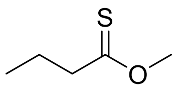

NN-rank 1: ['C', 'CCCC(O)=S']

EI-MS matched molecular weight (relative abundance: 8.39%) for CCCC(O)=S

GHS hazard classification: none of the hazards selected for correlation

NN-rank 2: ['C', 'S=C1CCCO1']

NN-rank 3: ['C', 'CCC1OC1=S']

NN-rank 4: ['C', 'CC1CC(=S)O1']

NN-rank 5: ['C', 'CCC(=S)OC']

EI-MS matched molecular weight (relative abundance: 8.39%) for CCC(=S)OC

GHS hazard classification: none of the hazards selected for correlation

NN-rank 6: ['CCC(C)C(O)=S']

NN-rank 7: ['CC', 'COC(C)=S']

NN-rank 8: ['C', 'CC', 'S=C1CO1']

NN-rank 9: ['CCCC=S', 'CO']

NN-rank 10: ['C', 'CCC(=S)OC']

EI-MS matched molecular weight (relative abundance: 8.39%) for CCC(=S)OC

GHS hazard classification: none of the hazards selected for correlation

NN-rank 11: ['C', 'OCCCC=S']

EI-MS matched molecular weight (relative abundance: 8.39%) for OCCCC=S

GHS hazard classification: none of the hazards selected for correlation

NN-rank 12: ['C', 'CCC(O)C=S']

EI-MS matched molecular weight (relative abundance: 8.39%) for CCC(O)C=S

GHS hazard classification: none of the hazards selected for correlation

NN-rank 13: ['C', 'CCCC(=O)S']

EI-MS matched molecular weight (relative abundance: 8.39%) for CCCC(=O)S

GHS hazard classification: none of the hazards selected for correlation

NN-rank 14: ['C', 'C', 'S=C1CCO1']

NN-rank 15: ['C=CCC(=S)OC']

NN-rank 16: ['C', 'CC(O)CC=S']

EI-MS matched molecular weight (relative abundance: 8.39%) for CC(O)CC=S

GHS hazard classification: none of the hazards selected for correlation

NN-rank 17: ['C', 'CCOC(C)=S']

NN/MS matches corresponding with tox GHS classification highlight in Bold font.

EI-MS matched molecular weight (relative abundance: 8.39%) for CCOC(C)=S

GHS hazard classification: none of the hazards selected for correlation

NN-rank 18: ['COC(=S)C1CC1']

NN-rank 19: ['CC', 'CCC(O)=S']

NN-rank 20: ['C', 'S=C1CC=CO1']

NN-rank 21: ['C', 'CCCC=O', 'S']

EI-MS matched molecular weight (relative abundance: 36.44%) for CCCC=O

GHS hazard classification: none of the hazards selected for correlation

NN-rank 22: ['C', 'S=C1OC12CC2']

NN-rank 23: ['C', 'C=C1CC(=S)O1']

NN-rank 24: ['CCC=C=S', 'CO']

NN-rank 25: ['CC=CC(=S)OC']

NN/MS matches corresponding with tox GHS classification highlight in Bold font.

160. Terpinolene: CC1=CCC(=C(C)C)CC1

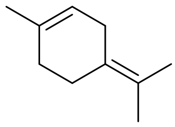

Summary Report of GHS Classification for NN/MS Matches:

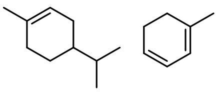

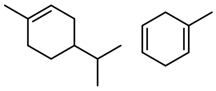

NN-rank 1: ['CC1=CCC(C(C)C)=CC1']

NN-rank 2: ['CC1=CC=C(C(C)C)CC1']

NN-rank 3: ['CC1=C=CC(=C(C)C)CC1']

NN-rank 4: ['CC1=CC=CCC1', 'CCC']

EI-MS matched molecular weight (relative abundance: 99.99%) for CC1=CC=CCC1

GHS hazard classification: Health Hazard

EI-MS matched molecular weight (relative abundance: 11.02%) for CCC

GHS hazard classification: none of the hazards selected for correlation

NN-rank 5: ['CC1=C=C=C(C(C)C)CC1']

NN-rank 6: ['CC1=CC=C=CC1', 'CCC']

EI-MS matched molecular weight (relative abundance: 61.73%) for CC1=CC=C=CC1

GHS hazard classification: none of the hazards selected for correlation

EI-MS matched molecular weight (relative abundance: 11.02%) for CCC

GHS hazard classification: none of the hazards selected for correlation

NN-rank 7: ['CC1=CCC=CC1', 'CCC']

EI-MS matched molecular weight (relative abundance: 99.99%) for CC1=CCC=CC1

GHS hazard classification: Irritant

EI-MS matched molecular weight (relative abundance: 11.02%) for CCC

GHS hazard classification: none of the hazards selected for correlation

NN-rank 8: ['CC(C)=C1C=CC(C)CC1']

NN-rank 9: ['CC1=CCC(C(C)C)CC1']

EI-MS matched molecular weight (relative abundance: 6.93%) for CC1=CCC(C(C)C)CC1

GHS hazard classification: Health Hazard, Irritant

NN-rank 10: ['CC(C)=C1CCC(C)CC1']

NN/MS matches corresponding with tox GHS classification highlight in Bold font.

EI-MS matched molecular weight (relative abundance: 6.93%) for CC(C)=C1CCC(C)CC1

GHS hazard classification: none of the hazards selected for correlation

NN-rank 11: ['CC1=CCC(=C(C)C)CC1']

NN-rank 12: ['C=C(C)C1=CCC(C)=CC1']

NN-rank 13: ['C=C(C)C1=CCC(C)=CC1']

NN-rank 14: ['C=C(C)C1=CC=C(C)CC1']

NN-rank 15: ['C=C(C)C1=CC=C(C)CC1']

NN-rank 16: ['CC1=CCCCC1', 'CCC']

EI-MS matched molecular weight (relative abundance: 11.02%) for CCC

GHS hazard classification: none of the hazards selected for correlation

NN-rank 17: ['CC1=CCC(=C(C)C)C=C1']

NN-rank 18: ['C', 'CC(C)=C1CC=CCC1']

EI-MS matched molecular weight (relative abundance: 78.35%) for CC(C)=C1CC=CCC1

GHS hazard classification: none of the hazards selected for correlation

NN-rank 19: ['CC1=C=C=CCC1', 'CCC']

EI-MS matched molecular weight (relative abundance: 61.73%) for CC1=C=C=CCC1

GHS hazard classification: none of the hazards selected for correlation

EI-MS matched molecular weight (relative abundance: 11.02%) for CCC

GHS hazard classification: none of the hazards selected for correlation

NN-rank 20: ['C', 'CC(C)=C1CCCCC1']

NN-rank 21: ['CC1=C=C=C=CC1', 'CCC']

EI-MS matched molecular weight (relative abundance: 11.02%) for CCC

GHS hazard classification: none of the hazards selected for correlation

NN-rank 22: ['C', 'CC(C)=C1C=CCCC1']

EI-MS matched molecular weight (relative abundance: 78.35%) for CC(C)=C1C=CCCC1

GHS hazard classification: none of the hazards selected for correlation

NN-rank 23: ['C=C(CC=C(C)C)C(C)C']

EI-MS matched molecular weight (relative abundance: 6.93%) for C=C(CC=C(C)C)C(C)C

GHS hazard classification: none of the hazards selected for correlation

NN-rank 24: ['CCC(C)=CC=C=C(C)C']

NN-rank 25: ['CC1C=C=C(C(C)C)CC1']

NN/MS matches corresponding with tox GHS classification highlight in Bold font.

161. Methyl-thio-methylpyrazine: CSC1=NC=CN=C1C

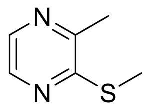

Summary Report of GHS Classification for NN/MS Matches:

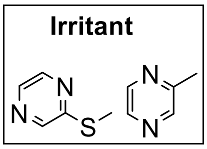

NN-rank 1: ['CS', 'Cc1cnccn1']

EI-MS matched molecular weight (relative abundance: 18.49%) for Cc1cnccn1

GHS hazard classification: Irritant

NN-rank 2: ['C', 'Cc1nccnc1S']

EI-MS matched molecular weight (relative abundance: 27.49%) for Cc1nccnc1S

GHS hazard classification: none of the hazards selected for correlation

NN-rank 3: ['C', 'CSc1cnccn1']

EI-MS matched molecular weight (relative abundance: 27.49%) for CSc1cnccn1

GHS hazard classification: Irritant

NN-rank 4: ['Cc1nc2cnc1SC2']

NN-rank 5: ['C=S', 'Cc1cnccn1']

EI-MS matched molecular weight (relative abundance: 18.49%) for Cc1cnccn1

GHS hazard classification: Irritant

NN-rank 6: ['c1cnc2c(n1)CCS2']

NN-rank 7: ['Cc1ncc2nc1SC2']

NN-rank 8: ['CSc1cncc(C)n1']

EI-MS matched molecular weight (relative abundance: 12.19%) for CSc1cncc(C)n1

GHS hazard classification: none of the hazards selected for correlation

NN-rank 9: ['Cc1c2ncc[n+]1CS2']

NN-rank 10: ['Cc1ncc[n+]2c1SC2']

NN-rank 11: ['C', 'c1cnc2c(n1)CS2']

NN-rank 12: ['CSc1cnc(C)cn1']

EI-MS matched molecular weight (relative abundance: 12.19%) for CSc1cnc(C)cn1

GHS hazard classification: none of the hazards selected for correlation

NN-rank 13: ['Cc1cnc(S)c(C)n1']

NN/MS matches corresponding with tox GHS classification highlight in Bold font.

EI-MS matched molecular weight (relative abundance: 12.19%) for Cc1cnc(S)c(C)n1

GHS hazard classification: none of the hazards selected for correlation

NN-rank 14: ['CCSc1cnccn1']

EI-MS matched molecular weight (relative abundance: 12.19%) for CCSc1cnccn1

GHS hazard classification: none of the hazards selected for correlation

NN-rank 15: ['Cc1c(S)ncc[n+]1C']

EI-MS matched molecular weight (relative abundance: 99.99%) for Cc1c(S)ncc[n+]1C

GHS hazard classification: unknown

NN-rank 16: ['CCc1nccnc1S']

EI-MS matched molecular weight (relative abundance: 12.19%) for CCc1nccnc1S

GHS hazard classification: none of the hazards selected for correlation

NN-rank 17: ['C', 'Cc1nc2cnc1s2']

NN-rank 18: ['Cc1cnc(C)c(S)n1']

EI-MS matched molecular weight (relative abundance: 12.19%) for Cc1cnc(C)c(S)n1

GHS hazard classification: none of the hazards selected for correlation

NN-rank 19: ['Cc1cncc(C=S)n1']

NN-rank 20: ['Cc1ncc[n+](C)c1S']

EI-MS matched molecular weight (relative abundance: 99.99%) for Cc1ncc[n+](C)c1S

GHS hazard classification: unknown

NN-rank 21: ['C', 'Cc1ncc2nc1S2']

NN-rank 22: ['Cc1cnc(C=S)cn1']

NN-rank 23: ['C', 'Cc1cncc(S)n1']

EI-MS matched molecular weight (relative abundance: 27.49%) for Cc1cncc(S)n1

GHS hazard classification: none of the hazards selected for correlation

NN-rank 24: ['S=CCc1cnccn1']

NN-rank 25: ['C', 'Cc1cnc(S)cn1']

EI-MS matched molecular weight (relative abundance: 27.49%) for Cc1cnc(S)cn1

GHS hazard classification: none of the hazards selected for correlation

NN/MS matches corresponding with tox GHS classification highlight in Bold font.

162. Myrcene: CC(=CCCC(=C)C=C)C

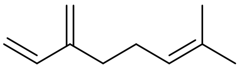

Summary Report of GHS Classification for NN/MS Matches:

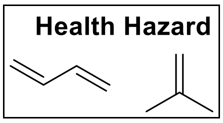

NN-rank 1: ['C', 'C=CC(=C)CCCCC']

NN-rank 2: ['C', 'C=CC(=C)CCCCC']

NN-rank 3: ['C=CC(=C)CCCC(C)C']

NN-rank 4: ['C=CC(=C)CCCC(=C)C']

NN-rank 5: ['C=CC(=C)CCCC(=C)C']

NN-rank 6: ['C=CC(=C)CC=CC(C)C']

NN-rank 7: ['CCC', 'C=CC(=C)CCC']

NN-rank 8: ['C', 'C', 'C=CC(=C)CCCC']

NN-rank 9: ['C', 'C=CC(=C)CC=CCC']

EI-MS matched molecular weight (relative abundance: 5.5%) for C=CC(=C)CC=CCC

GHS hazard classification: none of the hazards selected for correlation

NN-rank 10: ['C', 'C=CC(=C)CC=CCC']

EI-MS matched molecular weight (relative abundance: 5.5%) for C=CC(=C)CC=CCC

GHS hazard classification: none of the hazards selected for correlation

NN-rank 11: ['C=C(CC)CCC=C(C)C']

NN-rank 12: ['C', 'C=CC(=C)CCC=CC']

EI-MS matched molecular weight (relative abundance: 5.5%) for C=CC(=C)CCC=CC

GHS hazard classification: none of the hazards selected for correlation

NN-rank 13: ['C', 'C=CC(=C)CCC=CC']

EI-MS matched molecular weight (relative abundance: 5.5%) for C=CC(=C)CCC=CC

GHS hazard classification: none of the hazards selected for correlation

NN-rank 14: ['C', 'C', 'C=CC(=C)CC=CC']

NN-rank 15: ['C=CC(C)CCC=C(C)C']

NN-rank 16: ['CC(C)C', 'C=CC(=C)CC']

NN-rank 17: ['C=C(C)C', 'C=CC(=C)CC']

EI-MS matched molecular weight (relative abundance: 6.81%) for C=C(C)C

NN/MS matches corresponding with tox GHS classification highlight in Bold font.

GHS hazard classification: Health Hazard

NN-rank 18: ['C=CC=C', 'CCC=C(C)C']

EI-MS matched molecular weight (relative abundance: 14.11%) for C=CC=C

GHS hazard classification: Health Hazard

NN-rank 19: ['C=C(C)C', 'C=CC(=C)CC']

EI-MS matched molecular weight (relative abundance: 6.81%) for C=C(C)C

GHS hazard classification: Health Hazard

NN-rank 20: ['C=C(C)C', 'C=CC(=C)CC']

EI-MS matched molecular weight (relative abundance: 6.81%) for C=C(C)C

GHS hazard classification: Health Hazard

NN-rank 21: ['C', 'C', 'C=CCCC(=C)C=C']

NN-rank 22: ['C=CCC(=C)C=C', 'CCC']

EI-MS matched molecular weight (relative abundance: 85.58%) for C=CCC(=C)C=C

GHS hazard classification: none of the hazards selected for correlation

NN-rank 23: ['C=CCC', 'CCC=C(C)C']

EI-MS matched molecular weight (relative abundance: 6.81%) for C=CCC

GHS hazard classification: none of the hazards selected for correlation

NN-rank 24: ['C=C1CCCC(C)(C)CC1']

NN-rank 25: ['C=CC(C)=CCC=C(C)C']

NN/MS matches corresponding with tox GHS classification highlight in Bold font.

163. n-Butanol: CCCCO

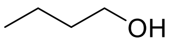

Summary Report of GHS Classification for NN/MS Matches:

Acute Toxic	Health Hazard	Irritant
—OH	—OH	═

NN-rank 1: ['C#CCC', 'O']

NN-rank 2: ['C=CCC', 'O']

EI-MS matched molecular weight (relative abundance: 27.95%) for C=CCC

GHS hazard classification: none of the hazards selected for correlation

NN-rank 3: ['CCCC', 'O']

EI-MS matched molecular weight (relative abundance: 7.6%) for CCCC

GHS hazard classification: none of the hazards selected for correlation

NN-rank 4: ['C#C', 'CC', 'O']

EI-MS matched molecular weight (relative abundance: 38.46%) for CC

GHS hazard classification: none of the hazards selected for correlation

NN-rank 5: ['CCC', 'CO']

EI-MS matched molecular weight (relative abundance: 68.42%) for CCC

GHS hazard classification: none of the hazards selected for correlation

EI-MS matched molecular weight (relative abundance: 98.12%) for CO

GHS hazard classification: Acute Toxic, Health Hazard

NN-rank 6: ['CCC#CO']

NN-rank 7: ['C', 'CCC', 'O']

EI-MS matched molecular weight (relative abundance: 10.46%) for C

GHS hazard classification: none of the hazards selected for correlation

EI-MS matched molecular weight (relative abundance: 68.42%) for CCC

GHS hazard classification: none of the hazards selected for correlation

NN-rank 8: ['C', 'CCCO']

EI-MS matched molecular weight (relative abundance: 10.46%) for C

GHS hazard classification: none of the hazards selected for correlation

NN-rank 9: ['CC', 'CCO']

EI-MS matched molecular weight (relative abundance: 38.46%) for CC

GHS hazard classification: none of the hazards selected for correlation

EI-MS matched molecular weight (relative abundance: 7.71%) for CCO

NN/MS matches corresponding with tox GHS classification highlight in Bold font.

GHS hazard classification: none of the hazards selected for correlation

NN-rank 10: ['C=C', 'CC', 'O']

EI-MS matched molecular weight (relative abundance: 57.03%) for C=C

GHS hazard classification: Irritant

EI-MS matched molecular weight (relative abundance: 38.46%) for CC

GHS hazard classification: none of the hazards selected for correlation

NN-rank 11: ['CCC(C)O']

NN-rank 12: ['C#CO', 'CC']

EI-MS matched molecular weight (relative abundance: 87.67%) for C#CO

GHS hazard classification: none of the hazards selected for correlation

EI-MS matched molecular weight (relative abundance: 38.46%) for CC

GHS hazard classification: none of the hazards selected for correlation

NN-rank 13: ['CCCCO']

NN-rank 14: ['CCCC=O']

NN-rank 15: ['C1CCC1', 'O']

EI-MS matched molecular weight (relative abundance: 27.95%) for C1CCC1

GHS hazard classification: none of the hazards selected for correlation

NN-rank 16: ['CCC=CO']

NN-rank 17: ['CC', 'CCO']

EI-MS matched molecular weight (relative abundance: 38.46%) for CC

GHS hazard classification: none of the hazards selected for correlation

EI-MS matched molecular weight (relative abundance: 7.71%) for CCO

GHS hazard classification: none of the hazards selected for correlation

NN-rank 18: ['CCC1CO1']

NN-rank 19: ['CC1CC1', 'O']

EI-MS matched molecular weight (relative abundance: 27.95%) for CC1CC1

GHS hazard classification: none of the hazards selected for correlation

NN-rank 20: ['C1CCOC1']

NN-rank 21: ['C1CC1', 'CO']

EI-MS matched molecular weight (relative abundance: 87.67%) for C1CC1

GHS hazard classification: none of the hazards selected for correlation

EI-MS matched molecular weight (relative abundance: 98.12%) for CO

GHS hazard classification: Acute Toxic, Health Hazard

NN-rank 22: ['C#CO', 'CC']

EI-MS matched molecular weight (relative abundance: 87.67%) for C#CO

NN/MS matches corresponding with tox GHS classification highlight in Bold font.

GHS hazard classification: none of the hazards selected for correlation

EI-MS matched molecular weight (relative abundance: 38.46%) for CC

GHS hazard classification: none of the hazards selected for correlation

NN-rank 23: ['OCC1CC1']

NN-rank 24: ['C=C(O)CC']

NN-rank 25: ['CC#CCO']

NN/MS matches corresponding with tox GHS classification highlight in Bold font.

164. n-Hexanol: CCCCCCO

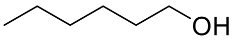

Summary Report of GHS Classification for NN/MS Matches:

Acute Toxic	Health Hazard	Irritant
—OH	—OH	=
=O	=O	=O

NN-rank 1: ['C#CCCCC', 'O']

NN-rank 2: ['C=CCCCC', 'O']

NN-rank 3: ['CCCCCC', 'O']

NN-rank 4: ['C', 'CCCCCO']

EI-MS matched molecular weight (relative abundance: 11.61%) for C

GHS hazard classification: none of the hazards selected for correlation

NN-rank 5: ['CCCCCC=O']

NN-rank 6: ['CCCCC', 'CO']

EI-MS matched molecular weight (relative abundance: 51.75%) for CO

GHS hazard classification: Acute Toxic, Health Hazard

NN-rank 7: ['CC', 'CCCCO']

EI-MS matched molecular weight (relative abundance: 46.44%) for CC

GHS hazard classification: none of the hazards selected for correlation

NN-rank 8: ['CCCC', 'CCO']

EI-MS matched molecular weight (relative abundance: 9.51%) for CCCC

GHS hazard classification: none of the hazards selected for correlation

EI-MS matched molecular weight (relative abundance: 5.81%) for CCO

GHS hazard classification: none of the hazards selected for correlation

NN-rank 9: ['C#C', 'CCCC', 'O']

EI-MS matched molecular weight (relative abundance: 9.51%) for CCCC

GHS hazard classification: none of the hazards selected for correlation

NN-rank 10: ['CCCCC#CO']

NN-rank 11: ['C=C', 'CCCC', 'O']

EI-MS matched molecular weight (relative abundance: 49.84%) for C=C

GHS hazard classification: Irritant

EI-MS matched molecular weight (relative abundance: 9.51%) for CCCC

GHS hazard classification: none of the hazards selected for correlation

NN/MS matches corresponding with tox GHS classification highlight in Bold font.

NN-rank 12: ['CCCCC(C)O']

NN-rank 13: ['CCCCC=CO']

NN-rank 14: ['C', 'CCCCC', 'O']

EI-MS matched molecular weight (relative abundance: 11.61%) for C

GHS hazard classification: none of the hazards selected for correlation

NN-rank 15: ['CCC', 'CCCO']

EI-MS matched molecular weight (relative abundance: 83.17%) for CCC

GHS hazard classification: none of the hazards selected for correlation

NN-rank 16: ['CCCCCCO']

NN-rank 17: ['C1CCCOCC1']

NN-rank 18: ['CCCCC1CO1']

NN-rank 19: ['CCCCCOC']

NN-rank 20: ['CCCC=CCO']

NN-rank 21: ['C=O', 'CCCCC']

EI-MS matched molecular weight (relative abundance: 46.44%) for C=O

GHS hazard classification: Acute Toxic, Health Hazard, Irritant

NN-rank 22: ['OCC1CCCC1']

NN-rank 23: ['C=CCCC', 'CO']

EI-MS matched molecular weight (relative abundance: 23.92%) for C=CCCC

GHS hazard classification: none of the hazards selected for correlation

EI-MS matched molecular weight (relative abundance: 51.75%) for CO

GHS hazard classification: Acute Toxic, Health Hazard

NN-rank 24: ['C#CO', 'CCCC']

EI-MS matched molecular weight (relative abundance: 59.05%) for C#CO

GHS hazard classification: none of the hazards selected for correlation

EI-MS matched molecular weight (relative abundance: 9.51%) for CCCC

GHS hazard classification: none of the hazards selected for correlation

NN-rank 25: ['C=C(O)CCCC']

NN/MS matches corresponding with tox GHS classification highlight in Bold font.

165. n-Octanal: CCCCCCCC=O

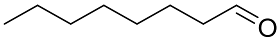

Summary Report of GHS Classification for NN/MS Matches:

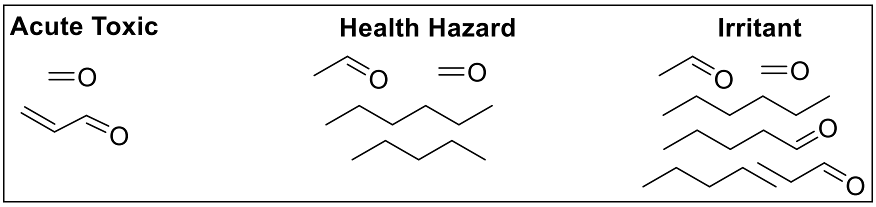

NN-rank 1: ['CCCCCC=CC=O']

NN-rank 2: ['C', 'CCCCCCC=O']

NN-rank 3: ['CCCCCCCCO']

NN-rank 4: ['CC', 'CCCCCC=O']

EI-MS matched molecular weight (relative abundance: 45.89%) for CC

GHS hazard classification: none of the hazards selected for correlation

NN-rank 5: ['C=O', 'C=CCCCCC']

EI-MS matched molecular weight (relative abundance: 45.89%) for C=O

GHS hazard classification: Acute Toxic, Health Hazard, Irritant

NN-rank 6: ['C=O', 'CCCCCCC']

EI-MS matched molecular weight (relative abundance: 45.89%) for C=O

GHS hazard classification: Acute Toxic, Health Hazard, Irritant

NN-rank 7: ['CCC', 'CCCCC=O']

EI-MS matched molecular weight (relative abundance: 99.99%) for CCC

GHS hazard classification: none of the hazards selected for correlation

EI-MS matched molecular weight (relative abundance: 18.79%) for CCCCC=O

GHS hazard classification: Irritant

NN-rank 8: ['CC=O', 'CCCCCC']

EI-MS matched molecular weight (relative abundance: 99.99%) for CC=O

GHS hazard classification: Health Hazard, Irritant

EI-MS matched molecular weight (relative abundance: 18.79%) for CCCCCC

GHS hazard classification: Health Hazard, Irritant

NN-rank 9: ['CCCCC=CCC=O']

NN-rank 10: ['CCCCCCC=C=O']

NN-rank 11: ['CCCC', 'CCCC=O']

NN/MS matches corresponding with tox GHS classification highlight in Bold font.

EI-MS matched molecular weight (relative abundance: 51.39%) for CCCC

GHS hazard classification: none of the hazards selected for correlation

EI-MS matched molecular weight (relative abundance: 10.29%) for CCCC=O

GHS hazard classification: none of the hazards selected for correlation

NN-rank 12: ['CCC=O', 'CCCCC']

EI-MS matched molecular weight (relative abundance: 51.39%) for CCC=O

GHS hazard classification: Irritant

EI-MS matched molecular weight (relative abundance: 10.29%) for CCCCC

GHS hazard classification: Health Hazard, Irritant

NN-rank 13: ['O=CCC1CCCCC1']

NN-rank 14: ['CCCCCCCC', 'O']

NN-rank 15: ['C=CCCCCCC', 'O']

NN-rank 16: ['CCCC=CCCC=O']

NN-rank 17: ['CCCCCCC=CO']

NN-rank 18: ['CCCCC(C)CC=O']

NN-rank 19: ['O=C1CCCCCCC1']

NN-rank 20: ['OC1CCCCCCC1']

NN-rank 21: ['C1CCCCOCCC1']

NN-rank 22: ['O=CC=C1CCCCC1']

NN-rank 23: ['C=CC=O', 'CCCCC']

EI-MS matched molecular weight (relative abundance: 50.99%) for C=CC=O

GHS hazard classification: Acute Toxic

EI-MS matched molecular weight (relative abundance: 10.29%) for CCCCC

GHS hazard classification: Health Hazard, Irritant

NN-rank 24: ['CCCCCCC(C)=O']

NN-rank 25: ['CCCCCC=C=C=O']

NN/MS matches corresponding with tox GHS classification highlight in Bold font.

166. n-Propanol: CCCO

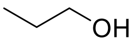

Summary Report of GHS Classification for NN/MS Matches:

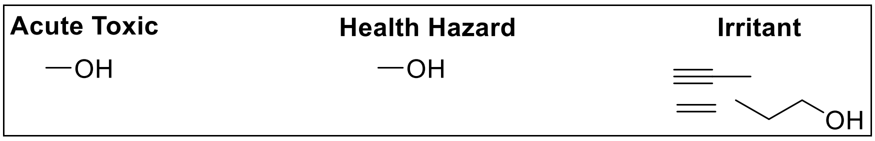

NN-rank 1: ['CCC', 'O']

NN-rank 2: ['C#CC', 'O']

EI-MS matched molecular weight (relative abundance: 6.58%) for C#CC

GHS hazard classification: Irritant

NN-rank 3: ['C=CC', 'O']

EI-MS matched molecular weight (relative abundance: 8.54%) for C=CC

GHS hazard classification: none of the hazards selected for correlation

NN-rank 4: ['CC#CO']

NN-rank 5: ['C', 'CCO']

NN-rank 6: ['C#CCO']

NN-rank 7: ['CCC=O']

NN-rank 8: ['C1CC1', 'O']

EI-MS matched molecular weight (relative abundance: 8.54%) for C1CC1

GHS hazard classification: none of the hazards selected for correlation

NN-rank 9: ['CC', 'CO']

EI-MS matched molecular weight (relative abundance: 17.66%) for CC

GHS hazard classification: none of the hazards selected for correlation

EI-MS matched molecular weight (relative abundance: 99.99%) for CO

GHS hazard classification: Acute Toxic, Health Hazard

NN-rank 10: ['C', 'C#C', 'O']

NN-rank 11: ['C', 'CC', 'O']

EI-MS matched molecular weight (relative abundance: 17.66%) for CC

GHS hazard classification: none of the hazards selected for correlation

NN-rank 12: ['CC=CO']

NN-rank 13: ['C=CCO']

NN-rank 14: ['C', 'C=C', 'O']

EI-MS matched molecular weight (relative abundance: 16.28%) for C=C

NN/MS matches corresponding with tox GHS classification highlight in Bold font.

GHS hazard classification: Irritant

NN-rank 15: ['C', 'C#CO']

EI-MS matched molecular weight (relative abundance: 8.54%) for C#CO

GHS hazard classification: none of the hazards selected for correlation

NN-rank 16: ['OC1CC1']

NN-rank 17: ['CCCO']

EI-MS matched molecular weight (relative abundance: 10.93%) for CCCO

GHS hazard classification: Irritant

NN-rank 18: ['CC1CO1']

NN-rank 19: ['CC(C)O']

EI-MS matched molecular weight (relative abundance: 10.93%) for CC(C)O

GHS hazard classification: none of the hazards selected for correlation

NN-rank 20: ['C1#CC1', 'O']

NN-rank 21: ['C#C', 'CO']

EI-MS matched molecular weight (relative abundance: 99.99%) for CO

GHS hazard classification: Acute Toxic, Health Hazard

NN-rank 22: ['C1COC1']

NN-rank 23: ['C1#CC1', 'O']

NN-rank 24: ['C1#CC1', 'O']

NN-rank 25: ['C', 'C=CO']

NN/MS matches corresponding with tox GHS classification highlight in Bold font.

167. Neral: CC(=CCC/C(=C\C=O)/C)C

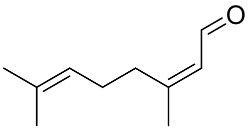

Summary Report of GHS Classification for NN/MS Matches:

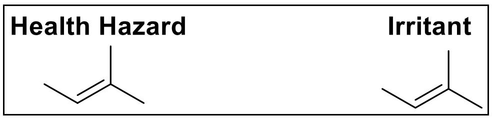

NN-rank 1: ['CC(C)=CCCC(C)CC=O']

NN-rank 2: ['C', 'CC=CCC/C(C)=C\\C=O']

NN-rank 3: ['C', 'CC=CCC/C(C)=C\\C=O']

NN-rank 4: ['CC(C)=C=CC/C(C)=C\\C=O']

NN-rank 5: ['CC(C)=CCC/C(C)=C\\CO']

NN-rank 6: ['CC(C)=CC=O', 'CC=C(C)C']

EI-MS matched molecular weight (relative abundance: 85.0%) for CC=C(C)C

GHS hazard classification: Health Hazard, Irritant

NN-rank 7: ['CC(C)=CCC=C(C)CC=O']

NN-rank 8: ['C', 'C', 'C=CCC/C(C)=C\\C=O']

NN-rank 9: ['C=C(CC=O)CCC=C(C)C']

NN-rank 10: ['C/C(=C/C=O)CCCC(C)C']

NN-rank 11: ['CC(C)=CC=C/C(C)=C\\C=O']

NN-rank 12: ['C', 'CCCCC/C(C)=C\\C=O']

NN-rank 13: ['C', 'CCCCC/C(C)=C\\C=O']

NN-rank 14: ['C=C(C)CCC/C(C)=C\\C=O']

NN-rank 15: ['C=C(C)CCC/C(C)=C\\C=O']

NN-rank 16: ['CCC', 'CCC/C(C)=C\\C=O']

NN-rank 17: ['CC(=C=C=O)CCC=C(C)C']

NN-rank 18: ['C', 'C=C=CCC/C(C)=C\\C=O']

NN-rank 19: ['C', 'C=C=CCC/C(C)=C\\C=O']

NN-rank 20: ['CC(C)=CC=O', 'C=C=C(C)C']

NN-rank 21: ['C/C(=C/C=O)CC=CC(C)C']

NN-rank 22: ['CC(C)=CCCC(C)C=C=O']

NN-rank 23: ['C', 'C', 'CCCC/C(C)=C\\C=O']

NN/MS matches corresponding with tox GHS classification highlight in Bold font.

NN-rank 24: ['C/C=C(/C)CCC=C(C)C', 'O']

NN-rank 25: ['C=CC/C(C)=C\\C=O', 'CCC']

NN/MS matches corresponding with tox GHS classification highlight in Bold font.

168. Nerol: CC(=CCC/C(=C\CO)/C)C

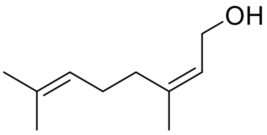

Summary Report of GHS Classification for NN/MS Matches:

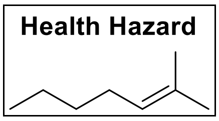

NN-rank 1: ['CC(C)=CCC/C(C)=C\\C=O']

NN-rank 2: ['CC(C)=CCCC(C)CCO']

NN-rank 3: ['C', 'CC=CCC/C(C)=C\\CO']

NN-rank 4: ['C', 'CC=CCC/C(C)=C\\CO']

NN-rank 5: ['C=C(CCO)CCC=C(C)C']

NN-rank 6: ['C/C(=C/CO)CCCC(C)C']

NN-rank 7: ['CC(C)=CCC=C(C)CCO']

NN-rank 8: ['C=C(C)CCC/C(C)=C\\CO']

NN-rank 9: ['C=C(C)CCC/C(C)=C\\CO']

NN-rank 10: ['C', 'CCCCC/C(C)=C\\CO']

NN-rank 11: ['C', 'CCCCC/C(C)=C\\CO']

NN-rank 12: ['C', 'C', 'C=CCC/C(C)=C\\CO']

NN-rank 13: ['CC(C)=C=CC/C(C)=C\\CO']

NN-rank 14: ['CCCCC=C(C)C', 'CCO']

EI-MS matched molecular weight (relative abundance: 7.31%) for CCCCC=C(C)C

GHS hazard classification: Health Hazard

NN-rank 15: ['C', 'CC(C)=CCCC=CCO']

NN-rank 16: ['C/C1=C/COC(C)(C)CCC1']

NN-rank 17: ['CCC', 'CCC/C(C)=C\\CO']

EI-MS matched molecular weight (relative abundance: 8.01%) for CCC

GHS hazard classification: none of the hazards selected for correlation

NN-rank 18: ['C/C=C(/C)CCC=C(C)C', 'O']

NN-rank 19: ['C', 'CC1=CCC/C(C)=C\\CO1']

NN-rank 20: ['C', 'CC1=CCC/C(C)=C\\CO1']

NN-rank 21: ['C', 'CC(C)=CCCCCCO']

NN/MS matches corresponding with tox GHS classification highlight in Bold font.

NN-rank 22: ['C', 'C', 'CCCC/C(C)=C\\CO']

NN-rank 23: ['CC=CCC=C(C)C', 'CCO']

NN-rank 24: ['C/C(=C/CO)CC=CC(C)C']

NN-rank 25: ['CC1(C)CCCC1(C)CCO']

169. Neryl Acetate: CC(=CCC/C(=C\COC(=O)C)/C)C

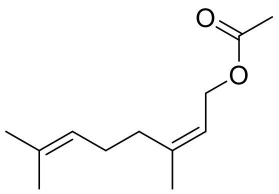

Summary Report of GHS Classification for NN/MS Matches:

Health Hazard	Irritant
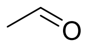	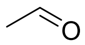

NN-rank 1: ['CC(C)=CCC/C(C)=C\\CO', 'CC=O']

EI-MS matched molecular weight (relative abundance: 36.23%) for CC=O

GHS hazard classification: Health Hazard, Irritant

NN-rank 2: ['CC(=O)OC/C=C(/C)CCCC(C)C']

NN-rank 3: ['C=C=C(C)CCC=C(C)C', 'CC(=O)O']

NN-rank 4: ['CC(=O)OCCC(C)CCC=C(C)C']

NN-rank 5: ['CC(C)=CCC/C(C)=C\\C=O', 'CC=O']

EI-MS matched molecular weight (relative abundance: 36.23%) for CC=O

GHS hazard classification: Health Hazard, Irritant

NN-rank 6: ['CC1=CCC(C)(C)CCC1', 'CC(=O)O']

NN-rank 7: ['C=CC(C)CCC=C(C)C', 'CC(=O)O']

NN-rank 8: ['C/C=C(/C)CCC=C(C)C', 'CC(=O)O']

NN-rank 9: ['CC(=O)OC=C=C(C)CCC=C(C)C']

NN-rank 10: ['CC(=O)OC=CC(C)CCC=C(C)C']

NN-rank 11: ['CC(=O)OCCC(C)=CCC=C(C)C']

NN-rank 12: ['C', 'CCCCC/C(C)=C\\COC(C)=O']

NN-rank 13: ['C', 'CCCCC/C(C)=C\\COC(C)=O']

NN-rank 14: ['CC(=O)OC1C=C(C)CCCC1(C)C']

NN-rank 15: ['CC1=CC(=O)C(C)(C)CCC1', 'CC=O']

EI-MS matched molecular weight (relative abundance: 36.23%) for CC=O

GHS hazard classification: Health Hazard, Irritant

NN-rank 16: ['CC(=C=C=O)CCC=C(C)C', 'CC=O']

EI-MS matched molecular weight (relative abundance: 36.23%) for CC=O

GHS hazard classification: Health Hazard, Irritant

NN/MS matches corresponding with tox GHS classification highlight in Bold font.

NN-rank 17: ['C/C1=C/COC(=O)C=C(C(C)C)CC1']

NN-rank 18: ['C=CC(C)=CCC=C(C)C', 'CC(=O)O']

NN-rank 19: ['CC(=O)O', 'CC1=C=CC(C)(C)CCC1']

NN-rank 20: ['C', 'CC=CCC/C(C)=C\\COC(C)=O']

NN-rank 21: ['C', 'CC=CCC/C(C)=C\\COC(C)=O']

NN-rank 22: ['CC(C)=CCCC(C)C=C=O', 'CC=O']

EI-MS matched molecular weight (relative abundance: 36.23%) for CC=O

GHS hazard classification: Health Hazard, Irritant

NN-rank 23: ['C=C(C)CCC/C(C)=C\\COC(C)=O']

NN-rank 24: ['C=C(C)CCC/C(C)=C\\COC(C)=O']

NN-rank 25: ['CC(=O)OC/C=C(/C)CC=C=C(C)C']

NN/MS matches corresponding with tox GHS classification highlight in Bold font.

170. Tabanone: CC=CC=C1C(=CC(=O)CC1(C)C)C

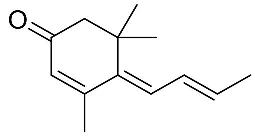

NN-rank 1: ['CC=CC=C1C(C)CC(=O)CC1(C)C']

NN-rank 2: ['CC=CC=C1C(C)=CC(O)CC1(C)C']

NN-rank 3: ['C', 'C=CC=C1C(C)=CC(=O)CC1(C)C']

EI-MS matched molecular weight (relative abundance: 54.0%) for C=CC=C1C(C)=CC(=O)CC1(C)C

GHS hazard classification: none of the hazards selected for correlation

NN-rank 4: ['CC=CC=CC(C)(C)CC(=O)C=CC']

NN-rank 5: ['C=CCC=C1C(C)=CC(=O)CC1(C)C']

NN-rank 6: ['CCCC=C1C(C)=CC(=O)CC1(C)C']

NN-rank 7: ['C=C=CC=C1C(C)=CC(=O)CC1(C)C']

NN-rank 8: ['CC', 'CC1=CC(=O)C2=CC=C1C2(C)C']

NN-rank 9: ['C', 'CC=CC=C1C(C)=CC(=O)CC1C']

EI-MS matched molecular weight (relative abundance: 54.0%) for CC=CC=C1C(C)=CC(=O)CC1C

GHS hazard classification: unknown

NN-rank 10: ['C', 'CC=CC=C1C(C)=CC(=O)CC1C']

EI-MS matched molecular weight (relative abundance: 54.0%) for CC=CC=C1C(C)=CC(=O)CC1C

GHS hazard classification: unknown

NN-rank 11: ['CC1=CC(=O)CC(C)(C)C1', 'CC=CC']

EI-MS matched molecular weight (relative abundance: 32.0%) for CC=CC

GHS hazard classification: none of the hazards selected for correlation

NN-rank 12: ['CC=CC=C1C(C)=CCCC1(C)C', 'O']

EI-MS matched molecular weight (relative abundance: 54.0%) for CC=CC=C1C(C)=CCCC1(C)C

GHS hazard classification: none of the hazards selected for correlation

NN-rank 13: ['CC=C=C=C1C(C)=CC(=O)CC1(C)C']

NN-rank 14: ['C', 'CCC=C1C(C)=CC(=O)CC1(C)C']

NN-rank 15: ['CC=CC=CC(C)(C)CC(=O)CCC']

NN-rank 16: ['CC=CC=C1C(C)=CC(O)=CC1(C)C']

NN/MS matches corresponding with tox GHS classification highlight in Bold font.

NN-rank 17: ['CCC1=C2C(=O)C=C(C)C(=C1)C2(C)C']

NN-rank 18: ['CC', 'CC=C1C(C)=CC(=O)CC1(C)C']

NN-rank 19: ['CC=CC=C1C(C)=CC=CC1(C)C', 'O']

NN-rank 20: ['CC', 'C=C=C1C(C)=CC(=O)CC1(C)C']

NN-rank 21: ['CC=CCC1C(C)=CC(=O)CC1(C)C']

NN-rank 22: ['C', 'CC1=CC(=O)C2=C(C)C=C1C2(C)C']

NN-rank 23: ['C', 'CC=CC=C1C(C)=CC(=O)C=C1C']

NN-rank 24: ['C', 'CC=CC=C1C(C)=CC(=O)C=C1C']

NN-rank 25: ['C=CC1=C2C(=O)C=C(C)C(=C1)C2(C)C']

NN/MS matches corresponding with tox GHS classification highlight in Bold font.

171. Octanoic Acid: CCCCCCCC(=O)O

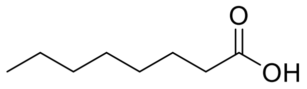

Summary Report of GHS Classification for NN/MS Matches:

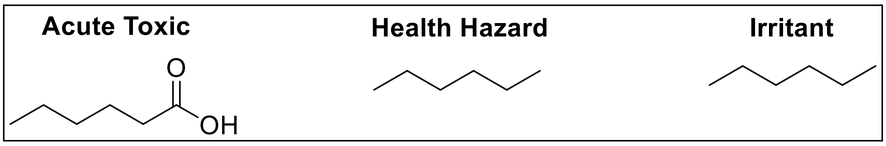

NN-rank 1: ['CCCCCCCC(=O)O']

NN-rank 2: ['C', 'CCCCCCC(=O)O']

NN-rank 3: ['O=C1CCCCCCCO1']

NN-rank 4: ['CCCCCCCCO', 'O']

NN-rank 5: ['CCCCCCCCO', 'O']

NN-rank 6: ['CC', 'CCCCCC(=O)O']

EI-MS matched molecular weight (relative abundance: 14.6%) for CC

GHS hazard classification: none of the hazards selected for correlation

EI-MS matched molecular weight (relative abundance: 9.8%) for CCCCCC(=O)O

GHS hazard classification: Acute Toxic

NN-rank 7: ['CCCCCCC(=O)OC']

NN-rank 8: ['CC(=O)O', 'CCCCCC']

EI-MS matched molecular weight (relative abundance: 18.7%) for CCCCCC

GHS hazard classification: Health Hazard, Irritant

NN-rank 9: ['CCCCCCC', 'O=CO']

EI-MS matched molecular weight (relative abundance: 15.0%) for O=CO

GHS hazard classification: none of the hazards selected for correlation

NN-rank 10: ['CCC', 'CCCCC(=O)O']

EI-MS matched molecular weight (relative abundance: 42.7%) for CCC

GHS hazard classification: none of the hazards selected for correlation

EI-MS matched molecular weight (relative abundance: 23.7%) for CCCCC(=O)O

GHS hazard classification: none of the hazards selected for correlation

NN-rank 11: ['CCCCCCCC=O', 'O']

NN-rank 12: ['CCCC', 'CCCC(=O)O']

EI-MS matched molecular weight (relative abundance: 10.6%) for CCCC

GHS hazard classification: none of the hazards selected for correlation

NN/MS matches corresponding with tox GHS classification highlight in Bold font.

EI-MS matched molecular weight (relative abundance: 12.5%) for CCCC(=O)O

GHS hazard classification: none of the hazards selected for correlation

NN-rank 13: ['CCCCCCCC=O', 'O']

NN-rank 14: ['CCCCCC=CC(=O)O']

NN-rank 15: ['CCCCCCC', 'O=CO']

EI-MS matched molecular weight (relative abundance: 15.0%) for O=CO

GHS hazard classification: none of the hazards selected for correlation

NN-rank 16: ['CCCC=CCCC(=O)O']

NN-rank 17: ['CCCCC=CCC(=O)O']

NN-rank 18: ['CCC(=O)O', 'CCCCC']

EI-MS matched molecular weight (relative abundance: 62.79%) for CCC(=O)O

GHS hazard classification: none of the hazards selected for correlation

NN-rank 19: ['OCCCCCCCCO']

NN-rank 20: ['CCCCCCC=CO', 'O']

NN-rank 21: ['CCCCCCCC', 'O', 'O']

NN-rank 22: ['CCCCCCC=C=O', 'O']

NN-rank 23: ['O=CCCCCCCCO']

NN-rank 24: ['C=CCCCCCC', 'O', 'O']

NN-rank 25: ['C1CCCCOCCC1', 'O']

NN/MS matches corresponding with tox GHS classification highlight in Bold font.

172. Octanol: CCCCCCCCO

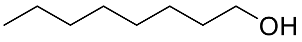

Summary Report of GHS Classification for NN/MS Matches:

Acute Toxic	Health Hazard	Irritant
—OH<br>=O	—OH =O	=<br>=O

NN-rank 1: ['C#CCCCCCC', 'O']

NN-rank 2: ['C=CCCCCCC', 'O']

NN-rank 3: ['C', 'CCCCCCCO']

NN-rank 4: ['CCCCCCCC', 'O']

NN-rank 5: ['CCCCCCCC=O']

NN-rank 6: ['CCCCCCC', 'CO']

EI-MS matched molecular weight (relative abundance: 27.59%) for CO

GHS hazard classification: Acute Toxic, Health Hazard

NN-rank 7: ['CC', 'CCCCCCO']

EI-MS matched molecular weight (relative abundance: 34.89%) for CC

GHS hazard classification: none of the hazards selected for correlation

NN-rank 8: ['CCCCCCC#CO']

NN-rank 9: ['C#C', 'CCCCCC', 'O']

NN-rank 10: ['CCCCCC', 'CCO']

NN-rank 11: ['CCCCCCC(C)O']

NN-rank 12: ['CCC', 'CCCCCO']

EI-MS matched molecular weight (relative abundance: 74.99%) for CCC

GHS hazard classification: none of the hazards selected for correlation

NN-rank 13: ['CCCCCCCCO']

NN-rank 14: ['CCCCCCC=CO']

NN-rank 15: ['C', 'CCCCCCC', 'O']

NN-rank 16: ['C1CCCCOCCC1']

NN-rank 17: ['CCCCCCCOC']

NN-rank 18: ['CCCCCCC1CO1']

NN-rank 19: ['C=C', 'CCCCCC', 'O']

EI-MS matched molecular weight (relative abundance: 23.19%) for C=C

GHS hazard classification: Irritant

NN/MS matches corresponding with tox GHS classification highlight in Bold font.

NN-rank 20: ['C1CCCCCC1', 'CO']

EI-MS matched molecular weight (relative abundance: 27.59%) for CO

GHS hazard classification: Acute Toxic, Health Hazard

NN-rank 21: ['CCCC', 'CCCCO']

EI-MS matched molecular weight (relative abundance: 38.59%) for CCCC

GHS hazard classification: none of the hazards selected for correlation

NN-rank 22: ['OCC1CCCCCC1']

NN-rank 23: ['CCCCCC(C)CO']

NN-rank 24: ['C=O', 'CCCCCCC']

EI-MS matched molecular weight (relative abundance: 34.89%) for C=O

GHS hazard classification: Acute Toxic, Health Hazard, Irritant

NN-rank 25: ['CC1CCCCC1', 'CO']

EI-MS matched molecular weight (relative abundance: 27.59%) for CO

GHS hazard classification: Acute Toxic, Health Hazard

NN/MS matches corresponding with tox GHS classification highlight in Bold font.

173. Oleic Acid: CCCCCCCC/C=C\CCCCCCCC(=O)O

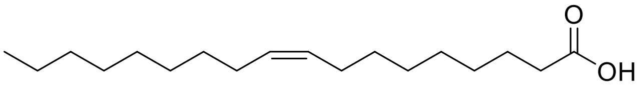

NN-rank 1: ['C', 'CCCCCCC/C=C\\CCCCCCCC(=O)O']

NN-rank 2: ['CCCCCCCCCCCCCCCCCC(=O)O']

NN-rank 3: ['CCCCCCCC/C=C\\CCCCCCCC(=O)O']

NN-rank 4: ['CCCCCCCC=CCCCCCCCCC(=O)O']

NN-rank 5: ['CCCCCCCCCC=CCCCCCCC(=O)O']

NN-rank 6: ['CCCCCCCC/C=C\\CCCCCCCCO', 'O']

NN-rank 7: ['CCCCCCCCC', 'CCCCCCCCC(=O)O']

NN-rank 8: ['CCCCCCCC/C=C\\CCCCCCCCO', 'O']

NN-rank 9: ['CC', 'CCCCCC/C=C\\CCCCCCCC(=O)O']

EI-MS matched molecular weight (relative abundance: 50.49%) for CC

GHS hazard classification: none of the hazards selected for correlation

NN-rank 10: ['CCCCCCCC=C=CCCCCCCCC(=O)O']

NN-rank 11: ['CCCCCCCCC=C=CCCCCCCC(=O)O']

NN-rank 12: ['CCCCCCC/C=C\\CCCCCCCC', 'O=CO']

EI-MS matched molecular weight (relative abundance: 12.3%) for O=CO

GHS hazard classification: none of the hazards selected for correlation

NN-rank 13: ['CCCCCCCC/C=C1\\CCCCCCCC(=O)O1']

NN-rank 14: ['CCCCCCCCCC1CCCCCCCC(=O)O1']

NN-rank 15: ['CCCCCCCC/C1=C\\CCCCCCCC(=O)O1']

NN-rank 16: ['CCCCCCCC/C=C1\\CCCCCCCC(=O)O1']

NN-rank 17: ['CCCCCCCCC1CCCCCCCCC(=O)O1']

NN-rank 18: ['CCCCCCCCCC1CCCCCCCC(=O)O1']

NN-rank 19: ['CCCCCCCC/C(O)=C\\CCCCCCCCO']

NN-rank 20: ['CCCCCCCC/C=C(/O)CCCCCCCCO']

NN-rank 21: ['C=CCCCCCCCC(=O)O', 'CCCCCCCC']

NN-rank 22: ['CCCCCCCC(=O)O', 'C=CCCCCCCCC']

NN-rank 23: ['C=CCCCCCCC', 'CCCCCCCCC(=O)O']

NN-rank 24: ['C=CCCCCCCC(=O)O', 'CCCCCCCCC']

NN-rank 25: ['CCCCCCCCC', 'O=C1CCCCCCCCO1']

NN/MS matches corresponding with tox GHS classification highlight in Bold font.

174. p-Cymene: CC1=CC=C(C=C1)C(C)C

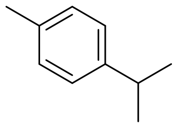

Summary Report of GHS Classification for NN/MS Matches:

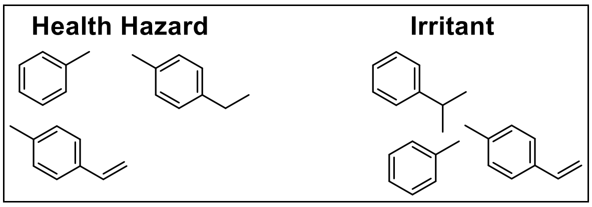

NN-rank 1: ['C=C(C)c1ccc(C)cc1']

NN-rank 2: ['C=C(C)c1ccc(C)cc1']

NN-rank 3: ['C', 'C=Cc1ccc(C)cc1']

EI-MS matched molecular weight (relative abundance: 9.19%) for C=Cc1ccc(C)cc1

GHS hazard classification: Irritant

NN-rank 4: ['C', 'C=Cc1ccc(C)cc1']

EI-MS matched molecular weight (relative abundance: 9.19%) for C=Cc1ccc(C)cc1

GHS hazard classification: Irritant

NN-rank 5: ['CCC', 'Cc1ccccc1']

EI-MS matched molecular weight (relative abundance: 15.89%) for Cc1ccccc1

GHS hazard classification: Health Hazard, Irritant

NN-rank 6: ['C=CC', 'Cc1ccccc1']

EI-MS matched molecular weight (relative abundance: 5.99%) for C=CC

GHS hazard classification: none of the hazards selected for correlation

EI-MS matched molecular weight (relative abundance: 15.89%) for Cc1ccccc1

GHS hazard classification: Health Hazard, Irritant

NN-rank 7: ['C=CC', 'Cc1ccccc1']

EI-MS matched molecular weight (relative abundance: 5.99%) for C=CC

GHS hazard classification: none of the hazards selected for correlation

EI-MS matched molecular weight (relative abundance: 15.89%) for Cc1ccccc1

GHS hazard classification: Health Hazard, Irritant

NN-rank 8: ['C', 'CCc1ccc(C)cc1']

EI-MS matched molecular weight (relative abundance: 99.99%) for CCc1ccc(C)cc1

NN/MS matches corresponding with tox GHS classification highlight in Bold font.

GHS hazard classification: Health Hazard

NN-rank 9: ['C', 'CCc1ccc(C)cc1']

EI-MS matched molecular weight (relative abundance: 99.99%) for CCc1ccc(C)cc1

GHS hazard classification: Health Hazard

NN-rank 10: ['C=C=C', 'Cc1ccccc1']

EI-MS matched molecular weight (relative abundance: 15.89%) for Cc1ccccc1

GHS hazard classification: Health Hazard, Irritant

NN-rank 11: ['C', 'C=C', 'Cc1ccccc1']

EI-MS matched molecular weight (relative abundance: 15.89%) for Cc1ccccc1

GHS hazard classification: Health Hazard, Irritant

NN-rank 12: ['C', 'C=C', 'Cc1ccccc1']

EI-MS matched molecular weight (relative abundance: 15.89%) for Cc1ccccc1

GHS hazard classification: Health Hazard, Irritant

NN-rank 13: ['C', 'CC(C)c1ccccc1']

EI-MS matched molecular weight (relative abundance: 99.99%) for CC(C)c1ccccc1

GHS hazard classification: Irritant

NN-rank 14: ['C', 'C', 'Cc1ccc(C)cc1']

NN-rank 15: ['CC1=Cc2cc(C)ccc21']

NN-rank 16: ['CC1=Cc2cc(C)ccc21']

NN-rank 17: ['CC1=Cc2cc(C)ccc21']

NN-rank 18: ['CC1=Cc2cc(C)ccc21']

NN-rank 19: ['CC1(C)Cc2ccc1cc2']

NN-rank 20: ['Cc1ccc2c(c1)CC2C']

NN-rank 21: ['Cc1ccc2c(c1)CC2C']

NN-rank 22: ['Cc1ccc2c(c1)CC2C']

NN-rank 23: ['Cc1ccc2c(c1)CC2C']

NN-rank 24: ['Cc1ccc2c(c1)C2(C)C']

NN-rank 25: ['Cc1ccc2c(c1)C2(C)C']

NN/MS matches corresponding with tox GHS classification highlight in Bold font.

175. Piperonal: C1OC2=C(O1)C=C(C=C2)C=O

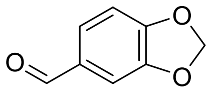

Summary Report of GHS Classification for NN/MS Matches:

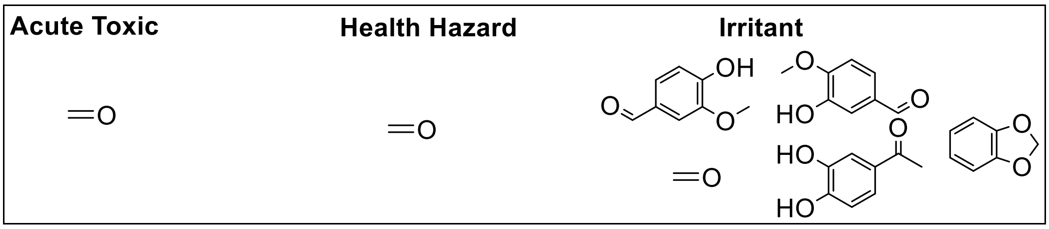

NN-rank 1: ['c1ccc2c(c1)OCO2', 'C=O']

EI-MS matched molecular weight (relative abundance: 26.79%) for c1ccc2c(c1)OCO2

GHS hazard classification: Irritant

EI-MS matched molecular weight (relative abundance: 5.39%) for C=O

GHS hazard classification: Acute Toxic, Health Hazard, Irritant

NN-rank 2: ['OCc1ccc2c(c1)OCO2']

EI-MS matched molecular weight (relative abundance: 8.79%) for OCc1ccc2c(c1)OCO2

GHS hazard classification: none of the hazards selected for correlation

NN-rank 3: ['COc1cc(C=O)ccc1O']

EI-MS matched molecular weight (relative abundance: 8.79%) for COc1cc(C=O)ccc1O

GHS hazard classification: Irritant

NN-rank 4: ['COc1ccc(C=O)cc1O']

EI-MS matched molecular weight (relative abundance: 8.79%) for COc1ccc(C=O)cc1O

GHS hazard classification: Irritant

NN-rank 5: ['C', 'O=Cc1ccc(O)c(O)c1']

NN-rank 6: ['OC1c2ccc3c(c2)OC1O3']

EI-MS matched molecular weight (relative abundance: 99.99%) for OC1c2ccc3c(c2)OC1O3

GHS hazard classification: unknown

NN-rank 7: ['O=C1c2ccc3c(c2)OC1O3']

NN-rank 8: ['O=Cc1cccc(OCO)c1']

NN/MS matches corresponding with tox GHS classification highlight in Bold font.

EI-MS matched molecular weight (relative abundance: 8.79%) for O=Cc1cccc(OCO)c1

GHS hazard classification: none of the hazards selected for correlation

NN-rank 9: ['Oc1ccc2cc1OCC2O']

EI-MS matched molecular weight (relative abundance: 8.79%) for Oc1ccc2cc1OCC2O

GHS hazard classification: unknown

NN-rank 10: ['O=C1COc2cc1ccc2O']

EI-MS matched molecular weight (relative abundance: 99.99%) for O=C1COc2cc1ccc2O

GHS hazard classification: unknown

NN-rank 11: ['c1ccc2c(c1)OCO2', 'CO']

EI-MS matched molecular weight (relative abundance: 26.79%) for c1ccc2c(c1)OCO2

GHS hazard classification: Irritant

NN-rank 12: ['O=CC1Oc2ccccc2O1']

EI-MS matched molecular weight (relative abundance: 99.99%) for O=CC1Oc2ccccc2O1

GHS hazard classification: none of the hazards selected for correlation

NN-rank 13: ['O=C1COc2ccc1cc2O']

EI-MS matched molecular weight (relative abundance: 99.99%) for O=C1COc2ccc1cc2O

GHS hazard classification: unknown

NN-rank 14: ['Oc1cc2ccc1OCC2O']

EI-MS matched molecular weight (relative abundance: 8.79%) for Oc1cc2ccc1OCC2O

GHS hazard classification: unknown

NN-rank 15: ['O=Cc1ccc(OCO)cc1']

EI-MS matched molecular weight (relative abundance: 8.79%) for O=Cc1ccc(OCO)cc1

GHS hazard classification: none of the hazards selected for correlation

NN-rank 16: ['Cc1ccc2c(c1)OCO2', 'O']

NN-rank 17: ['O=Cc1cc2c3cc1C(O2)O3']

NN-rank 18: ['CC(=O)c1ccc(O)c(O)c1']

EI-MS matched molecular weight (relative abundance: 8.79%) for CC(=O)c1ccc(O)c(O)c1

GHS hazard classification: Irritant

NN-rank 19: ['O=Cc1cc2c3c(c1)C(O2)O3']

NN-rank 20: ['CC(O)c1ccc(O)c(O)c1']

NN-rank 21: ['OC1c2cc3c(c1c2)OCO3']

EI-MS matched molecular weight (relative abundance: 99.99%) for OC1c2cc3c(c1c2)OCO3

GHS hazard classification: unknown

NN-rank 22: ['c1cc2c3cc1CC(O2)O3', 'O']

NN-rank 23: ['OCC1Oc2ccccc2O1']

EI-MS matched molecular weight (relative abundance: 8.79%) for OCC1Oc2ccccc2O1

GHS hazard classification: none of the hazards selected for correlation

NN-rank 24: ['O=C1c2cc3c(c1c2)OCO3']

NN-rank 25: ['O=Cc1cc2c(O)cc1CO2']

EI-MS matched molecular weight (relative abundance: 99.99%) for O=Cc1cc2c(O)cc1CO2

GHS hazard classification: unknown

NN/MS matches corresponding with tox GHS classification highlight in Bold font.

176. Sorbic Acid: CC=CC=CC(=O)O

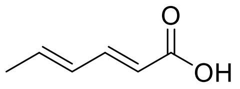

Summary Report of GHS Classification for NN/MS Matches:

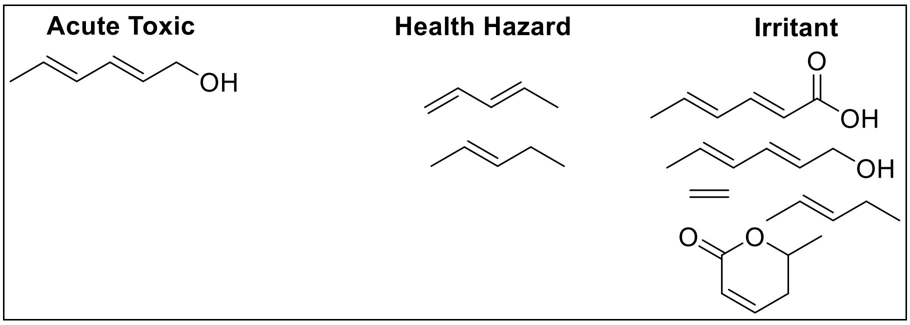

NN-rank 1: ['CC=CCCC(=O)O']

EI-MS matched molecular weight (relative abundance: 5.49%) for CC=CCCC(=O)O

GHS hazard classification: none of the hazards selected for correlation

NN-rank 2: ['C=C=CC=CC(=O)O']

NN-rank 3: ['C', 'C=CC=CC(=O)O']

EI-MS matched molecular weight (relative abundance: 99.99%) for C=CC=CC(=O)O

GHS hazard classification: none of the hazards selected for correlation

NN-rank 4: ['C=CCC=CC(=O)O']

EI-MS matched molecular weight (relative abundance: 8.29%) for C=CCC=CC(=O)O

GHS hazard classification: none of the hazards selected for correlation

NN-rank 5: ['CC=CC=CCO', 'O']

EI-MS matched molecular weight (relative abundance: 99.99%) for CC=CC=CCO

GHS hazard classification: Acute Toxic, Irritant

NN-rank 6: ['CCCC=CC(=O)O']

EI-MS matched molecular weight (relative abundance: 5.49%) for CCCC=CC(=O)O

GHS hazard classification: none of the hazards selected for correlation

NN-rank 7: ['CC=CC=CC=O', 'O']

EI-MS matched molecular weight (relative abundance: 6.99%) for CC=CC=CC=O

GHS hazard classification: none of the hazards selected for correlation

NN-rank 8: ['C', 'CCC=CC(=O)O']

NN-rank 9: ['CC=CC=CC(=O)O']

EI-MS matched molecular weight (relative abundance: 8.29%) for CC=CC=CC(=O)O

NN/MS matches corresponding with tox GHS classification highlight in Bold font.

GHS hazard classification: Irritant

NN-rank 10: ['C#CCC=CC(=O)O']

NN-rank 11: ['CC1=CC=CC1=O', 'O']

NN-rank 12: ['CC=CC=CCO', 'O']

EI-MS matched molecular weight (relative abundance: 99.99%) for CC=CC=CCO

GHS hazard classification: Acute Toxic, Irritant

NN-rank 13: ['C=CC(C)=CC(=O)O']

EI-MS matched molecular weight (relative abundance: 8.29%) for C=CC(C)=CC(=O)O

GHS hazard classification: none of the hazards selected for correlation

NN-rank 14: ['C', 'O', 'O=C1C=CC=C1']

NN-rank 15: ['Cc1cccc(=O)o1']

NN-rank 16: ['C=CC=CC', 'O=CO']

EI-MS matched molecular weight (relative abundance: 64.09%) for C=CC=CC

GHS hazard classification: Health Hazard

EI-MS matched molecular weight (relative abundance: 5.69%) for O=CO

GHS hazard classification: none of the hazards selected for correlation

NN-rank 17: ['C', 'O=c1cccco1']

EI-MS matched molecular weight (relative abundance: 6.99%) for O=c1cccco1

GHS hazard classification: none of the hazards selected for correlation

NN-rank 18: ['CC=CCC', 'O=CO']

EI-MS matched molecular weight (relative abundance: 15.39%) for CC=CCC

GHS hazard classification: Health Hazard, Irritant

EI-MS matched molecular weight (relative abundance: 5.69%) for O=CO

GHS hazard classification: none of the hazards selected for correlation

NN-rank 19: ['CC', 'CC=CC(=O)O']

NN-rank 20: ['O=C(O)C=C1C=CC1']

NN-rank 21: ['Cc1cccc(=O)o1']

NN-rank 22: ['O=C(O)C=CC1=CC1']

NN-rank 23: ['CC1CC=CC(=O)O1']

EI-MS matched molecular weight (relative abundance: 8.29%) for CC1CC=CC(=O)O1

GHS hazard classification: Irritant

NN-rank 24: ['O=C(O)CC1C=CC1']

EI-MS matched molecular weight (relative abundance: 8.29%) for O=C(O)CC1C=CC1

GHS hazard classification: unknown

NN-rank 25: ['C=C', 'CC=CC(=O)O']

NN/MS matches corresponding with tox GHS classification highlight in Bold font.

EI-MS matched molecular weight (relative abundance: 9.09%) for C=C

GHS hazard classification: Irritant

NN/MS matches corresponding with tox GHS classification highlight in Bold font.

177. Propenyl Guaethol: CCOC1=CC=C(/C=C/C)C=C1O

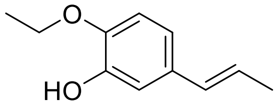

NN-rank 1: ['CCCc1ccc(OCC)c(O)c1']

EI-MS matched molecular weight (relative abundance: 12.0%) for CCCc1ccc(OCC)c(O)c1

GHS hazard classification: none of the hazards selected for correlation

NN-rank 2: ['C/C=C/c1ccc(O)c(O)c1', 'CC']

EI-MS matched molecular weight (relative abundance: 87.0%) for C/C=C/c1ccc(O)c(O)c1

GHS hazard classification: none of the hazards selected for correlation

NN-rank 3: ['CC', 'CC1Cc2ccc(c(O)c2)O1']

EI-MS matched molecular weight (relative abundance: 87.0%) for CC1Cc2ccc(c(O)c2)O1

GHS hazard classification: unknown

NN-rank 4: ['C', 'CCOc1ccc(CC)cc1O']

NN-rank 5: ['CCOc1ccc2cc1OC(C)C2']

NN-rank 6: ['CC1Cc2ccc(c(O)c2)OC1C']

NN-rank 7: ['CCOc1ccc(C2CC2)cc1O']

NN-rank 8: ['CCC(C)Cc1ccc(O)c(O)c1']

EI-MS matched molecular weight (relative abundance: 12.0%) for CCC(C)Cc1ccc(O)c(O)c1

GHS hazard classification: none of the hazards selected for correlation

NN-rank 9: ['C=CCc1ccc(OCC)c(O)c1']

NN-rank 10: ['C', 'C/C=C/c1ccc(OC)c(O)c1']

NN-rank 11: ['CC1=Cc2ccc(c(O)c2)O1', 'CC']

NN-rank 12: ['CCC1CC1c1ccc(O)c(O)c1']

NN-rank 13: ['CCC(CC)c1ccc(O)c(O)c1']

EI-MS matched molecular weight (relative abundance: 12.0%) for CCC(CC)c1ccc(O)c(O)c1

GHS hazard classification: none of the hazards selected for correlation

NN-rank 14: ['CCC1c2ccc(c(O)c2)OC1C']

NN-rank 15: ['CC', 'Oc1cc2ccc1OC1CC21']

NN/MS matches corresponding with tox GHS classification highlight in Bold font.

NN-rank 16: ['C', 'C=Cc1ccc(OCC)c(O)c1']

NN-rank 17: ['CCOC(C)Cc1cccc(O)c1']

EI-MS matched molecular weight (relative abundance: 12.0%) for CCOC(C)Cc1cccc(O)c1

GHS hazard classification: none of the hazards selected for correlation

NN-rank 18: ['CC1Oc2ccc(cc2O)C2CC12']

NN-rank 19: ['C', 'CC', 'Oc1cc2ccc1OCC2']

EI-MS matched molecular weight (relative abundance: 14.0%) for Oc1cc2ccc1OCC2

GHS hazard classification: unknown

NN-rank 20: ['C', 'CC1COc2ccc(cc2O)C1']

NN-rank 21: ['C', 'CC(C)Cc1ccc(O)c(O)c1']

NN-rank 22: ['CCOc1ccc(C2=CC2)cc1O']

NN-rank 23: ['CC/C(C)=C\\c1ccc(O)c(O)c1']

NN-rank 24: ['C/C1=C/c2ccc(c(O)c2)OC1C']

NN-rank 25: ['CCOc1ccc2cc1OC(C)=C2']

NN/MS matches corresponding with tox GHS classification highlight in Bold font.

178. Propionic Acid: CCC(=O)O

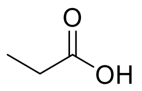

Summary Report of GHS Classification for NN/MS Matches:

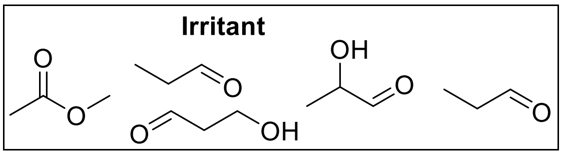

NN-rank 1: ['CCC(=O)O']

EI-MS matched molecular weight (relative abundance: 64.79%) for CCC(=O)O

GHS hazard classification: none of the hazards selected for correlation

NN-rank 2: ['O=C1CCO1']

NN-rank 3: ['CC1OC1=O']

NN-rank 4: ['CC', 'O=CO']

EI-MS matched molecular weight (relative abundance: 83.39%) for CC

GHS hazard classification: none of the hazards selected for correlation

EI-MS matched molecular weight (relative abundance: 90.09%) for O=CO

GHS hazard classification: none of the hazards selected for correlation

NN-rank 5: ['CCCO', 'O']

NN-rank 6: ['CCC=O', 'O']

EI-MS matched molecular weight (relative abundance: 46.6%) for CCC=O

GHS hazard classification: Irritant

NN-rank 7: ['CCCO', 'O']

NN-rank 8: ['C#CC(=O)O']

NN-rank 9: ['C', 'CC(=O)O']

NN-rank 10: ['CC', 'O=CO']

EI-MS matched molecular weight (relative abundance: 83.39%) for CC

GHS hazard classification: none of the hazards selected for correlation

EI-MS matched molecular weight (relative abundance: 90.09%) for O=CO

GHS hazard classification: none of the hazards selected for correlation

NN-rank 11: ['COC(C)=O']

EI-MS matched molecular weight (relative abundance: 64.79%) for COC(C)=O

GHS hazard classification: Irritant

NN/MS matches corresponding with tox GHS classification highlight in Bold font.

NN-rank 12: ['C', 'O=C1CO1']

EI-MS matched molecular weight (relative abundance: 46.6%) for O=C1CO1

GHS hazard classification: none of the hazards selected for correlation

NN-rank 13: ['C=CC(=O)O']

NN-rank 14: ['O=C1C#CO1']

NN-rank 15: ['CC=C(O)O']

EI-MS matched molecular weight (relative abundance: 64.79%) for CC=C(O)O

GHS hazard classification: none of the hazards selected for correlation

NN-rank 16: ['O', 'O=C1CC1']

EI-MS matched molecular weight (relative abundance: 26.6%) for O=C1CC1

GHS hazard classification: none of the hazards selected for correlation

NN-rank 17: ['CC=C=O', 'O']

EI-MS matched molecular weight (relative abundance: 26.6%) for CC=C=O

GHS hazard classification: none of the hazards selected for correlation

NN-rank 18: ['CCC=O', 'O']

EI-MS matched molecular weight (relative abundance: 46.6%) for CCC=O

GHS hazard classification: Irritant

NN-rank 19: ['CC1OC1=O']

NN-rank 20: ['O=CCCO']

EI-MS matched molecular weight (relative abundance: 64.79%) for O=CCCO

GHS hazard classification: Irritant

NN-rank 21: ['O=C1C=CO1']

NN-rank 22: ['CCC(O)O']

NN-rank 23: ['OC1CCO1']

EI-MS matched molecular weight (relative abundance: 64.79%) for OC1CCO1

GHS hazard classification: none of the hazards selected for correlation

NN-rank 24: ['CC(O)C=O']

EI-MS matched molecular weight (relative abundance: 64.79%) for CC(O)C=O

GHS hazard classification: Irritant

NN-rank 25: ['CC=CO', 'O']

EI-MS matched molecular weight (relative abundance: 46.6%) for CC=CO

GHS hazard classification: none of the hazards selected for correlation

NN/MS matches corresponding with tox GHS classification highlight in Bold font.

179. Propyl Acetate: CCCOC(=O)C

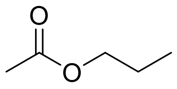

Summary Report of GHS Classification for NN/MS Matches:

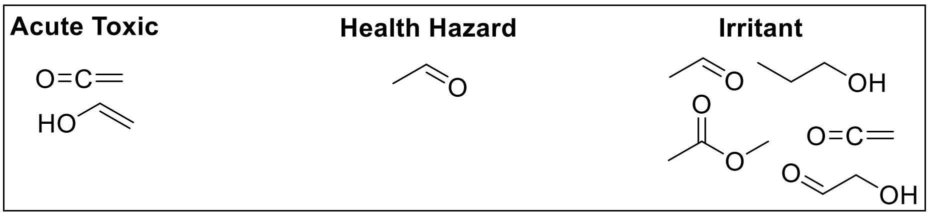

NN-rank 1: ['CC=O', 'CCCO']

EI-MS matched molecular weight (relative abundance: 99.99%) for CC=O

GHS hazard classification: Health Hazard, Irritant

EI-MS matched molecular weight (relative abundance: 5.09%) for CCCO

GHS hazard classification: Irritant

NN-rank 2: ['CC(=O)O', 'CCC']

EI-MS matched molecular weight (relative abundance: 5.09%) for CC(=O)O

GHS hazard classification: none of the hazards selected for correlation

EI-MS matched molecular weight (relative abundance: 99.99%) for CCC

GHS hazard classification: none of the hazards selected for correlation

NN-rank 3: ['CCCO', 'CCO']

EI-MS matched molecular weight (relative abundance: 5.09%) for CCCO

GHS hazard classification: Irritant

NN-rank 4: ['C=C=O', 'CCCO']

EI-MS matched molecular weight (relative abundance: 6.89%) for C=C=O

GHS hazard classification: Acute Toxic, Irritant

EI-MS matched molecular weight (relative abundance: 5.09%) for CCCO

GHS hazard classification: Irritant

NN-rank 5: ['C', 'CCOC(C)=O']

NN-rank 6: ['CC', 'COC(C)=O']

EI-MS matched molecular weight (relative abundance: 13.79%) for COC(C)=O

GHS hazard classification: Irritant

NN-rank 7: ['C1COC1', 'CC=O']

EI-MS matched molecular weight (relative abundance: 99.99%) for CC=O

NN/MS matches corresponding with tox GHS classification highlight in Bold font.

GHS hazard classification: Health Hazard, Irritant

NN-rank 8: ['CC=O', 'CCC=O']

EI-MS matched molecular weight (relative abundance: 99.99%) for CC=O

GHS hazard classification: Health Hazard, Irritant

NN-rank 9: ['CC(=O)CCCO']

NN-rank 10: ['CCCOC(C)O']

NN-rank 11: ['C1CC1', 'CC(=O)O']

EI-MS matched molecular weight (relative abundance: 6.89%) for C1CC1

GHS hazard classification: none of the hazards selected for correlation

EI-MS matched molecular weight (relative abundance: 5.09%) for CC(=O)O

GHS hazard classification: none of the hazards selected for correlation

NN-rank 12: ['CC=O', 'CCCO']

EI-MS matched molecular weight (relative abundance: 99.99%) for CC=O

GHS hazard classification: Health Hazard, Irritant

EI-MS matched molecular weight (relative abundance: 5.09%) for CCCO

GHS hazard classification: Irritant

NN-rank 13: ['CC1CO1', 'CC=O']

EI-MS matched molecular weight (relative abundance: 99.99%) for CC=O

GHS hazard classification: Health Hazard, Irritant

NN-rank 14: ['C', 'CCCOC=O']

NN-rank 15: ['CCCOCC=O']

NN-rank 16: ['CCC', 'O=CCO']

EI-MS matched molecular weight (relative abundance: 99.99%) for CCC

GHS hazard classification: none of the hazards selected for correlation

EI-MS matched molecular weight (relative abundance: 5.09%) for O=CCO

GHS hazard classification: Irritant

NN-rank 17: ['CCCOC(C)=O']

NN-rank 18: ['CC(C)O', 'CC=O']

EI-MS matched molecular weight (relative abundance: 5.09%) for CC(C)O

GHS hazard classification: none of the hazards selected for correlation

EI-MS matched molecular weight (relative abundance: 99.99%) for CC=O

GHS hazard classification: Health Hazard, Irritant

NN-rank 19: ['C', 'C=O', 'CCCO']

EI-MS matched molecular weight (relative abundance: 5.09%) for CCCO

GHS hazard classification: Irritant

NN/MS matches corresponding with tox GHS classification highlight in Bold font.

NN-rank 20: ['CC(C)=O', 'CCO']

NN-rank 21: ['CC=O', 'CCOC']

EI-MS matched molecular weight (relative abundance: 99.99%) for CC=O

GHS hazard classification: Health Hazard, Irritant

EI-MS matched molecular weight (relative abundance: 5.09%) for CCOC

GHS hazard classification: none of the hazards selected for correlation

NN-rank 22: ['CCC', 'O=C1CO1']

EI-MS matched molecular weight (relative abundance: 99.99%) for CCC

GHS hazard classification: none of the hazards selected for correlation

NN-rank 23: ['C=CO', 'CCCO']

EI-MS matched molecular weight (relative abundance: 99.99%) for C=CO

GHS hazard classification: Acute Toxic

EI-MS matched molecular weight (relative abundance: 5.09%) for CCCO

GHS hazard classification: Irritant

NN-rank 24: ['CC(=O)OC1CC1']

NN-rank 25: ['CCOCCCO']

NN/MS matches corresponding with tox GHS classification highlight in Bold font.

180. Benzoic Acid: C1=CC=C(C=C1)C(=O)O

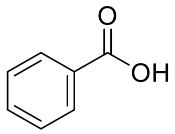

Summary Report of GHS Classification for NN/MS Matches:

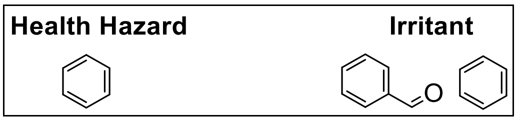

NN-rank 1: ['O', 'OCc1ccccc1']

NN-rank 2: ['O=C(O)c1ccccc1']

NN-rank 3: ['O', 'O=Cc1ccccc1']

EI-MS matched molecular weight (relative abundance: 99.99%) for O=Cc1ccccc1

GHS hazard classification: Irritant

NN-rank 4: ['O=CO', 'c1ccccc1']

EI-MS matched molecular weight (relative abundance: 66.59%) for c1ccccc1

GHS hazard classification: Health Hazard, Irritant

NN-rank 5: ['O', 'OCc1ccccc1']

NN-rank 6: ['O', 'O=C1C2=CC=C1C=C2']

NN-rank 7: ['O', 'O=c1c2ccccc12']

NN-rank 8: ['O', 'O=c1c2ccccc12']

NN-rank 9: ['O=CO', 'c1ccccc1']

EI-MS matched molecular weight (relative abundance: 66.59%) for c1ccccc1

GHS hazard classification: Health Hazard, Irritant

NN-rank 10: ['Cc1ccccc1', 'O', 'O']

NN-rank 11: ['O', 'O=Cc1ccccc1']

EI-MS matched molecular weight (relative abundance: 99.99%) for O=Cc1ccccc1

GHS hazard classification: Irritant

NN-rank 12: ['C=O', 'O', 'c1ccccc1']

EI-MS matched molecular weight (relative abundance: 66.59%) for c1ccccc1

GHS hazard classification: Health Hazard, Irritant

NN-rank 13: ['O=C1Oc2cccc1c2']

NN-rank 14: ['O=C1Oc2cccc1c2']

NN-rank 15: ['O=C1Oc2ccccc21']

NN/MS matches corresponding with tox GHS classification highlight in Bold font.

NN-rank 16: ['O=C1Oc2ccccc21']

NN-rank 17: ['O', 'OC1c2ccccc21']

EI-MS matched molecular weight (relative abundance: 99.99%) for OC1c2ccccc21

GHS hazard classification: none of the hazards selected for correlation

NN-rank 18: ['O', 'OC1c2ccccc21']

EI-MS matched molecular weight (relative abundance: 99.99%) for OC1c2ccccc21

GHS hazard classification: none of the hazards selected for correlation

NN-rank 19: ['O', 'O=C1C2=CC=C1C=C2']

NN-rank 20: ['O', 'OC1C2=CC=C1C=C2']

EI-MS matched molecular weight (relative abundance: 99.99%) for OC1C2=CC=C1C=C2

GHS hazard classification: none of the hazards selected for correlation

NN-rank 21: ['O', 'O=c1c2ccccc12']

NN-rank 22: ['O', 'O=c1c2ccccc12']

NN-rank 23: ['O=C1Oc2ccccc21']

NN-rank 24: ['O=C1Oc2ccccc21']

NN-rank 25: ['O', 'OCc1ccccc1']

NN/MS matches corresponding with tox GHS classification highlight in Bold font.
